# Sulfenofunctionalization of Chiral α‐Trifluoromethyl Allylboronic Acids: Asymmetric Synthesis of SCF_3_, SCF_2_R, SCN and SAr Compounds

**DOI:** 10.1002/anie.202210509

**Published:** 2022-10-17

**Authors:** Qiang Wang, Tomas Nilsson, Lars Eriksson, Kálmán J. Szabó

**Affiliations:** ^1^ Department of Organic Chemistry Stockholm University SE-10691 Stockholm Sweden; ^2^ Department of Materials and Environmental Chemistry Stockholm University SE-10691 Stockholm Sweden

**Keywords:** Asymmetric Synthesis, Boron, Fluorine, Lewis Base, Sulfur

## Abstract

We report herein a new method for the synthesis of densely functionalized chiral allyl SCF_3_, SCF_2_R, SCN and SAr species with a separate CF_3_ functionality. The synthetic approach is based on selenium‐catalyzed sulfenofunctionalization of chiral α‐CF_3_ allylboronic acids. The reactions proceeded with remarkably high stereo‐, diastereo‐ and site‐selectivity, based on the formation of a stable thiiranium ion followed by rapid deborylative ring opening.

## Graphical abstract

Densely functionalized chiral allyl species, with an alkenyl CF_3_ group, were prepared with excellent selectivity by selenium‐catalyzed sulfenofunctionalization of allylboronic acids. The method can be used to access chiral allyl SCF_3_, SCF_2_R, SCN and SAr compounds.

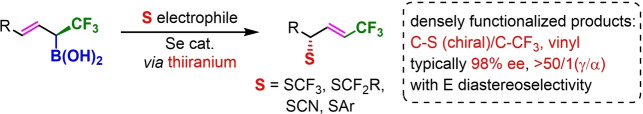

## Introduction

**Scheme 1:**
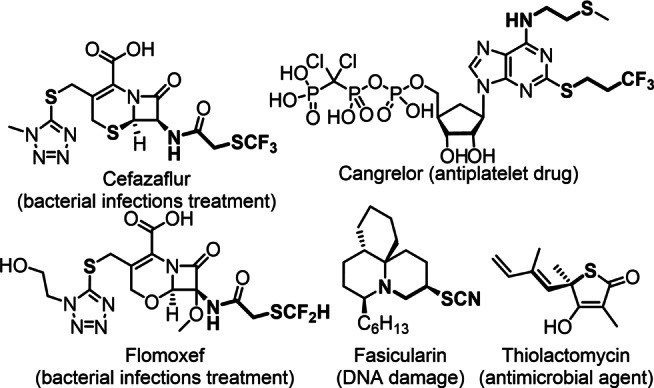
Examples of organosulfur drugs and natural products.

Sulfur and fluorine are major constituents in drug substances together with the principal elements of C, H, N, O.^[1]^ Sulfur containing drugs are used for treatment of bacterial infections (Scheme 1), diabetes, cancer, AIDS and many other clinical conditions.[^1^, ^2^] About 30 % of the recently approved pharmaceuticals contain at least one carbon‐fluorine bond.^[3]^ The beneficial pharmacokinetical properties of fluorinated bioactive compounds include high metabolic stability, usually low polarity and possibilities to alter the acid‐base properties of the drug substances.^[4]^ Thus, an increasing number of drug substances expected to appear, which contain both sulfur and fluorine.

**Scheme 2:**
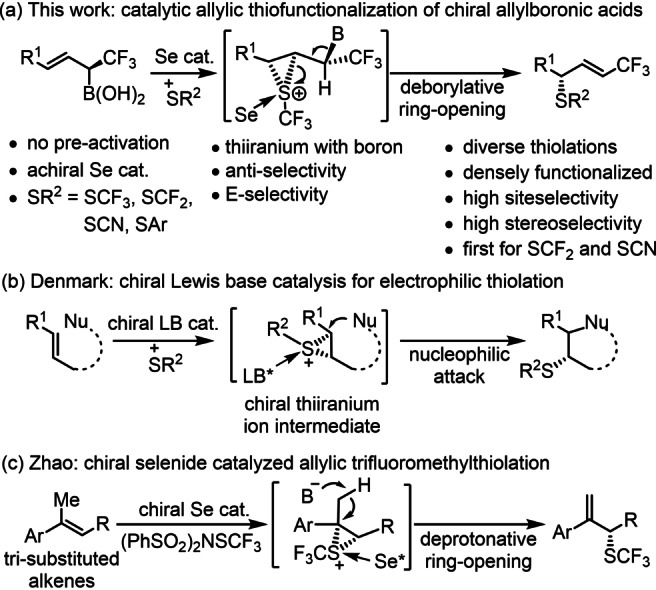
Asymmetric electrophilic thiolation reactions of alkenes.

Today there is a large interest in application of di‐ and trifluoromethylthiol containing drugs, such as Cefazaflur, Cangrelor and Flomoxef (Scheme 1).[^1^, ^3d^] However, considering that a trifluoromethyl group has excellent properties for modification of the polarity, solubility and metabolic stability of the drug substances, the simultaneous presence of CF_3_ and sulfur containing groups (SCF_3_, SCF_2_R, SCN, SR etc.) is an attractive approach for design of modern pharmaceuticals with complex bioactivity and fine‐tuned pharmacokinetic properties. Carbon‐sulfur^[5]^ and carbon‐fluorine^[6]^ bond formation reactions have been the subject of many excellent synthetic studies because of the high importance of these principal motifs in drug design. Among these methods the most challenging ones are directed to synthesis of chiral C−S/C−F motifs, which are important for the bioactivity of the drug substances.[^5^, ^6c^, ^7^] In this paper we report our results on asymmetric synthesis of chiral sulfur compounds (SCF_3_, SCF_2_R, SCN, SR etc.) comprising a separate CF_3_ group (Scheme 2a). This synthesis can be achieved by thiofunctionalization of allylic α‐CF_3_ boronic acids, which are available via a recently reported simple and efficient organocatalytic method.^[8]^ This procedure leads to trifluoromethylated chiral allyl sulfides comprising an alkene unit, which is a useful handle for further asymmetric functionalizations.

## Results and Discussion

Several excellent transition metal catalyzed allylic thiofunctionalization methods have been reported for synthesis of chiral allyl sulfides.^[9]^ Although, palladium catalysis can be employed for stereoselective transformation of allylboron compounds,^[10]^ the presence of an α‐CF_3_ group requires application of other synthetic approaches. The reason is that transmetallation of the boronate functionality with transition metals, such as Pd, leads to formation of an M−L bond in α‐position to the CF_3_ group, triggering a β‐fluorine elimination due to the M−C(σ) → C−F(σ*) anomeric effect.^[11]^

To avoid this anomeric β‐defluorination, the buildup of the negative charge at the stereogenic carbon has to be avoided. Thus, we selected an electrophilic thiofunctionalization method proceeding via Lewis base stabilized thiiranium ion intermediates.[^5c^, ^12^] This methodology has been pioneered by Denmark and co‐workers[^5c^, ^12^, ^13^] (Scheme 1b) and further developed by the Zhao group^[14]^ and others^[15]^ (Scheme 2c). This approach has been demonstrated to be highly efficient for synthesizing various aryl, alkyl and trifluoromethyl thioethers with high selectivity.

Initially we targeted the asymmetric synthesis of allylic SCF_3_ compounds with CF_3_ substituent (**3 a**) using α‐CF_3_ allylboronic acid derivatives^[8]^ **1 a** (Table 1). As a source of the sulfenyl group we used electrophilic SCF_3_ transfer reagent **2 a** reported independently by Zhao^[14b]^ and Shen.^[16]^ Zhao and co‐workers[^14a^, ^14b^, ^14c^, ^14d^] reported several trfluoromethylthiolation reactions by **2 a** (Scheme 2c) using selenium based Lewis base catalysts in the presence of strong acids (such as Tf_2_NH, TMSOTf etc.). We used commercially available diphenyl selenide as catalyst in the presence of various acids to activate **2 a**. Using pinacol boronate **1 a‐Bpin** and **2 a** as substrates in the presence of Ph_2_Se and Tf_2_NH at 25 °C resulted in allyl‐SCF_3_ product **3 a** in 8 % NMR yield (entry 1). In this reaction **1 a‐Bpin** was completely consumed. The low yield indicated that the thiiranium intermediate of the process was probably unstable at this temperature. Decrease of the temperature to 0 °C and −20 °C led to improvement of the yields to 23 and 31 %, respectively (entries 2, 3). However, further decrease of the temperature to −50 °C lowered the yield to 21 % (entry 4). Variation of the acidic additives by using TfOH or TMSOTf led to 30 and 23 % yields, respectively (entries 5 and 6).

**Table 1:** Optimization of the reaction conditions.^[a]^

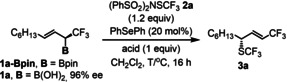					
Entry	Substrate	Acid	*T* [°C]	Yield [%]^[b]^	ee/es [%]^[c]^
1	**1 a‐Bpin**	Tf_2_NH	25	8	–
2	**1 a‐Bpin**	Tf_2_NH	0	23	–
3	**1 a‐Bpin**	Tf_2_NH	−20	31	–
4	**1 a‐Bpin**	Tf_2_NH	−50	21	–
5	**1 a‐Bpin**	TfOH	−20	30	–
6	**1 a‐Bpin**	TMSOTf	−20	23	–
7	**1 a**	Tf_2_NH	−20	55	–
8^[d]^	**1 a**	Tf_2_NH	−20	60	–
9^[d,e]^	**1 a**	Tf_2_NH	−20	80	–
10^[d,e]^	**1 a**	No acid	−20	24	–
11^[d,e,f]^	**1 a**	Tf_2_NH	−20	0	–
**12^[d,e,g]^**	**1 a**	**Tf_2_NH**	**−20**	**81(63)^[h]^**	**98/100**

[a] Unless otherwise stated: **1 a** (0.05 mmol), **2 a** (0.06 mmol), Ph_2_Se (20 mol %) and acid (0.05 mmol) dissolved in CH_2_Cl_2_ (0.5 mL) and stirred at indicated temperature for 16 h. [b] Determined by ^19^F NMR. [c] Determined by chiral GC. [d] 1.5 equivalent of **2 a**. [e] 3 Å Molecular sieves was used. [f] Reaction without selenide catalyst. [g] 0.3 mmol scale. [h] Isolated yield, regioselectivity: γ/α>50/1.

The above results indicated that Bpin is not a sufficiently reactive leaving group in the desired sulfenofunctionalization process. A possible way of activation of Bpin would be the application of ArLi reagents, which was employed by Aggarwal^[17]^ and Denmark^[13d]^ in sulfenofunctionalization of Bpin derivatives. However, we had to avoid this activation method, as it involves conversion of the Bpin group to a hypervalent ate complex. Formation of the negatively charged ate complex would lead to β‐fluoride elimination (see above) and possibly other undesired side reactions. Instead we opted to use of allylboronic acid^[8]^ **1 a** instead of the Bpin derivative (**1 a‐Bpin**). Our previous allylboration studies indicated that allylboronic acids or the corresponding boroxines are much more reactive species than the corresponding allyl‐Bpin reagents.[^8^, ^18^] Indeed, replacement of **1 a‐Bpin** with **1 a**, under otherwise identical reaction conditions, led to a major increase of the yield from 31 to 55 % (c.f. entries 3 and 7). When the amount of **2 a** was increased to 1.5 equiv. the yield was slightly increased to 60 % (entry 8). A substantial increase of the yield (to 80 %) was observed by adding molecular sieves (entry 9). Previous studies indicate that addition of the molecular sieves to allylboronic acids leads to formation of the corresponding allylboroxines.[^18c^, ^18d^] Allylboroxines are stronger Lewis acids than the corresponding allylboronic acids, and thus the reactivity of allylboronic acids is increased by boroxine formation under anhydrous conditions.[^18c^, ^18d^] Apparently, this is also the case for selenium‐catalyzed sulfenylation reactions, as the yield of **3 a** was improved from 60 % to 80 % by addition of molecular sieves (c.f. entries 8 and 9). Activation of **2 a** by Tf_2_NH is apparently important^[14b]^ as in the absence of acids, the yield is poor (24 %, entry 10). In the absence of Ph_2_Se catalyst formation of SCF_3_ product **3 a** was not observed at all (entry 11). When the reaction was scaled up to 0.3 mmol, the NMR yield was 81 % and the **3 a** could be isolated in 63 % yield. The isolation loss was due to the high volatility of **3 a**, which can be explained by the simultaneous presence of the CF_3_ and SCF_3_ groups. The reaction proceeded with excellent stereoselectivity with 98 % ee, which corresponds to a full chirality transfer (100 % es). In addition, the reaction occurred with high site selectivity, as we observed exclusively the formation of the γ‐SCF_3_ product, which occurred by allylic rearrangement. In the selenium catalyzed asymmetric trifluoromethylthiolation of tri‐substituted alkenes (c.f. Scheme 2c), the diastereoselectivity is difficult to control.^[14a]^ Yet, in the present reaction exclusively the E isomer of alkenyl trifluoromethyl compound was formed.

**Scheme 3:**
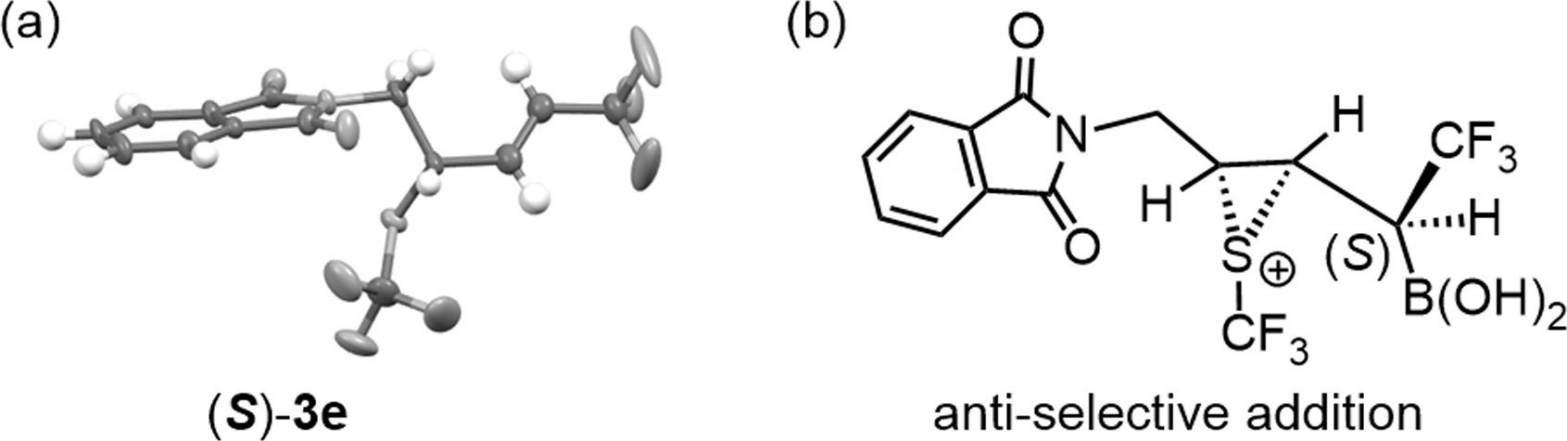
a. X‐ray structure of compound **3 e**. b. Suggested structure of the thiiranium ion intermediate for formation of **3 e**.

With the optimal conditions (Table 1, entry 12) in hand, the synthetic scope of the trifluoromethylthiolation reaction was studied using allylboronic acids **1 a**–**h** as substrates (Table 2, entries 1–8). Notably, a very high, stereo‐, diastereo‐ and site selectivity was observed as for trifluoromethylthiolation of **1 a** (Table 1, entry 12). The reactions usually proceeded with a high degree of functional group tolerance. Replacement of the alkyl moiety (**1 a**) with a benzyl one (**1 b**) did not alter the outcome of the reaction (c.f. entries 1 and 2). The chloro substituent in **1 c** was tolerated maintaining the high diastereo‐ and site‐selectivity but the ee dropped to 88 % (entry 3). The tosylester group of **1 d** remained unchanged in the reaction, resulting in **3 d** with very high selectivity offering two handles for further derivatizations. Phthalimide derivative **1 e** was converted to **3 e** (entry 5), which contains all six principal elements of drug design, with great opportunities for further asymmetric transformations. In this process, formation of traces of the α‐SCF_3_ product (γ/α=16/1) could also be detected. The absolute configuration of crystalline **3 e** was determined to be (S) by X‐ray diffraction (Scheme 3a).^[19]^ Considering the structural similarities and reaction conditions, we tentatively assigned the configuration of the other thiofunctionalized products based on the absolute configuration of **3 e**. In the presence of an alkenyl cyclohexyl group (**1 f**), the high selectivity could be still maintained (entry 6). Selective sulfenofunctionalization of cinnamylboronic acid **1 g** is expected to be challenging. Considering the highly selective formation of **3 f** (entry 6), we predicted a high diastereo‐ and stereoselectivity in case of replacement of the cyclohexyl (**1 f**) with phenyl (**1 g**) group. However, the reactivity and the site selectivity were anticipated to decrease, because of the allylic rearrangement, which disrupts the π‐conjugation between the phenyl group and the double bond in **1 g**. Initial attempts using the standard optimized conditions gave **3 g** with low yield. However, changing the catalyst to (*p*‐OMePh)_2_Se and using MsOH instead of Tf_2_NH as activator **3 g** was formed in acceptable yield (53 %) and excellent siteselectivity. We also attempted to obtain substituted analogs of **3 g**. However, when **1 g** with p‐chloro phenyl group was used, we obtained an intractable mixture of products, probably because of the instability of the allylboronic acid under the applied reaction conditions. On the other hand, the p‐methoxy phenyl analog of **1 g** had a very low reactivity towards trifluoromethylthiolation, as we got only traces of the product. Compound **1 h** with bulky tert‐butyl group was converted to product **3 h** under the standard conditions (entry 8). The reactions proceeded with excellent stereo‐, diastereo‐ and site selectivity in 64 % yield indicating that bulky alkenyl substituents did not affect the selectivity of the reaction.

**Table 2:**
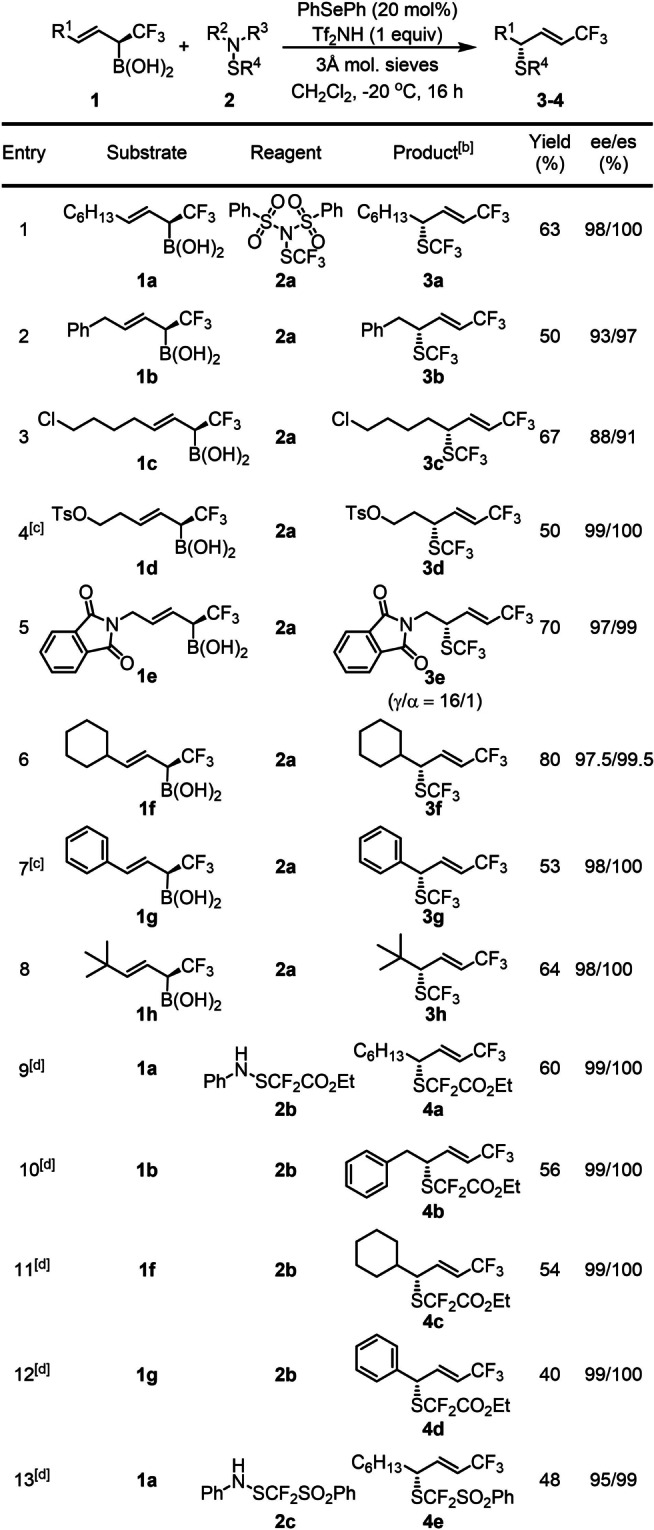
Substrate scope for trifluoromethylthiolation and difluoromethylthiolation.^[a]^

[a] Unless otherwise stated: **1** (0.3 mmol), **2** (0.45 mmol), Ph_2_Se (20 mol %) and Tf_2_NH (0.3 mmol) dissolved in CH_2_Cl_2_ (3 mL) and stirred at −20 °C for 16 h. [b] Unless otherwise stated: γ/α>50/1. [c] Reaction was performed with (*p*‐OMePh)_2_Se as catalyst, MsOH as additive at −50 °C for 48 h. [d] Reaction was performed on 0.1 mmol scale with (*p*‐OMePh)_2_Se (20 mol %) as catalyst and MsOH as additive.

Difluoromethylthio motif is a rising star in modern drug design. For example Flomoxef has slightly different antibacterial activity compared to the SCF_3_ analogue Cefazaflur (Scheme 1).[^1^, ^3d^] In addition, difluoromethyl groups are bioisosteres for hydroxy and thiol functionalities.^[20]^ Asymmetric electrophilic difluoromethylthiolation reactions based on Lewis‐base catalysis have never been reported. Synthesis of chiral SCF_2_R species was not described either. As far as we know, only two studies have been published on asymmetric synthesis of SCF_2_H species by the groups^[21]^ of Cahard and Shibata and the Shen group.^[22]^ The success of the above asymmetric trifluoromethylthiolation reactions inspired us to extend the same concept to the highly challenging difluoromethylthiolation reactions. In these studies, we used electrophilic, aniline based difluoromethyltiolation reagents **2 b**–**c** (entries 9–13) reported by Billard and co‐workers.^[23]^ Because of using these SCF_2_ sources, we slightly modified the reaction conditions used for the SCF_3_ transfer reactions. Thus, we used electron‐rich (*p*‐OMePh)_2_Se catalyst, which is more efficient (stronger Lewis base) than Ph_2_Se. In addition, an excess of acid additive was required to inhibit the competitive nucleophilic amination by aniline. Aliphatic allylboronic acid derivative **1 a** gave the chiral SCF_2_COOEt product **4 a** with full allylic rearrangement, in good yield (60 %) and with remarkably high stereo‐, diastereo‐ and site‐selectivities (entry 9). Slightly lower yields but still very high selectivity were observed for benzyl and cyclohexyl allylboronic acid substrates **1 b** and **1 f** (entries 10–11). Cinnamylboronic acid **1 g** was reacted with **2 b** to give benzylic difluoromethylthiolated product **4 d** with excellent γ‐siteselectivity in 40 % yield (entry 12). The sulfone‐containing reagent **2 c** reacted with **1 a** in somewhat lower yield and with slightly lower selectivity than the carbethoxy analogue **2 b** (c.f. entries 9 and 13).

Considering that the selenium catalyzed transformation of allylboronic acids proceeded with usually good yield and remarkably high selectivity for synthesis of densely functionalized chiral di‐ and trifluoromethyl thiols **3 a**–**h** and **4 a**–**e**, we sought to extent the concept to other sulfenofunctionalization reactions. Chiral thiocyanates (Scheme 1) such as Fasicularin^[24]^ attracted a considerable attention in medicinal applications. An obvious reason is that organic thiocyanates can be easily converted into various sulfur‐containing drug intermediates especially to sulfur‐heterocycles.^[25]^ One of the problems in wide application of this functionality in modern drug design is that very few methods are available for asymmetric synthesis of organic thiocyanates. Zhao and co‐workers reported a selenium catalyzed asymmetric thiocyanation method but this reaction occurred with poor enantioselectivity.^[14d]^ We used saccharin based electrophilic SCN transfer reagent **2 d** in the presence of (*p*‐OMePh)_2_Se catalyst in the thiocyanation of allylboronic acids (Table 3, entries 1–3). Using aliphatic allylboronic acid substrate **1 a** with reagent **2 d**, the corresponding densely functionalized thiocyanate **5 a** was formed in high yield (81 %) and excellent stereo‐, diastereo‐ and site‐selectivity (entry 1). Phthalimide derivative **1 e** gave a homoallyl amine **5 b** with SCN and CF_3_ functionalities (entry 2) with still high ee (90 %) and high γ‐selectivity (γ/α=32/1). As expected from the above thiofunctionalization reactions cyclohexyl substrate **1 f** gave the corresponding SCN product with allylic rearrangement and with high level of chirality transfer (entry 3).

**Table 3:**
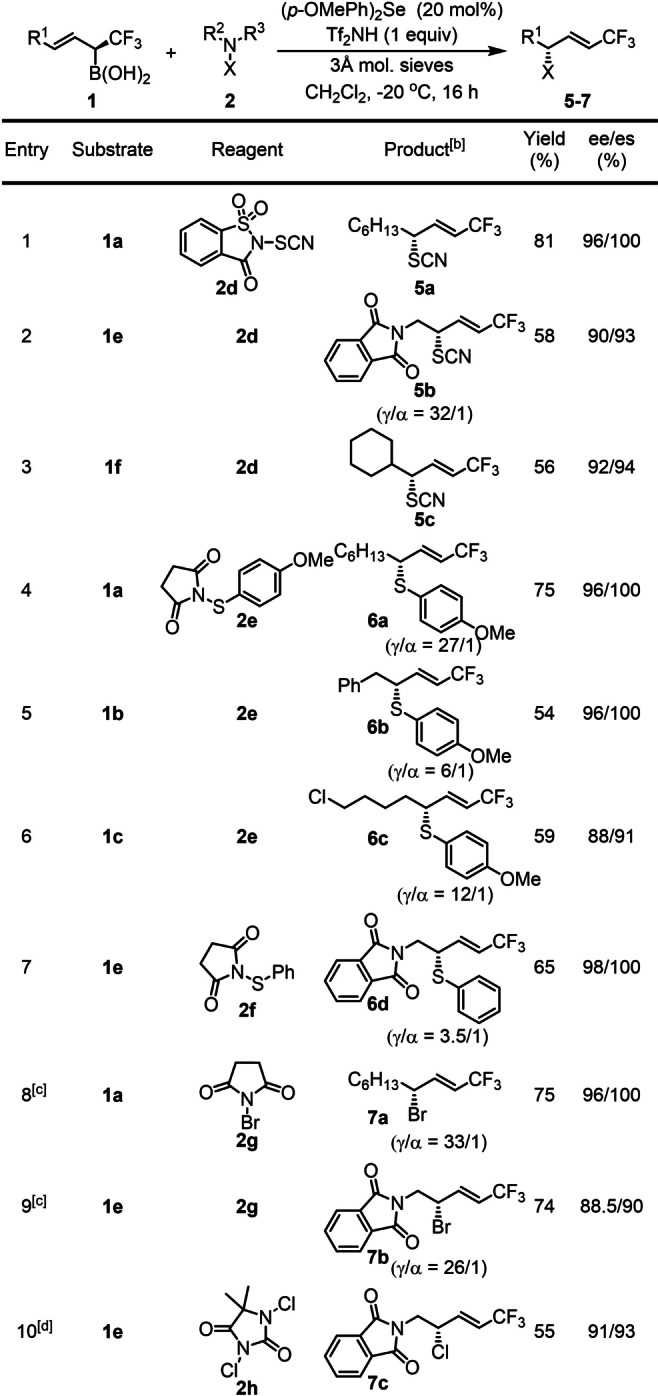
Substrate scope for thiocyanation, arylthiolation and halogenation.^[a]^

[a] Unless otherwise stated: **1** (0.1 mmol), **2** (0.15 mmol), (*p*‐OMePh)_2_Se (20 mol %) and Tf_2_NH (0.1 mmol) dissolved in CH_2_Cl_2_ (1 mL) and stirred at −20 °C for 16 h. [b] Unless otherwise stated: γ/α>50/1. [c] With Ph_2_Se as catalyst. [d] With Ph_2_S as catalyst.

We have also studied the arylthiolation of α‐CF_3_ allylboronic acids (entries 4–7). In these reactions we employed succinimide based reagents **2 e** and **2 f**. The stereo‐, diastereo‐ and siteselectivity was high for reaction of aliphatic allylboronic acid derivative **1 a** and **2 e** (entry 4). However, a major drop of the site‐selectivity occurred for the reaction of benzyl derivative **1 b** and **2 e** as allylic aryl sulfide **6 b** was formed with an γ/α ratio of 6/1 (entry 5). The site selectivity for sulfenofunctionalization of **1 c** was high (γ/α=12/1) but the ee dropped to 88 % (entry 6). The lowest site selectivity (γ/α=3.5/1) was obtained for thiofunctionalization of phthalimide substrate **1 e** (entry 7). Apparently, the site selectivity is significantly lower in arylthiolation, than in transfer of other groups with electron deficient sulfur (SCF_3_, SCF_2_R or SCN).

Considering the successful thiofunctionalization reactions with succinimide derivatives **2 e**–**f** and the similar chemical behavior of halogenides and SCN groups (also called pseudohalogenides), we also extended the electrophilic substitution of allylboron species to chlorination and bromination reactions (entries 8–10). In these processes, we used standard electrophilic halogenation reagents **2 g**–**h**. The reactions with aliphatic (**1 a**) and phthalimide (**1 e**) derivatives proceeded with high selectivities. The chiral allylic bromides (**7 a**–**b**) and chloride (**7 c**) with CF_3_ group are very useful building blocks in asymmetric synthesis. We have also attempted to extend this reaction to fluorination and iodination. The reaction of **1 a** under standard conditions with NFSI and NIS gave complex reaction mixtures from which we were not able to isolate halogenated products.

**Scheme 4:**
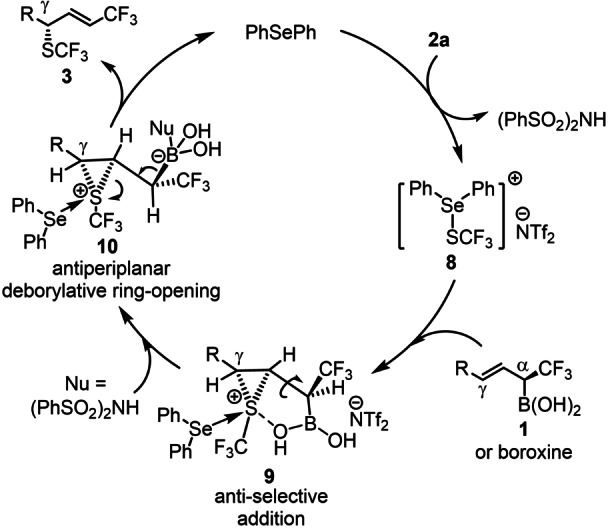
Plausible mechanism for the allylic trifluoromethylthiolation of allyl boronic acid.

Based on the above results and the literature reports,[^5c^, ^14a^] we suggest a mechanism for the selenium catalyzed sulfenofunctionalization of chiral α‐CF_3_ allylboronic acids exemplified with trifluoromethylthiolation by **2 a** (Scheme 4). The catalytic cycle is initiated by acid catalyzed^[14b]^ formation of **8** from the diphenyl selenide catalyst and **2 a**. Reagent **8** is apparently more reactive than **2 a** as trifluoromethylthiolation of **1 a** did not occur with **2 a** in the absence of Ph_2_Se (Table 1, entry 11). The next step is formation of chiral thiiranium ion **9** by the reaction of **8** and allylboronic acid (or its boroxine) **1**. This is the stereoselectivity determining step. As the absolute configuration of the stereogenic center in **1 e** is (S),^[8]^ the (S) configuration in **3 e** (Scheme 3a) suggests that addition of the sulfenium ion to the double bond occurs anti to the CF_3_ group (Scheme 3b). This geometry also leads to formation of the CF_3_‐alkene moiety (**3**) with a high E‐selectivity. The observed high selectivities can be explained by an interaction involving the sulfur of the thiiranium ring and an oxygen atom of the boronate group in **9**.

A thiiranium ion is usually thermally unstable and it may undergo isomerization reactions.[^5c^, ^14a^, ^26^] Strong carbon‐sulfur bonds in thiiranium ions lead to high configurational stability. An explanation of the high level of chirality transfer in the presented sulfenylation reactions may be that the thiiranium ions formed are relatively stable intermediates. A possible stabilizing factor is the presence of the CF_3_ group, which would destabilize a carbocationic intermediate formed by opening of the thiiranium ion. Yet, slight drops of the chirality transfer (Table 2, entry 3, Table 3 entries 2, 6) may be explained by minor stability issues of the thiiranium ion. The decrease of the site‐selectivity is a bit more difficult to explain. We had site selectivity issues mainly in case of arylthiolation reactions (Table 3 entries 5 and 7). The steric hindrance by the arylthiol group may contribute to the slow formation/low stability of the thiiranium ion.^[13e]^ This instability may be coupled with rearrangement of the thiiranium ion^[27]^ leading to a partial formation of the α‐substituted allyl product. The next step in the catalytic cycle is probably activation of the boronate group of **9** for elimination. This may occur by coordination of a nucleophile present in the reaction mixture (such as (PhSO_2_)_2_NH) to form intermediate **10**. Due to the acidic conditions (presence of Tf_2_NH) the nucleophilicity of (PhSO_2_)_2_NH is relatively poor. Therefore, it is important that the boron atom is highly Lewis acidic, such as in B(OH)_2_ or boroxine groups. Otherwise, the elimination of boronate is very slow and the thiiranium ion may undergo rearrangements or decomposition. This could be the explanation of the low reactivity/poor yield of **1 a‐Bpin** substrate in the above trifluoromethylthiolation reactions (Table 1, entries 1–6). The deborylative opening of the thiiranium ion in **10** leads to formation of **3**, which is preceded by folding the Cα‐Cβ bond to ensure the formation of the alkene unit with E‐selectivity. In addition, the Cα‐Cβ bond rotation renders Cα‐B and Cβ‐S bonds to antiperiplanar position. This geometry allows occurring a deborylative ring‐opening by interaction of the Cα‐B(σ) and Cβ‐S(σ*) MOs. The highly selective formation of the chiral allyl‐SCF_3_ product with CF_3_ group (**3**) leads to regeneration of the Ph_2_Se catalyst.

## Conclusion

We have developed a highly stereo‐, diastereo‐ and site‐selective method for sulfenofunctionalization of α‐CF_3_ allylboronic acids. This method is suitable for synthesis of densely functionalized SCF_3_, SCF_2_R, SCN and SAr compounds containing a CF_3_ group and one or more handles for further stereoselective derivatization. The stereo‐ and diastereoselectivity of the reaction is remarkably high for all substrates and the allylic γ‐site selectivity is also very high for most of the substrates and thiofunctional groups. Very few methods are available in the literature for introduction of chiral SCF_2_R and SCN groups and synthesis of allylthio derivatives in the presence of alkenyl CF_3_ group is also very limited. The presented synthetic concept can also be extended to preparation of allylic chlorides and bromides. A possible explanation of the high selectivity and broad synthetic scope is the intermediacy of a relatively stable thiiranium ion, which undergoes rapid deborylative ring opening in case of application of allylboronic acid/boroxine substrates. We hope that the above method can be readily used in designing multifunctional drugs comprising all the 6 fundamental elements (C, H, O, N, S, F) and synthesis of sulfur containing natural products.[^1^, ^2^, ^3^, ^5a^, ^5b^, ^5c^, ^7^]

## Conflict of interest

The authors declare no conflict of interest.

1

## Supporting information

As a service to our authors and readers, this journal provides supporting information supplied by the authors. Such materials are peer reviewed and may be re‐organized for online delivery, but are not copy‐edited or typeset. Technical support issues arising from supporting information (other than missing files) should be addressed to the authors.

Supporting Information

Supporting Information

## Data Availability

The data that support the findings of this study are available in the Supporting Information of this article.

## References

[1] K. A. Scott , J. T. Njardarson , Top. Curr. Chem. 2018, 376, 5. 10.1007/s41061-018-0184-5 29356979

[2] E. A. Ilardi , E. Vitaku , J. T. Njardarson , J. Med. Chem. 2014, 57, 2832–2842. 24102067 10.1021/jm401375q

[3a] H. Mei , J. Han , S. Fustero , M. Medio-Simon , D. M. Sedgwick , C. Santi , R. Ruzziconi , V. A. Soloshonok , Chem. Eur. J. 2019, 25, 11797–117819; 31099931 10.1002/chem.201901840

[3b] Y. Zhou , J. Wang , Z. Gu , S. Wang , W. Zhu , J. L. Aceña , V. A. Soloshonok , K. Izawa , H. Liu , Chem. Rev. 2016, 116, 422–518; 26756377 10.1021/acs.chemrev.5b00392

[3c] J. Wang , M. Sánchez-Roselló , J. L. Aceña , C. del Pozo , A. E. Sorochinsky , S. Fustero , V. A. Soloshonok , H. Liu , Chem. Rev. 2014, 114, 2432–2506; 24299176 10.1021/cr4002879

[3d] M. Inoue , Y. Sumii , N. Shibata , ACS Omega 2020, 5, 10633–10640. 32455181 10.1021/acsomega.0c00830 PMC7240833

[4a] D. O'Hagan , Chem. Soc. Rev. 2008, 37, 308–319; 18197347 10.1039/b711844a

[4b] A. Rodil , S. Bosisio , M. S. Ayoup , L. Quinn , D. B. Cordes , A. M. Z. Slawin , C. D. Murphy , J. Michel , D. O'Hagan , Chem. Sci. 2018, 9, 3023–3028. 29732086 10.1039/c8sc00299a PMC5916015

[5a] T. Toru , C. Bolm , Organosulfur Chemistry in Asymmetric Synthesis, Wiley-VCH, Weinheim, 2008;

[5b] P. Chauhan , S. Mahajan , D. Enders , Chem. Rev. 2014, 114, 8807–8864; 25144663 10.1021/cr500235v

[5c] A. Matviitsuk , J. L. Panger , S. E. Denmark , Angew. Chem. Int. Ed. 2020, 59, 19796–19819; 10.1002/anie.202005920 PMC7936392 32452077

[5d] H. Chen , W. Jiang , Q. Zeng , Chem. Rec. 2020, 20, 1269–1296. 32930488 10.1002/tcr.202000084

[6a] D. Cahard , J.-A. Ma , Emerging Fluorinated Motifs: Synthesis, Properties, and Applications, Wiley-VCH, Weinheim, 2020;

[6b] K. J. Szabó , N. Selander , Organofluorine Chemistry: Synthesis, Modeling, and Applications, Wiley-VCH, Weinheim, 2021;

[6c] Y. Zhu , J. Han , J. Wang , N. Shibata , M. Sodeoka , V. A. Soloshonok , J. A. S. Coelho , F. D. Toste , Chem. Rev. 2018, 118, 3887–3964; 29608052 10.1021/acs.chemrev.7b00778 PMC6497456

[6d] T. Liang , C. N. Neumann , T. Ritter , Angew. Chem. Int. Ed. 2013, 52, 8214–8264; 10.1002/anie.201206566 23873766

[6e] R. Britton , V. Gouverneur , J.-H. Lin , M. Meanwell , C. Ni , G. Pupo , J.-C. Xiao , J. Hu , Nat. Rev MethodsPrimers. 2021, 1, 47;

[6f] S. Purser , P. R. Moore , S. Swallow , V. Gouverneur , Chem. Soc. Rev. 2008, 37, 320–330. 18197348 10.1039/b610213c

[7] E. Miller , F. D. Toste in Organofluorine Chemistry (Eds.: K. J. Szabo , N. Selander ), Wiley-VCH, Weinheim, 2021, pp. 241–280.

[8] S. J. T. Jonker , R. Jayarajan , T. Kireilis , M. Deliaval , L. Eriksson , K. J. Szabó , J. Am. Chem. Soc. 2020, 142, 21254–21259. 33270462 10.1021/jacs.0c09923 PMC7760092

[9a] M. Roggen , E. M. Carreira , Angew. Chem. Int. Ed. 2012, 51, 8652–8655; 10.1002/anie.201202092 22833433

[9b] X.-H. Yang , R. T. Davison , V. M. Dong , J. Am. Chem. Soc. 2018, 140, 10443–10446; 30091909 10.1021/jacs.8b06957 PMC6563822

[9c] H.-J. Gais , T. Jagusch , N. Spalthoff , F. Gerhards , M. Frank , G. Raabe , Chem. Eur. J. 2003, 9, 4202–4221; 12953206 10.1002/chem.200204657

[9d] S. Zheng , N. Gao , W. Liu , D. Liu , X. Zhao , T. Cohen , Org. Lett. 2010, 12, 4454–4457; 20863112 10.1021/ol101915b

[9e] N. Gao , S. Zheng , W. Yang , X. Zhao , Org. Lett. 2011, 13, 1514–1516. 21348506 10.1021/ol200197v

[10a] S. Sebelius , V. J. Olsson , O. A. Wallner , K. J. Szabó , J. Am. Chem. Soc. 2006, 128, 8150; 16787075 10.1021/ja062585o

[10b] Y. Yamamoto , S. Takada , N. Miyaura , Chem. Lett. 2006, 35, 1368–1369;

[10c] L. Chausset-Boissarie , K. Ghozati , E. LaBine , J. L. Y. Chen , V. K. Aggarwal , C. M. Crudden , Chem. Eur. J. 2013, 19, 17698–17701; 24302640 10.1002/chem.201303683

[10d] Y. Yang , S. L. Buchwald , J. Am. Chem. Soc. 2013, 135, 10642–10645; 23837686 10.1021/ja405950c PMC3776608

[10e] J. L. Farmer , H. N. Hunter , M. G. Organ , J. Am. Chem. Soc. 2012, 134, 17470–17473. 23046477 10.1021/ja308613b

[11] O. A. Argintaru , D. Ryu , I. Aron , G. A. Molander , Angew. Chem. Int. Ed. 2013, 52, 13656–13660; 10.1002/anie.201308036 PMC3904770 24214845

[12] S. E. Denmark , G. L. Beutner , Angew. Chem. Int. Ed. 2008, 47, 1560–1638; 10.1002/anie.200604943 18236505

[13a] S. E. Denmark , D. J. P. Kornfilt , T. Vogler , J. Am. Chem. Soc. 2011, 133, 15308–15311; 21859086 10.1021/ja2064395 PMC3187834

[13b] S. E. Denmark , A. Jaunet , J. Am. Chem. Soc. 2013, 135, 6419–6422; 23597174 10.1021/ja401867b PMC3675264

[13c] S. E. Denmark , H. M. Chi , J. Am. Chem. Soc. 2014, 136, 8915–8918; 24926794 10.1021/ja5046296 PMC4073881

[13d] Z. Tao , K. A. Robb , J. L. Panger , S. E. Denmark , J. Am. Chem. Soc. 2018, 140, 15621–15625; 30411878 10.1021/jacs.8b10288 PMC6345169

[13e] S. E. Denmark , E. Hartmann , D. J. P. Kornfilt , H. Wang , Nat. Chem. 2014, 6, 1056–1064; 25411883 10.1038/nchem.2109 PMC4677329

[13f] A. Matviitsuk , J. L. Panger , S. E. Denmark , ACS Catal. 2022, 12, 7377–7385. 10.1021/acscatal.2c01232 PMC9851372 36686398

[14a] X. Liu , Y. Liang , J. Ji , J. Luo , X. Zhao , J. Am. Chem. Soc. 2018, 140, 4782–4786; 29583000 10.1021/jacs.8b01513

[14b] X. Liu , R. An , X. Zhang , J. Luo , X. Zhao , Angew. Chem. Int. Ed. 2016, 55, 5846–5850; 10.1002/anie.201601713 27027644

[14c] J. Luo , Q. Cao , X. Cao , X. Zhao , Nat. Commun. 2018, 9, 527; 29410415 10.1038/s41467-018-02955-0 PMC5802806

[14d] W. Wei , L. Liao , T. Qin , X. Zhao , Org. Lett. 2019, 21, 7846–7850; 31525984 10.1021/acs.orglett.9b02834

[14e] Y. Liang , X. Zhao , ACS Catal. 2019, 9, 6896–6902;

[14f] Y. Zhang , Y. Liang , X. Zhao , ACS Catal. 2021, 11, 3755–3761;

[14g] J. Luo , Z. Zhu , Y. Liu , X. Zhao , Org. Lett. 2015, 17, 3620–3623. 26158564 10.1021/acs.orglett.5b01727

[15] H.-Y. Luo , Z.-H. Li , D. Zhu , Q. Yang , R.-F. Cao , T.-M. Ding , Z.-M. Chen , J. Am. Chem. Soc. 2022, 144, 2943–2952. 35143185 10.1021/jacs.1c09635

[16] P. Zhang , M. Li , X.-S. Xue , C. Xu , Q. Zhao , Y. Liu , H. Wang , Y. Guo , L. Lu , Q. Shen , J. Org. Chem. 2016, 81, 7486–7509. 27441822 10.1021/acs.joc.6b01178

[17] C. García-Ruiz , J. L. Y. Chen , C. Sandford , K. Feeney , P. Lorenzo , G. Berionni , H. Mayr , V. K. Aggarwal , J. Am. Chem. Soc. 2017, 139, 15324–15327. 29028321 10.1021/jacs.7b10240 PMC5682599

[18a] R. Alam , C. Diner , S. Jonker , L. Eriksson , K. J. Szabó , Angew. Chem. Int. Ed. 2016, 55, 14417–14421; 10.1002/anie.201608605 PMC5129484 27735124

[18b] R. Alam , T. Vollgraff , L. Eriksson , K. J. Szabó , J. Am. Chem. Soc. 2015, 137, 11262–11265; 26316158 10.1021/jacs.5b07498

[18c] M. Raducan , R. Alam , K. J. Szabó , Angew. Chem. Int. Ed. 2012, 51, 13050–13053; 10.1002/anie.201207951 PMC3556694 23161757

[18d] G. Huang , C. Diner , K. J. Szabó , F. Himo , Org. Lett. 2017, 19, 5904–5907. 29039676 10.1021/acs.orglett.7b02901

[19] Deposition Number 2173301 (for **3 e**) contains the supplementary crystallographic data for this paper. These data are provided free of charge by the joint Cambridge Crystallographic Data Centre and Fachinformationszentrum Karlsruhe Access Structures service.

[20] N. A. Meanwell , J. Med. Chem. 2011, 54, 2529–2591. 21413808 10.1021/jm1013693

[21] S. Gondo , O. Matsubara , H. Chachignon , Y. Sumii , D. Cahard , N. Shibata , Molecules 2019, 24, 221. 30634428 10.3390/molecules24020221 PMC6359606

[22] H. Zhang , X. Wan , Q. Shen , Chin. J. Chem. 2019, 37, 1041–1050.

[23a] E. Ismalaj , D. Le Bars , T. Billard , Angew. Chem. Int. Ed. 2016, 55, 4790–4793; 10.1002/anie.201601280 27037994

[23b] E. Ismalaj , Q. Glenadel , T. Billard , Eur. J. Org. Chem. 2017, 1911–1914.

[24] A. D. Patil , A. J. Freyer , R. Reichwein , B. Carte , L. B. Killmer , L. Faucette , R. K. Johnson , D. J. Faulkner , Tetrahedron Lett. 1997, 38, 363–364.

[25] T. Castanheiro , J. Suffert , M. Donnard , M. Gulea , Chem. Soc. Rev. 2016, 45, 494–505. 26658383 10.1039/c5cs00532a

[26] S. E. Denmark , T. Vogler , Chem. Eur. J. 2009, 15, 11737–11745. 19760721 10.1002/chem.200901377

[27a] Y.-Y. Xie , Z.-M. Chen , H.-Y. Luo , H. Shao , Y.-Q. Tu , X. Bao , R.-F. Cao , S.-Y. Zhang , J.-M. Tian , Angew. Chem. Int. Ed. 2019, 58, 12491–12496; 10.1002/anie.201907115 31293063

[27b] Q. Wang , M. Biosca , F. Himo , K. J. Szabo , Angew. Chem. Int. Ed. 2021, 60, 26327–26331; 10.1002/anie.202109461 PMC9299629 34613633

