# Supplementary material for: Sulfenofunctionalization of Chiral α‐Trifluoromethyl Allylboronic Acids: Asymmetric Synthesis of SCF3, SCF2R, SCN and SAr Compounds
Source: Angew Chem Int Ed Engl. 2022 Oct 17;61(46):e202210509. doi: 10.1002/anie.202210509 (PMC9828052; doi:10.1002/anie.202210509)
Supplement: Supplementary file 1 — Supporting Information [file ANIE-61-0-s001.pdf]

Angewandte Chemie
Eine Zeitschrift der Gesellschaft Deutscher Chemiker

Supporting Information

Sulfenofunctionalization of Chiral α-Trifluoromethyl Allylboronic Acids: Asymmetric Synthesis of SCF_3, SCF_2R, SCN and SAr Compounds

*Q. Wang, T. Nilsson, L. Eriksson, K. J. Szabó**

Table of contents:

1. General information…………......……………………………………………………………………2

2. Procedure for the synthesis of chiral allylboronic acids **1a-1g**……………………………………......3

3. General procedure A for the trifluoromethylthiolation…………………………………..……..…15

4. General procedure B for the difluoromethylthiolation…………………………………..…..……21

5. General procedure C for the thiocyanation…………………………………..………………...…25

6. General procedure D for the arylthiolation…………………………………...………………...…28

7. General procedure E for the halogenation…………………………………..………………...…..33

8. ^{1}H NMR, ^{13}C NMR, ^{19}F NMR and ^{11}B NMR spectra of substrates and products…………………..….36

9. Reference……………………………………………………………………………...…………..94

General Information

Reagents were used as obtained from commercial suppliers without further purification. *trans*-1-Octen-1-ylboronic acid, *trans*-3-phenyl-1-propen-1-ylboronic acid, *trans*-2-phenylvinylboronic acid, Ph_2Se, MsOH were obtained from Sigma-Aldrich. Triflimide was obtained from TCI. CH_2Cl_2 was first dried by a solvent purification system and further dried by 3 Å molecular sieves. Dry DME was purchased from Sigma-Aldrich. Flash chromatography was carried out using 60 Å (35-70 µm mesh) silica gel (VWR). Analytical TLC was carried out by aluminum-backed plates (1.5 Å, ~ 5 cm) pre-coated (0.25 mm) with silica gel (Merck, Silica Gel 60 F254). Compounds were visualized by exposure to UV light (254 nm) or by dipping the plates into a solution of 0.75% $KMnO_4$ (w/w) in an aqueous solution of K_2CO_3 0.36 M. ^{1}H NMR spectra were recorded at 400 MHz, ^{13}C NMR spectra were recorded at 100 MHz, ^{19}F NMR spectra were recorded at 377 MHz and ^{11}B NMR spectra were recorded at 128 MHz with a Bruker Advance spectrometer. ^{1}H and ^{13}C NMR chemical shifts (δ) are reported in ppm from tetramethylsilane, using the residual solvent resonance (^{1}H-NMR: δ_H = 7.26 ppm ($CDCl_3$) and in ^{13}C-NMR: δ_C = 77.16 ppm ($CDCl_3$) as internal references). The multiplicity is abbreviated as follows: s (singlet), d (doublet), t (triplet), q (quartet), p (pentuplet), m (multiplet) and br (broad). The ^{13}C carbon shifts for the boronated carbon (C-B) are obscured because of the nuclear quadrupole coupling with the boron atom. Coupling constants (*J*) are given in Hz. For air-sensitive allyl boronic acids **1a-1h**, only the ^{1}H and ^{19}F NMR spectra were recorded. High-resolution mass spectra (HRMS) were recorded with a Bruker microTOF ESI-TOF mass spectrometer in positive ion mode unless otherwise specified. Low-resolution mass spectra were obtained by GC-MS (EI, 70 eV). Enantiomeric excesses were determined by SFC (Supercritical-Fluid Chromatography, as mobile phase serve mixtures of MeOH / supercritical CO_2) using the following columns (150 mm length, 3 mm ϕ, 3 µm particle size): Daicel Chiral Technologies Chiralpak IB, Chrialpak OJ, Chiralpak IF, or using chiral GC (Chiraldex β-6TBDM, Chiraldex β-DM). Optical rotation was recorded on a thermostated polarimeter using sodium lamp (589 nm) and a 10 cm cell. Thiolation reagents **2a**,[1] **2b**,[2] **2c**,[3] **2d**,[4] **2e**,[5] **2f**[5] and vinyl boronic acids[6,7] were synthesized according to literature reports.

Procedure for the synthesis of chiral allylboronic acids 1a-1h

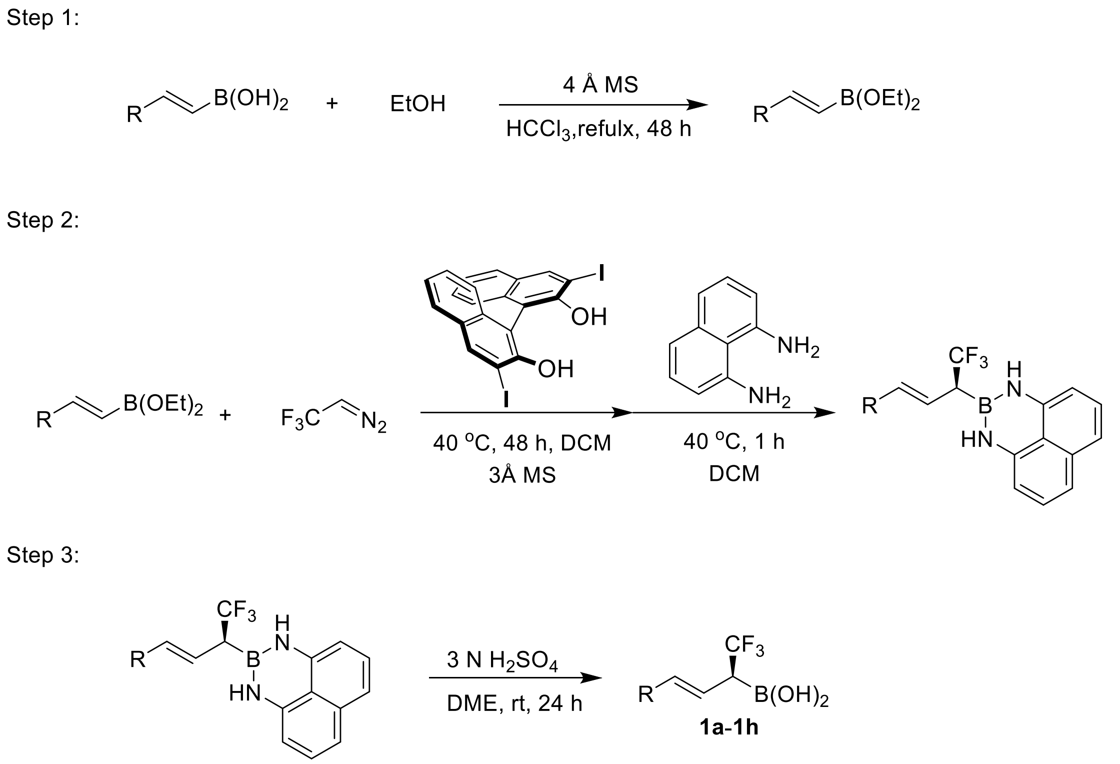

Scheme S1. Synthesis of allylboronic acids 1a-1h.

Step 1, vinylboronic acid (1 eq) was refluxed with EtOH (35 eq) and $CHCl_3$ (0.2 M) in a one-necked round-bottomed flask fitted with a soxhlet containing 4Å molecular sieves. After 48 hours, the excess solvent was removed in vacuo to provide the ethyl boronic ester, which was used without further purification.

Step 2 (Scheme S1) was furnished according to our previously reported method with some modifications.[8] An oven-dried screw cap reaction tube was charged with (*R*)-iodo-BINOL (20 mol%). The reaction tube was brought into the glovebox and vinyl boronic ester (1 eq), 3Å molecular sieves (20 mg per 0.1 mmol), CF_3-diazomethane (2.5 eq, prepared by Molander´s procedure[9]) in DCM were added sequentially. The concentration of vinyl boronic ester was maintained to be 0.125 M. The reaction tube was tightly screwed and brought out to the fumehood. The reaction mixture was stirred at 40 °C for 48 hours. After 48 hours, DanH (1.5 eq) was added inside glovebox and stirred for another hour at 40 °C. NMR of the crude reaction mixture was recorded using trifluorotoluene as an internal standard. The product was isolated by flash chromatography with pentane/DCM system.

Step 3 (Scheme S1) was carried out using our previously reported method.[8] In the glovebox, the allyl-Bdan (1 eq) was taken in a screw capped reaction tube, DME (0.13 M) was added followed by 3N H_2SO_4 (9 eq). The reaction tube was tightly screwed and brought out to the fumehood. The mixture was stirred for 24 h at room temperature. The reaction mixture was centrifuged and the solution was transferred to another vial in the glovebox. Then, 0.5 M HCl (2 eq) as well as 5 mL DCM were added

to the solution and shaken vigorously. The DCM layer was passed through a phase separator to another vial. The NMR yield of the product was determined using trifluorotoluene as an internal standard. Next, the solvent of the crude product was removed by Ar below in the fumehood. In the end, the 0.1 M stock solution of allyl boronic acid was prepared by dissolving the crude product in appropriate amount of dry DCM with 3Å molecular sieves as desiccant in the glovebox. The stock solution of **1a-1f** and **1h** can be stored in freezer at -20 °C for four weeks, but the stock solution of **1g** cannot be kept for more than one week.

The racemates of allylboronic acid products were prepared by following the above procedure except that the equimolar mixture of (*R*) and (*S*)-iodo-BINOL was used as a catalyst for step 2. Minor deviations of a 50/50 ratio of the *S*/*R* enantiomers of allyl Bdan product may be due to weighing errors.

Diethyl (*E*)-oct-1-en-1-ylboronate

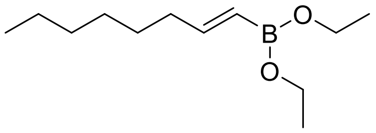

This compound was obtained as a colorless oil (0.65 g, 99% yield). **^{1}H NMR** (400 MHz, $CDCl_3$): δ = 6.57 (dt, *J* = 17.6, 6.5 Hz, 1H), 5.52 (dt, *J* = 17.6, 1.6 Hz, 1H), 3.93 (q, *J* = 7.0 Hz, 4H), 2.20 – 2.07 (m, 2H), 1.54 – 1.35 (m, 2H), 1.35 – 1.24 (m, 6H), 1.21 (t, *J* = 7.0 Hz, 6H), 0.92 – 0.83 (m, 3H) ppm.

(*S*,*E*)-2-(1,1,1-Trifluorodec-3-en-2-yl)-2,3-dihydro-1*H*-naphtho[1,8*de*][1,3,2]diazaborinine

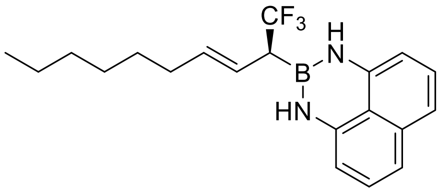

This compound was obtained as a colorless oil (1 mmol scale, 277 mg, 77% yield). **^{1}H NMR** (400 MHz, $CDCl_3$): δ = 7.16 – 7.03 (m, 4H), 6.34 (dd, *J* = 7.2, 1.2 Hz, 2H), 5.80 – 5.65 (m, 3H), 5.46 (ddt, *J* = 15.3, 9.3, 1.4 Hz, 1H), 2.68 (qd, *J* = 11.7, 9.1 Hz, 1H), 2.18 – 2.06 (m, 2H), 1.42 – 1.25 (m, 8H), 0.97 – 0.81 (m, 3H) ppm.

Spectroscopic data is in agreement with previous report.[8]

Determination of *ee* by Chiral SFC: Diacel CHIRALPAK IB N-3, 25 °C, 0.3 cm ϕ, 15 cm column, 10% MeOH in CO_2, flow rate: 0.8 mL/min; *t*R: 6.30 min (minor enantiomer), 7.47 min (major enantiomer); *ee* (major enantiomer) = 96%.

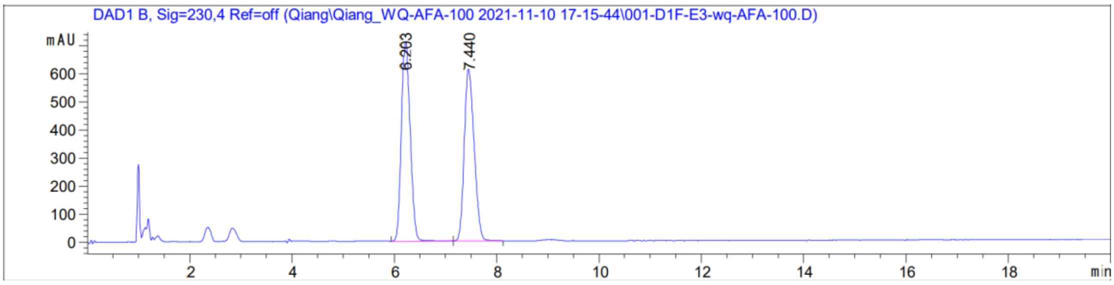

Signal 2: DAD1 B, Sig=230,4 Ref=off

Peak #	RetTime [min]	Type	Width [min]	Area [mAU*s]	Height [mAU]	Area %
1	6.203	BV R	0.1906	8535.81641	708.33044	50.8544
2	7.440	BV R	0.2113	8248.98828	611.99573	49.1456

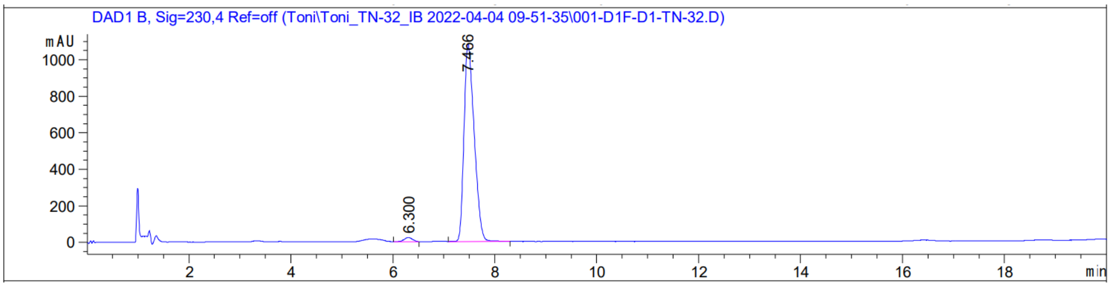

Signal 2: DAD1 B, Sig=230,4 Ref=off

Peak #	RetTime [min]	Type	Width [min]	Area [mAU*s]	Height [mAU]	Area %
1	6.300	VB	0.1765	263.63452	22.87230	1.7190
2	7.466	VV R	0.2181	1.50730e4	1082.93347	98.2810

(*S*,*E*)-(1,1,1-Trifluorodec-3-en-2-yl)boronic acid (1a)

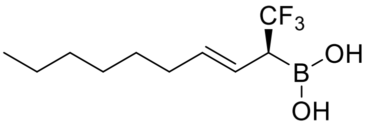

Compound **1a** was obtained as a 0.1 M stock solution (100% NMR yield). **^{1}H NMR** (400 MHz, $CDCl_3$): δ = 5.70 (dt, *J* = 14.2, 6.7 Hz, 1H), 5.44 (dd, *J* = 15.4, 9.0 Hz, 1H), 4.68 (br, 2H), 2.76 – 2.58 (m, 1H), 2.07 (q, *J* = 7.2 Hz, 2H), 1.44 – 1.13 (m, 8H), 0.88 (t, *J* = 6.6 Hz, 3H); **^{19}F NMR** (377 MHz, $CDCl_3$) δ = -63.04 (d, *J* = 11.7 Hz, 3F) ppm.

Spectroscopic data is in agreement with previous report.[8]

Diethyl (*E*)-oct-1-en-1-ylboronate

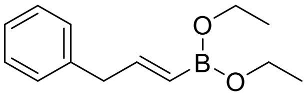

This compound was obtained as a colorless oil (0.84 g, 99% yield). **^{1}H NMR** (400 MHz, $CDCl_3$): δ = 7.36 – 7.26 (m, 2H), 7.26 – 7.13 (m, 3H), 6.79 – 6.64 (m, 1H), 5.67 – 5.55 (m, 1H), 3.99 – 3.89 (m, 4H), 3.51 (dt, *J* = 6.6, 1.8 Hz, 2H), 1.22 (tdd, *J* = 7.0, 2.3, 1.3 Hz, 6H) ppm.

(*S*,*E*)-2-(1,1,1-Trifluoro-5-phenylpent-3-en-2-yl)-2,3-dihydro-1*H*-naphtho[1,8 *de*][1,3,2]diazaborinine

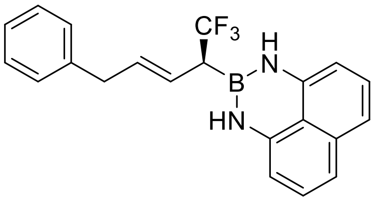

This compound was obtained as a colorless oil (0.5 mmol scale, 165 mg, 90% yield). **^{1}H NMR** (400 MHz, $CDCl_3$): δ = 7.35 (dd, *J* = 8.2, 6.8 Hz, 2H), 7.31 – 7.20 (m, 3H), 7.17 – 7.02 (m, 4H), 6.29 (dd, *J* = 7.2, 1.2 Hz, 2H), 5.89 (dt, *J* = 14.5, 6.9 Hz, 1H), 5.73 (br, 2H), 5.59 (ddt, *J* = 15.4, 9.4, 1.4 Hz, 1H), 3.47 (d, *J* = 6.9 Hz, 2H), 2.82 – 2.66 (m, 1H) ppm.

Spectroscopic data is in agreement with previous report.[8]

Determination of *ee* by Chiral SFC: Diacel CHIRALPAK IB N-3, 25 °C, 0.3 cm ϕ, 15 cm column, 10% MeOH in CO_2, flow rate: 0.8 mL/min; *t*R: 16.8 min (minor enantiomer), 18.7 min (major enantiomer); *ee* (major enantiomer) = 96%.

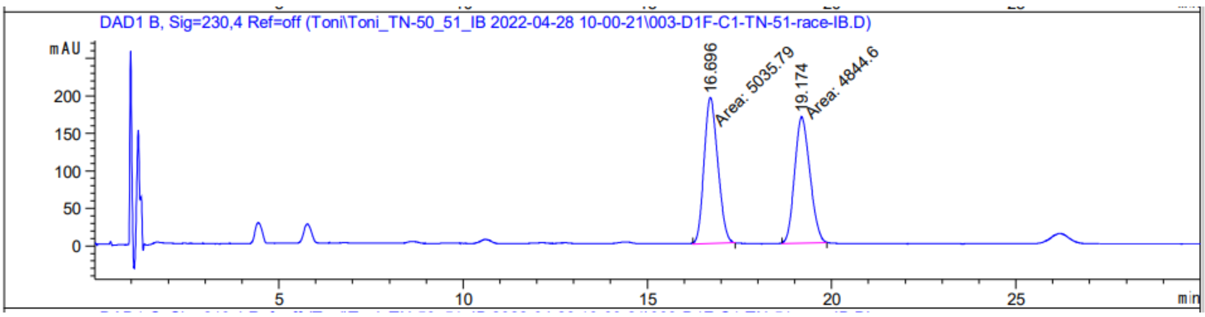

Signal 2: DAD1 B, Sig=230,4 Ref=off

Peak #	RetTime [min]	Type	Width [min]	Area [mAU*s]	Height [mAU]	Area %
1	16.696	MM	0.4310	5035.79053	194.72295	50.9675
2	19.174	MM	0.4806	4844.59668	168.01599	49.0325

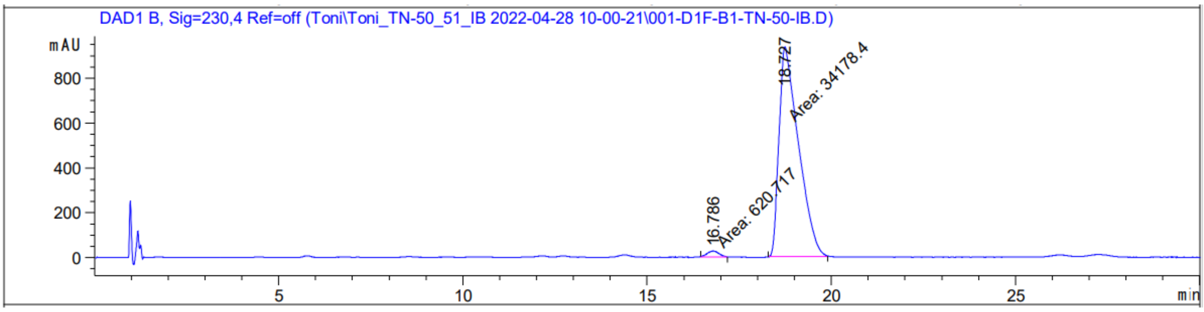

Signal 2: DAD1 B, Sig=230,4 Ref=off

Peak #	RetTime [min]	Type	Width [min]	Area [mAU*s]	Height [mAU]	Area %
1	16.786	MM	0.3849	620.71722	26.88131	1.7837
2	18.727	MM	0.6099	3.41784e4	933.95831	98.2163

(*S*,*E*)-(1,1,1-Trifluoro-5-phenylpent-3-en-2-yl)boronic acid (1b)

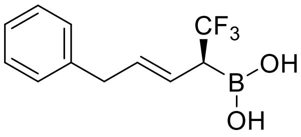

Compound **1b** was obtained as a 0.1 M stock solution (100% NMR yield). **^{1}H NMR** (400 MHz, $CDCl_3$): δ = 7.30 (t, J = 7.3 Hz, 2H), 7.21 (t, J = 7.2 Hz, 0H), 7.17 (d, J = 7.6 Hz, 2H), 5.86 (dt, J = 14.4, 6.8 Hz, 1H), 5.56 (dd, J = 15.4, 9.0 Hz, 1H), 4.74 (br, 2H), 3.42 (d, J = 6.9 Hz, 2H), 2.84 – 2.59 (m, 1H); **^{19}F NMR** (377 MHz, $CDCl_3$) δ = -62.93 (d, J = 11.6 Hz, 3F) ppm.

Diethyl (*E*)-(6-chlorohex-1-en-1-yl)boronate

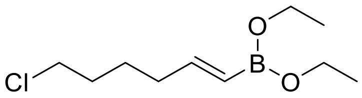

This compound was obtained as a colorless oil (0.86 g, 90% yield). **^{1}H NMR** (400 MHz, $CDCl_3$): δ = 6.54 (dt, J = 17.6, 6.5 Hz, 1H), 5.55 (dt, J = 17.6, 1.5 Hz, 1H), 3.93 (q, J = 7.0 Hz, 4H), 3.54 (t, J = 6.7 Hz, 2H), 2.18 (tdd, J = 7.3, 5.2, 1.6 Hz, 2H), 1.80 (dt, J = 14.3, 6.7 Hz, 2H), 1.67 – 1.51 (m, 2H), 1.21 (t, J = 7.0 Hz, 6H) ppm.

(*S*,*E*)-2-(8-Chloro-1,1,1-trifluorooct-3-en-2-yl)-2,3-dihydro-1*H*-naphtho[1,8-*de*][1,3,2]diazaborinine

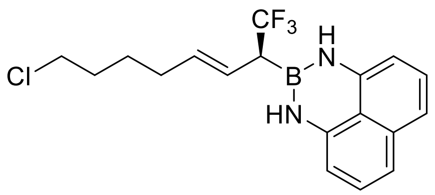

This compound was obtained as a colorless oil (0.5 mmol scale, 141 mg, 77% yield). **^{1}H NMR** (400 MHz, $CDCl_3$): δ = 7.17 – 6.97 (m, 4H), 6.35 (dd, J = 7.1, 1.1 Hz, 2H), 5.81 – 5.64 (m, 3H), 5.50 (ddt, J = 15.3, 9.2, 1.4 Hz, 1H), 3.56 (t, J = 6.6 Hz, 2H), 2.77 – 2.61 (m, 1H), 2.24 – 2.09 (m, 2H), 1.90 – 1.74 (m, 2H), 1.67 – 1.55 (m, 2H); **^{13}C NMR** (100 MHz, $CDCl_3$) δ = 140.2, 136.7, 136.3, 127.8 (q, J = 276.0 Hz), 127.7, 121.50 (q, J = 4.0 Hz), 119.9, 118.5, 106.5, 45.0, 32.1, 32.1, 26.5; **^{19}F NMR** (377 MHz, $CDCl_3$) = -62.39 (d, J = 11.7 Hz, 3F); **^{11}B NMR** (128 MHz, $CDCl_3$) δ = 29.01 ppm. **HRMS (pos. APCI-ESI)** m/z: calcd for $C_{18}H_{20}BClF_3N_2$ $[M+H]^+$ 367.1355, found 367.1352. **ORD** ($CHCl_3$, c 0.1, 25.6 °C): -53.

Determination of *ee* by Chiral SFC: Diacel CHIRALPAK IB N-3, 25 °C, 0.3 cm ϕ, 15 cm column, 15% MeOH in CO_2, flow rate: 0.8 mL/min; *t*R: 7.73 min (minor enantiomer), 9.75 min (major enantiomer); *ee* (major enantiomer) = 97%.

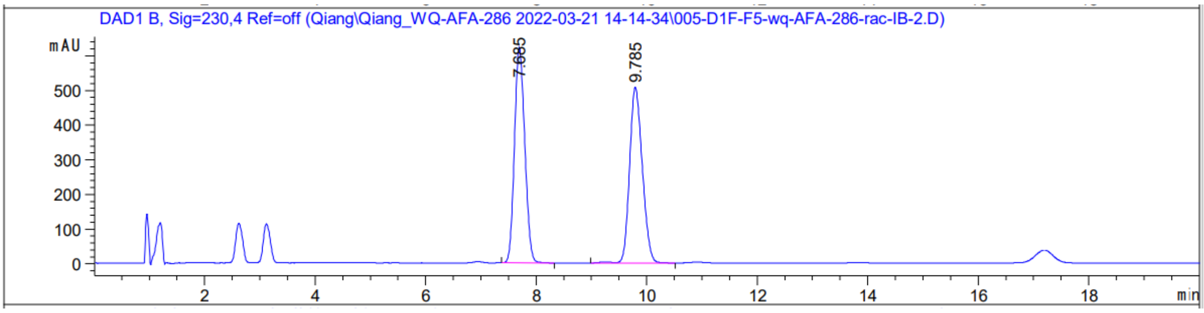

Signal 2: DAD1 B, Sig=230,4 Ref=off

Peak #	RetTime [min]	Type	Width [min]	Area [mAU*s]	Height [mAU]	Area %
1	7.685	BB	0.2031	8049.94873	621.73999	49.7930
2	9.785	VB R	0.2476	8116.86768	507.49512	50.2070

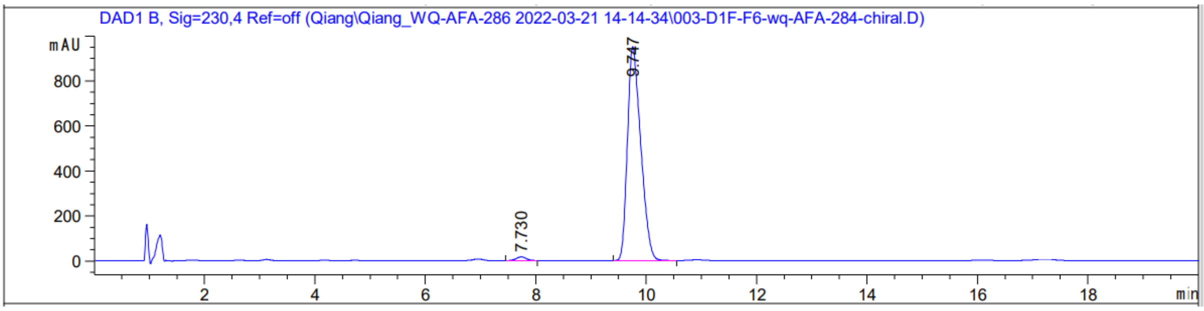

Signal 2: DAD1 B, Sig=230,4 Ref=off

Peak #	RetTime [min]	Type	Width [min]	Area [mAU*s]	Height [mAU]	Area %
1	7.730	BB	0.1881	204.74133	16.34693	1.2721
2	9.747	BV	0.2571	1.58899e4	950.33899	98.7279

(*S*,*E*)-(1,1,1-Trifluoro-5-phenylpent-3-en-2-yl)boronic acid (1c)

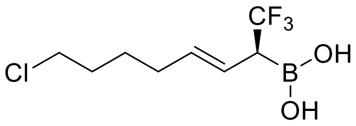

Compound **1c** was obtained as a 0.1 M stock solution (100% NMR yield). **^{1}H NMR** (400 MHz, $CDCl_3$): δ = 6.30 (br, 2H), 5.61 (dt, J = 14.2, 6.6 Hz, 1H), 5.48 – 5.42 (m, 1H), 3.72 – 3.66 (m, 2H), 2.57 (p, J = 11.2 Hz, 1H), 2.07 (q, J = 6.5 Hz, 2H), 1.80 – 1.68 (m, 2H), 1.55 – 1.47 (m, 2H); **^{19}F NMR** (377 MHz, $CDCl_3$) δ = -63.49 (d, J = 11.5 Hz, 3F) ppm.

(*S*,*E*)-6,6,6-Trifluoro-5-(1*H*-naphtho[1,8-*de*][1,3,2]diazaborinin-2(3*H*)-yl)hex-3-en-1-yl 4-methyl-benzenesulfonate

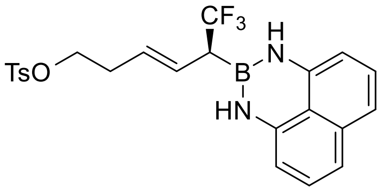

This compound was obtained as a colorless oil (0.5 mmol scale, 197 mg, 83% yield). **^{1}H NMR** (400 MHz, $CDCl_3$): δ = 7.78 (d, J = 8.0 Hz, 2H), 7.29 (d, J = 8.0 Hz, 2H), 7.18 – 6.97 (m, 4H), 6.40 (dd, J = 7.2, 1.2 Hz, 2H), 5.82 (br, 2H), 5.68 – 5.46 (m, 2H), 4.23 – 4.02 (m, 2H), 2.80 – 2.60 (m, 1H), 2.55 – 2.43 (m, 2H), 2.41 (s, 3H); **^{13}C NMR** (100 MHz, $CDCl_3$) δ = 145.1, 140.2, 136.3, 133.2, 131.1, 130.0, 128.0, 127.72, 127.67 (q, J = 275.9 Hz), 125.0 (q, J = 3.8 Hz), 120.0, 118.5, 106.7, 69.3, 32.5, 21.8; **^{19}F NMR** (377 MHz, $CDCl_3$) = -62.16 (d, J = 11.7 Hz, 3F); **^{11}B NMR** (128 MHz, $CDCl_3$) δ = 29.13 ppm. **HRMS (pos. ESI)** m/z: calcd for $C_{23}H_{22}BF_3N_2NaO_3S^+$ [M+Na]$^+$ 497.1288, found 497.1293. **ORD** ($CHCl_3$, c 0.1, 25.7 ºC): -38.

Determination of *ee* by Chiral SFC: Diacel CHIRALPAK IB N-3, 25 °C, 0.3 cm ϕ, 15 cm column, 20% MeOH in CO_2, flow rate: 0.8 mL/min; *t*R: 9.12 min (minor enantiomer), 9.97 min (major enantiomer); *ee* (major enantiomer) = 97%.

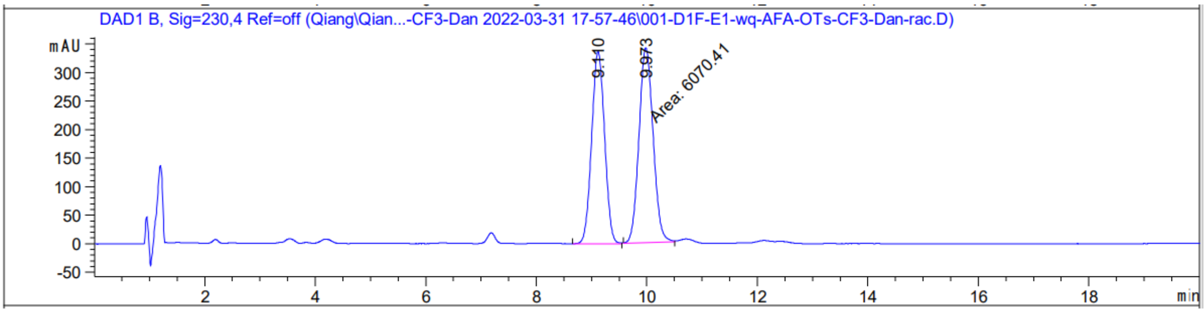

Signal 2: DAD1 B, Sig=230,4 Ref=off

Peak #	RetTime [min]	Type	Width [min]	Area [mAU*s]	Height [mAU]	Area %
1	9.110	BV	0.2556	5466.68066	336.33347	47.3835
2	9.973	MM T	0.2965	6070.41162	341.26239	52.6165

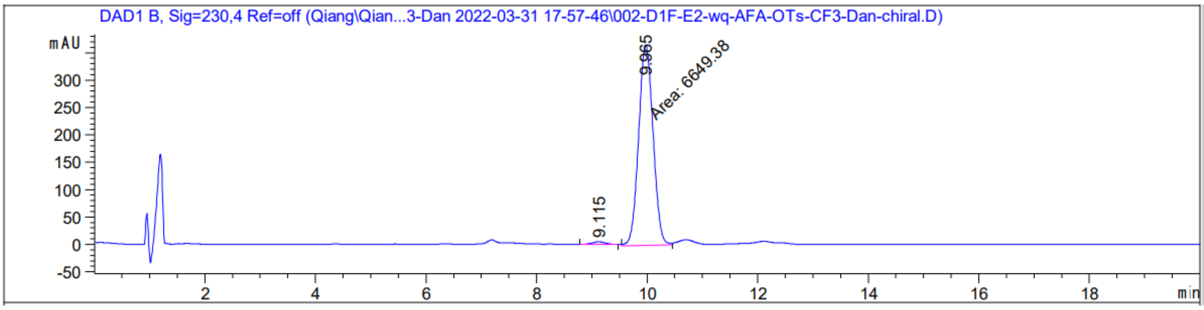

Signal 2: DAD1 B, Sig=230,4 Ref=off

Peak #	RetTime [min]	Type	Width [min]	Area [mAU*s]	Height [mAU]	Area %
1	9.115	BB	0.2392	76.73187	4.53947	1.1408
2	9.965	MM T	0.3026	6649.37695	366.19974	98.8592

(*S*,*E*)-(1,1,1-Trifluoro-6-(tosyloxy)hex-3-en-2-yl)boronic acid (1d)

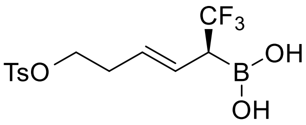

Compound **1d** was obtained as a 0.1 M stock solution (100% NMR yield). ^{1}**H NMR** (400 MHz, $CDCl_3$): δ = 7.45 (d, *J* = 6.4 Hz, 2H), 7.32 (d, *J* = 8.0 Hz, 2H), 6.26 (br, 2H), 4.02 (t, *J* = 6.6, 2H), 2.62 – 2.48 (m, 1H), 2.42 (s, 3H), 2.36 (q, *J* = 6.6 Hz, 2H), both vinyl C-H signals are covered by DCM; ^{19}**F NMR** (377 MHz, $CDCl_3$) δ = - 63.30 (d, *J* = 11.5 Hz, 3F) ppm.

Dethyl (*E*)-(3-(1,3-dioxoisoindolin-2-yl)prop-1-en-1-yl)boronate

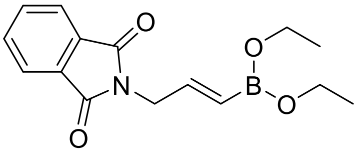

This compound was obtained as a white solid (0.84 g, containing 30% mono-ester and 70% di-ester, 70% yield, use directly). ^{1}**H NMR** (400 MHz, $CDCl_3$): δ = 7.91 – 7.83 (m, 2H), 7.76 – 7.70 (m, 2H), 6.58 – 6.41 (m, 1H), 5.76 – 5.67 (m, 1H), 4.42 – 4.31 (m, 2H), 3.98 – 3.82 (m, 2H), 1.21 – 1.07 (m, 6H) ppm.

(*S*,*E*)-2-(5,5,5-Trifluoro-4-(1*H*-naphtho[1,8-*de*][1,3,2]diazaborinin-2(3*H*)-yl)pent-2-en-1-yl)isoindoline-1,3-dione

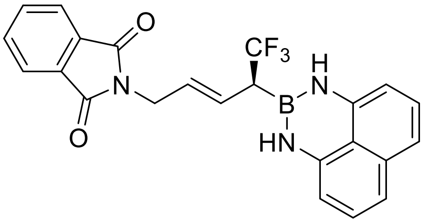

This compound was obtained as an orange solid (0.5 mmol scale, 196 mg, 90% yield). ^{1}**H NMR** (400 MHz, $CDCl_3$): δ = 7.90 (dd, *J* = 5.5, 3.1 Hz, 2H), 7.75 (dd, *J* = 5.5, 3.1 Hz, 2H), 7.12 (t, *J* = 7.8 Hz, 2H), 7.04 (d, *J* = 8.2 Hz, 2H), 6.08 (br, 2H), 5.86 (dd, *J* = 15.5, 9.6 Hz, 1H), 5.69 (ddd, *J* = 15.4, 9.9, 5.5 Hz, 1H), 4.52 (dd, *J* = 15.0, 4.7 Hz, 1H), 4.29 (dd, *J* = 15.1, 7.5 Hz, 1H), 2.80 (p, *J* = 11.3 Hz, 1H); ^{13}**C NMR** (100 MHz, $CDCl_3$) δ = 168.4, 140.5, 136.3, 134.4, 132.2, 129.0, 127.7, 127.6 (q, *J* = 275.9 Hz), 126.3 (q, *J* = 3.8 Hz), 123.6, 120.1, 118.4, 106.6, 40.1; ^{19}**F NMR** (377 MHz, $CDCl_3$) = -61.84 (d, *J* = 11.6 Hz, 3F); ^{11}**B NMR** (128 MHz, $CDCl_3$) δ = 28.66 ppm. **HRMS (pos. ESI)** m/z: calcd for $C_{23}H_{17}BF_3N_3NaO_2^+$ $[M+Na]^+$ 458.1258, found 458.1256. **ORD** ($CHCl_3$, c 0.1, 25.0 °C): -98.

Determination of *ee* by Chiral SFC: Diacel CHIRALPAK IB N-3, 25 °C, 0.3 cm ϕ, 15 cm column, 10% MeOH in CO_2, flow rate: 0.8 mL/min; *t*R: 28.27 min (minor enantiomer), 25.44 min (major enantiomer); *ee* (major enantiomer) = 98%.

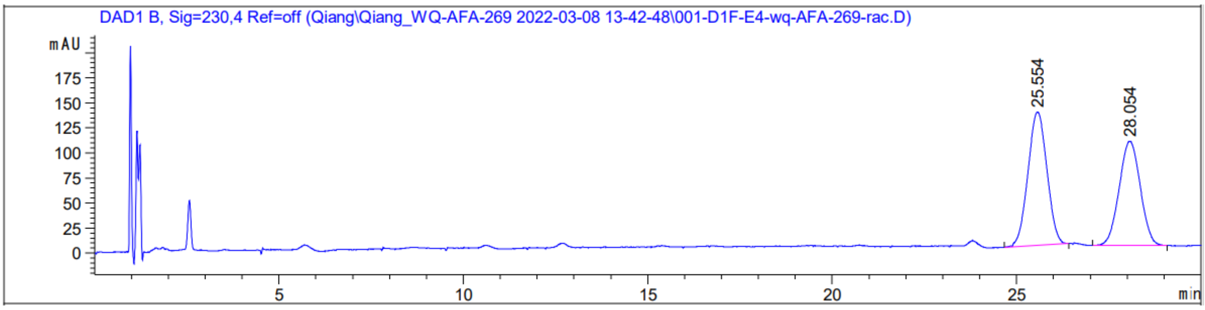

Signal 2: DAD1 B, Sig=230,4 Ref=off

Peak #	RetTime [min]	Type	Width [min]	Area [mAU*s]	Height [mAU]	Area %
1	25.554	VB R	0.5425	4755.17334	133.02852	53.3894
2	28.054	BB	0.5657	4151.42090	104.44360	46.6106

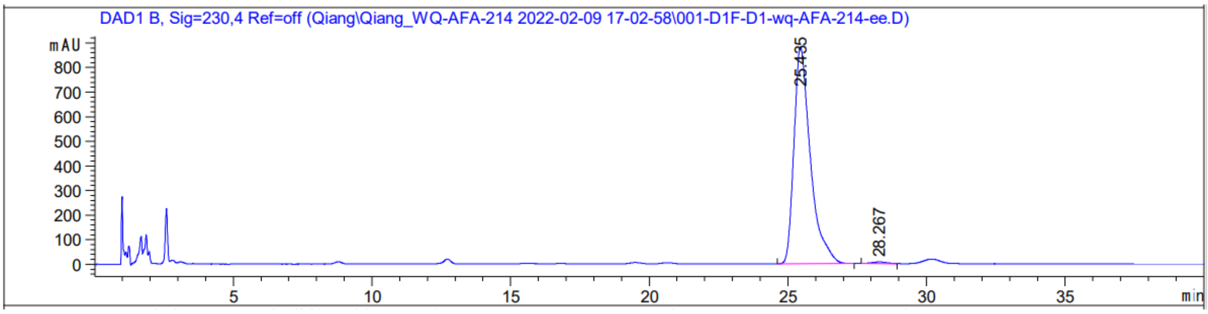

Signal 2: DAD1 B, Sig=230,4 Ref=off

Peak #	RetTime [min]	Type	Width [min]	Area [mAU*s]	Height [mAU]	Area %
1	25.435	BV R	0.6072	3.63350e4	880.86249	99.3026
2	28.267	BV	0.4546	255.17978	6.60656	0.6974

(*S*,*E*)-(5-(1,3-Dioxoisoindolin-2-yl)-1,1,1-trifluoropent-3-en-2-yl)boronic acid (1e)

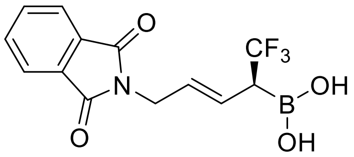

Compound **1e** was obtained as a 0.1 M stock solution (100% NMR yield). **^{1}H NMR** (400 MHz, $CDCl_3$): δ = 7.89 – 7.79 (m, 2H), 7.78 – 7.67 (m, 2H), 5.91 – 5.76 (m, 1H), 5.76 – 5.60 (m, 1H), 5.22 (br, 2H), 5.49 – 4.37 (m, 1H), 4.22 (dd, *J* = 15.0, 7.2 Hz, 1H), 2.77 (p, *J* = 12.9 Hz, 1H); **^{19}F NMR** (377 MHz, $CDCl_3$) δ = - 62.52 (d, *J* = 11.9 Hz, 3F) ppm.

Diethyl (*E*)-(2-cyclohexylvinyl)boronate

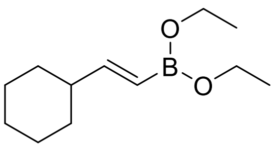

This compound was obtained as a colorless oil (0.8 g, 98% yield). **^{1}H NMR** (400 MHz, $CDCl_3$): δ = 6.51 (dd, *J* = 17.8, 6.4 Hz, 1H), 5.48 (dd, *J* = 17.7, 1.4 Hz, 1H), 3.93 (q, *J* = 7.1 Hz, 4H), 2.07 – 1.96 (m, 1H), 1.81 – 1.69 (m, 4H), 1.34 – 1.04 (m, 12H) ppm.

(*S*,*E*)-2-(4-Cyclohexyl-1,1,1-trifluorobut-3-en-2-yl)-2,3-dihydro-1*H*-naphtho[1,8-*de*][1,3,2]diazaborinine

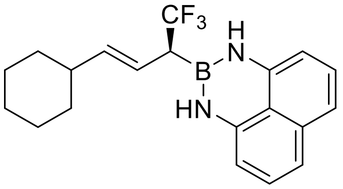

This compound was obtained as a colorless oil (0.5 mmol scale, 158 mg, 88% yield). **^{1}H NMR** (400 MHz, $CDCl_3$): δ = 7.18 – 7.01 (m, 4H), 6.34 (dd, J = 7.2, 1.1 Hz, 2H), 5.75 (br, 2H), 5.66 (dd, J = 15.5, 6.8 Hz, 1H), 5.42 (ddd, J = 15.5, 9.3, 1.3 Hz, 1H), 2.75 – 2.54 (m, 1H), 2.10 – 1.98 (m, 1H), 1.79 – 1.66 (m, 4H), 1.38 – 1.00 (m, 6H); **^{13}C NMR** (100 MHz, $CDCl_3$) δ = 143.3, 140.3, 136.3, 128.0 (d, J = 277.3 Hz), 127.7, 119.9, 118.3 (q, J = 4.0 Hz), 106.4, 41.0, 33.1, 32.9, 26.2, 26.1, 26.0; **^{19}F NMR** (377 MHz, $CDCl_3$) = -62.37 (d, J = 11.6 Hz, 3F); **^{11}B NMR** (128 MHz, $CDCl_3$) δ = 29.35 ppm. **HRMS (pos. APCI-ESI)** m/z: calcd for $C_{20}H_{23}BF_3N_2^+$ $[M+H]^+$ 359.1901, found 359.1924. **ORD** ($CHCl_3$, c 0.1, 25.3 °C): -49.

Determination of *ee* by Chiral SFC: Diacel CHIRALPAK IB N-3, 25 °C, 0.3 cm ϕ, 15 cm column, 10% MeOH in CO_2, flow rate: 0.8 mL/min; *t*R: 7.69 min (minor enantiomer), 8.44 min (major enantiomer); *ee* (major enantiomer) = 98%.

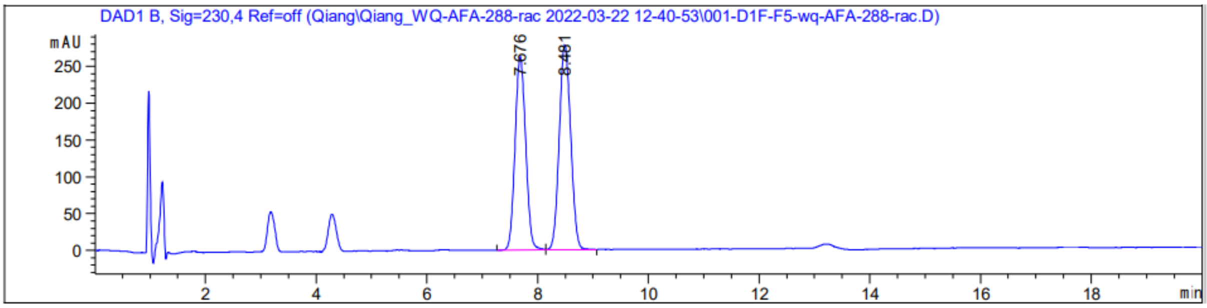

Signal 2: DAD1 B, Sig=230,4 Ref=off

Peak #	RetTime [min]	Type	Width [min]	Area [mAU*s]	Height [mAU]	Area %
1	7.676	VV R	0.2098	3510.18823	262.80386	46.7276
2	8.481	VB	0.2258	4001.83667	278.20126	53.2724

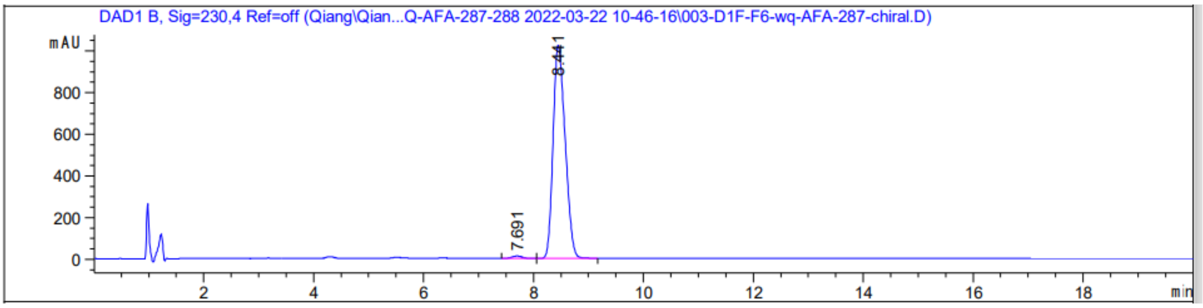

Signal 2: DAD1 B, Sig=230,4 Ref=off

Peak #	RetTime [min]	Type	Width [min]	Area [mAU*s]	Height [mAU]	Area %
1	7.691	BB	0.2024	156.18803	11.80074	0.9918
2	8.441	BV R	0.2399	1.55920e4	1022.80603	99.0082

(*S*,*E*)-(4-Cyclohexyl-1,1,1-trifluorobut-3-en-2-yl)boronic acid (1f)

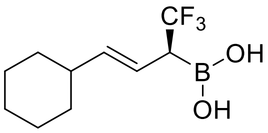

Compound **1f** was obtained as a 0.1 M stock solution (100% NMR yield). **^{1}H NMR** (400 MHz, $CDCl_3$): δ = 6.24 (br, 2H), 5.55 (dd, *J* = 15.5, 6.6 Hz, 1H), 5.43 – 5.32 (m, 1H), 2.52 (p, *J* = 11.2 Hz, 1H), 1.72 – 1.56 (m, 4H), 1.29 – 0.90 (m, 6H); **^{19}F NMR** (377 MHz, $CDCl_3$) δ = - 63.51 (d, *J* = 11.8 Hz, 3F) ppm.

Diethyl (*E*)-styrylboronate

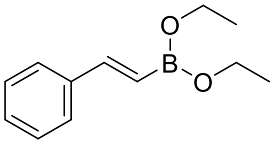

This compound was obtained as a colorless oil (0.82 g, 100% yield). **^{1}H NMR** (400 MHz, $CDCl_3$): δ = 7.63 – 7.44 (m, 2H), 7.41 – 7.26 (m, 4H), 6.29 (d, *J* = 18.1 Hz, 1H), 4.03 (q, *J* = 7.1 Hz, 4H), 1.27 (t, *J* = 7.1 Hz, 6H) ppm.

(*S*,*E*)-2-(1,1,1-Trifluoro-4-phenylbut-3-en-2-yl)-2,3-dihydro-1*H*-naphtho[1,8-*de*][1,3,2]diazaborinine

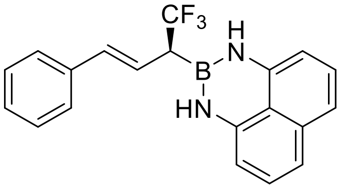

This compound was obtained as a colorless oil (0.5 mmol scale, 176 mg, 90% yield). **^{1}H NMR** (400 MHz, $CDCl_3$): δ = 7.45 – 7.40 (m, 2H), 7.38 – 7.33 (m, 2H), 7.31 – 7.26 (m, 1H), 7.16 – 6.97 (m, 4H), 6.63 (d, *J* = 15.9 Hz, 1H), 6.35 (dd, *J* = 7.2, 1.2 Hz, 2H), 6.22 (dd, *J* = 15.9, 9.4 Hz, 1H), 5.82 (br, 2H), 2.92 (p, *J* = 11.2 Hz, 1H) ppm.

Spectroscopic data is in agreement with previous report.[8]

Determination of *ee* by Chiral SFC: Diacel CHIRALPAK OJ-H, 25 °C, 0.3 cm ϕ, 15 cm column, 20% MeOH in CO_2, flow rate: 1.2 mL/min; *t*R: 10.90 min (minor enantiomer), 9.51 min (major enantiomer); *ee* (major enantiomer) = 98%.

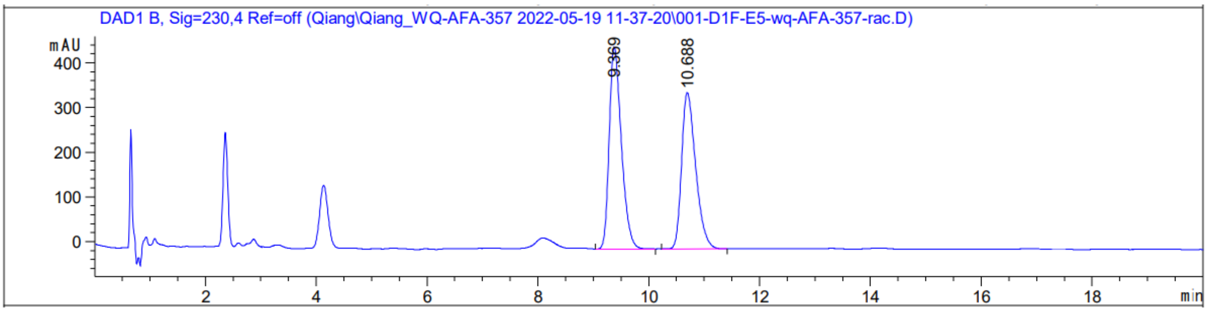

Signal 2: DAD1 B, Sig=230,4 Ref=off

Peak #	RetTime [min]	Type	Width [min]	Area [mAU*s]	Height [mAU]	Area %
1	9.369	BB	0.2318	6881.20947	451.55106	52.7683
2	10.688	BB	0.2695	6159.20947	349.61584	47.2317

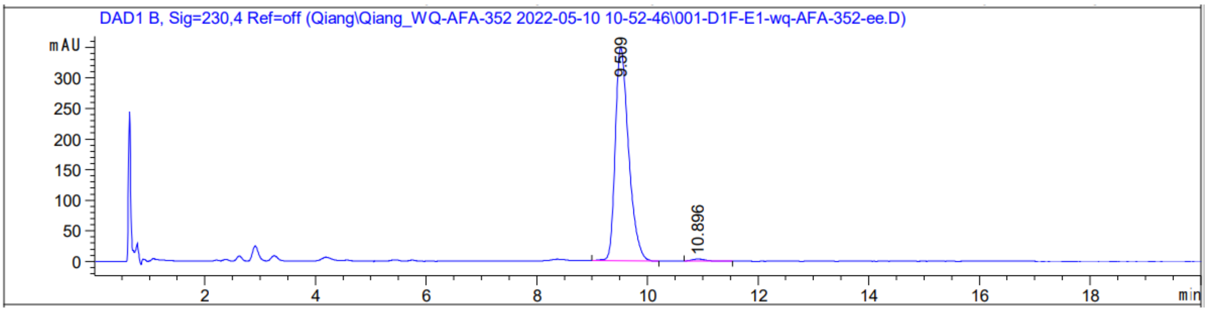

Signal 2: DAD1 B, Sig=230,4 Ref=off

Peak #	RetTime [min]	Type	Width [min]	Area [mAU*s]	Height [mAU]	Area %
1	9.509	BB	0.2551	5849.54980	349.76126	98.9824
2	10.896	BB	0.2212	60.13546	3.38598	1.0176

(*S*,*E*)-(1,1,1-Trifluoro-4-phenylbut-3-en-2-yl)boronic acid (1g)

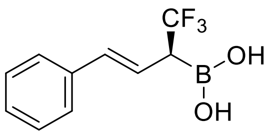

Compound **1g** was obtained as a 0.1 M stock solution (100% NMR yield). **^{1}H NMR** (400 MHz, $CDCl_3$): δ = 7.29 (d, *J* = 9.3 Hz, 2H), 7.21 (t, *J* = 8.2 Hz, 3H), 7.13 (t, *J* = 7.6 Hz, 1H), 6.57 (br, 2H), 6.47 (d, *J* = 16.0 Hz, 1H), 6.16 (dd, *J* = 15.8, 9.4 Hz, 1H), 2.71 (p, *J* = 10.9 Hz, 1H).; **^{19}F NMR** (377 MHz, $CDCl_3$) δ = -63.15 (d, *J* = 11.1 Hz, 3F) ppm.

Spectroscopic data is in agreement with previous report.[8]

Diethyl (*E*)-(3,3-dimethylbut-1-en-1-yl)boronate

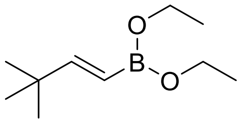

This compound was obtained as a yellow oil (0.56 g, 100% yield). **^{1}H NMR** (400 MHz, $CDCl_3$): δ = 6.58 (d, *J* = 18.0 Hz, 1H), 5.43 (d, *J* = 18.0 Hz, 1H), 3.94 (q, *J* = 7.0 Hz, 4H), 1.21 (t, *J* = 7.0 Hz, 6H), 1.03 (s, 9H) ppm.

(*S*,*E*)-2-(1,1,1-trifluoro-5,5-dimethylhex-3-en-2-yl)-2,3-dihydro-1*H*-naphtho[1,8-de][1,3,2]diazaborinine

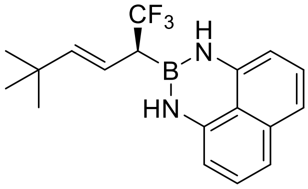

This compound was obtained as a white solid (0.5 mmol scale, 150 mg, 90% yield). **^{1}H NMR** (400 MHz, $CDCl_3$): δ = 7.19 – 6.95 (m, 4H), 6.34 (dd, *J* = 7.2, 1.1 Hz, 2H), 5.86 – 5.59 (m, 3H), 5.37 (dd, *J* = 15.7, 9.3 Hz, 1H), 2.66 (qd, *J* = 11.8, 9.1 Hz, 1H), 1.08 (s, 9H); **^{13}C NMR** (100 MHz, $CDCl_3$): δ = 148.3, 140.3, 136.3, 128.0 (q, *J* = 277.3 Hz), 127.7, 118.5, 115.8 (q, *J* = 3.8 Hz), 106.4, 33.7, 29.7; **^{19}F NMR** (377 MHz, $CDCl_3$) δ = -62.38 (d, *J* = 11.8 Hz, 3F); **^{11}B NMR** (128 MHz, $CDCl_3$) δ = 29.19 ppm. **HRMS (pos. APCI-ESI)** m/z: calcd for $C_{18}H_{21}BF_3N_2^+$ $[M+H]^+$ 333,1744, found 333,1750. **ORD** ($CHCl_3$, c 0.1, 25.3 ºC): -60.

Determination of *ee* by Chiral SFC: Diacel CHIRALPAK IB, 25 °C, 0.3 cm ϕ, 15 cm column, 10% MeOH in CO_2, flow rate: 0.8 mL/min; *t*R: 3.44 min (minor enantiomer), 3.73 min (major enantiomer); *ee* (major enantiomer) = 95%.

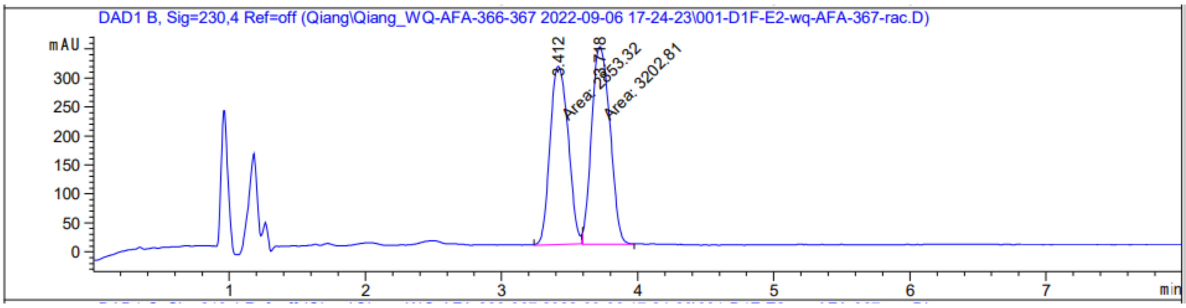

Signal 2: DAD1 B, Sig=230,4 Ref=off

Peak #	RetTime [min]	Type	Width [min]	Area [mAU*s]	Height [mAU]	Area %
1	3.412	MM T	0.1543	2853.31958	308.10687	47.1145
2	3.718	MM T	0.1564	3202.81348	341.36011	52.8855

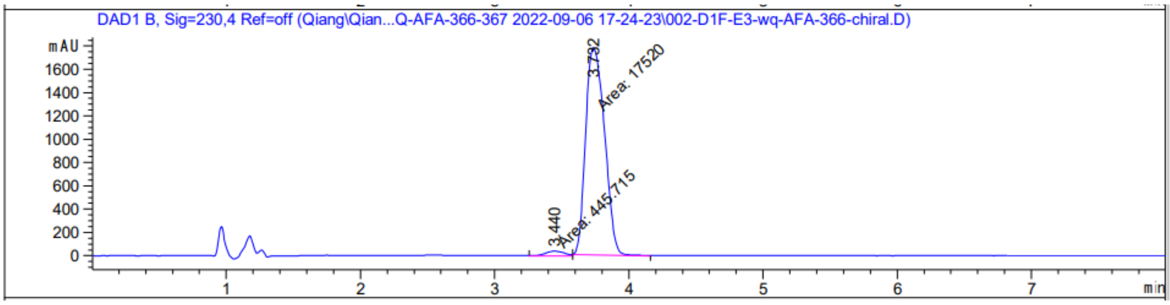

Signal 2: DAD1 B, Sig=230,4 Ref=off

Peak #	RetTime [min]	Type	Width [min]	Area [mAU*s]	Height [mAU]	Area %
1	3.440	MM T	0.1759	445.71497	42.24302	2.4809
2	3.732	MM T	0.1639	1.75200e4	1781.22864	97.5191

(*S*,*E*)-(1,1,1-trifluoro-5,5-dimethylhex-3-en-2-yl)boronic acid (1h)

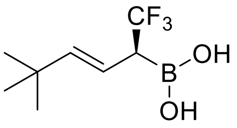

Compound **1h** was obtained as a 0.1 M stock solution (100% NMR yield). ^{1}**H NMR** (400 MHz, $CDCl_3$): δ = 5.66 (d, J = 15.7 Hz, 1H), 2.55 (p, J = 11.3 Hz, 1H), 1.00 (s, 9H); ^{19}**F NMR** (377 MHz, $CDCl_3$) δ = -63.51 (d, J = 11.3 Hz, 3F) ppm.

General procedure A for the trifluoromethylthiolation

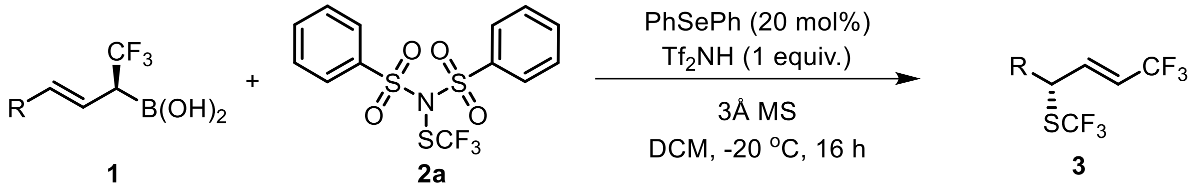

Scheme S2. Synthesis of allyl trifluoromethyl thioester 3a-3h.

An oven-dried vial was charged with $(PhSO_2)_2NSCF_3$ **2a** (0.45 mmol) followed addition of triflimide (0.3 mmol) and 3Å molecular sieves (60 mg) in a glove box. The vial was sealed and transferred to a -20 °C cooling bath. To this reaction mixture was added selenide catalyst (0.06 mmol) and the stock solution of allylboronic acid **1** (0.3 mmol, 3 mL) by syringe. Then, the reaction mixture was stirred at -20 °C for 16 h. After quenching by adding water, the NMR of the crude reaction mixture was recorded using trifluorotoluene as an internal standard. The product was isolated by flash chromatography. (Note: products **3a-3c** and **3f-3h** are volatile, the solvent after chromatography should be evaporated carefully at 0 °C in these cases.)

(*R*,*E*)-(1,1,1-Trifluorodec-2-en-4-yl)(trifluoromethyl)sulfane (3a)

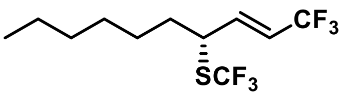

This compound was obtained according to the general procedure A. Product **3a** was isolated in 63% yield (55.6 mg) as a colorless oil by silica gel chromatography using pentane as eluent. **^{1}H NMR** (400 MHz, $CDCl_3$): δ = 6.33 (ddq, J = 15.6, 9.1, 2.0 Hz, 1H), 5.78 (dq, J = 15.5, 6.2 Hz, 1H), 3.95 – 3.71 (m, 1H), 1.77 – 1.62 (m, 2H), 1.47 – 1.19 (m, 8H), 0.93 – 0.84 (m, 3H); **^{13}C NMR** (100 MHz, $CDCl_3$) δ = 139.6 (q, J = 6.3 Hz), 130.3 (q, J = 307.3 Hz), 122.6 (q, J = 269.6 Hz), 120.3 (q, J = 34.1 Hz), 45.9 (q, J = 1.8 Hz), 33.5, 31.6, 28.8, 26.8, 22.6, 14.1; **^{19}F NMR** (377 MHz, $CDCl_3$) δ = -39.13 (s, 3F), -63.48 – -65.04 (m, 3F) ppm. **EI-MS (m/z, relative intensity)** 275 ($[M-F]^+$, 7), 225 ($[M-CF_3]^+$, 44), 151 (37), 131 (24), 122 (13), 69 (36), 57 (40), 43 (100). **ORD** ($CHCl_3$, c 0.1, 25.0 °C): -16.

Determination of *ee* by Chiral GC: Column Chiraldex β-DM, 50 °C, 60 min, *t*R: 23.43 min (minor enantiomer), 23.73 min (major enantiomer); *ee* (major enantiomer) = 98%.

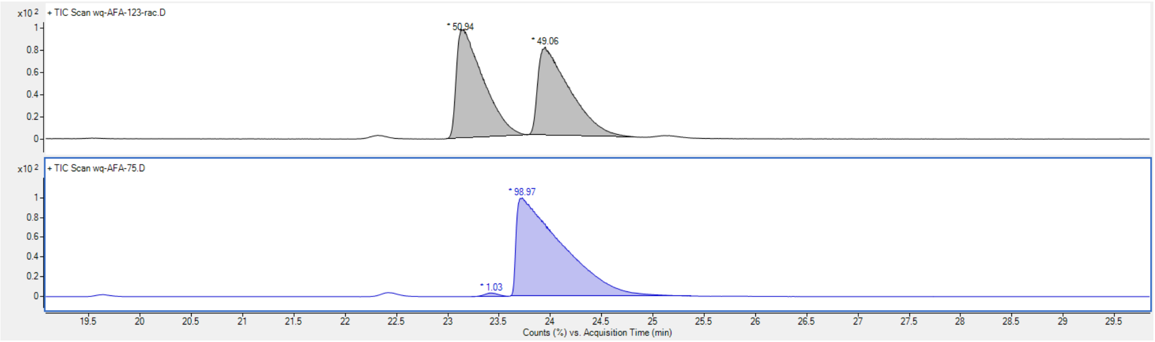

Area	Height	Type	Peak	RT	Area %	Area Sum %	Saturated	Width	FWHM	SNR	RI
782336.55	84389.55	M	1	23.427	1.04	1.03		0.355	3.879		
74899325.51	2408931.6	M	2	23.727	100	98.97		1.763	0.482		

(*R*,*E*)-(5,5,5-Trifluoro-1-phenylpent-3-en-2-yl)(trifluoromethyl)sulfane (3b)

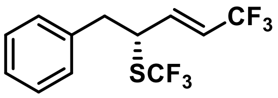

This compound was obtained according to the general procedure A. Product **3b** was isolated in 50% yield (45 mg) as a colorless oil by silica gel chromatography using pentane as eluent. **^{1}H NMR** (400 MHz, $CDCl_3$): δ = 7.36 – 7.29 (m, 3H), 7.15 (dd, J = 6.8, 1.9 Hz, 2H), 6.36 (ddq, J = 15.6, 9.2, 2.0 Hz, 1H), 5.67 (dq, J = 15.5, 6.0 Hz, 1H), 4.17 – 4.02 (m, 1H), 3.17 – 2.90 (m, 2H); **^{13}C NMR** (100 MHz, $CDCl_3$) δ = 138.6 (q, J = 6.4 Hz), 135.8, 130.1 (q, J = 305.6 Hz), 129.4, 128.9, 127.7, 122.4 (q, J = 268.0 Hz), 120.8 (q, J = 34.5 Hz), 47.0 (q, J = 1.6 Hz), 40.2; **^{19}F NMR** (377 MHz, $CDCl_3$) δ = -39.01 (s, 3F), -64.62 – -64.66 (m, 3F) ppm. **EI-MS (m/z, relative intensity)** 300 (M^+, 0.32), 281 ($[M-F]^+$, 0.22), 199 (4), 129 (3), 91 (100), 69 (3), 65 (6). **ORD** ($CHCl_3$, c 0.1, 25.9 °C): -5.

Determination of *ee* by Chiral GC: Column Chiraldex β-DM, 50 °C to 170 °C at 1°C/min, *t*R: 31.71 min (minor enantiomer), 33.38 min (major enantiomer); *ee* (major enantiomer) = 93%.

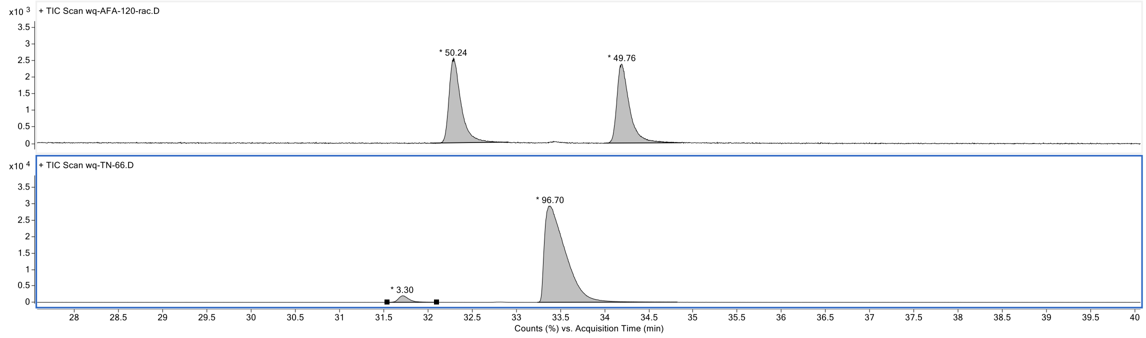

Area	Height	Type	Peak	RT	Area %	Area Sum %	Saturated	Width	FWHM	SNR	RI
550524.47	66021.29	M	1	31.707	3.42	3.3		0.562	0.124		
16119340.21	976310.61	M	2	33.381	100	96.7		1.586	0.255		

(*R*,*E*)-(8-Chloro-1,1,1-trifluorooct-2-en-4-yl)(trifluoromethyl)sulfane (3c)

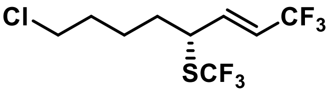

This compound was obtained according to the general procedure A. Product **3c** was isolated in 67% yield (60.3 mg) as a colorless oil by silica gel chromatography using pentane as eluent. **^{1}H NMR** (400 MHz, $CDCl_3$): δ = 6.34 (ddq, J = 15.5, 9.0, 2.1 Hz, 1H), 5.81 (dq, J = 15.7, 6.6, 1H), 3.88 – 3.77 (m, 1H), 3.55 (t, J = 6.4 Hz, 2H), 1.89 – 1.70 (m, 4H), 1.70 – 1.55 (m, 2H); **^{13}C NMR** (100 MHz, $CDCl_3$) δ = 139.1 (q, J = 6.6 Hz), 130.1 (d, J = 307.5 Hz), 122.5 (q, J = 266.1 Hz), 120.7 (q, J = 33.0 Hz), 45.7 (q, J = 2.2 Hz), 44.4, 32.8, 31.9, 24.2; **^{19}F NMR** (377 MHz, $CDCl_3$) δ = -39.07 (s, 3F), -64.53 (d, J = 6.1 Hz, 3F) ppm. **EI-MS (m/z, relative intensity)** 300 (M^+, 0.12), 281 ($[M-F]^+$, 0.07), 195 (17), 163 (100), 122 (22),115 (22), 91 (29), 55 (42), 41 (19). **ORD** ($CHCl_3$, c 0.1, 25.4 °C): -13.

Determination of *ee* by Chiral GC: Column Chiraldex β-DM, 50 °C to 170 °C at 1°C/min, *t*R: 29.84 min (minor enantiomer), 30.24 min (major enantiomer); *ee* (major enantiomer) = 88%.

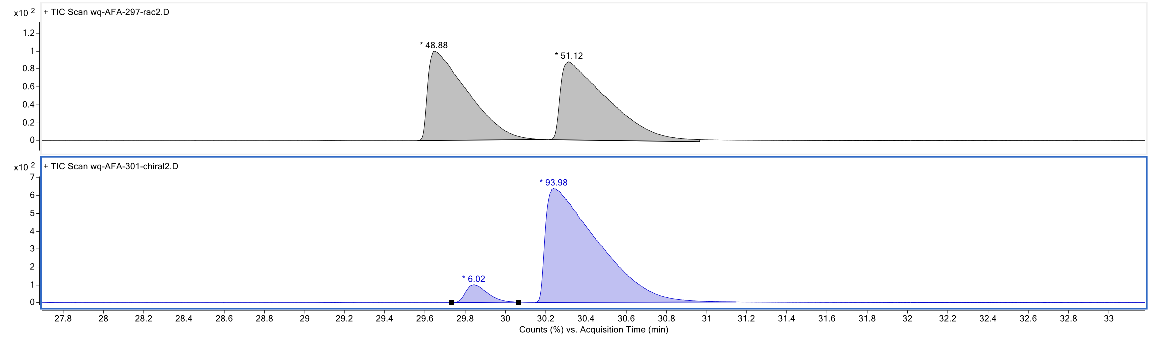

Area	Height	Type	Peak	RT	Area %	Area Sum %	Saturated	Width	FWHM	SNR	RI
2033222.29	271529.5	M	1	29.842	6.41	6.02		0.334	0.118		
31730548.42	1755123.17	M	2	30.239	100	93.98		1.002	0.282		

(*R*,*E*)-6,6,6-Trifluoro-3-((trifluoromethyl)thio)hex-4-en-1-yl 4-methylbenzenesulfonate (3d)

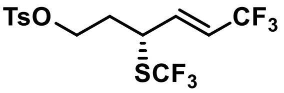

This compound was obtained according to the general procedure A but using bis(4-methoxyphenyl)selane as catalyst and MsOH as acid activator at -50 ºC for 48 h. Product **3d** was isolated in 50% yield (61.2 mg) as a colorless oil by silica gel chromatography using pentane/ethyl acetate (7/1) as eluent. **^{1}H NMR** (400 MHz, $CDCl_3$): δ = 7.79 (d, *J* = 8.5 Hz, 2H), 7.37 (d, *J* = 8.0 Hz, 2H), 6.24 (ddq, *J* = 15.4, 9.0, 2.1 Hz, 1H), 5.70 (dq, *J* = 15.6, 6.4, 1H), 4.18 (ddd, *J* = 11.4, 6.5, 4.8 Hz, 1H), 4.07 (ddd, *J* = 10.5, 6.7, 4.8 Hz, 1H), 4.00 – 3.86 (m, 1H), 2.46 (s, 3H), 2.14 – 1.96 (m, 2H); **^{13}C NMR** (100 MHz, $CDCl_3$) δ = 145.6, 137.7 (q, *J* = 6.3 Hz), 132.6, 130.2, 129.8 (q, *J* = 307.9 Hz), 128.1, 122.2 (d, *J* = 269.9 Hz), 121.6 (q, *J* = 34.5), 65.9, 41.8 (q, *J* = 2.5 Hz), 32.7, 21.8; **^{19}F NMR** (377 MHz, $CDCl_3$) δ = -38.77 (s, 3F), -64.65 (d, *J* = 6.1 Hz, 3F) ppm. **HRMS (pos. ESI)** m/z: calcd for $C_{14}H_{14}F_6NaO_3S_2^+$ [M+Na]$^+$ 431.0181, found 431.0160. **ORD** ($CHCl_3$, c 0.1, 26.7 ºC): -18.

Determination of *ee* by Chiral SFC: Diacel CHIRALPAK OJ-H, 25 °C, 0.3 cm ϕ, 20 cm column, 5% MeOH in CO_2, flow rate: 0.8 mL/min; *t*R: 3.03 min (major enantiomer); *ee* (major enantiomer) = >99%.

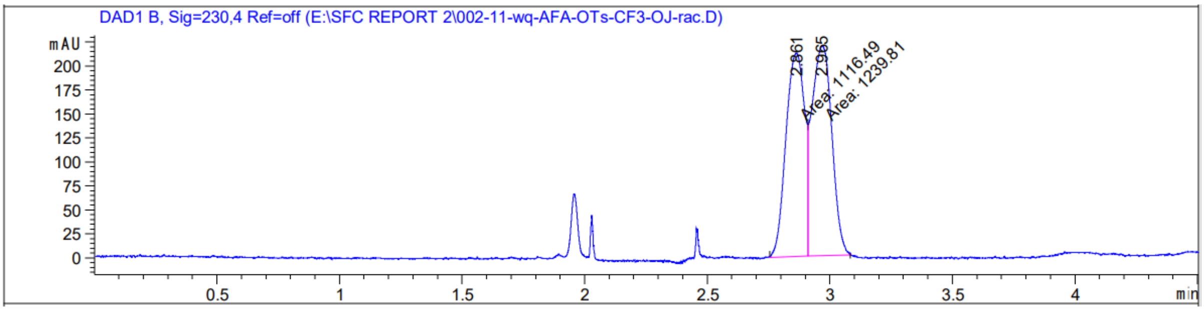

Signal 2: DAD1 B, Sig=230,4 Ref=off

Peak #	RetTime [min]	Type	Width [min]	Area [mAU*s]	Height [mAU]	Area %
1	2.861	MM T	0.0879	1116.48657	211.76253	47.3831
2	2.965	MM T	0.0946	1239.81042	218.43401	52.6169

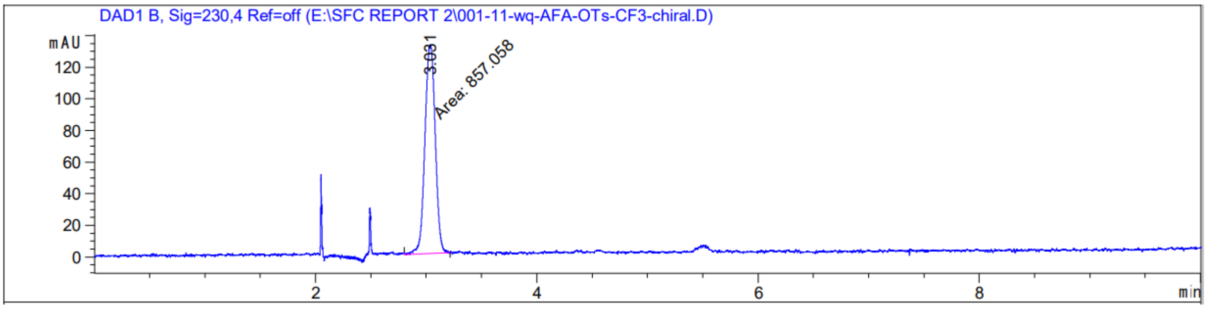


```
Signal 2: DAD1 B, Sig=230,4 Ref=off

Peak RetTime Type  Width     Area       Height      Area
  #   [min]        [min]   [mAU*s]      [mAU]        %
----|-------|----|-------|----------|----------|--------|
   1   3.031 MM T  0.1082  857.05847  132.04788 100.0000
```

(*S*,*E*)-2-(5,5,5-Trifluoro-2-((trifluoromethyl)thio)pent-3-en-1-yl)isoindoline-1,3-dione (3e)

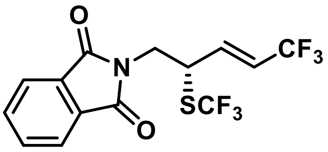

This compound was obtained according to the general procedure A. Product **3e** was isolated in 70% yield (77.5 mg, γ/α = 16/1) as a white solid by silica gel chromatography using pentane/ethyl acetate (5/1) as eluent. **^{1}H NMR** (400 MHz, $CDCl_3$): δ = 7.89 (dd, J = 5.5, 3.1 Hz, 2H), 7.77 (dd, J = 5.5, 3.0 Hz, 2H), 6.40 (ddq, J = 15.6, 8.8, 2.0 Hz, 1H), 5.91 (dqd, J = 15.6, 6.0, 1.0 Hz, 1H), 4.42 – 4.30 (m, 1H), 4.07 – 3.92 (m, 2H); **^{13}C NMR** (100 MHz, $CDCl_3$) δ = 167.8, 135.9 (q, J = 6.4 Hz), 134.7, 131.6, 129.9 (q, J = 307.9 Hz), 123.9, 122.1 (q, J = 268.4 Hz), 122.7 (q, J = 34.6 Hz), 44.1 (q, J = 1.7 Hz), 40.0; **^{19}F NMR** (377 MHz, $CDCl_3$) δ = -39.00 (s, 3F), -64.70 – -65.47 (m, 3F) ppm. **HRMS (pos. ESI)** m/z: calcd for $C_{14}H_9F_6NNaO_2S^+$ $[M+Na]^+$ 392.0150, found 392.0159. **ORD** ($CHCl_3$, c 0.1, 26.5 °C): -26.

Determination of *ee* by Chiral GC: Column Chiraldex β-6TBDM, 50 °C to 170 °C at 1°C/min, *t*R: 118.38 min (major enantiomer), 118.70 min (minor enantiomer); *ee* (major enantiomer) = 97%.

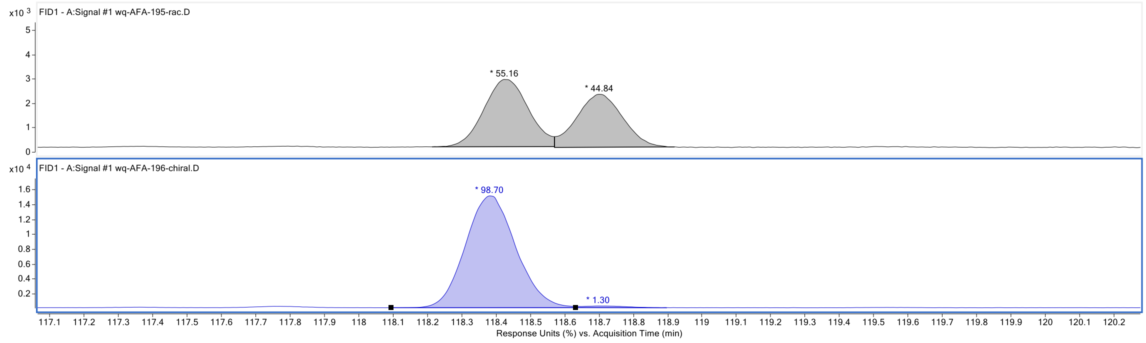

Area	Height	Type	Peak	RT	Area %	Area Sum %	Saturated	Width	FWHM	SNR	RI
13446.94	1497.24	M	2	118.697	1.31	1.3		0.26	0.149		
1024380.48	100228.31	M	1	118.38	100	98.7		0.537	116.504		

Crystals of **3e** were obtained by dissolving approx. 5 mg of pure substance in 5 mL of pentane in a glass tube. Storage at 5 °C for 3 days resulted in the growth of crystalline needles.

CCDC deposition number: 2173301

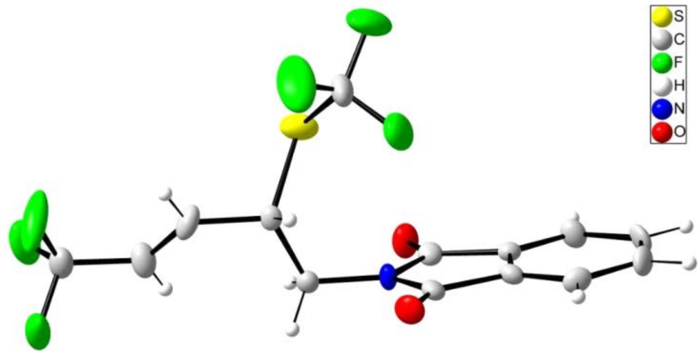

(*R*,*E*)-(1-Cyclohexyl-4,4,4-trifluorobut-2-en-1-yl)(trifluoromethyl)sulfane (3f)

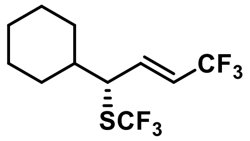

This compound was obtained according to the general procedure A. Product **3f** was isolated in 80% yield (70.1 mg) as a colorless oil by silica gel chromatography using pentane as eluent. **^{1}H NMR** (400 MHz, $CDCl_3$): δ = 6.36 (ddq, J = 15.8, 9.5, 2.1 Hz, 1H), 5.77 (dq, J = 15.7, 6.2 Hz, 1H), 3.68 (dd, J = 9.5, 6.2 Hz, 1H), 1.91 – 1.59 (m, 6H), 1.33 – 0.95 (m, 5H); **^{13}C NMR** (100 MHz, $CDCl_3$) δ = 138.4 (q, J = 6.5 Hz), 130.6 (q, J = 307.0 Hz), 122.6 (q, J = 269.6 Hz), 120.7 (q, J = 34.1 Hz), 52.1 (q, J = 1.2 Hz), 41.4, 30.5, 30.3, 26.1, 26.03, 26.00; **^{19}F NMR** (377 MHz, $CDCl_3$) δ = -39.20 (s, 3F), -64.38 (dd, J = 6.4, 2.2 Hz, 3F) ppm. **EI-MS (m/z, relative intensity)** 149 (5), 127 (3), 83(100), 69 (7), 55 (48), 41 (14). **ORD** ($CHCl_3$, c 0.1, 25.5 ºC): -19.

Determination of *ee* by Chiral GC: Column Chiraldex β-DM, 50 °C, 60 min, *t*R: 35.14 min (minor enantiomer), 39.56 min (major enantiomer); *ee* (major enantiomer) = 97.5%.

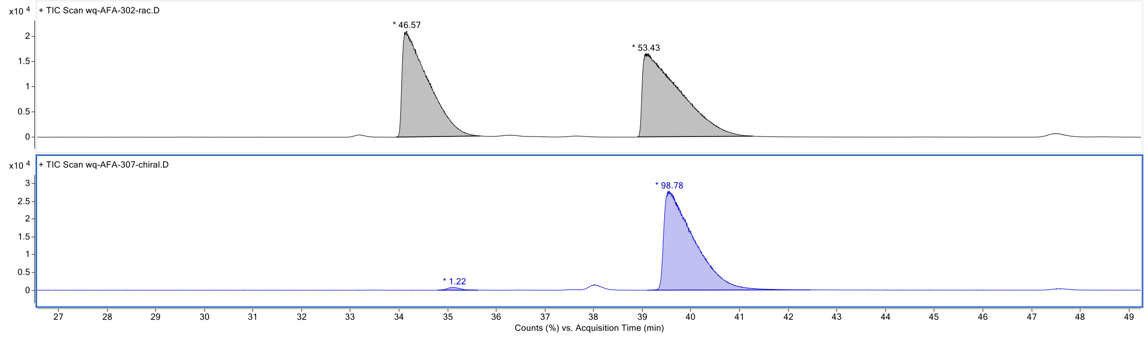

Area	Height	Type	Peak	RT	Area %	Area Sum %	Saturated	Width	FWHM	SNR	RI
299539.68	16421.98	M	1	35.14	1.24	1.22		0.829	30.092		
24168423.26	563409.69	M	2	39.555	100	98.78		3.362	0.657		

(*S*,*E*)-(4,4,4-Trifluoro-1-phenylbut-2-en-1-yl)(trifluoromethyl)sulfane (3g)

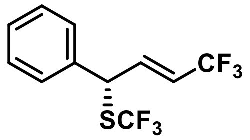

This compound was obtained according to the general procedure A but using bis(4-methoxyphenyl)selane as catalyst and MsOH as acid activator at -50 ºC for 48 h. Product **3g** was isolated in 53% yield (45.5 mg) as a colorless oil by silica gel chromatography using pentane as eluent. **^{1}H NMR** (400 MHz, $CDCl_3$): δ = 7.48 – 7.31 (m, 5H), 6.73 (ddq, J = 15.6, 7.6, 2.0 Hz, 1H), 5.88 (dqd, J = 15.6, 6.2, 1.3 Hz, 1H), 5.10 (d, J = 7.8 Hz,

1H); **^{13}C NMR** (100 MHz, $CDCl_3$) δ = 138.01 (d, J = 6.4 Hz), 135.7, 129.6, 129.2, 127.9, 129.8 (q, J = 308.5 Hz), 122.6 (q, J = 270.0 Hz), 121.3 (q, J = 34.5 Hz), 49.6 (d, J = 2.1 Hz); **^{19}F NMR** (377 MHz, $CDCl_3$) δ = -40.09 (s, 3F), -64.21 – -64.74 (m, 3F) ppm. **EI-MS (m/z, relative intensity)** 286 (M^+, 2), 217 ($[M\text{-}CF_3]^+$, 1.3), 185 ($[M\text{-}SCF_3]^+$, 100), 165 (66), 145 (20), 115 (40). **ORD** ($CHCl_3$, c 0.1, 26.7 ºC): -47.

Determination of *ee* by Chiral GC: Column Chiraldex β-DM, 50 °C to 170 °C at 1°C/min, *t*R: 19.82 min (minor enantiomer), 20.39 min (major enantiomer); *ee* (major enantiomer) = 98%.

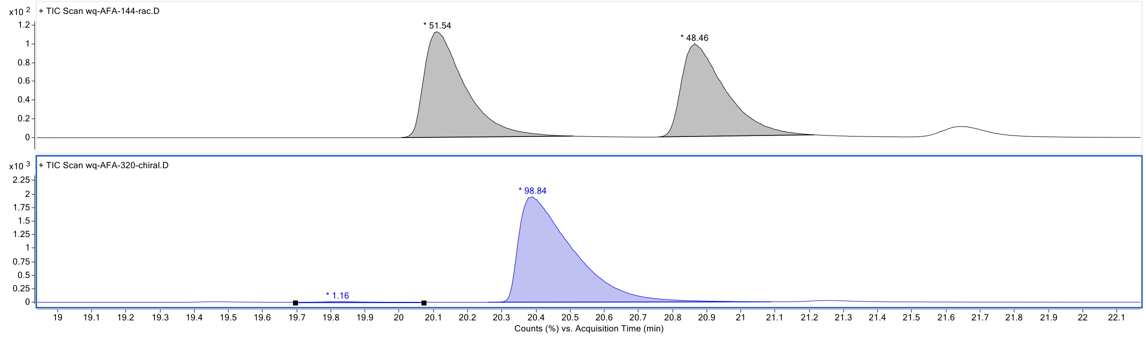

Area	Height	Type	Peak	RT	Area %	Area Sum %	Saturated	Width	FWHM	SNR	RI
337566.93	27803.07	M	1	19.82	1.18	1.16		0.376	5.04		
28644633.48	2620561.37	M	2	20.391	100	98.84		0.829	0.163		

(S,*E*)-(6,6,6-trifluoro-2,2-dimethylhex-4-en-3-yl)(trifluoromethyl)sulfane (3h)

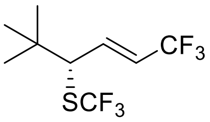

This compound was obtained according to the general procedure A. Product **3h** was isolated in 64% yield (51 mg) as a colorless oil by silica gel chromatography using pentane as eluent. **^{1}H NMR** (400 MHz, $CDCl_3$): δ = 6.43 (ddq, J = 14.5, 10.3, 2.1 Hz, 1H), 5.76 (dq, J = 15.5, 6.2 Hz, 1H), 3.57 (d, J = 10.2 Hz, 1H), 1.05 (s, 9H); **^{13}C NMR** (100 MHz, $CDCl_3$) δ = 137.6 (q, J = 6.5 Hz), 130.7 (d, J = 306.6 Hz), 122.5 (d, J = 268.2 Hz), 120.9 (q, J = 34.1 Hz), 57.5, 34.7, 27.7; **^{19}F NMR** (377 MHz, $CDCl_3$) δ = -39.35 (s, 3F), -64.43 (dd, J = 6.1, 2.0 Hz, 3F) ppm. **EI-MS (m/z, relative intensity)** 251 (6), 209 (1), 182(1), 177 (4), 165 (2), 149 (6), 57 (100). **ORD** ($CHCl_3$, c 0.2, 25.5 ºC): -20.

Determination of *ee* by Chiral GC: Column Chiraldex β-DM, 30 °C, 20 min, *t*R: 6.18 min (minor enantiomer), 6.23 min (major enantiomer); *ee* (major enantiomer) = 98%.

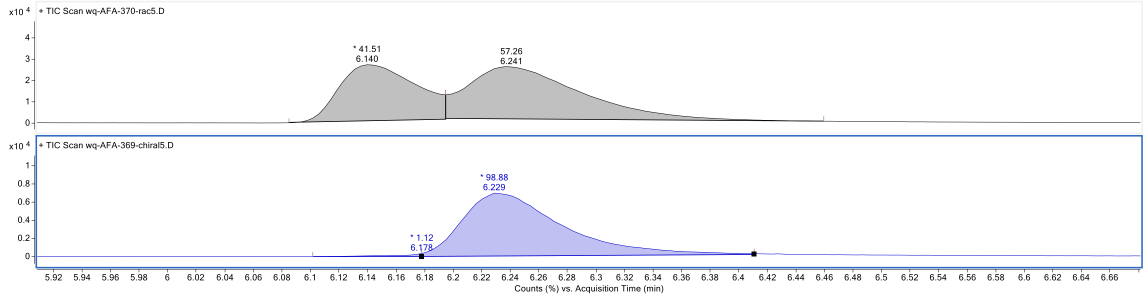

General procedure B for the difluoromethylthiolation

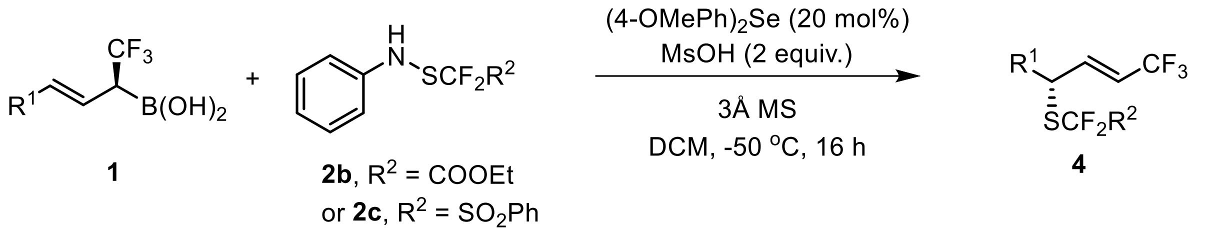

Scheme S3. Synthesis of allyl difluoromethyl thioester 4a-4e.

In the glovebox, an oven-dried microwave vial was charged with the stock solution of allylboronic acid **1** (0.1 mmol, 1mL) and 3Å molecular sieves (20 mg). The reaction vial was tightly sealed with rubber septum and transferred to a -50 °C cooling bath. To the reaction mixture was added selenide catalyst (0.02 mmol, dissolved in 0.1 mL DCM), MsOH (0.2 mmol) and $PhNHSCF_2R$ (0.15 mmol) sequentially by syringe. Then, the reaction mixture was stirred at -50 °C for 16 h. After quenching by adding water, the NMR of the crude reaction mixture was recorded using trifluorotoluene as an internal standard. The product was isolated by flash chromatography with pentane/Et_2O system.

Ethyl (*R*,*E*)-2,2-difluoro-2-((1,1,1-trifluorodec-2-en-4-yl)thio)acetate (4a)

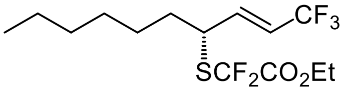

This compound was obtained according to the general procedure B using **2b** as reagent. Product **4a** was isolated in 60% yield (20.9 mg) as a colorless oil by silica gel chromatography using pentane/Et_2O (25/1) as eluent. **^{1}H NMR** (400 MHz, $CDCl_3$): δ = 6.33 (ddq, J = 15.3, 8.8, 2.1 Hz, 1H), 5.79 (dqd, J = 15.6, 6.7, 5.8 Hz, 1H), 4.35 (q, J = 7.1 Hz, 2H), 3.98 – 3.77 (m, 1H), 1.79 – 1.59 (m, 2H), 1.47 – 1.19 (m, 11H), 0.94 – 0.85 (m, 3H); **^{13}C NMR** (100 MHz, $CDCl_3$) δ = 161.6 (t, J = 32.0 Hz), 140.19 (q, J = 6.4 Hz), 122.8 (q, J = 270.0 Hz), 120.3 (t, J = 285.2 Hz), 119.77 (q, J = 34.0 Hz), 64.0, 44.6 (t, J = 2.4 Hz), 34.1, 31.6, 28.8, 26.8, 22.7, 14.1, 14.0; **^{19}F NMR** (377 MHz, $CDCl_3$) δ = -64.03 – -64.80 (m, 3F), -80.04 (d, J = 221.4 Hz, 1F), -82.08 (d, J = 221.4 Hz, 1F) ppm. **HRMS (pos. ESI)** m/z: calcd for $C_{14}H_{21}F_5NaO_2S^+$ $[M+Na]^+$ 371.1075, found 371.1076. **ORD** ($CHCl_3$, c 0.27, 27.6 °C): +5.9.

Determination of *ee* by chiral GC: Column Chiraldex β-DM, 90 °C, 120 min, *t*R: 58.04 min (major enantiomer); *ee* (major enantiomer) = >99%.

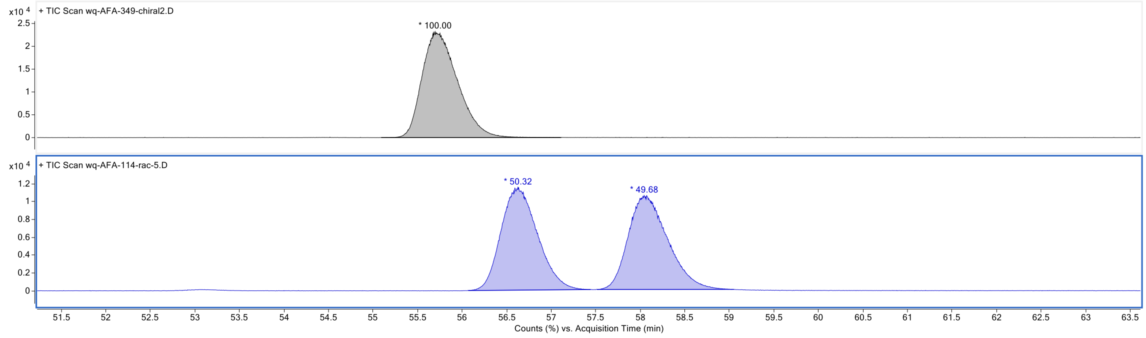

Area	Height	Type	Peak	RT	Area %	Area Sum %	Saturated	Width	FWHM	SNR	RI
14911046.28	486666.69	M	2	58.043	98.73	49.68		1.535	0.478		
15102789.43	531984.07	M	1	56.627	100	50.32		1.374	0.448		

Ethyl (*R,E*)-2,2-difluoro-2-((5,5,5-trifluoro-1-phenylpent-3-en-2-yl)thio)acetate (4b)

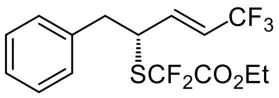

This compound was obtained according to the general procedure B using **2b** as reagent. Product **4b** was isolated in 56% yield (19.8 mg) as a colorless oil by silica gel chromatography using pentane/Et_2O (25/1) as eluent. **^{1}H NMR** (400 MHz, $CDCl_3$): δ = 7.37 – 7.23 (m, 3H), 7.22 – 7.08 (m, 2H), 6.36 (ddq, J = 15.4, 8.7, 2.2 Hz, 1H), 5.68 (dq, J = 15.6, 6.2 Hz, 1H), 4.31 (q, J = 7.1 Hz, 2H), 4.13 (q, J = 7.8 Hz, 1H), 3.08 (dd, J = 14.0, 6.5 Hz, 1H), 2.97 (dd, J = 14.0, 8.1 Hz, 1H), 1.34 (t, J = 7.1 Hz, 3H); **^{13}C NMR** (100 MHz, $CDCl_3$) δ = 161.5 (t, J = 32.5 Hz), 139.2 (q, J = 6.5 Hz), 136.2, 129.5, 128.8, 127.5, 122.6 (q, J = 269.8 Hz), 120.5 (q, J = 34.2 Hz), 120.2 (t, J = 286.1 Hz), 64.0, 45.7 (t, J = 2.5 Hz), 40.9, 14.0; **^{19}F NMR** (377 MHz, $CDCl_3$) δ = -64.16 – -64.87 (m, 3F), -80.28 (d, J = 221.7 Hz, 1F), -82.19 (d, J = 221.5 Hz, 1F) ppm. **HRMS (pos. ESI)** m/z: calcd for $C_{15}H_{15}F_5NaO_2S^+$ $[M+Na]^+$ 377.0605, found 377.0608. **ORD** ($CHCl_3$, c 0.36, 27.6 ºC): +1.1.
Determination of *ee* by Chiral GC: Column Chiraldex β-DM, 90 °C, 120 min, *t*R: 123.33 min (major enantiomer); *ee* (major enantiomer) = >99%.

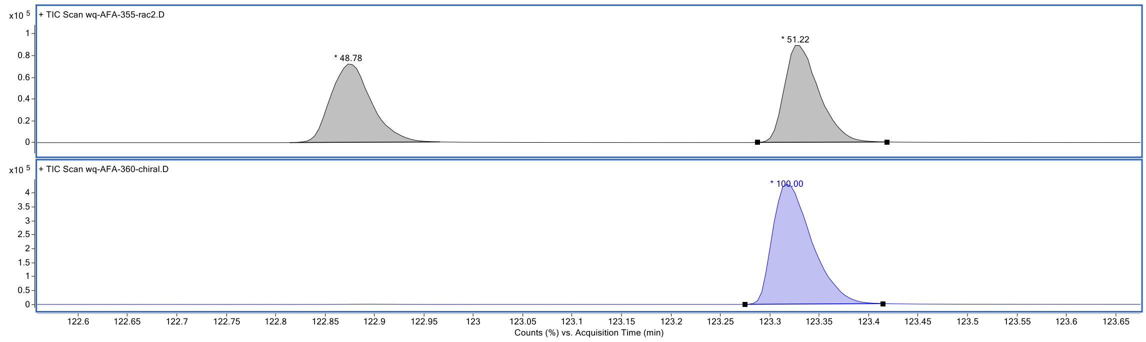

Area	Height	Type	Peak	RT	Area %	Area Sum %	Saturated	Width	FWHM	SNR	RI
13493147.68	4704450.03	M	1	122.873	95.23	48.78		0.152	0.044		
14169244.99	5818677.47	M	2	123.326	100	51.22		0.131	0.037		

Ethyl (*R,E*)-2-((1-cyclohexyl-4,4,4-trifluorobut-2-en-1-yl)thio)-2,2-difluoroacetate (4c)

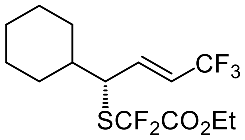

This compound was obtained according to the general procedure B using **2b** as reagent. Product **4c** was isolated in 54% yield (18.7 mg) as a colorless oil by silica gel chromatography using pentane/Et_2O (25/1) as eluent. **^{1}H NMR** (400 MHz, $CDCl_3$): δ = 6.36 (ddq, J = 15.6, 9.3, 2.1 Hz, 1H), 5.79 (dtd, J = 15.5, 6.7, 5.8 Hz, 1H), 4.34 (q, J = 7.1 Hz, 2H), 3.78 – 3.70 (m, 1H), 1.90 – 1.57 (m, 6H), 1.36 (t, J = 7.2 Hz, 3H), 1.31 – 0.95 (m, 5H); **^{13}C NMR** (100 MHz, $CDCl_3$) δ = 161.7 (t, J = 32.8 Hz), 139.0 (q, J = 6.5 Hz), 122.8 (q, J = 269.5 Hz), 120.4 (dd, J = 288.4, 285.9 Hz), 120.3 (q, J = 33.9 Hz), 63.9, 50.6 (t, J = 2.8 Hz), 41.9, 30.3, 30.2, 26.11, 26.08, 26.05, 14.0; **^{19}F NMR** (377 MHz, $CDCl_3$) δ = -64.09 – -64.20 (m, 3F), -80.32 (d, J = 220.4 Hz, 1F), -82.80 (d, J = 220.4 Hz, 1F) ppm. **HRMS (pos. ESI)** m/z: calcd for $C_{14}H_{19}F_5NaO_2S^+$ $[M+Na]^+$ 369.0918, found 369.0911. **ORD** ($CHCl_3$, c 0.1, 26.7 ºC): +1.
Determination of *ee* by Chiral GC: Column Chiraldex β-DM, 90 °C, 120 min, *t*R: 80.06 min (major enantiomer); *ee* (major enantiomer) = >99%.

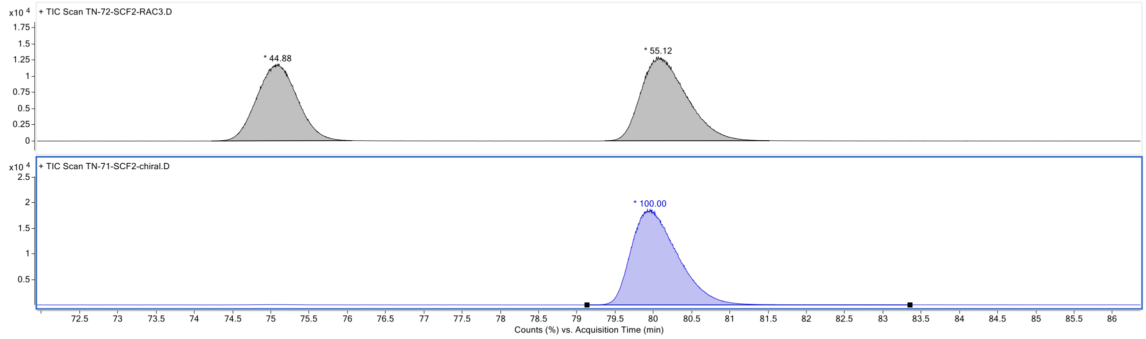

Area	Height	Type	Peak	RT	Area %	Area Sum %	Saturated	Width	FWHM	SNR	RI
11352404.72	309717.21	M	1	75.089	81.71	44.97		1.704	0.589		
13892756.03	340217.76	M	2	80.062	100	55.03		1.899	0.642		

Ethyl (*S*,*E*)-2,2-difluoro-2-((4,4,4-trifluoro-1-phenylbut-2-en-1-yl)thio)acetate (4d)

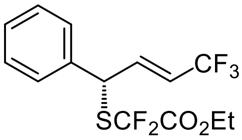

This compound was obtained according to the general procedure B using **2b** as reagent. Product **4d** was isolated in 40% yield (13.6 mg) as a colorless oil by silica gel chromatography using pentane/Et_2O (25/1) as eluent. **^{1}H NMR** (400 MHz, $CDCl_3$): δ = 7.43 – 7.28 (m, 5H), 6.70 (ddq, J = 15.5, 7.0, 2.2 Hz, 1H), 5.92 – 5.76 (m, 1H), 5.27 – 5.06 (m, 1H), 4.30 (q, J = 7.1 Hz, 2H), 1.34 (t, J = 7.1 Hz, 3H); **^{13}C NMR** (100 MHz, $CDCl_3$) δ = 161.4 (t, J = 32.3 Hz), 138.8 (q, J = 6.5 Hz), 136.7, 129.4, 128.8, 128.1, 122.8 (q, J = 269.8 Hz), 121.0 (q, J = 34.2 Hz), 120.0 (t, J = 288.5 Hz), 64.1, 48.3 (t, J = 3.1 Hz), 14.0; **^{19}F NMR** (377 MHz, $CDCl_3$) δ = -64.15 – -64.36 (m, 3F), -80.80 (d, J = 221.5 Hz, 1F), -82.70 (d, J = 221.5 Hz, 1F) ppm. **HRMS (pos. ESI)** m/z: calcd for $C_{14}H_{13}F_5NaO_2S^+$ $[M+Na]^+$ 363.0449, found 363.0446. **ORD** ($CHCl_3$, c 0.26, 27.5 °C): -40.4.

Determination of *ee* by Chiral GC: Column Chiraldex β-DM, 90 °C, 120 min, *t*R: 71.17 min (major enantiomer); *ee* (major enantiomer) = >99%.

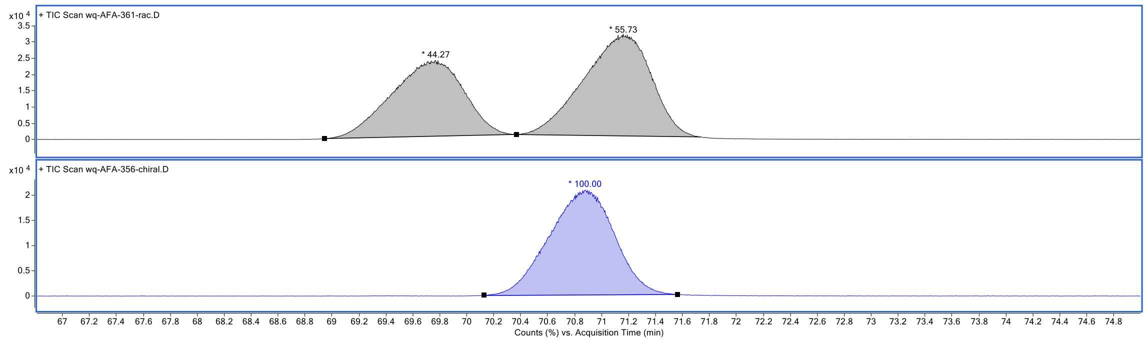

Area	Height	Type	Peak	RT	Area %	Area Sum %	Saturated	Width	FWHM	SNR	RI
9268730.29	250637.46	M	1	69.769	79.43	44.27		1.425	0.607		
11669221.38	336395.73	M	2	71.161	100	55.73		1.357	1.889		

(*R*,*E*)-(Difluoro(phenylsulfonyl)methyl)(1,1,1-trifluorodec-2-en-4-yl)sulfane (4e)

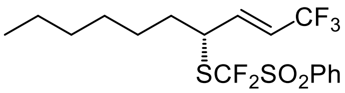

This compound was obtained according to the general procedure B using **2c** as reagent. Product **4e** was isolated in 48% yield (20.0 mg) as a colorless oil by silica gel chromatography using pentane/Et_2O (20/1) as eluent. **^{1}H NMR** (400 MHz,

$CDCl_3$): δ = 8.03 – 7.86 (m, 2H), 7.85 – 7.69 (m, 1H), 7.71 – 7.57 (m, 2H), 6.35 (ddq, J = 15.3, 8.8, 2.1 Hz, 1H), 5.83 (dq, J = 15.7, 6.3 Hz, 1H), 4.18 (q, J = 7.8 Hz, 1H), 1.88 – 1.63 (m, 2H), 1.50 – 1.16 (m, 8H), 0.96 – 0.76 (m, 3H); **^{13}C NMR** (100 MHz, $CDCl_3$) δ = 139.8 (q, J = 6.5 Hz), 135.8, 132.2, 131.0, 129.6, 129.3 (dd, J = 327.7, 320.8 Hz), 122.7 (q, J = 269.5 Hz), 120.3 (q, J = 34.2 Hz), 46.5 (t, J = 2.5 Hz), 34.2, 31.6, 28.8, 26.8, 22.6, 14.2; **^{19}F NMR** (377 MHz, $CDCl_3$) δ = -63.50 – -65.04 (m, 3F), -76.91 (d, J = 210.1 Hz, 1F), -78.79 (d, J = 209.9 Hz, 1F) ppm. **HRMS (pos. ESI)** m/z: calcd for $C_{17}H_{21}F_5NaO_2S_2^+$ $[M+Na]^+$ 439.0795, found 439.0793. **ORD** ($CHCl_3$, c 0.28, 27.6 ºC): +12.9.

Determination of *ee* by Chiral SFC: Diacel CHIRALPAK OJ-H, 25 °C, 0.3 cm ϕ, 20 cm column, 5% MeOH in CO_2, flow rate: 0.8 mL/min; *t*R: 3.44 min (minor enantiomer), 3.91 min (major enantiomer); *ee* (major enantiomer) = 95%.

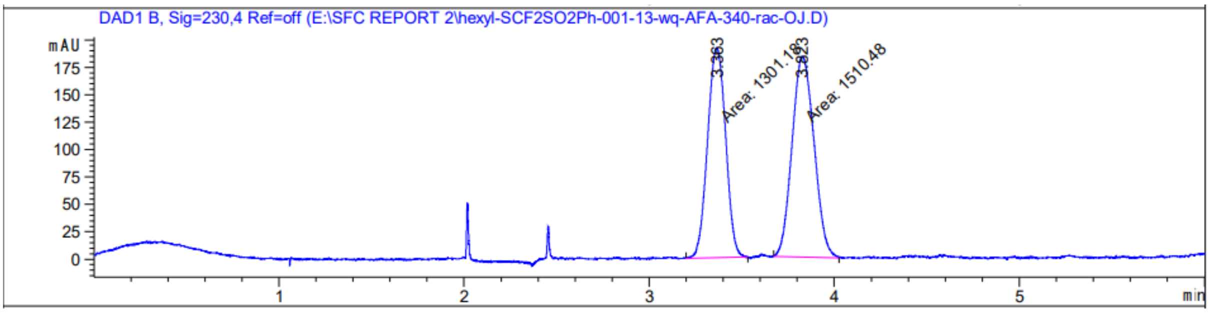

Signal 2: DAD1 B, Sig=230,4 Ref=off

Peak #	RetTime [min]	Type	Width [min]	Area [mAU*s]	Height [mAU]	Area %
1	3.363	MM T	0.1298	1301.17859	191.43932	46.2780
2	3.823	MM T	0.1376	1510.47693	182.95610	53.7220

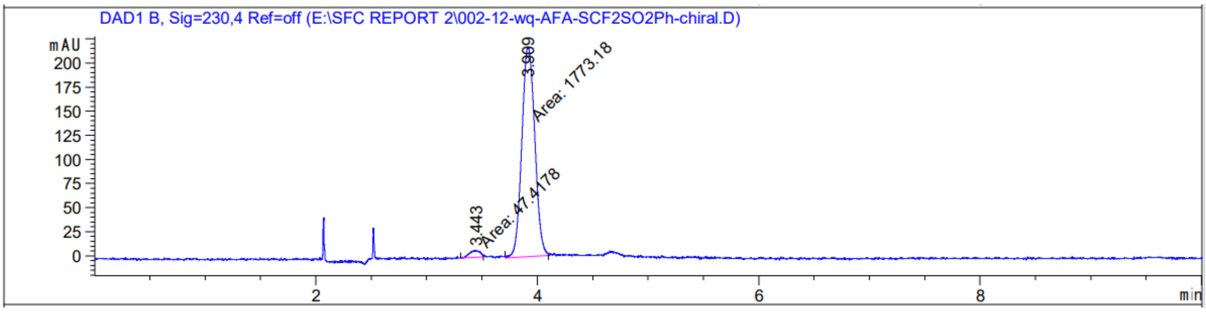

Signal 2: DAD1 B, Sig=230,4 Ref=off

Peak #	RetTime [min]	Type	Width [min]	Area [mAU*s]	Height [mAU]	Area %
1	3.443	MM T	0.1172	47.41784	7.33777	2.6045
2	3.909	MM T	0.1359	1773.18481	217.53798	97.3955

General procedure C for the thiocyanation

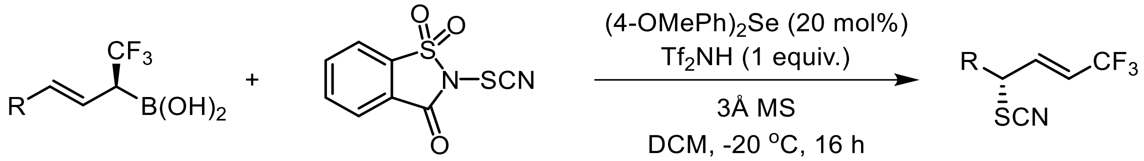

1 **2d** **5**

Scheme S5. Synthesis of allyl aryl thioester 5a-5c.

An oven-dried microwave vial was charged with 2-thiocyanatobenzo[*d*]isothiazol-3(2*H*)-one 1,1-dioxide **2d** (0.15 mmol). The reaction vial was brought into the glovebox and triflimide (0.1 mmol) and 3Å molecular sieves (20 mg) were added. The reaction vial was tightly sealed with rubber septum and transferred to a -20 °C cooling bath. To the reaction mixture was added selenide catalyst (0.02 mmol, dissolved in 0.1 mL DCM) and the stock solution of allylboronic acid **1** (0.1 mmol, 1 mL) sequentially by syringe. Then, the reaction mixture was stirred at -20 °C for 16 h. After quenching by adding water, the NMR of the crude reaction mixture was recorded using trifluorotoluene as an internal standard. The product was isolated by flash chromatography with appropriate eluent.

(*R*,*E*)-1,1,1-Trifluoro-4-thiocyanatodec-2-ene (5a)

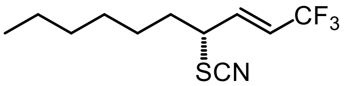

This compound was obtained according to the general procedure C. Product **5a** was isolated in 81% yield (20.3 mg) as a colorless oil by silica gel chromatography using pentane/Et_2O (25/1) as eluent. **^{1}H NMR** (400 MHz, $CDCl_3$): δ = 6.36 (ddq, *J* = 15.6, 9.0, 2.2 Hz, 1H), 5.89 (dq, *J* = 15.6, 6.1 Hz, 1H), 3.77 (q, *J* = 7.9 Hz, 1H), 1.91 – 1.76 (m, 2H), 1.52 – 1.22 (m, 8H), 0.89 (t, *J* = 6.6 Hz, 3H); **^{13}C NMR** (100 MHz, $CDCl_3$) δ = 137.0 (q, *J* = 6.4 Hz), 122.2 (q, *J* = 270.0 Hz), 122.1 (q, *J* = 34.6 Hz), 110.1, 49.6, 33.7, 31.5, 28.7, 27.1, 22.6, 14.1; **^{19}F NMR** (377 MHz, $CDCl_3$) δ = -64.28 – -64.38 (m, 3F) ppm. **HRMS (pos. ESI)** m/z: calcd for $C_{11}H_{16}F_3NNaS^+$ $[M+Na]^+$ 274.0848, found 274.0836. **ORD** ($CHCl_3$, c 0.1, 25.8 °C): -9.

Determination of *ee* by Chiral GC: Column Chiraldex β-DM, 80 °C, 90 min; *t*R: 60.12 min (major enantiomer), 64.72 min (minor enantiomer); *ee* (major enantiomer) = 96%.

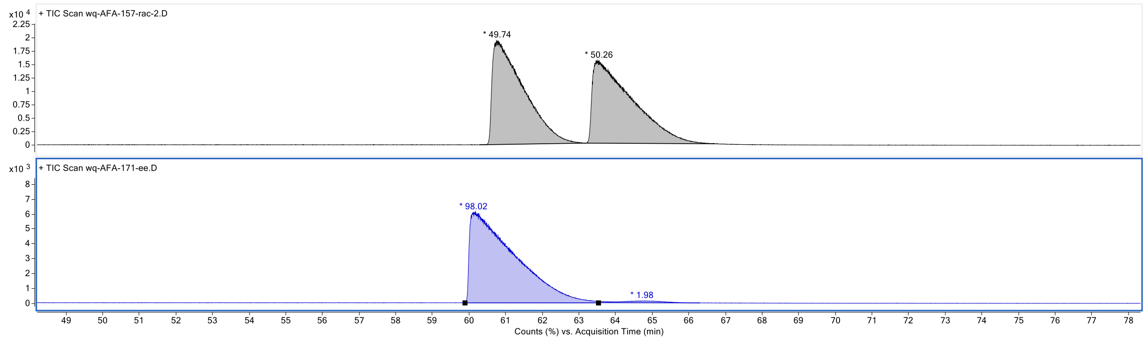

Area	Height	Type	Peak	RT	Area %	Area Sum %	Saturated	Width	FWHM	SNR	RI
1194611.44	14343.38	M	2	64.72	2.02	1.98		2.761	60.013		
59243473.32	697164.55	M	1	60.12	100	98.02		3.658	1.321		

(*S*,*E*)-2-(5,5,5-Trifluoro-2-thiocyanatopent-3-en-1-yl)isoindoline-1,3-dione (5b)

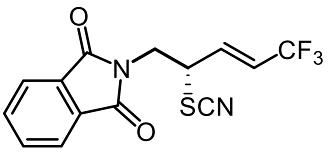

This compound was obtained according to the general procedure C. Product **5b** was isolated in 58% yield (18.9 mg, γ/α = 32/1) as a white solid by silica gel chromatography using pentane/EtOAc (3.5/1) as eluent. **^{1}H NMR** (400 MHz, $CDCl_3$): δ = 8.03 – 7.84 (m, 2H), 7.83 – 7.71 (m, 2H), 6.45 (ddq, *J* = 15.7, 8.7, 2.0 Hz, 1H), 6.02 (dqd, *J* = 15.7, 6.0, 1.0 Hz, 1H), 4.34 – 4.22 (m, 1H), 4.18 – 4.00 (m, 2H); **^{13}C NMR** (100 MHz, $CDCl_3$) δ = 167.7, 134.9, 133.5 (q, *J* = 6.6 Hz), 131.6, 124.4, 124.2 (q, J = 35.8 Hz), 124.1, 121.8 (q, *J* = 270.5 Hz), 108.7, 47.0, 40.2; **^{19}F NMR** (377 MHz, $CDCl_3$) δ = -64.71 – -64.76 (m, 3F) ppm. **HRMS (pos. ESI)** m/z: calcd for $C_{14}H_9F_3N_2NaO_2S^+$ $[M+Na]^+$ 349.0229, found 349.0232. **ORD** ($CHCl_3$, c 0.1, 26.0 ºC): -9.

Determination of *ee* by Chiral SFC: Diacel CHIRALPAK IF, 25 °C, 0.3 cm ϕ, 15 cm column, 3% MeOH in CO_2, flow rate: 1.2 mL/min; *t*R: 6.90 min (major enantiomer), 8.30 min (minor enantiomer); *ee* (major enantiomer) = 90%.

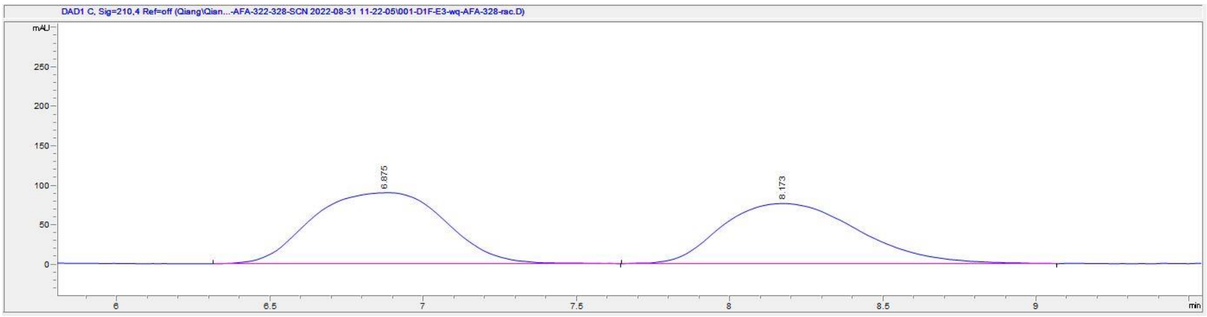

#	Time	Type	Area	Height	Width	Area%	Symmetry
1	6.875	BB	2714.8	89.8	0.4386	53.387	1.105
2	8.173	BB	2370.4	76.2	0.4811	46.613	0.786

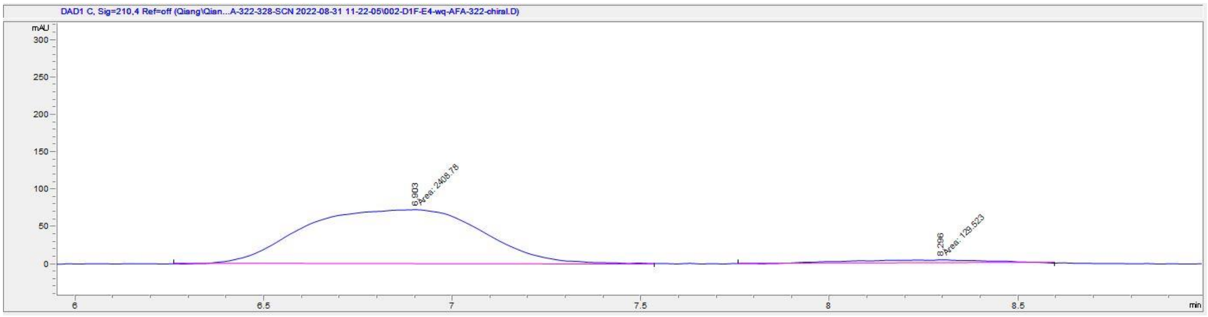

#	Time	Type	Area	Height	Width	Area%	Symmetry
1	6.903	MM T	2408.8	72.7	0.5526	94.897	0
2	8.296	MM T	129.5	4.8	0.4534	5.103	4.31E-3

(*R*,*E*)-(4,4,4-Trifluoro-1-thiocyanatobut-2-en-1-yl)cyclohexane (5c)

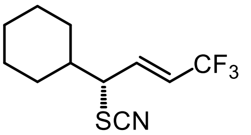

This compound was obtained according to the general procedure C. Product **5c** was isolated in 56% yield (13.9 mg) as a colorless oil by silica gel chromatography using pentane/Et_2O (25/1) as eluent. **^{1}H NMR** (400 MHz, $CDCl_3$): δ = 6.35 (ddq, *J* = 15.6, 10.0, 2.0 Hz, 1H), 5.86 (dq, *J* = 15.6, 6.1 Hz, 1H), 3.65 (dd, *J* = 10.0, 7.6 Hz, 1H), 1.97 – 1.85 (m, 1H), 1.87 – 1.62 (m, 5H), 1.37 – 0.98 (m, 5H); **^{13}C NMR** (100 MHz,

$CDCl_3$) δ = 135.8 (q, J = 6.5 Hz), 122.6 (q, J = 34.5 Hz), 122.2 (q, J = 270.2 Hz), 110.8, 56.5, 41.1, 30.7, 30.6, 25.83, 25.82; **^{19}F NMR** (377 MHz, $CDCl_3$) δ = -64.15 – -64.21 (m, 3F) ppm. **HRMS (pos. ESI)** m/z: calcd for $C_{11}H_{14}F_3NNaS^+$ $[M+Na]^+$ 272.0691, found 272.0688. **ORD** ($CHCl_3$, c 0.1, 26.3 ºC): -22.

Determination of *ee* by Chiral GC: Column Chiraldex β-DM, 80 °C, 90 min; *t*R: 92.57 min (major enantiomer), 92.64 min (minor enantiomer); *ee* (major enantiomer) = 92%.

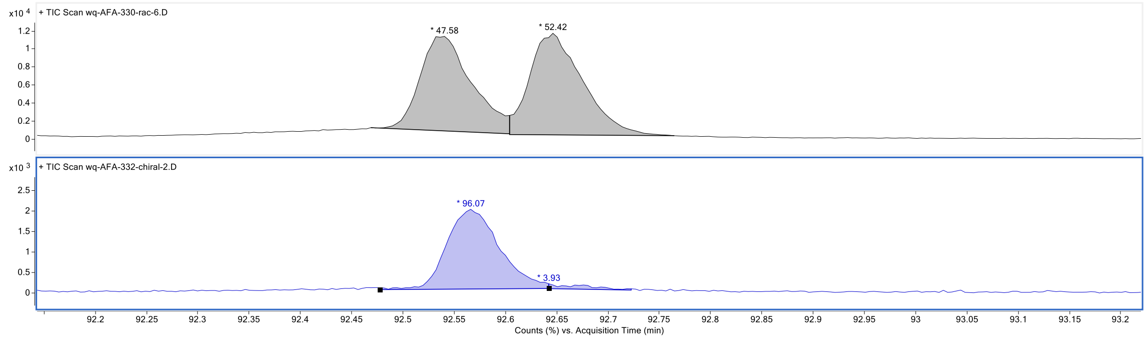

Area	Height	Type	Peak	RT	Area %	Area Sum %	Saturated	Width	FWHM	SNR	RI
5372.86	2237.76	M	2	92.642	4.1	3.93		0.08	87.548		
131192.1	36656.94	M	1	92.566	100	96.07		0.165	0.054		

General procedure D for the arylthiolation

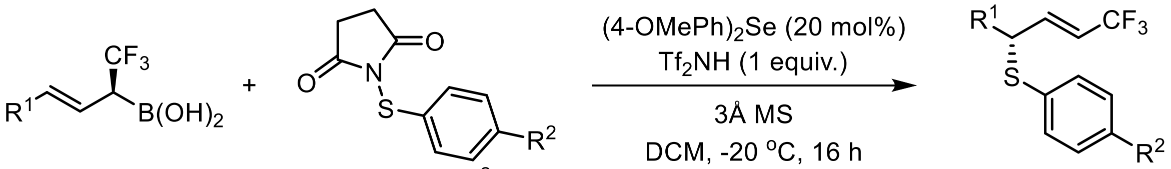

1 — **2e**, R^2 = OMe or **2f**, R^2 = H — **6**

Scheme S4. Synthesis of allyl aryl thioester 6a-6d.

An oven-dried microwave vial was charged with 1-((4-methoxyphenyl)thio)pyrrolidine-2,5-dione **2e** (0.15 mmol) or 1-(phenylthio)pyrrolidine-2,5-dione **2f** (0.15 mmol). The reaction vial was brought into the glovebox and triflimide (0.1 mmol) and 3Å molecular sieves (20 mg) were added. The reaction vial was tightly sealed with rubber septum and transferred to a -20 °C cooling bath. To the reaction mixture was added selenide catalyst (0.02 mmol, dissolved in 0.1 mL DCM) and the stock solution of allylboronic acid **1** (0.1 mmol, 1 mL) sequentially by syringe. Then, the reaction mixture was stirred at -20 °C for 16 h. After quenching by adding water, the NMR of the crude reaction mixture was recorded using trifluorotoluene as an internal standard. The product was isolated by flash chromatography with appropriate eluent.

(*R*,*E*)-(4-Methoxyphenyl)(1,1,1-trifluorodec-2-en-4-yl)sulfane (6a)

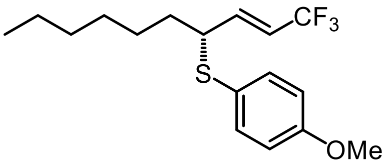

This compound was obtained according to the general procedure D using **2e** as reagent. Product **6a** was isolated in 75% yield (24.9 mg, γ/α = 27/1) as a colorless oil by silica gel chromatography using pentane/Et_2O (25/1) as eluent. **^{1}H NMR** (400 MHz, $CDCl_3$): δ = 7.36 – 7.29 (m, 2H), 6.89 – 6.79 (m, 2H), 6.19 (ddq, J = 15.6, 9.3, 2.1 Hz, 1H), 5.19 – 5.06 (m, 1H), 3.80 (s, 3H), 3.39 – 3.29 (m, 1H), 1.78 – 1.54 (m, 2H), 1.51 – 1.20 (m, 8H), 0.97 – 0.83 (m, 3H); **^{13}C NMR** (100 MHz, $CDCl_3$) δ = 160.3, 140.4 (q, J = 6.4 Hz), 137.3, 123.2, 122.9 (q, J = 269.5 Hz), 118.3 (q, J = 33.6 Hz), 114.6, 55.5, 51.6, 33.3, 31.7, 29.1, 27.3, 22.7, 14.2; **^{19}F NMR** (377 MHz, $CDCl_3$) δ = -63.55 – -64.04 (m, 3F) ppm. **HRMS (pos. APCI-ESI)** m/z: calcd for $C_{17}H_{24}F_3OS^+$ $[M+H]^+$ 333.1494, found 333.1474. **ORD** ($CHCl_3$, c 0.1, 24.8 °C): +3.

Determination of *ee* by Chiral SFC: Diacel CHIRALPAK ADH, 25 °C, 0.3 cm ϕ, 20 cm column, 5% MeOH in CO_2, flow rate: 0.8 mL/min; *t*R: 2.67 min (major enantiomer), 2.76 min (minor enantiomer); *ee* (major enantiomer) = 96%.

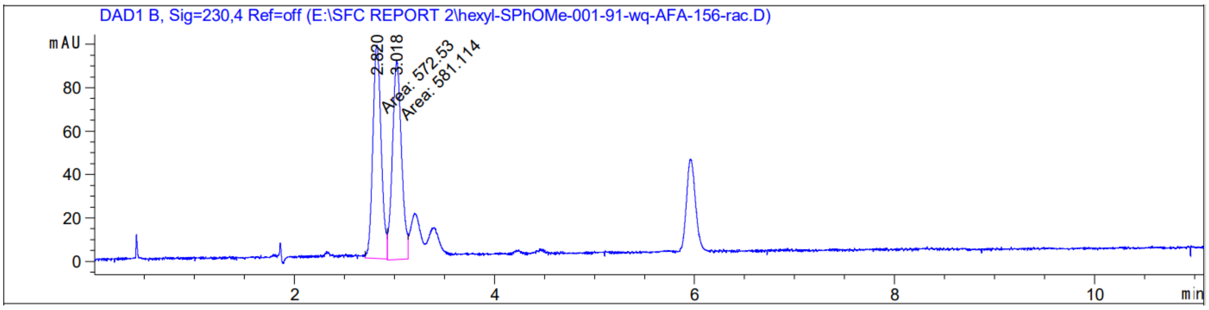

Signal 2: DAD1 B, Sig=230,4 Ref=off

Peak #	RetTime [min]	Type	Width [min]	Area [mAU*s]	Height [mAU]	Area %
1	2.820	MM T	0.0971	572.52954	98.28365	49.6280
2	3.018	MM T	0.1059	581.11377	91.47192	50.3720

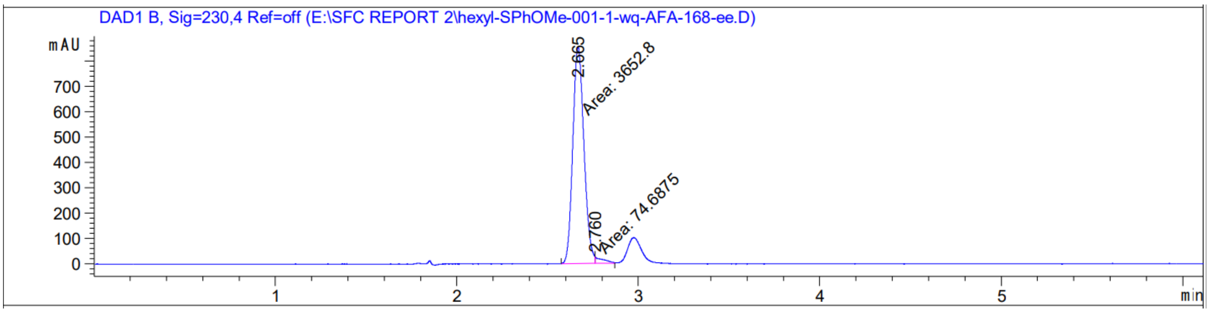

Signal 2: DAD1 B, Sig=230,4 Ref=off

Peak #	RetTime [min]	Type	Width [min]	Area [mAU*s]	Height [mAU]	Area %
1	2.665	MM T	0.0713	3652.80469	854.20355	97.9963
2	2.760	MM T	0.0499	74.68745	24.92593	2.0037

(*R*,*E*)-(4-Methoxyphenyl)(5,5,5-trifluoro-1-phenylpent-3-en-2-yl)sulfane (6b)

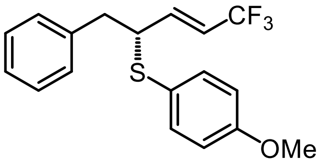

This compound was obtained according to the general procedure D using **2e** as reagent. Product **6b** was isolated in 54% yield (18.3 mg, γ/α = 6/1) as a colorless oil by silica gel chromatography using pentane/Et_2O (25/1) as eluent. **^{1}H NMR** (400 MHz, $CDCl_3$): *δ* = 7.37 – 7.21 (m, 5H), 7.16 (d, *J* = 6.7 Hz, 2H), 6.84 (d, *J* = 8.8 Hz, 2H), 6.24 (ddq, *J* = 15.6, 9.3, 2.1 Hz, 1H), 5.07 (dqd, J = 15.7, 7.2, 0.8 Hz, 1H), 3.80 (s, 3H), 3.62 (dddd, J = 9.5, 8.4, 6.1, 1.1 Hz, 1H), 3.05 (dd, J = 13.9, 6.2 Hz, 1H), 2.90 (dd, J = 13.9, 8.4 Hz, 1H); **^{13}C NMR** (100 MHz, $CDCl_3$) *δ* = 160.4, 139.5 (q, *J* = 6.5 Hz), 137.8, 137.2, 129.3, 128.6, 127.0, 123.0, 122.7 (q, *J* = 269.7 Hz), 119.0 (q, *J* = 33.7 Hz), 114.7, 55.5, 52.8, 40.1; **^{19}F NMR** (377 MHz, $CDCl_3$) *δ* = -63.50 – -64.14 (m, 3F) ppm. **HRMS (pos. APCI-ESI)** m/z: calcd for $C_{18}H_{18}F_3OS^+$ $[M+H]^+$ 339.1025, found 339.1026. **ORD** ($CHCl_3$, c 0.1, 26.1 ºC): -7.

Determination of *ee* by Chiral SFC: Diacel CHIRALPAK OJ-H, 25 °C, 0.3 cm *ϕ*, 15 cm column, 1% ^{i}PrOH in CO_2, flow rate: 1.2 mL/min; *t*R: 3.25 min (minor enantiomer), 4.77 min (major enantiomer); *ee* (major enantiomer) = 96%.

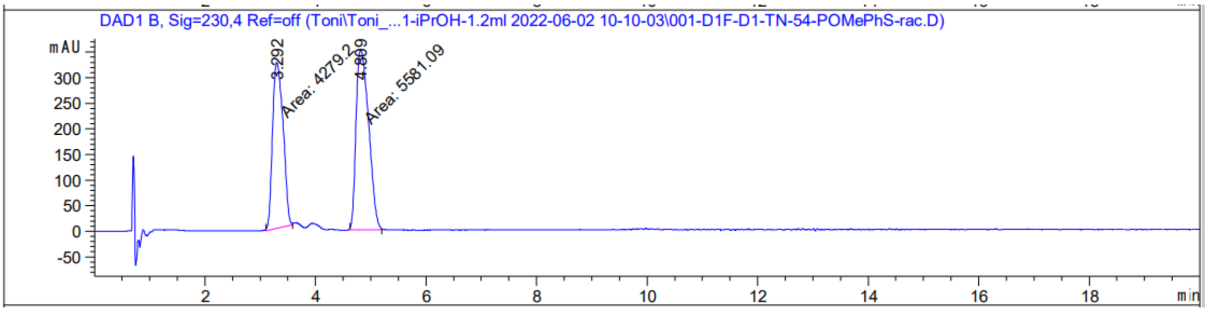

Signal 2: DAD1 B, Sig=230,4 Ref=off

Peak #	RetTime [min]	Type	Width [min]	Area [mAU*s]	Height [mAU]	Area %
1	3.292	MM	0.2203	4279.19824	323.67004	43.3983
2	4.809	MM	0.2637	5581.08691	352.74374	56.6017

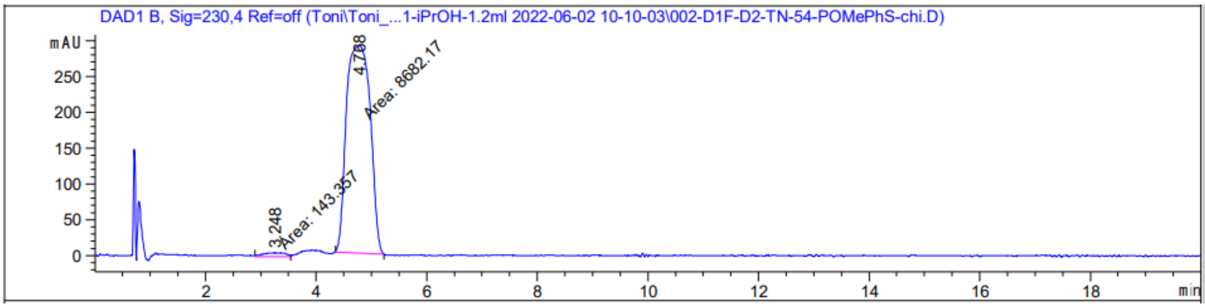

Signal 2: DAD1 B, Sig=230,4 Ref=off

Peak #	RetTime [min]	Type	Width [min]	Area [mAU*s]	Height [mAU]	Area %
1	3.248	MM T	0.4541	143.35698	5.26215	1.6243
2	4.768	MM	0.4990	8682.17090	289.99496	98.3757

(*R*,*E*)-(8-Chloro-1,1,1-trifluorooct-2-en-4-yl)(4-methoxyphenyl)sulfane (6c)

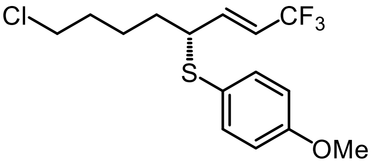

This compound was obtained according to the general procedure D using **2e** as reagent. Product **6c** was isolated in 59% yield (19.9 mg, γ/α = 12/1) as a colorless oil by silica gel chromatography using pentane/Et_2O (25/1) as eluent. **^{1}H NMR** (400 MHz, $CDCl_3$): δ = 7.39 – 7.28 (m, 2H), 6.92 – 6.78 (m, 2H), 6.19 (ddq, J = 15.8, 9.3, 2.2 Hz, 1H), 5.24 – 5.07 (m, 1H), 3.80 (s, 3H), 3.54 (t, J = 6.5 Hz, 2H), 3.34 (q, J = 7.5 Hz, 1H), 1.85 – 1.56 (m, 6H); **^{13}C NMR** (100 MHz, $CDCl_3$) δ = 160.4, 139.9 (q, J = 6.6 Hz), 137.4, 122.78, 122.76 (q, J = 269.6 Hz), 118.6 (q, J = 33.6 Hz), 114.7, 55.5, 51.3, 44.7, 32.5, 32.3, 24.7; **^{19}F NMR** (377 MHz, $CDCl_3$) δ = -63.78 – -63.84 (m, 3F) ppm. **HRMS (pos. ESI)** m/z: calcd for $C_{15}H_{18}ClF_3NaOS^+$ $[M+Na]^+$ 361.0611, found 361.0600. **ORD** ($CHCl_3$, c 0.1, 26.1 °C): +4.

Determination of *ee* by Chiral SFC: Diacel CHIRALPAK IB, 25 °C, 0.3 cm ϕ, 20 cm column, 4% MeOH in CO_2, flow rate: 0.8 mL/min; *t*R: 5.22 min (minor enantiomer), 5.48 min (major enantiomer); *ee* (major enantiomer) = 88%.

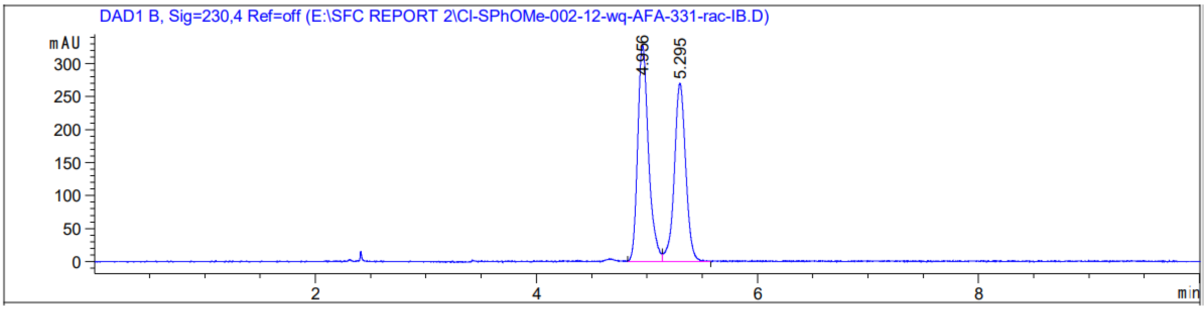

Signal 2: DAD1 B, Sig=230,4 Ref=off

Peak #	RetTime [min]	Type	Width [min]	Area [mAU*s]	Height [mAU]	Area %
1	4.956	VV R	0.1006	2240.93164	328.50235	53.5984
2	5.295	VV R	0.0912	1940.03967	270.43591	46.4016

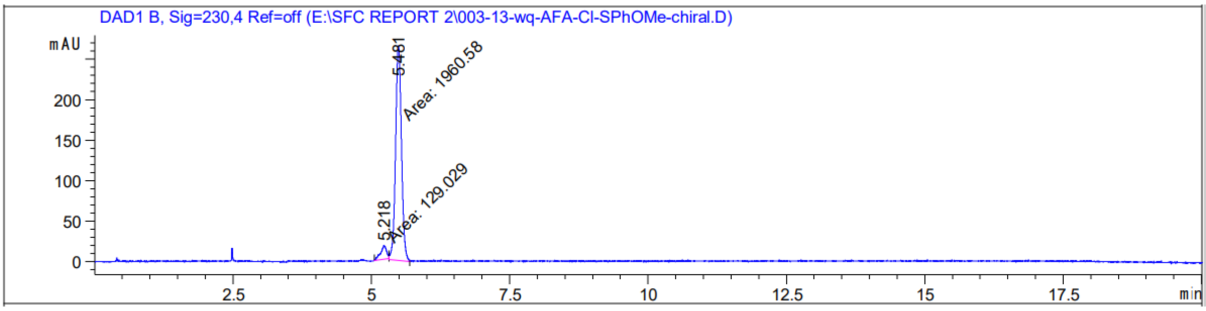

Signal 2: DAD1 B, Sig=230,4 Ref=off

Peak #	RetTime [min]	Type	Width [min]	Area [mAU*s]	Height [mAU]	Area %
1	5.218	MM T	0.1268	129.02856	16.95471	6.1748
2	5.481	MM T	0.1234	1960.57690	264.90036	93.8252

(S,*E*)-2-(5,5,5-Trifluoro-2-(phenylthio)pent-3-en-1-yl)isoindoline-1,3-dione (6d)

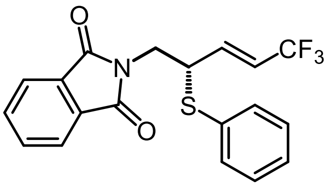

This compound was obtained according to the general procedure D using **2f** as reagent. Product **6d** was isolated in 65% yield (24.5 mg, γ/α = 3.5/1) as a white solid by silica gel chromatography using pentane/EtOAc (6/1) as eluent. **^{1}H NMR** (400 MHz, $CDCl_3$): δ = 7.89 – 7.80 (m, 2H), 7.77 – 7.68 (m, 2H), 7.42 (dd, J = 7.2, 2.2 Hz, 2H), 7.35 – 7.21 (m, 3H), 6.30 (ddq, J = 16.1, 9.0, 2.3 Hz, 1H), 5.42 (dq, J = 15.8, 6.2 Hz, 1H), 4.13 (p, J = 7.8 Hz, 1H), 4.02 (dd, J = 13.9, 7.2 Hz, 1H), 3.92 (dd, J = 13.9, 8.3 Hz, 1H); **^{13}C NMR** (100 MHz, $CDCl_3$) δ = 168.1, 136.8 (q, J = 6.5 Hz), 134.4, 134.3, 131.8, 131.5, 129.3, 128.7, 123.7, 122.34 (q, J = 269.9 Hz), 120.9 (q, J = 34.1 Hz), 48.2, 40.6; **^{19}F NMR** (377 MHz, $CDCl_3$) δ = -64.35 – -64.42 (m, 3F) ppm. **HRMS (pos. ESI)** m/z: calcd for $C_{19}H_{14}F_3NNaO_2S^+$ $[M+Na]^+$ 400.0590, found 400.0585. **ORD** ($CHCl_3$, c 0.1, 25.7 ºC): +27.

Determination of *ee* by Chiral SFC: Diacel CHIRALPAK IA, 25 °C, 0.3 cm ϕ, 15 cm column, 3% MeOH in CO_2, flow rate: 0.8 mL/min; *t*R: 6.00 min (major enantiomer), 6.75 min (minor enantiomer); *ee* (major enantiomer) = 98%.

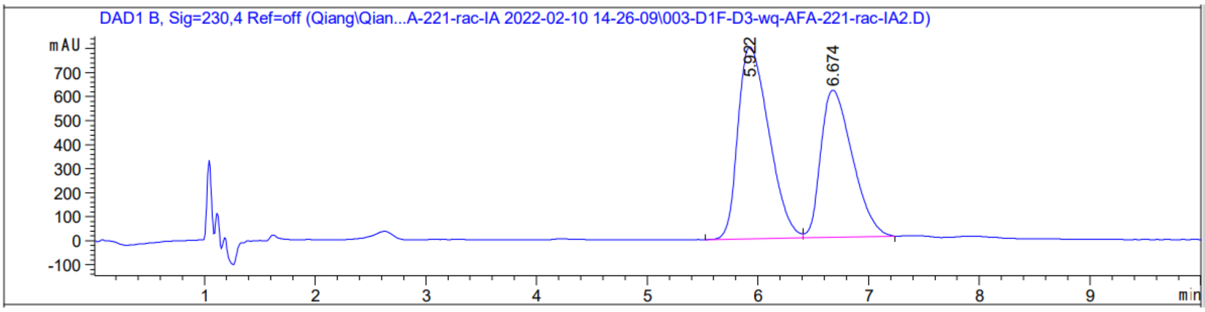

Signal 2: DAD1 B, Sig=230,4 Ref=off

Peak #	RetTime [min]	Type	Width [min]	Area [mAU*s]	Height [mAU]	Area %
1	5.922	BV	0.3102	1.54928e4	798.95972	56.0725
2	6.674	VB	0.3132	1.21372e4	612.41248	43.9275

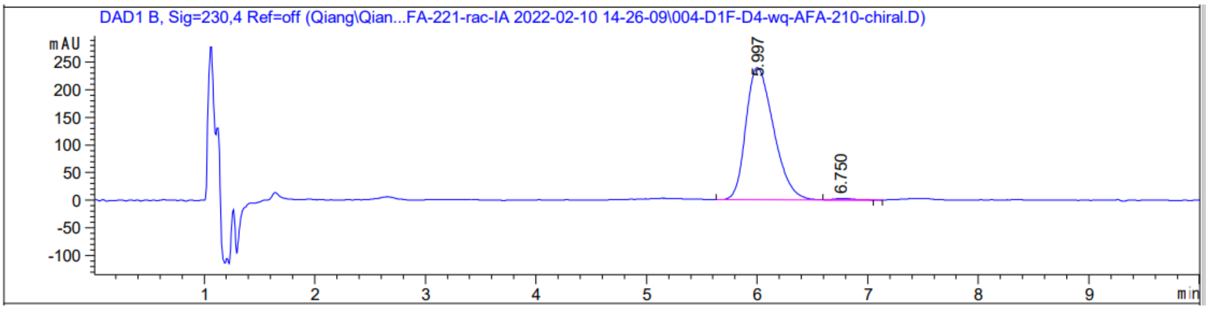

Signal 2: DAD1 B, Sig=230,4 Ref=off

Peak #	RetTime [min]	Type	Width [min]	Area [mAU*s]	Height [mAU]	Area %
1	5.997	BV R	0.2754	4177.37354	239.54993	99.1857
2	6.750	VV E	0.1745	34.29462	2.37437	0.8143

General procedure E for the halogenation

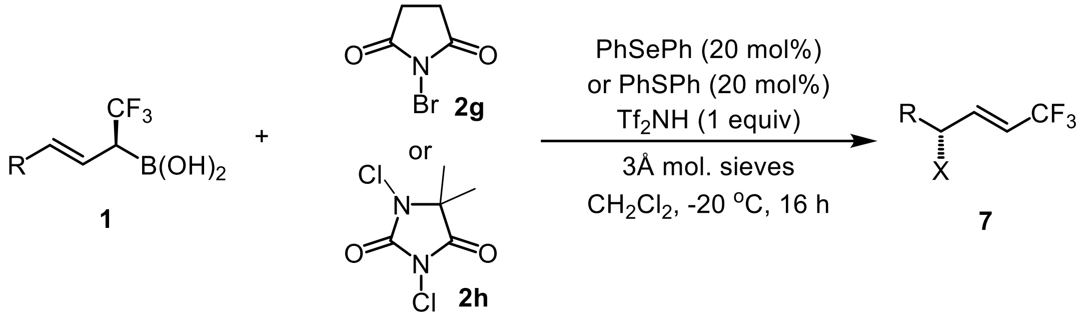

Scheme S6. Synthesis of allylic halides 7a-7c.

An oven-dried microwave vial was charged with 1-bromopyrrolidine-2,5-dione **2g** (0.15 mmol) or 1,3-dichloro-5,5-dimethylimidazolidine-2,4-dione **2h** (0.15 mmol). The reaction vial was brought into the glovebox and triflimide (0.1 mmol) and 3Å molecular sieves (20 mg) were added. The reaction vial was tightly sealed with rubber septum and transferred to a -20 °C cooling bath. To the reaction mixture was added selenide catalyst (0.02 mmol) or sulfide catalyst (0.02 mmol) and the stock solution of allylboronic acid **1** (0.1 mmol, 1 mL) sequentially by syringe. Then, the reaction mixture was stirred at -20 °C for 16 h. After quenching by adding water, the NMR of the crude reaction mixture was recorded using trifluorotoluene as an internal standard. The product was isolated by flash chromatography with appropriate eluent.

(*R*,*E*)-4-Bromo-1,1,1-trifluorodec-2-ene (7a)

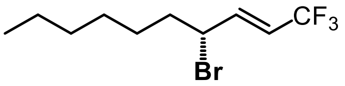

This compound was obtained according to the general procedure E using **2g** as reagent and PhSePh as catalyst. Product **7a** was isolated in 75% yield (20.4 mg, γ/α = 33/1) as a colorless oil by silica gel chromatography using pentane as eluent. **^{1}H NMR** (400 MHz, $CDCl_3$): δ = 6.46 (ddq, J = 15.4, 8.9, 2.1 Hz, 1H), 5.79 (dq, J = 15.6, 6.3 Hz, 1H), 4.46 (q, J = 7.7 Hz, 1H), 2.03 – 1.81 (m, 2H), 1.51 – 1.22 (m, 8H), 0.97 – 0.76 (m, 3H); **^{13}C NMR** (100 MHz, $CDCl_3$) δ = 140.1 (q, J = 6.5 Hz), 122.6 (q, J = 269.8 Hz), 119.4 (q, J = 34.2 Hz), 50.5, 38.1, 31.7, 28.6, 27.5, 22.7, 14.2; **^{19}F NMR** (377 MHz, $CDCl_3$) δ = -64.28 – -64.33 (m, 3F) ppm. **EI-MS (m/z, relative intensity)** 193 ($[M-Br]^+$, 1.3), 191 ($[M-Br]^+$, 0.54), 151 (80), 131 (34), 122 (18), 57 (52), 55 (38), 43 (100). **ORD** ($CHCl_3$, c 0.1, 25.7 °C): -1.8.

Determination of *ee* by Chiral GC: Column Chiraldex β-DM, 50 °C, 60 min; *t*R: 61.10 min (major enantiomer), 61.97 min (minor enantiomer); *ee* (major enantiomer) = 96%.

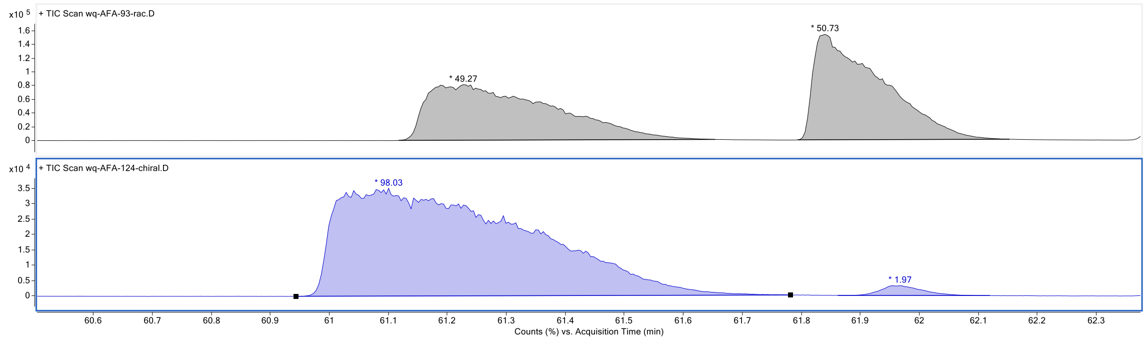

Area	Height	Type	Peak	RT	Area %	Area Sum %	Saturated	Width	FWHM	SNR	RI
725886.81	147683.47	M	2	61.967	2.01	1.97		0.258	0.077		
36132218.24	1606675.11	M	1	61.101	100	98.03		0.837	0.392		

(*S*,*E*)-2-(2-Bromo-5,5,5-trifluoropent-3-en-1-yl)isoindoline-1,3-dione (7b)

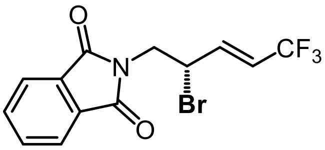

This compound was obtained according to the general procedure E using **2g** as reagent and PhSePh as catalyst. Product **7b** was isolated in 74% yield (25.7 mg, γ/α = 26/1) as a white solid by silica gel chromatography using pentane/EtOAc (7/1) as eluent. **^{1}H NMR** (400 MHz, $CDCl_3$): δ = 7.96 – 7.85 (m, 2H), 7.83 – 7.71 (m, 2H), 6.48 (ddq, J = 15.2, 9.2, 1.6 Hz, 1H), 5.86 (dq, J = 15.2, 6.0 Hz, 1H), 4.93 – 4.79 (m, 1H), 4.24 – 4.03 (m, 2H); **^{13}C NMR** (100 MHz, $CDCl_3$) δ = 167.8, 136.9 (q, J = 6.6 Hz), 134.6, 131.7, 123.9, 122.2 (q, J = 34.7 Hz), 122.1 (q, J = 270.3 Hz), 44.7, 43.2; **^{19}F NMR** (377 MHz, $CDCl_3$) δ = -64.28 – -64.33 (m, 3F) ppm. **HRMS (pos. ESI)** m/z: calcd for $C_{13}H_9BrF_3NNaO_2^+$ $[M+Na]^+$ 369.9661, found 369.9666 and 371.9722. **ORD** ($CHCl_3$, c 0.1, 26.4 ºC): -39.

Determination of *ee* by Chiral SFC: Diacel CHIRALPAK IF, 25 °C, 0.3 cm ϕ, 15 cm column, 3% MeOH in CO_2, flow rate: 0.8 mL/min; *t*R: 4.16 min (minor enantiomer), 5.20 min (major enantiomer); *ee* (major enantiomer) = 88.5%.

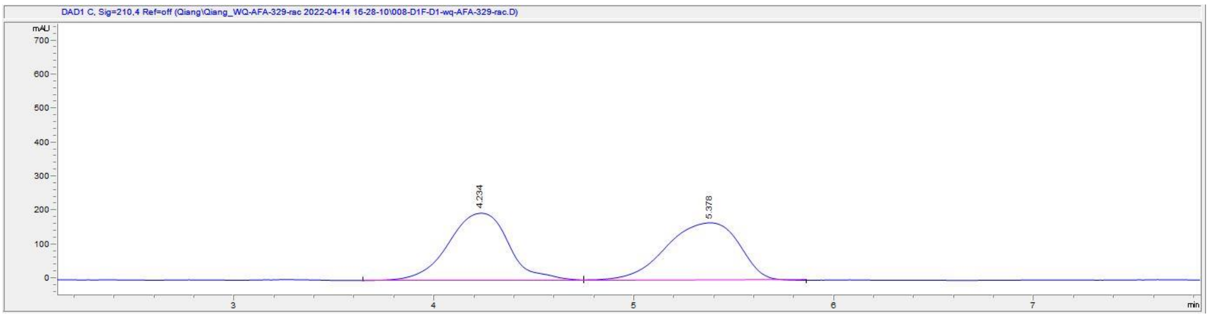

#	Time	Type	Area	Height	Width	Area%	Symmetry
1	4.234	BV	4202.6	198.9	0.3366	49.289	1.131
2	5.378	VB	4323.8	169.9	0.4129	50.711	1.458

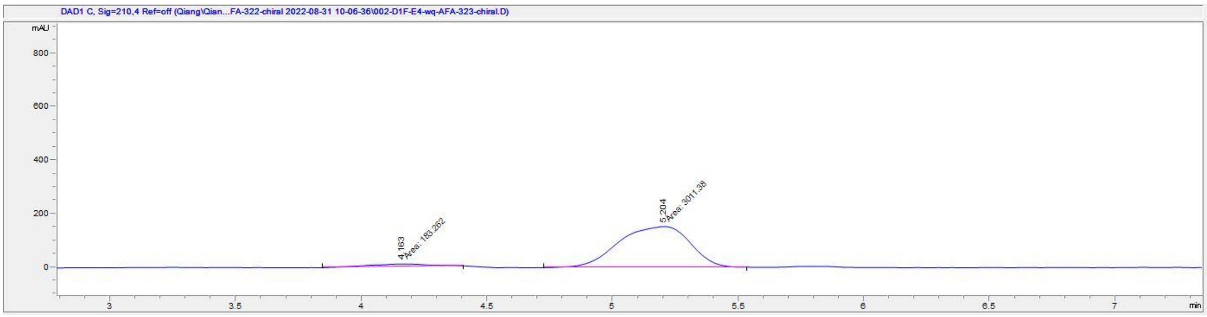

#	Time	Type	Area	Height	Width	Area%	Symmetry
1	4.163	MM T	183.3	10.3	0.297	5.737	1.312
2	5.204	MM T	3011.4	153.1	0.3279	94.263	1.642

(S,E)-2-(2-chloro-5,5,5-trifluoropent-3-en-1-yl)isoindoline-1,3-dione (7c)

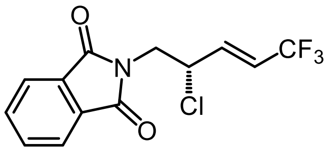

This compound was obtained according to the general procedure E using **2h** as reagent and PhSPh as catalyst. Product **7c** was isolated in 55% yield (16.7 mg) as a white solid by silica gel chromatography using pentane/EtOAc (7/1) as eluent. **^{1}H NMR** (400 MHz, $CDCl_3$): δ = 7.91 – 7.82 (m, 2H), 7.81 – 7.70 (m, 2H), 6.45 (ddq, J = 15.5, 7.6, 2.0 Hz, 1H), 5.97 (dqd, J = 15.5, 6.1, 1.2 Hz, 1H), 4.93 – 4.76 (m, 1H), 4.15 – 3.91 (m, 2H).; **^{13}C NMR** (100 MHz, $CDCl_3$) δ = 167.8, 136.2 (q, J = 6.5 Hz), 134.6, 131.7, 123.9, 122.6 (q, J = 34.5 Hz), 122.2 (q, J = 270.0 Hz), 55.2, 43.1; **^{19}F NMR** (377 MHz, $CDCl_3$) δ = -64.28 – -64.33 (m, 3F) ppm. **HRMS (pos. ESI)** m/z: calcd for $C_{13}H_9ClF_3NNaO_2^+$ [M+Na]$^+$ 326.0166, found 326.0164. **ORD** ($CHCl_3$, c 0.1, 26.7 ºC): -40.

Determination of *ee* by Chiral SFC: Diacel CHIRALPAK IF, 25 °C, 0.3 cm ϕ, 15 cm column, 0.5% MeOH in CO_2, flow rate: 1.2 mL/min; *t*R: 8.11 min (minor enantiomer), 12.21 min (major enantiomer); *ee* (major enantiomer) = 91%.

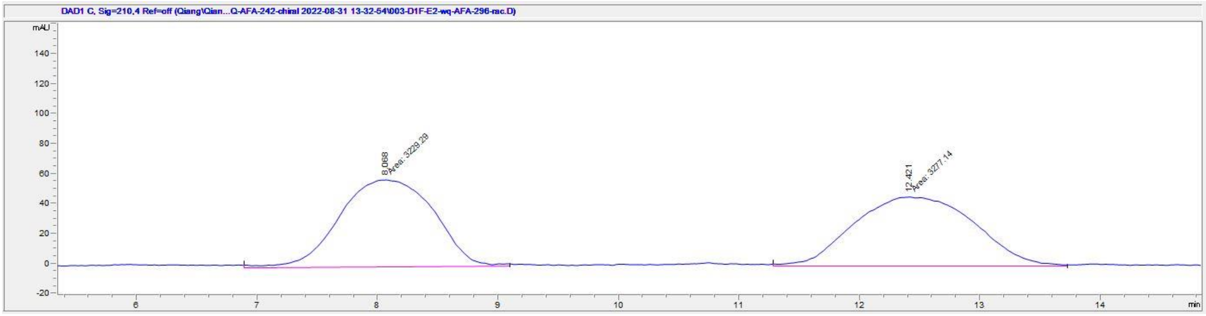

#	Time	Type	Area	Height	Width	Area%	Symmetry
1	8.068	MM T	3229.3	58.5	0.9208	49.632	2.27E-4
2	12.421	MM T	3277.1	46.5	1.1742	50.368	0

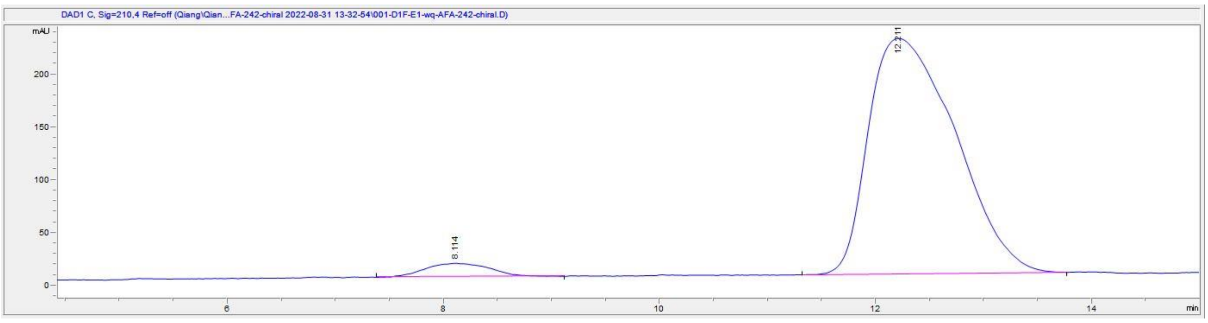

#	Time	Type	Area	Height	Width	Area%	Symmetry
1	8.114	BB	552.4	12.9	0.5097	4.253	0.889
2	12.211	BV	12435.9	222.6	0.7759	95.747	0.509

^{1}H NMR, ^{13}C NMR, ^{19}F NMR and ^{11}B NMR spectra of substrates and products

Diethyl (*E*)-oct-1-en-1-ylboronate (^{1}H NMR)

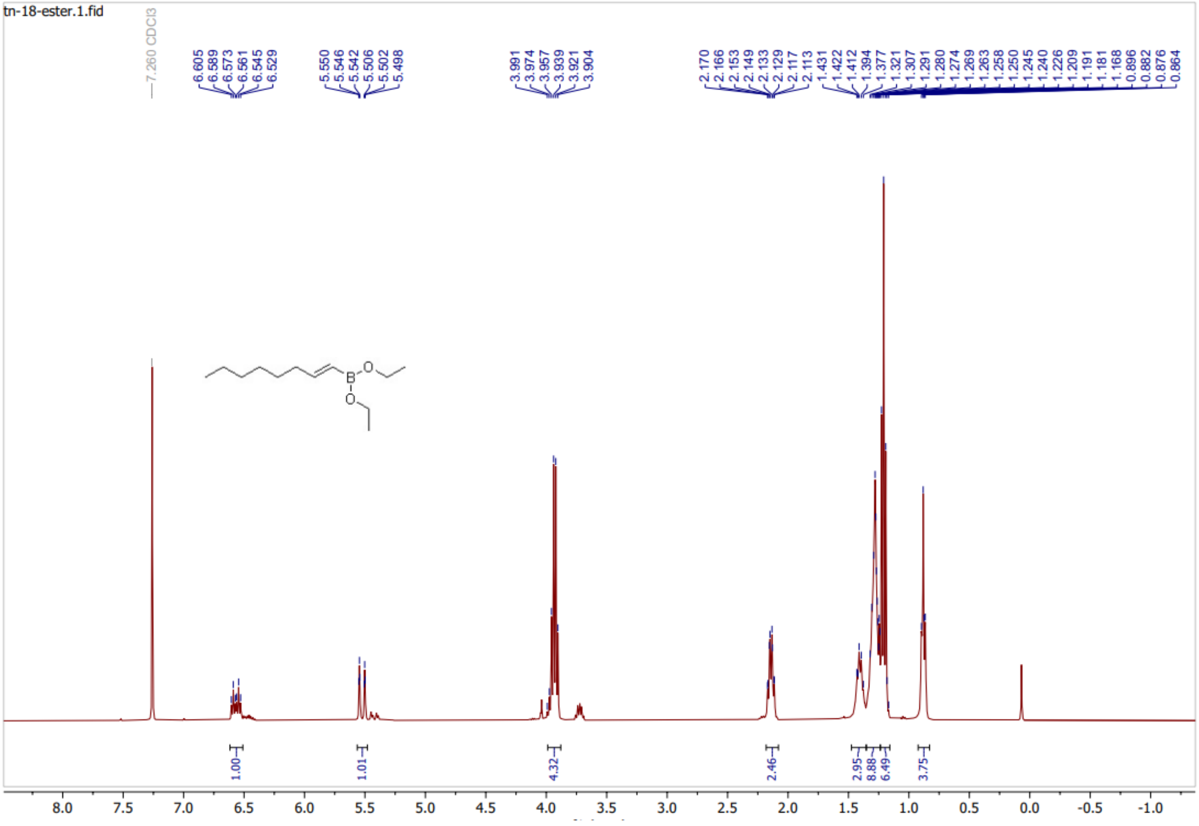

(*S*,*E*)-2-(1,1,1-Trifluorodec-3-en-2-yl)-2,3-dihydro-1*H*-naphtho[1,8*de*][1,3,2]diazaborinine (^{1}H NMR)

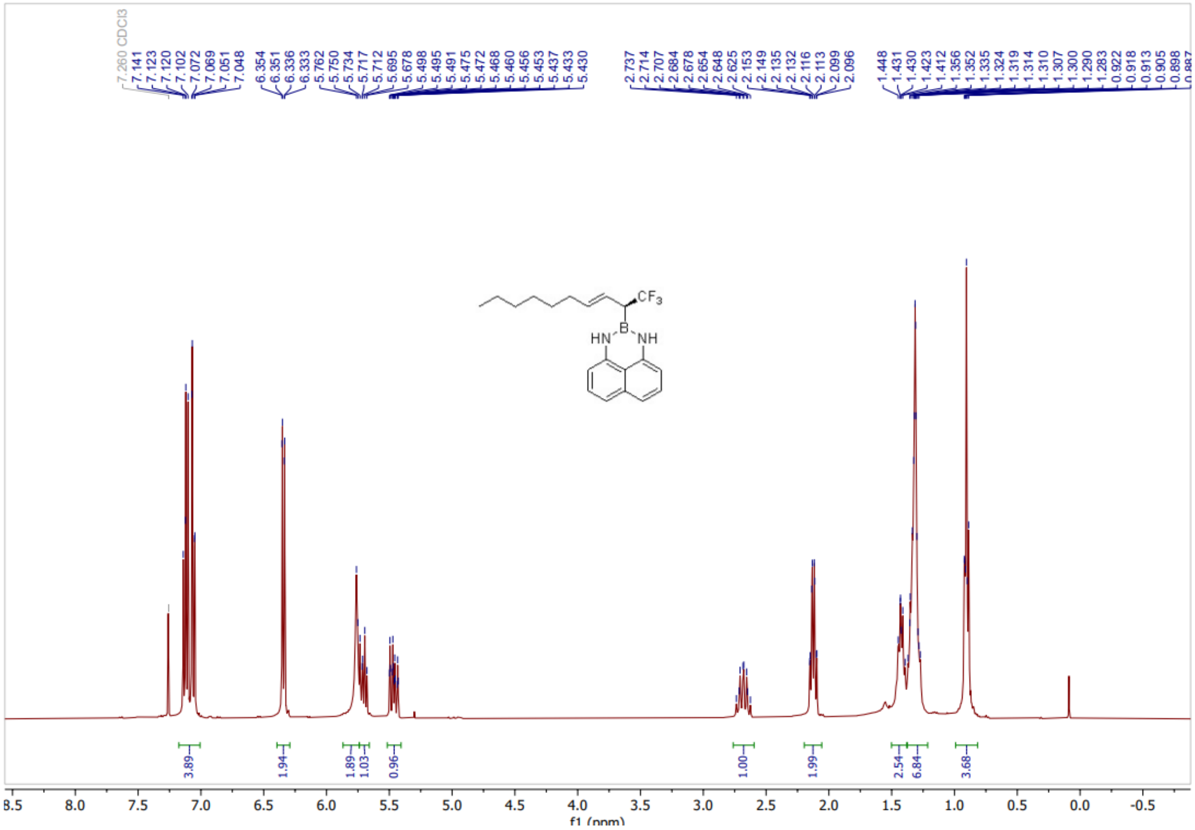

(*S*,*E*)-(1,1,1-Trifluorodec-3-en-2-yl)boronic acid (**1a**) (**[1]**H NMR)

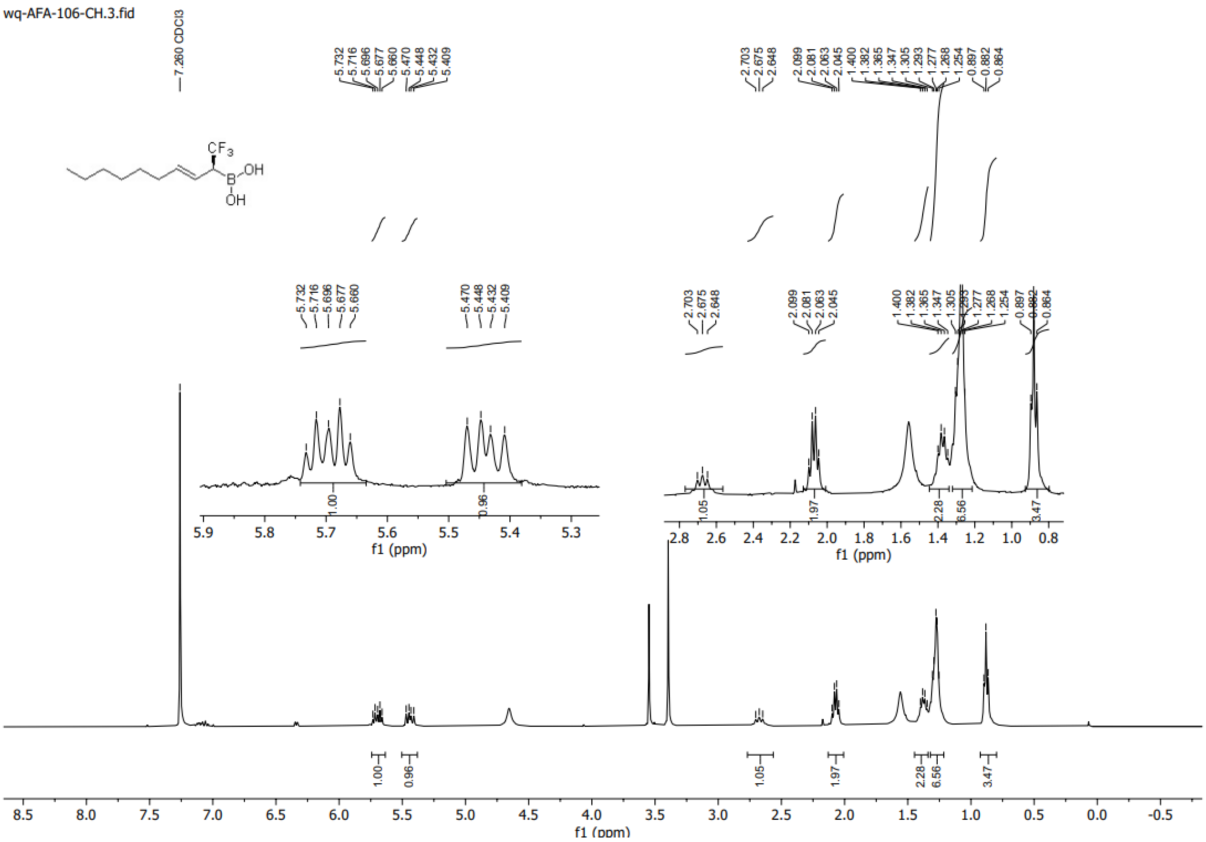

(*S*,*E*)-(1,1,1-Trifluorodec-3-en-2-yl)boronic acid (**1a**) (**[19]**F NMR)

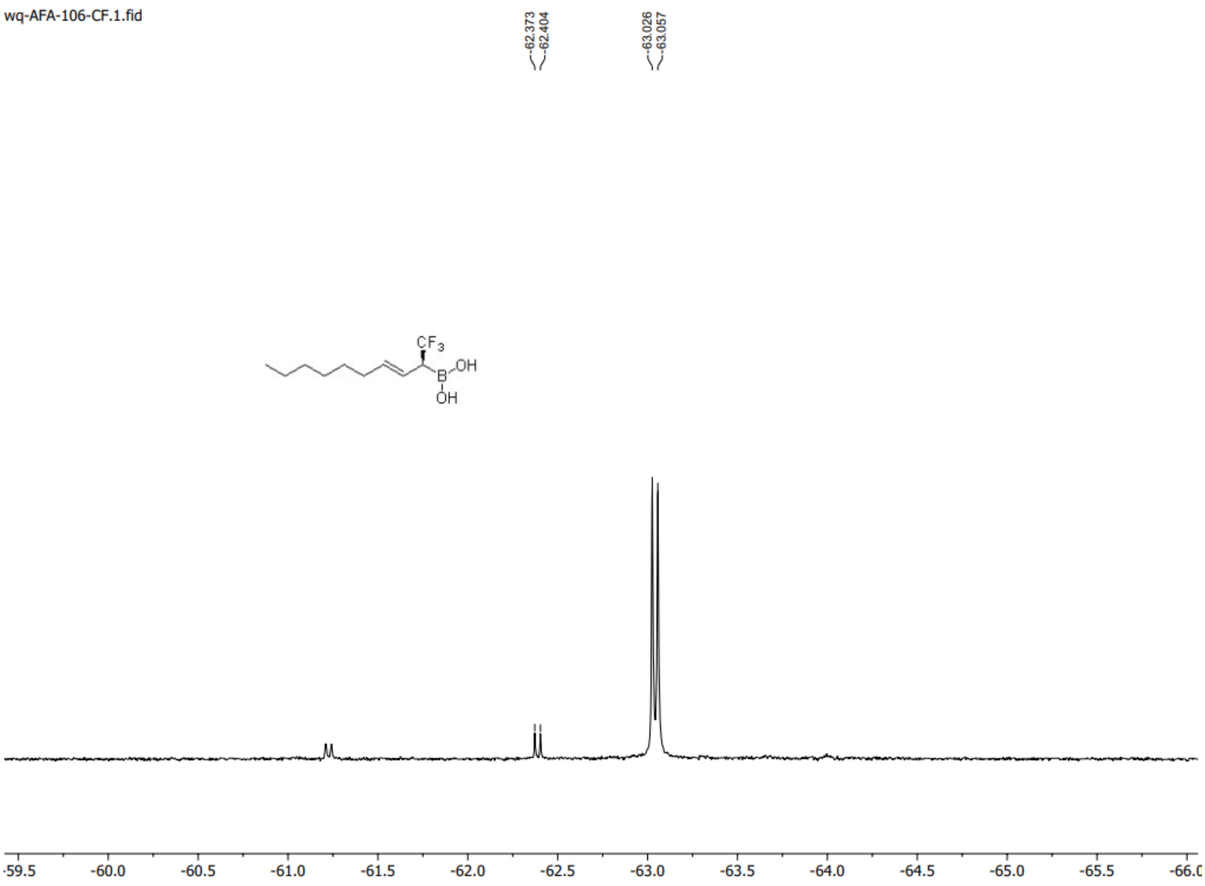

Diethyl (*E*)-oct-1-en-1-ylboronate (^{1}H NMR)

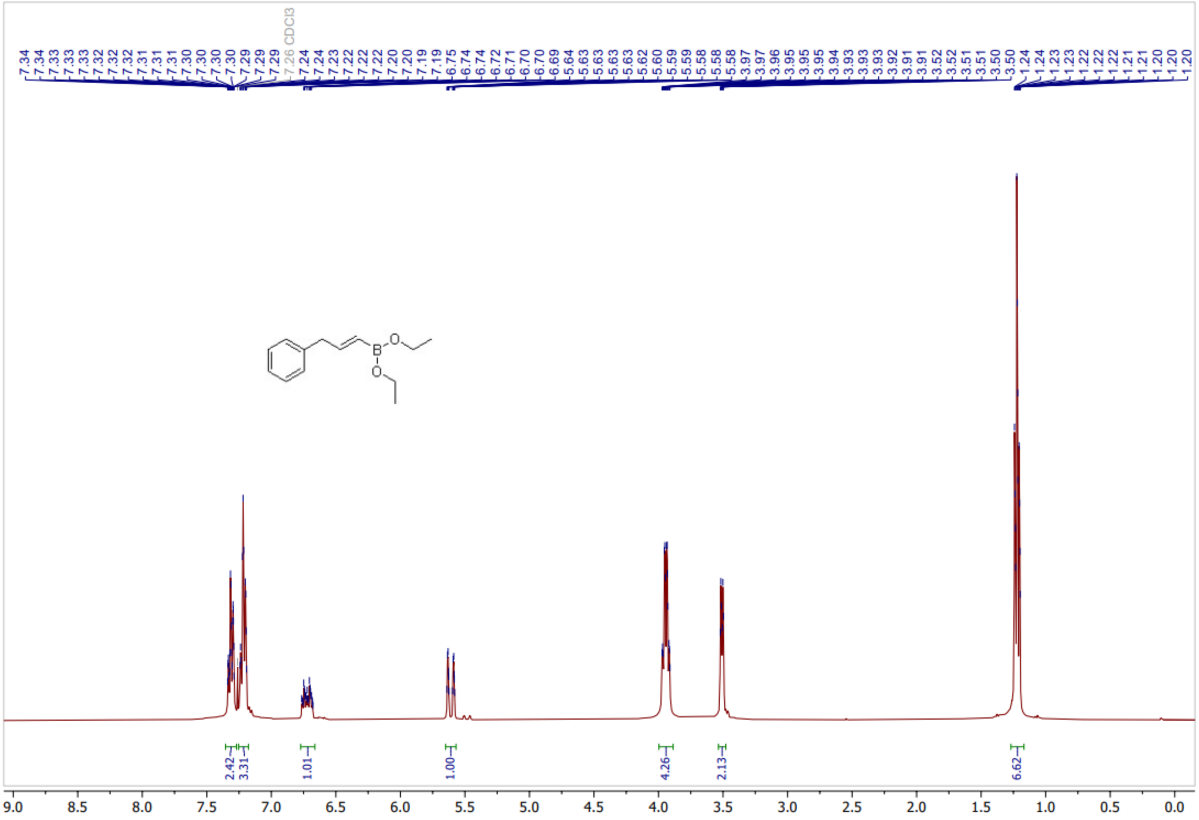

(*S*,*E*)-2-(1,1,1-Trifluoro-5-phenylpent-3-en-2-yl)-2,3-dihydro-1*H*-naphtho[1,8 *de*][1,3,2]diazaborinine (^{1}H NMR)

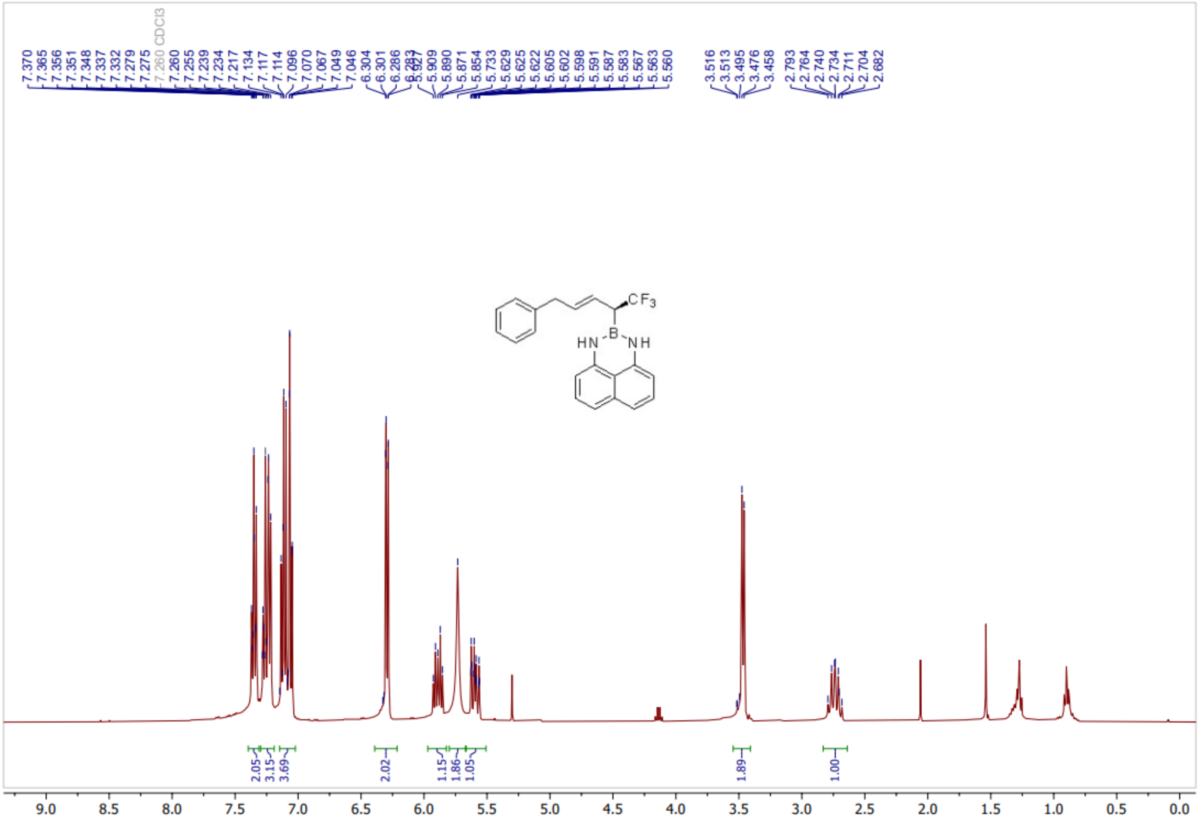

(*S*,*E*)-(1,1,1-Trifluoro-5-phenylpent-3-en-2-yl)boronic acid (**1b**) (^{1}H NMR)

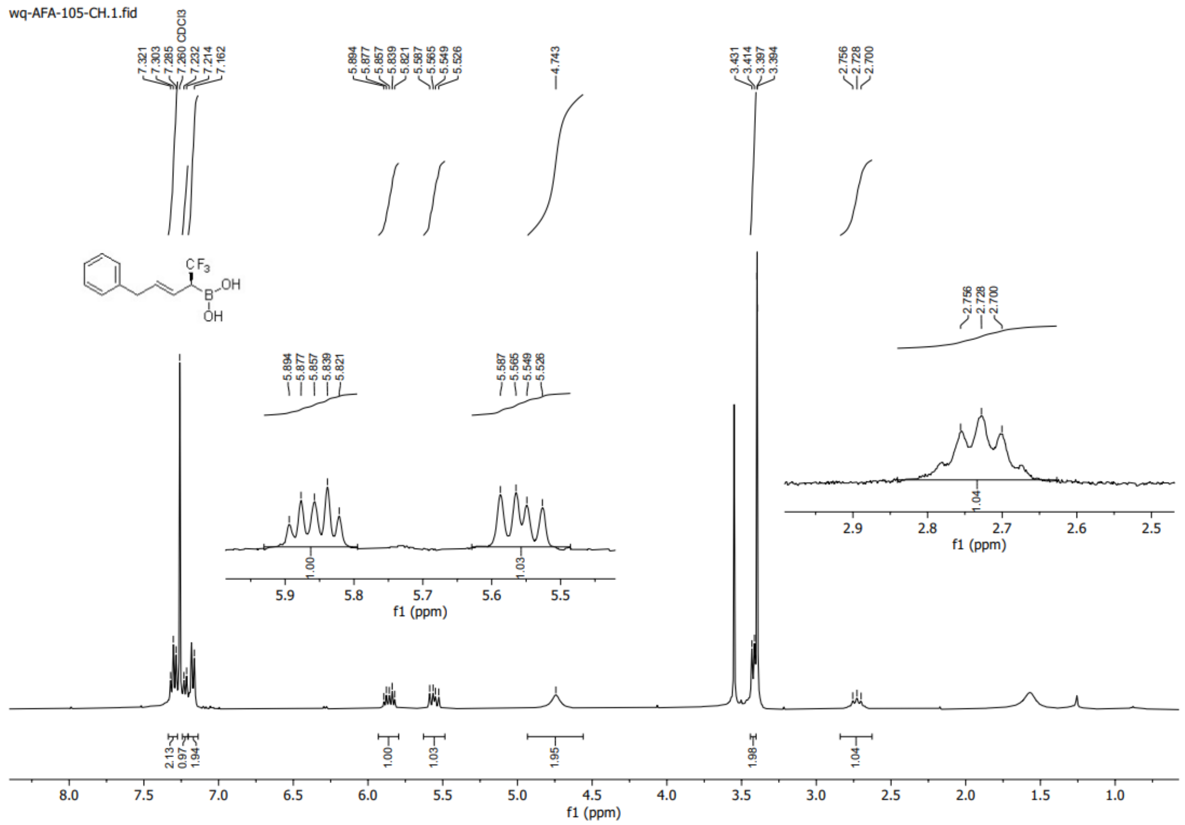

(*S*,*E*)-(1,1,1-Trifluoro-5-phenylpent-3-en-2-yl)boronic acid (**1b**) (^{19}F NMR)

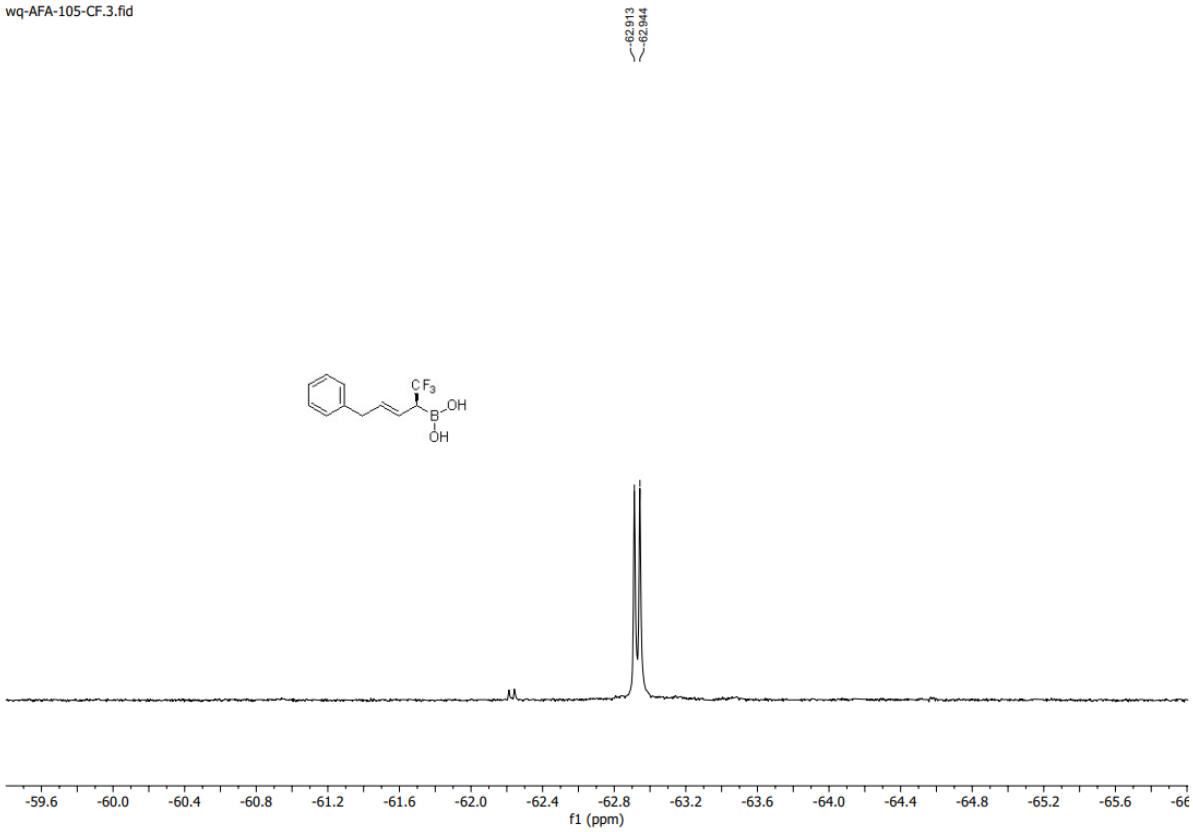

Diethyl (*E*)-(6-chlorohex-1-en-1-yl)boronate (^{1}H NMR)

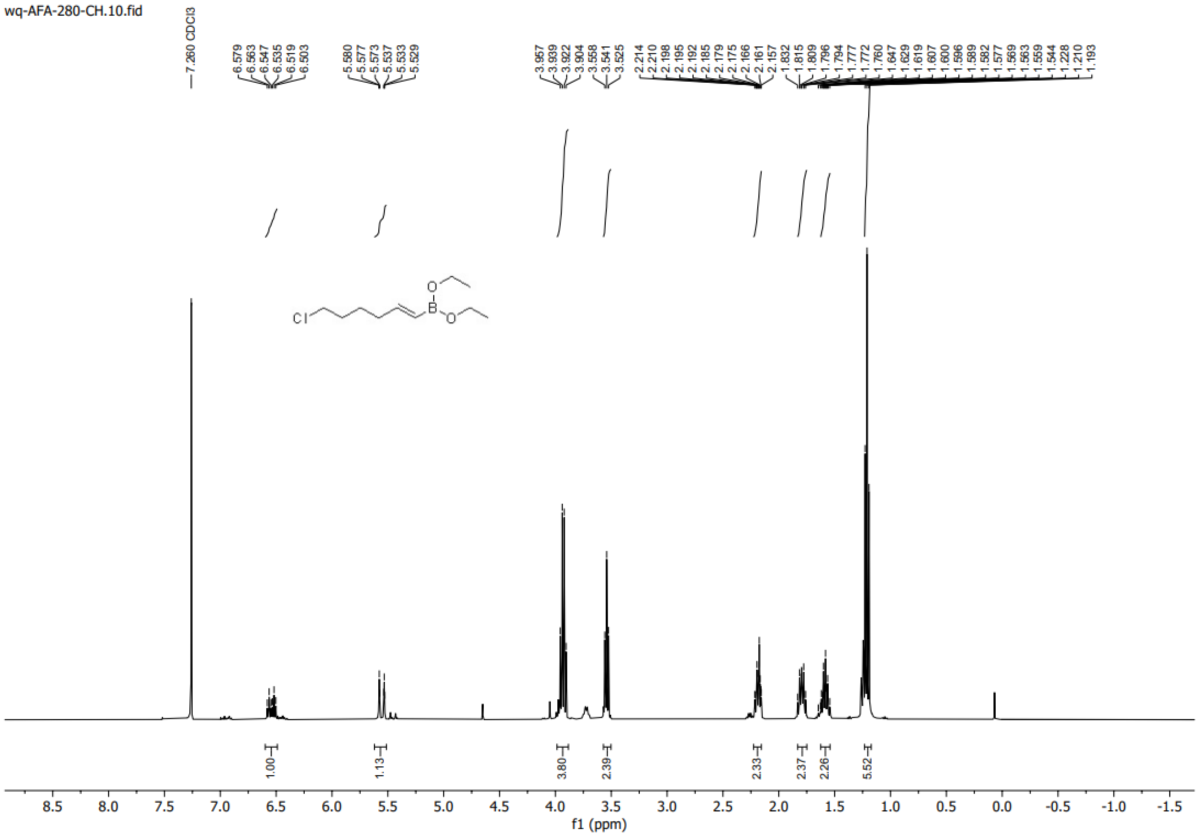

(*S*,*E*)-2-(8-Chloro-1,1,1-trifluorooct-3-en-2-yl)-2,3-dihydro-1*H*-naphtho[1,8-*de*][1,3,2]diazaborinine (^{1}H NMR)

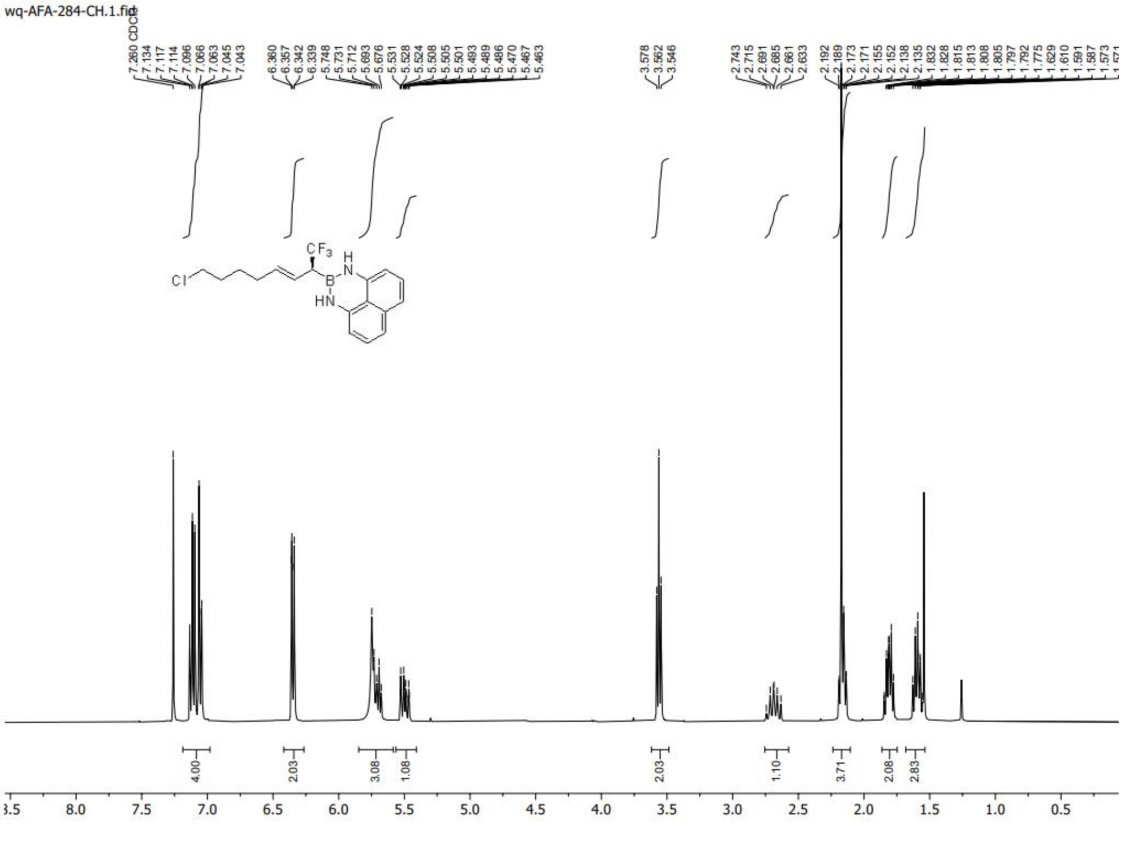

(*S*,*E*)-2-(8-Chloro-1,1,1-trifluorooct-3-en-2-yl)-2,3-dihydro-1*H*-naphtho[1,8-*de*][1,3,2]diazaborinine (^{13}C NMR)

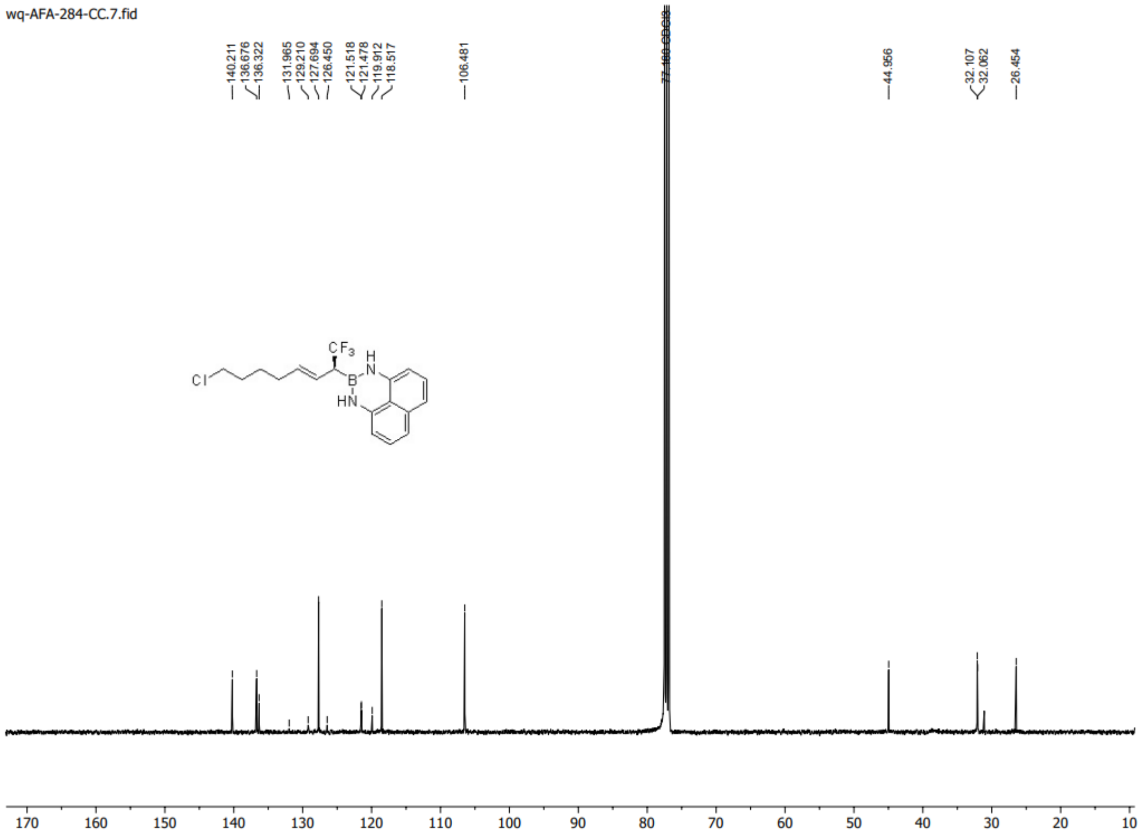

(*S*,*E*)-2-(8-Chloro-1,1,1-trifluorooct-3-en-2-yl)-2,3-dihydro-1*H*-naphtho[1,8-*de*][1,3,2]diazaborinine (^{19}F NMR)

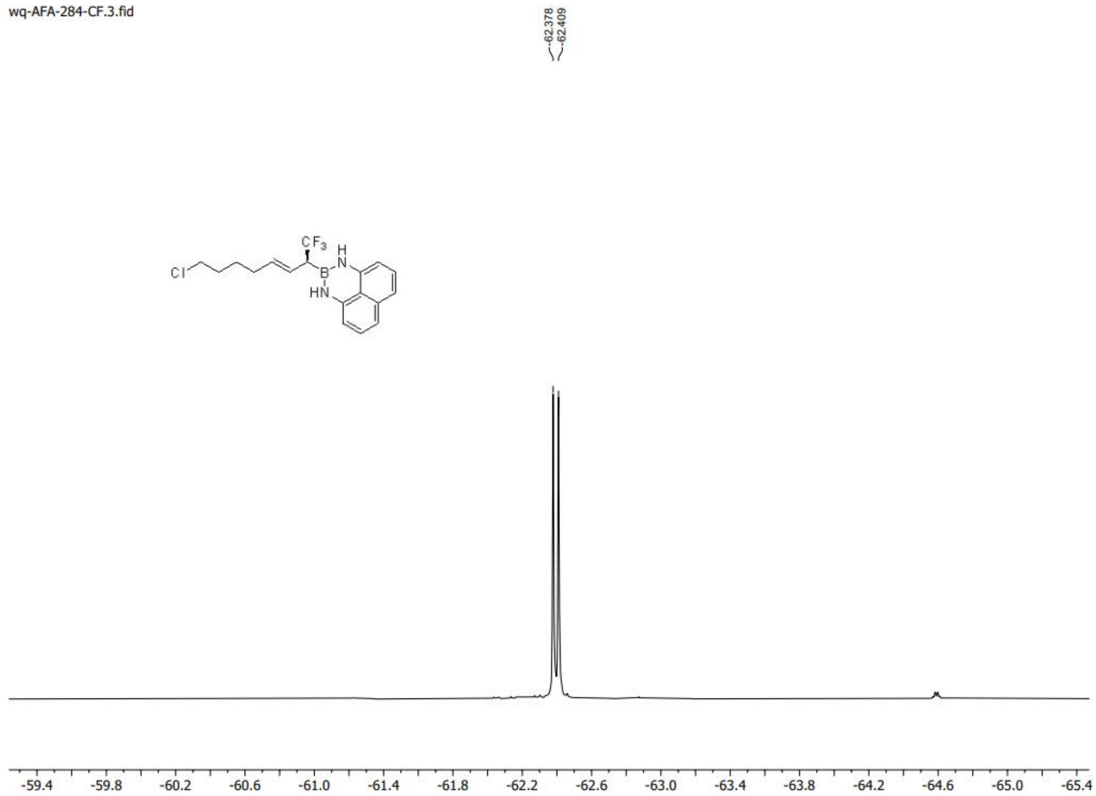

(*S*,*E*)-2-(8-Chloro-1,1,1-trifluorooct-3-en-2-yl)-2,3-dihydro-1*H*-naphtho[1,8-*de*][1,3,2]diazaborinine (^{11}B NMR)

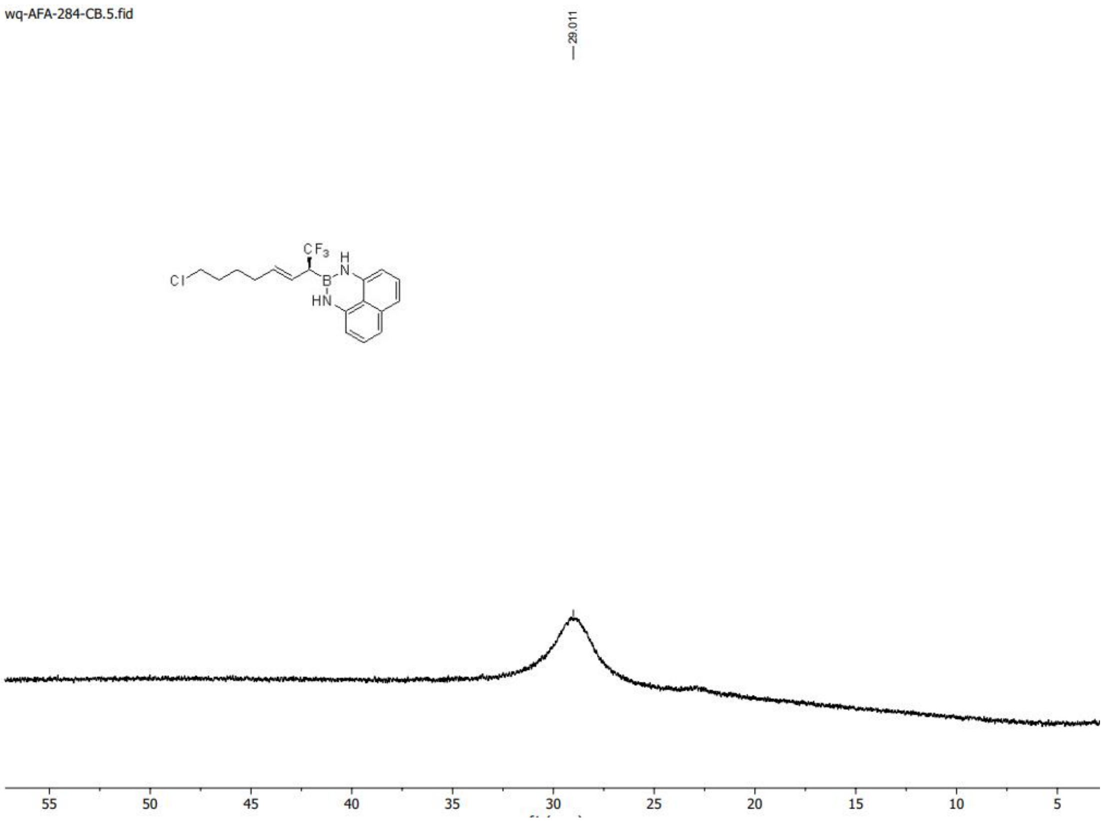

(*S*,*E*)-(1,1,1-Trifluoro-5-phenylpent-3-en-2-yl)boronic acid (**1c**) (^{1}H NMR)

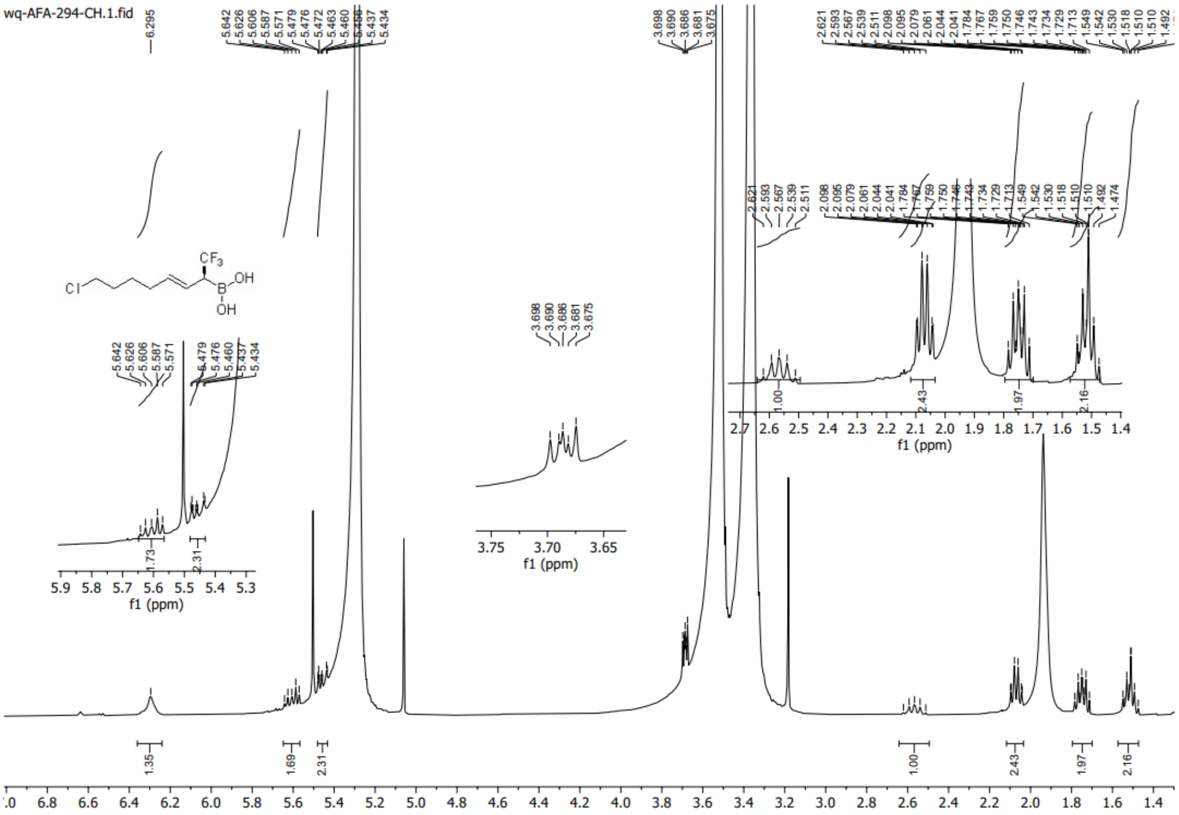

(*S*,*E*)-(1,1,1-Trifluoro-5-phenylpent-3-en-2-yl)boronic acid (**1c**) (^{19}F NMR)

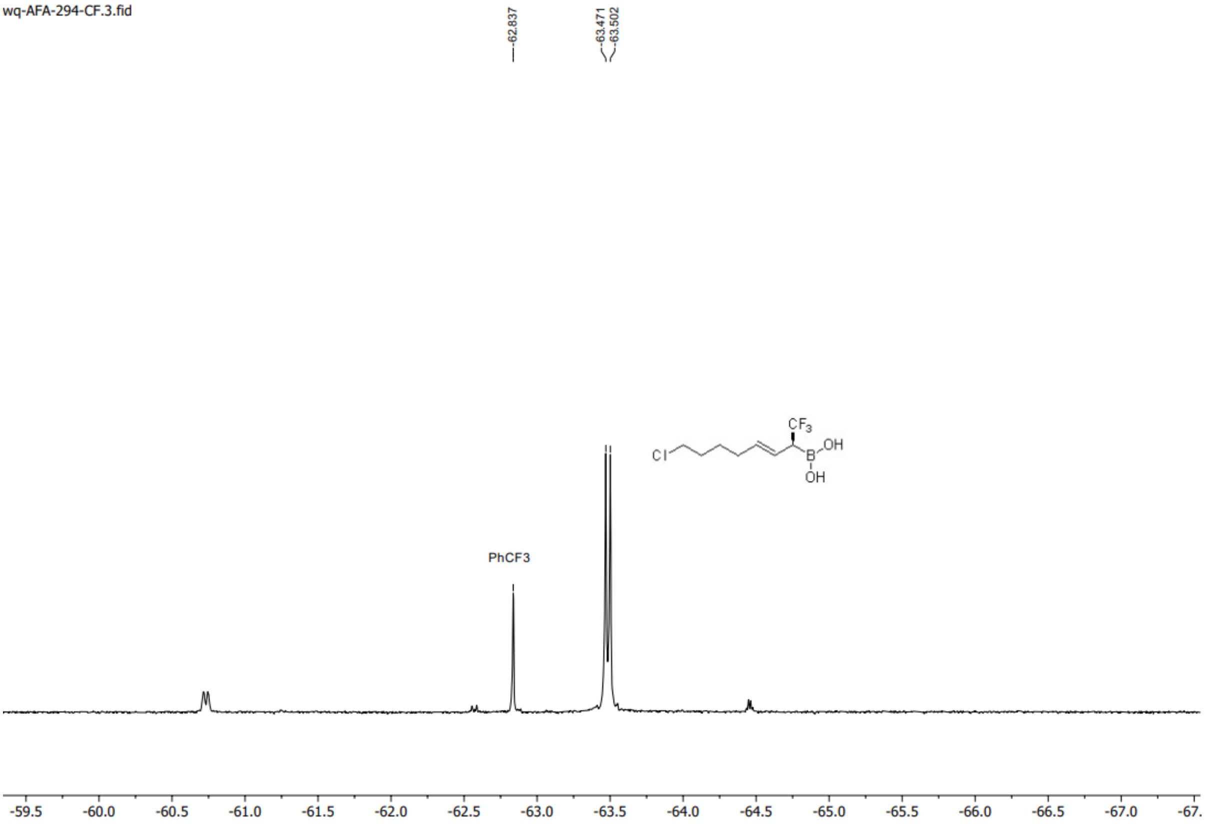

(*S*,*E*)-6,6,6-Trifluoro-5-(1*H*-naphtho[1,8-*de*][1,3,2]diazaborinin-2(3*H*)-yl)hex-3-en-1-yl 4-methylbenzenesulfonate (^{1}H NMR)

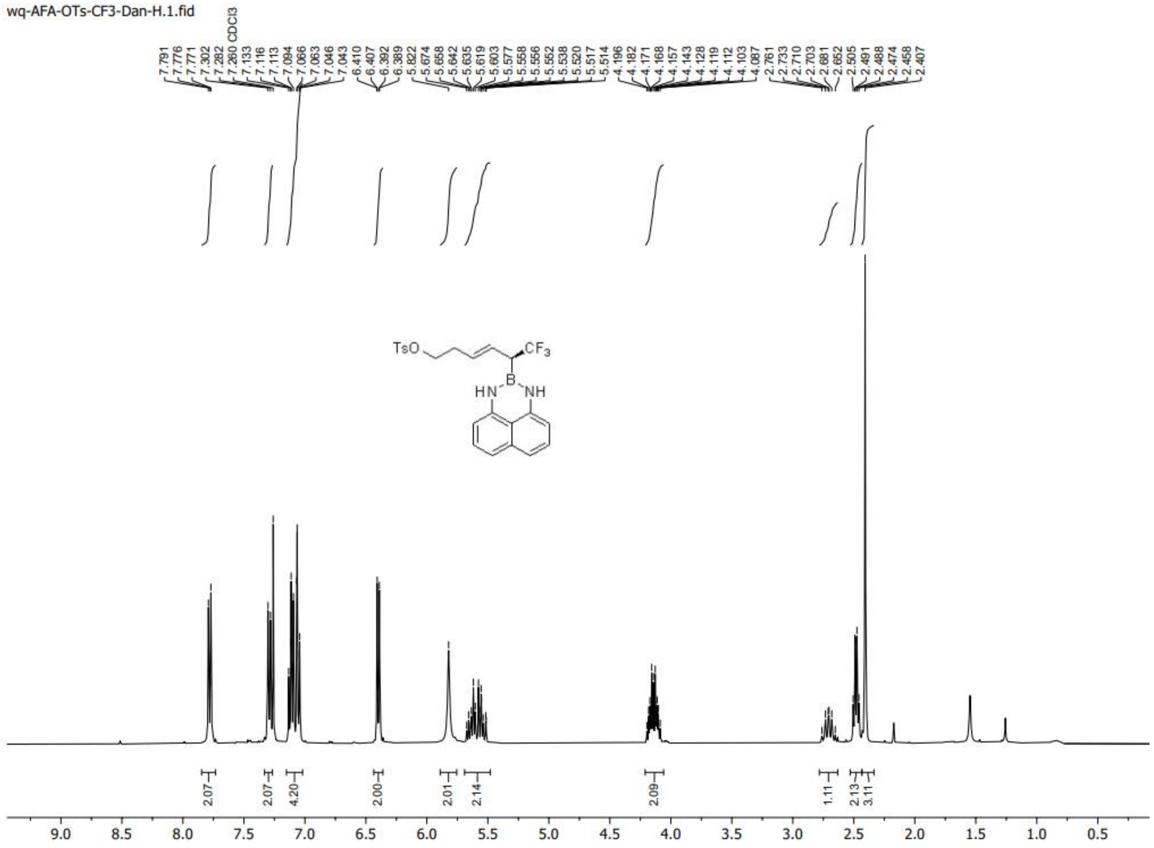

(*S*,*E*)-6,6,6-Trifluoro-5-(1*H*-naphtho[1,8-*de*][1,3,2]diazaborinin-2(3*H*)-yl)hex-3-en-1-yl 4-methylbenzenesulfonate (^{13}C NMR)

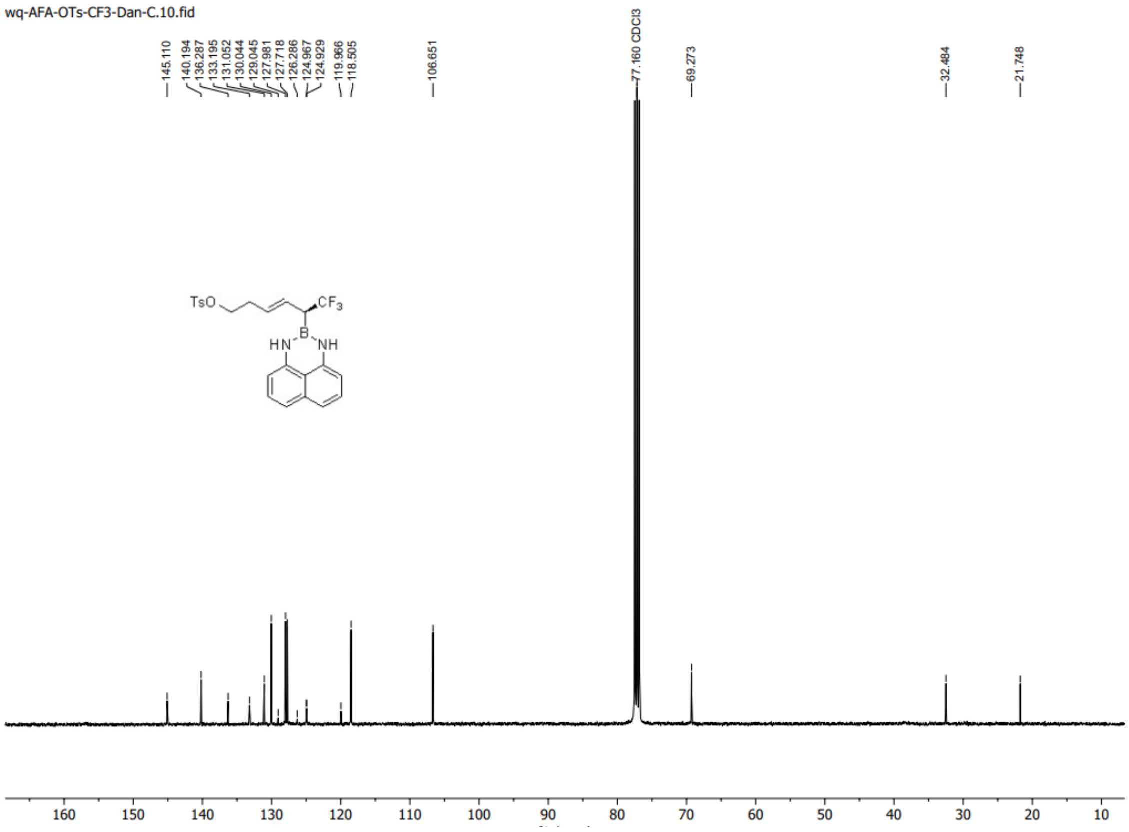

(*S*,*E*)-6,6,6-Trifluoro-5-(1*H*-naphtho[1,8-*de*][1,3,2]diazaborinin-2(3*H*)-yl)hex-3-en-1-yl 4-methylbenzenesulfonate (^{19}F NMR)

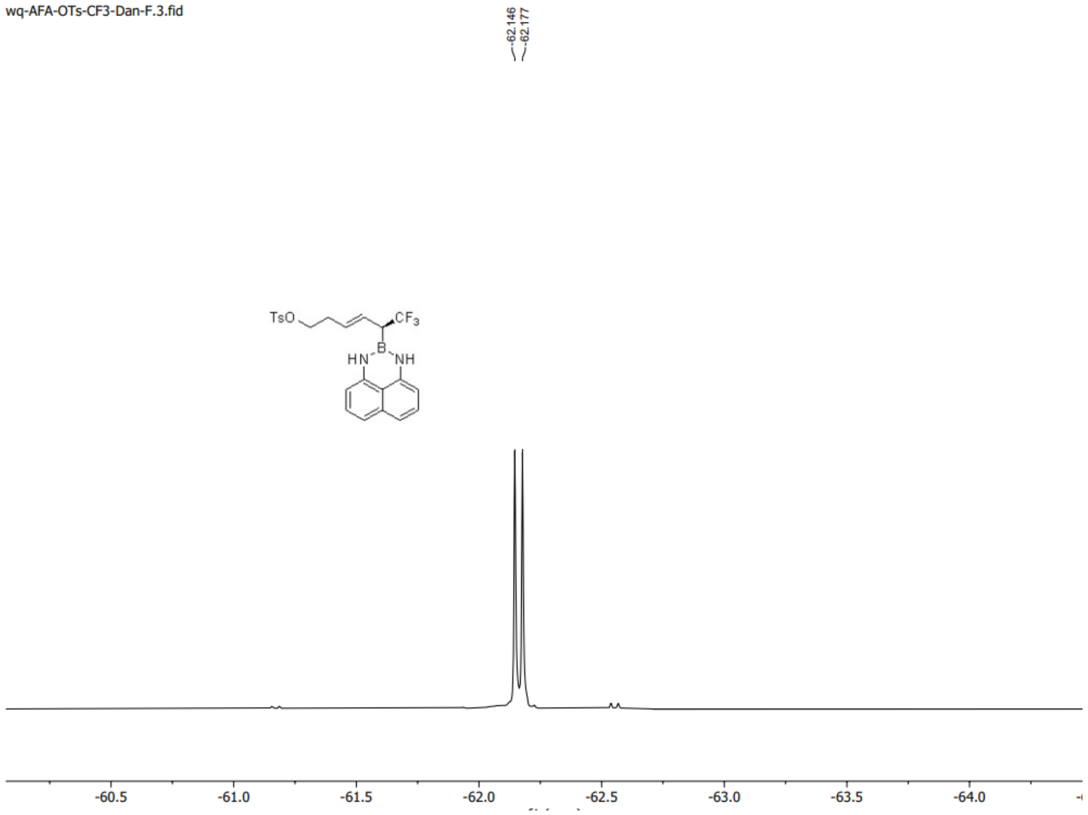

(*S*,*E*)-6,6,6-Trifluoro-5-(1*H*-naphtho[1,8-*de*][1,3,2]diazaborinin-2(3*H*)-yl)hex-3-en-1-yl 4-methylbenzenesulfonate (^{11}B NMR)

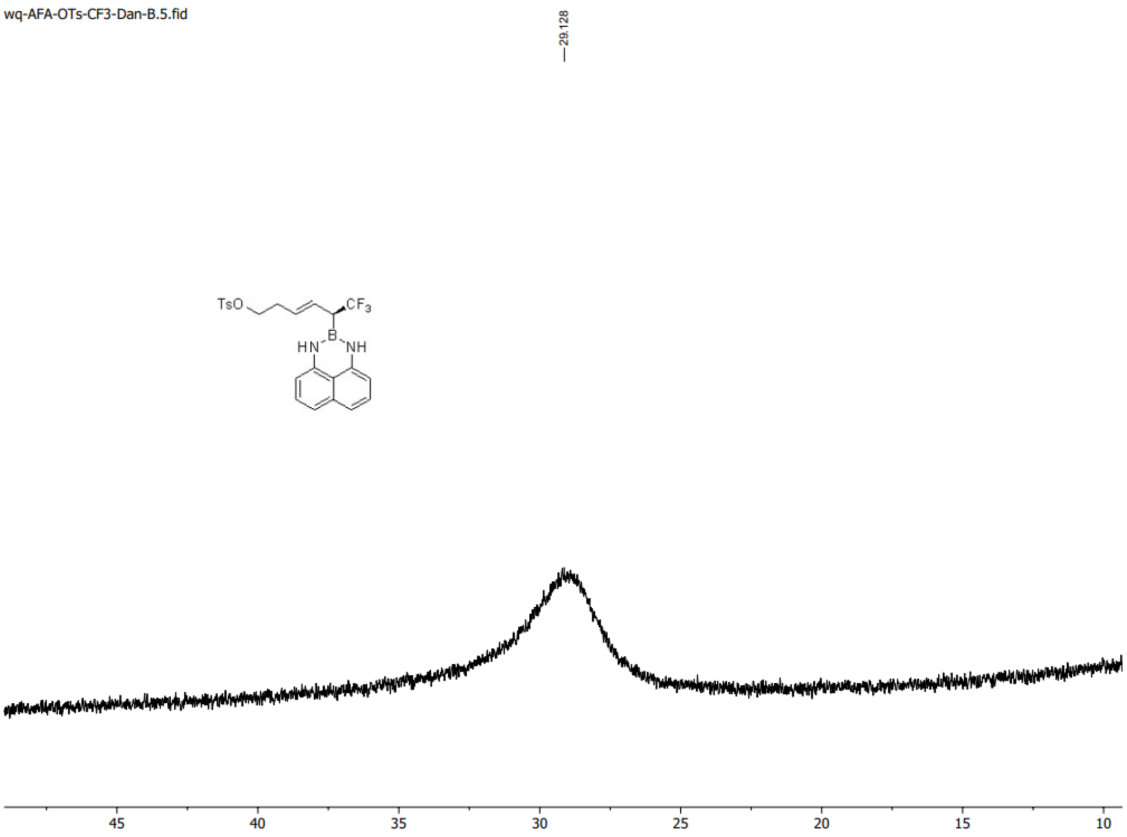

(*S*,*E*)-(1,1,1-Trifluoro-6-(tosyloxy)hex-3-en-2-yl)boronic acid (**1d**) (^{1}H NMR)

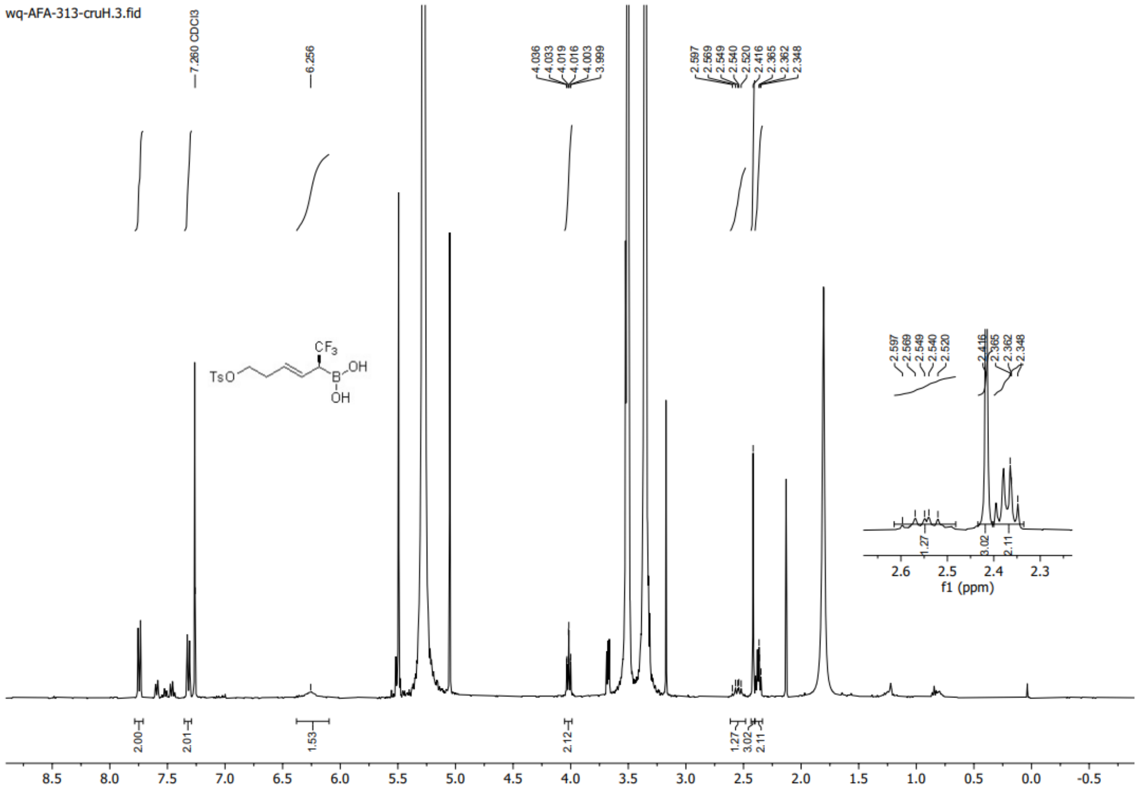

(*S*,*E*)-(1,1,1-Trifluoro-6-(tosyloxy)hex-3-en-2-yl)boronic acid (**1d**) (^{19}F NMR)

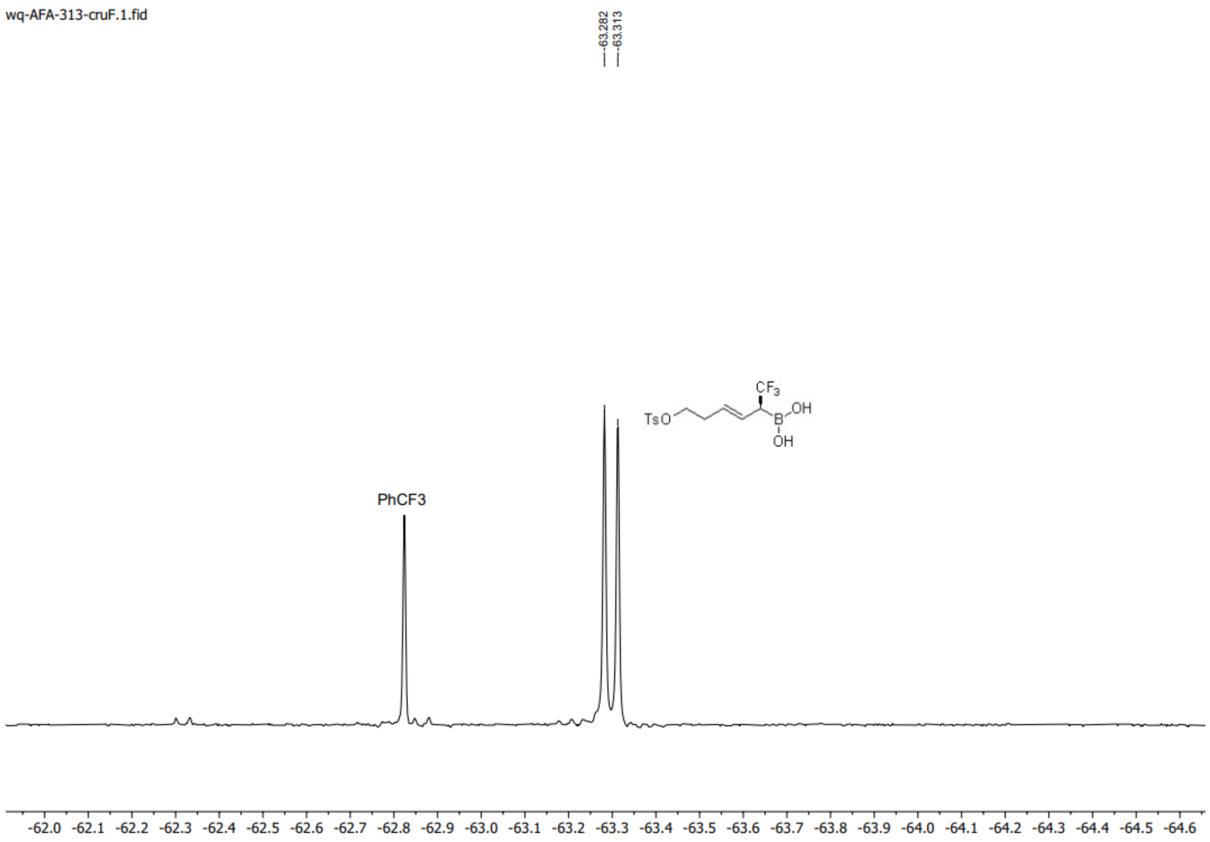

Dethyl (*E*)-(3-(1,3-dioxoisoindolin-2-yl)prop-1-en-1-yl)boronate (^{1}H NMR)

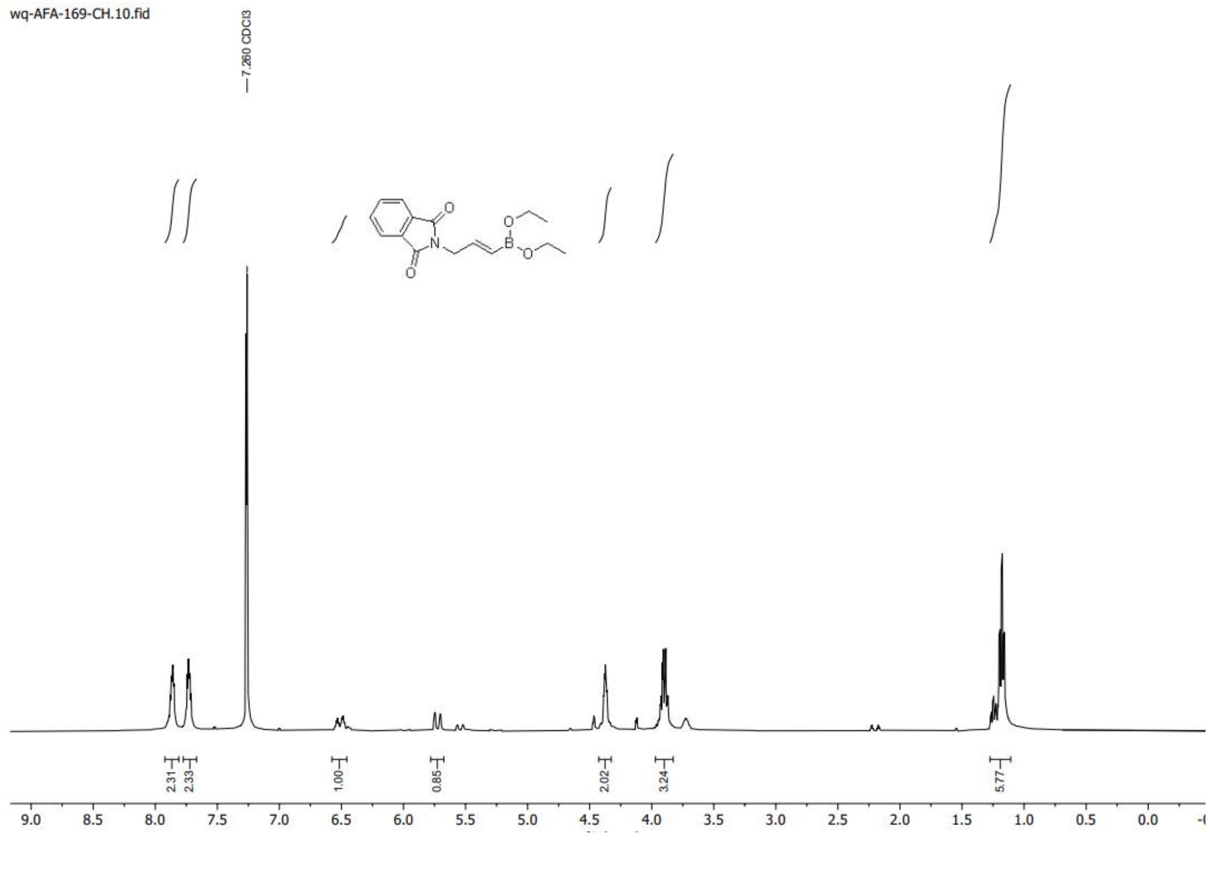

(*S*,*E*)-2-(5,5,5-trifluoro-4-(1*H*-naphtho[1,8-*de*][1,3,2]diazaborinin-2(3*H*)-yl)pent-2-en-1-yl)isoindoline-1,3-dione (^{1}H NMR)

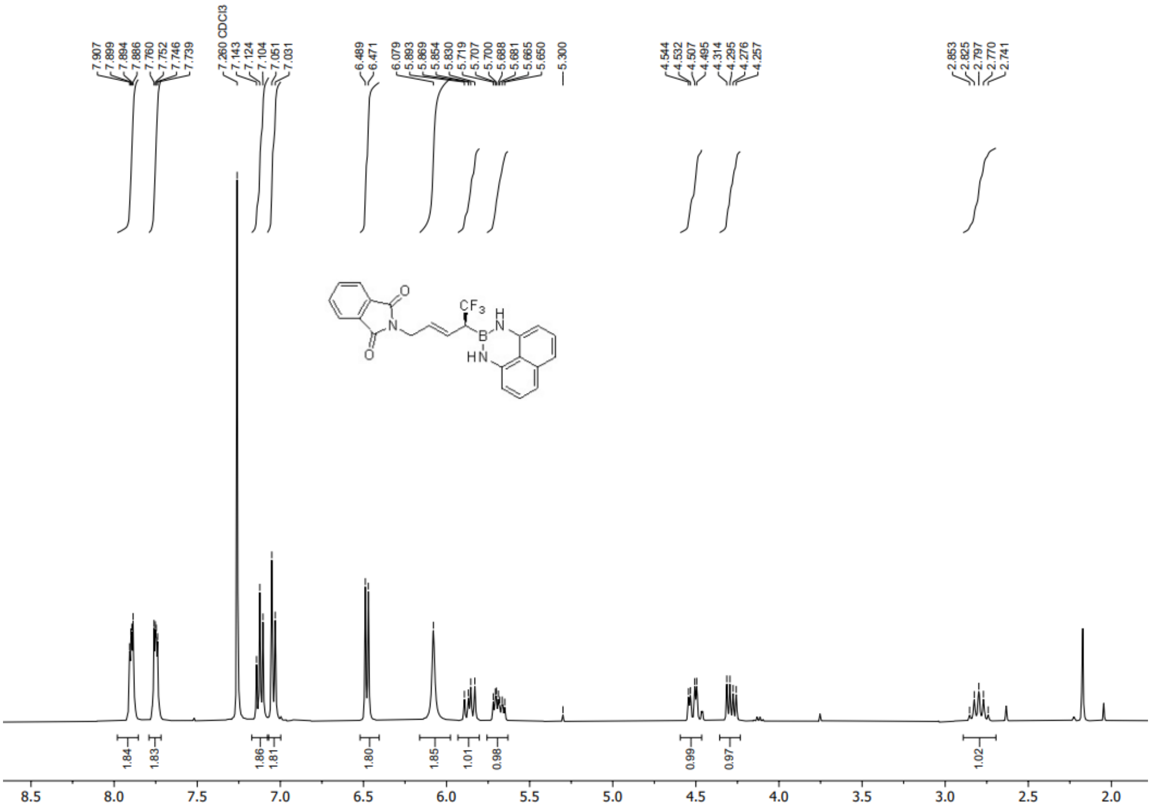

(*S*,*E*)-2-(5,5,5-trifluoro-4-(1*H*-naphtho[1,8-*de*][1,3,2]diazaborinin-2(3*H*)-yl)pent-2-en-1-yl)isoindoline-1,3-dione (^{13}C NMR)

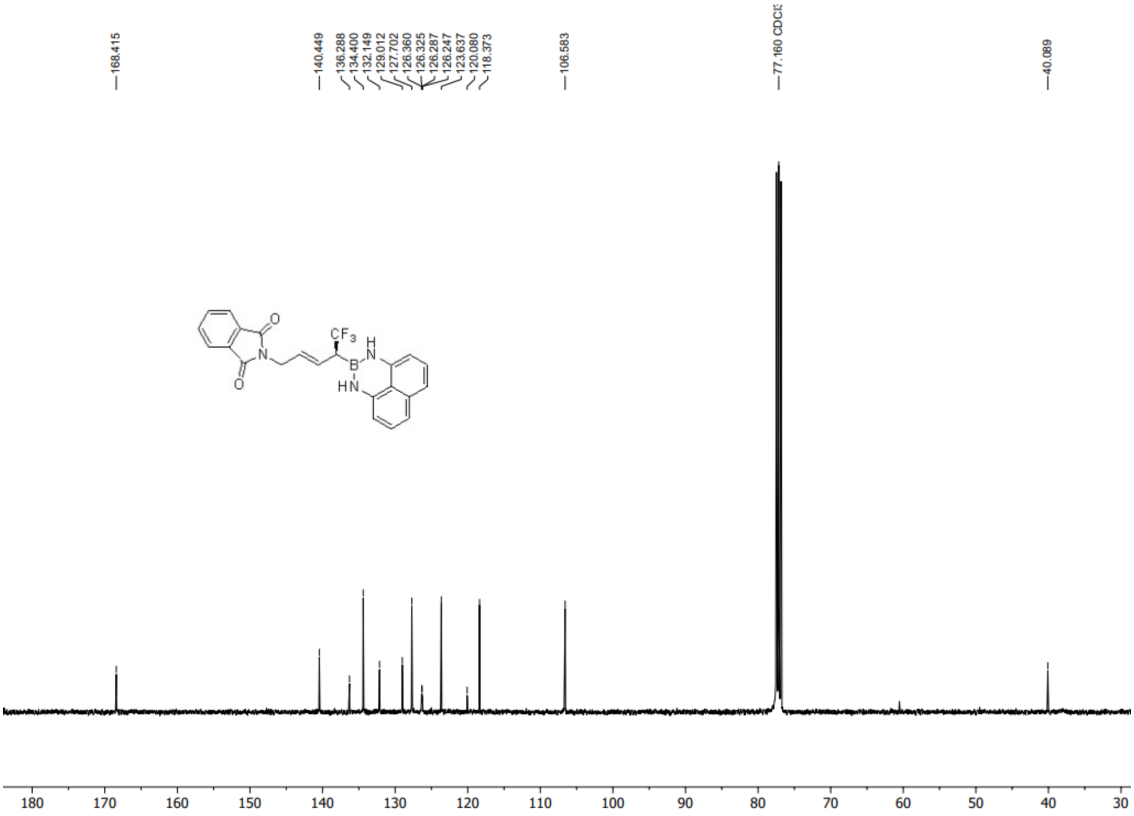

(*S*,*E*)-2-(5,5,5-trifluoro-4-(1*H*-naphtho[1,8-*de*][1,3,2]diazaborinin-2(3*H*)-yl)pent-2-en-1-yl)isoindoline-1,3-dione (^{19}F NMR)

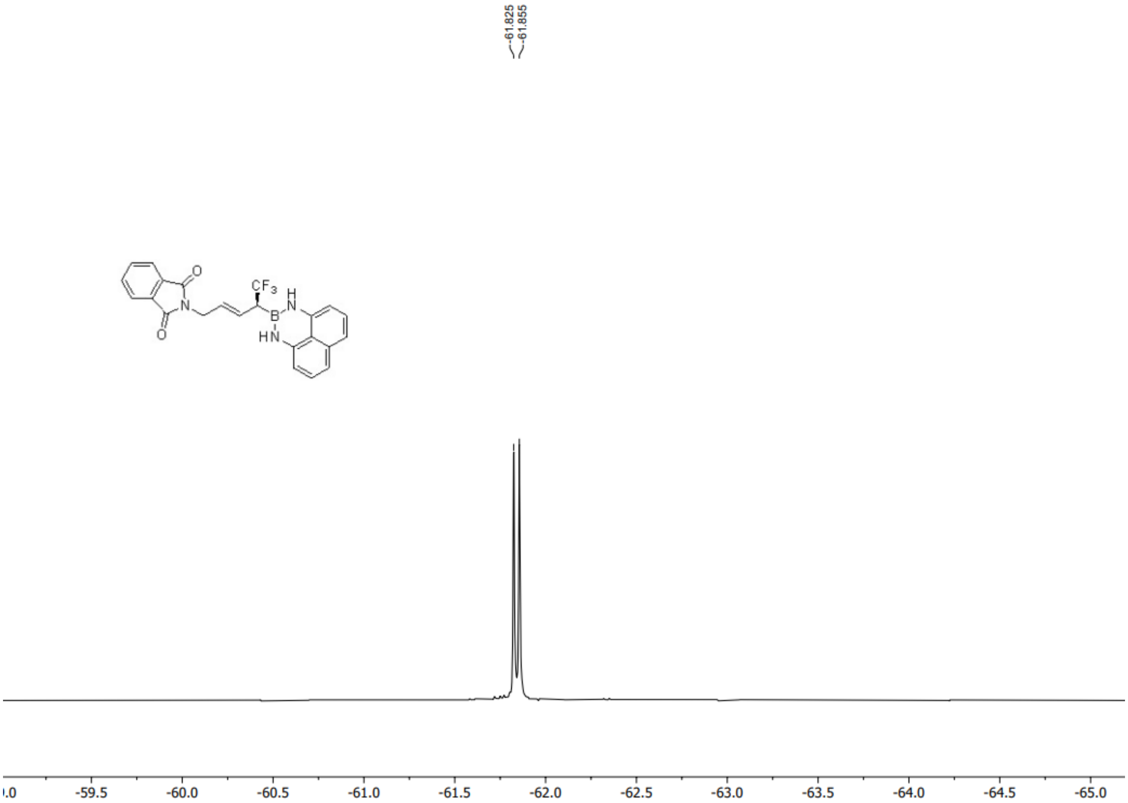

(*S*,*E*)-2-(5,5,5-trifluoro-4-(1*H*-naphtho[1,8-*de*][1,3,2]diazaborinin-2(3*H*)-yl)pent-2-en-1-yl)isoindoline-1,3-dione (^{11}B NMR)

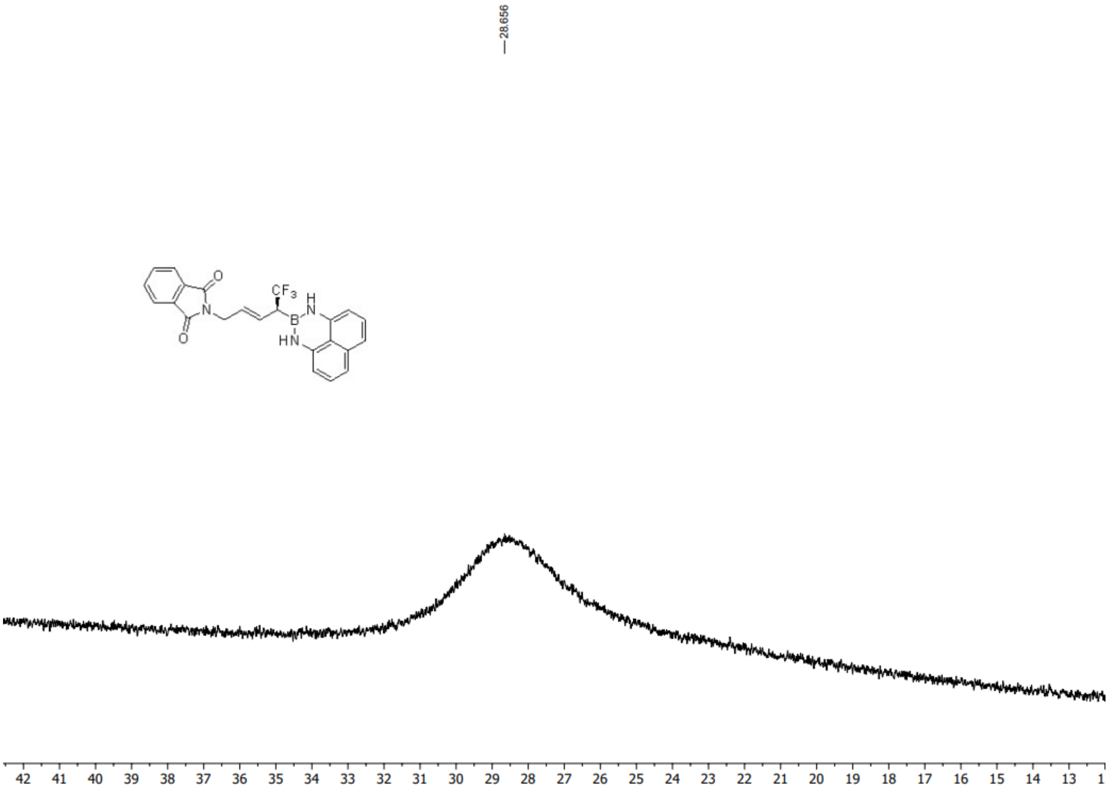

(*S*,*E*)-(5-(1,3-Dioxoisoindolin-2-yl)-1,1,1-trifluoropent-3-en-2-yl)boronic acid (**1e**) (^{1}H NMR)

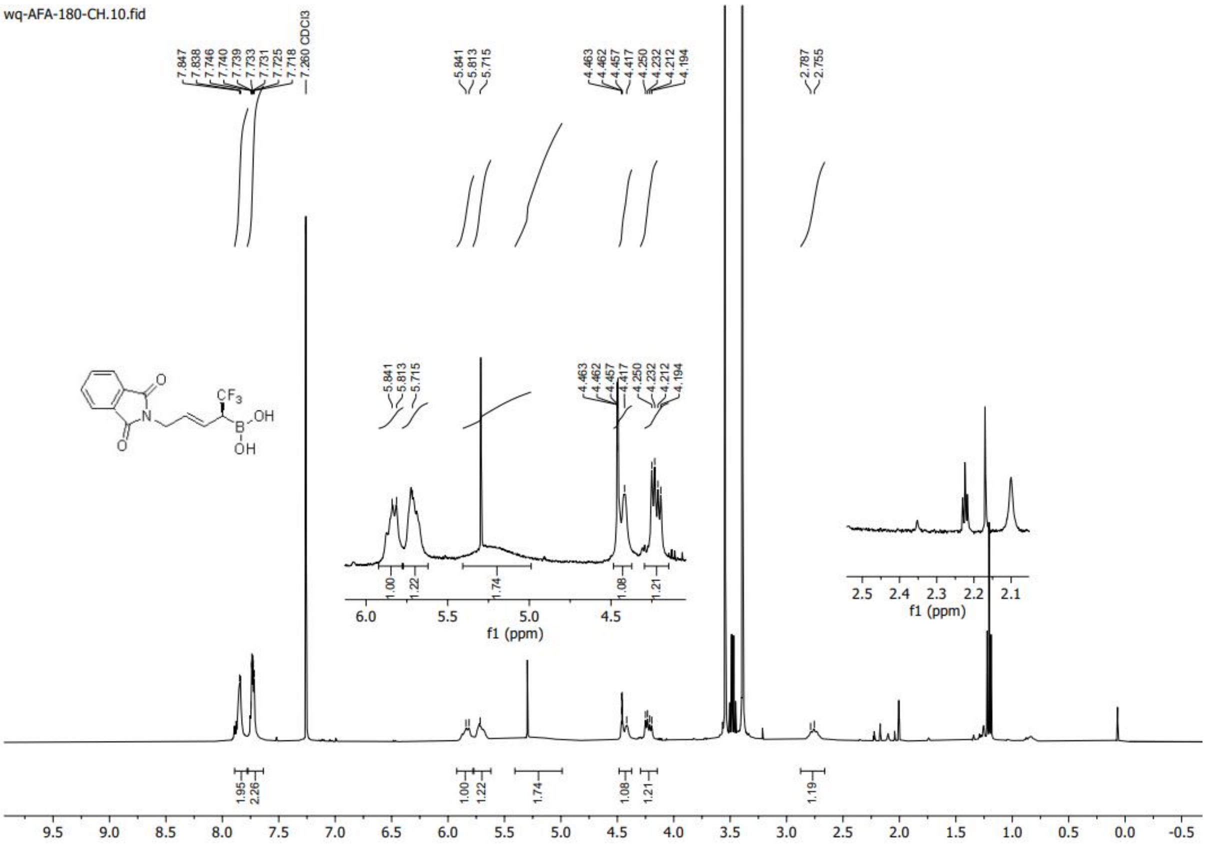

(*S*,*E*)-(5-(1,3-Dioxoisoindolin-2-yl)-1,1,1-trifluoropent-3-en-2-yl)boronic acid (**1e**) (^{19}F NMR)

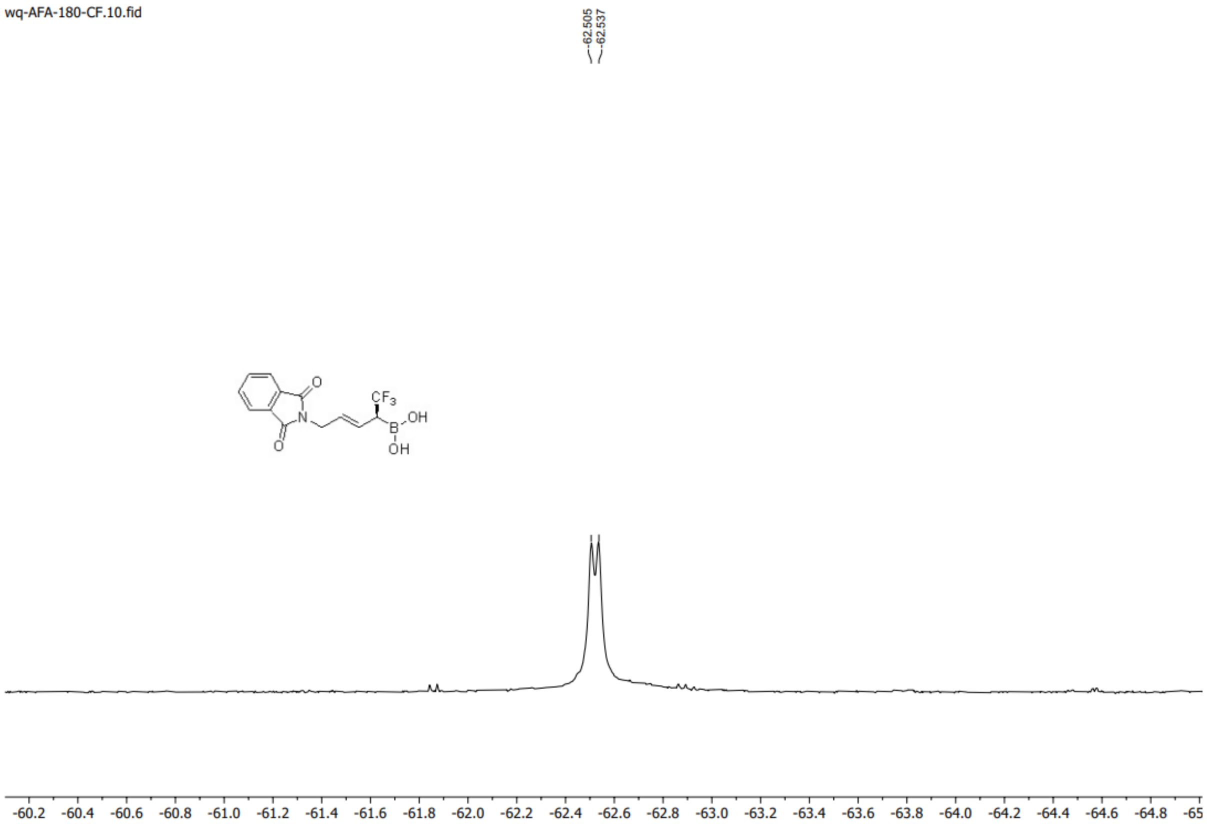

Diethyl (*E*)-(2-cyclohexylvinyl)boronate (^{1}H NMR)

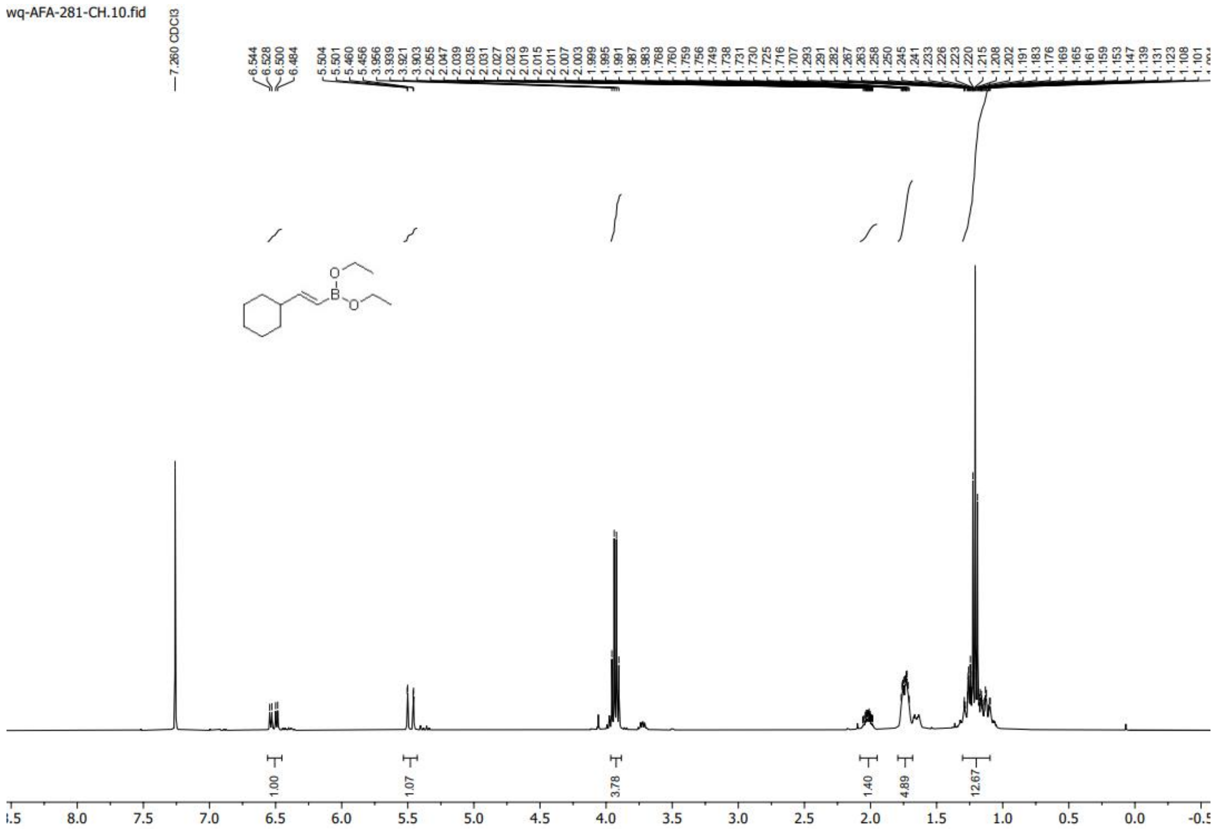

(*S*,*E*)-2-(4-Cyclohexyl-1,1,1-trifluorobut-3-en-2-yl)-2,3-dihydro-1*H*-naphtho[1,8-*de*][1,3,2]diazaborinine (^{1}H NMR)

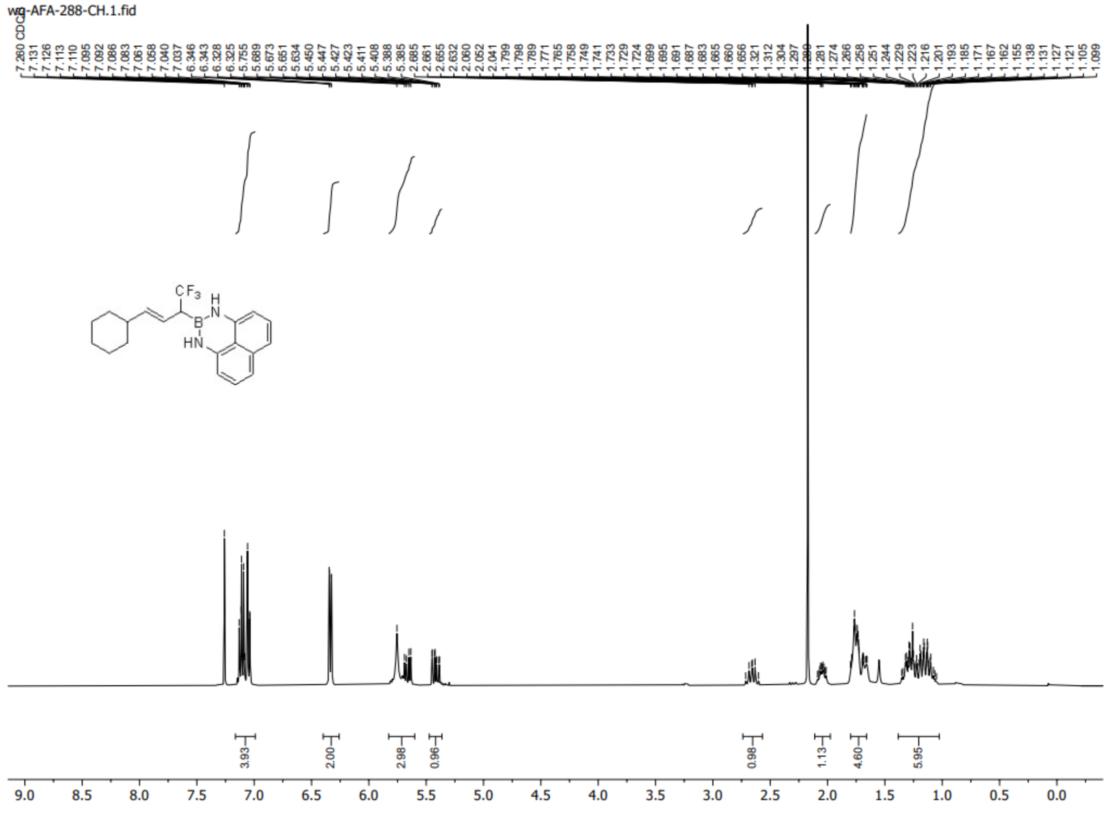

(*S*,*E*)-2-(4-Cyclohexyl-1,1,1-trifluorobut-3-en-2-yl)-2,3-dihydro-1*H*-naphtho[1,8-*de*][1,3,2]diazaborinine (^{13}C NMR)

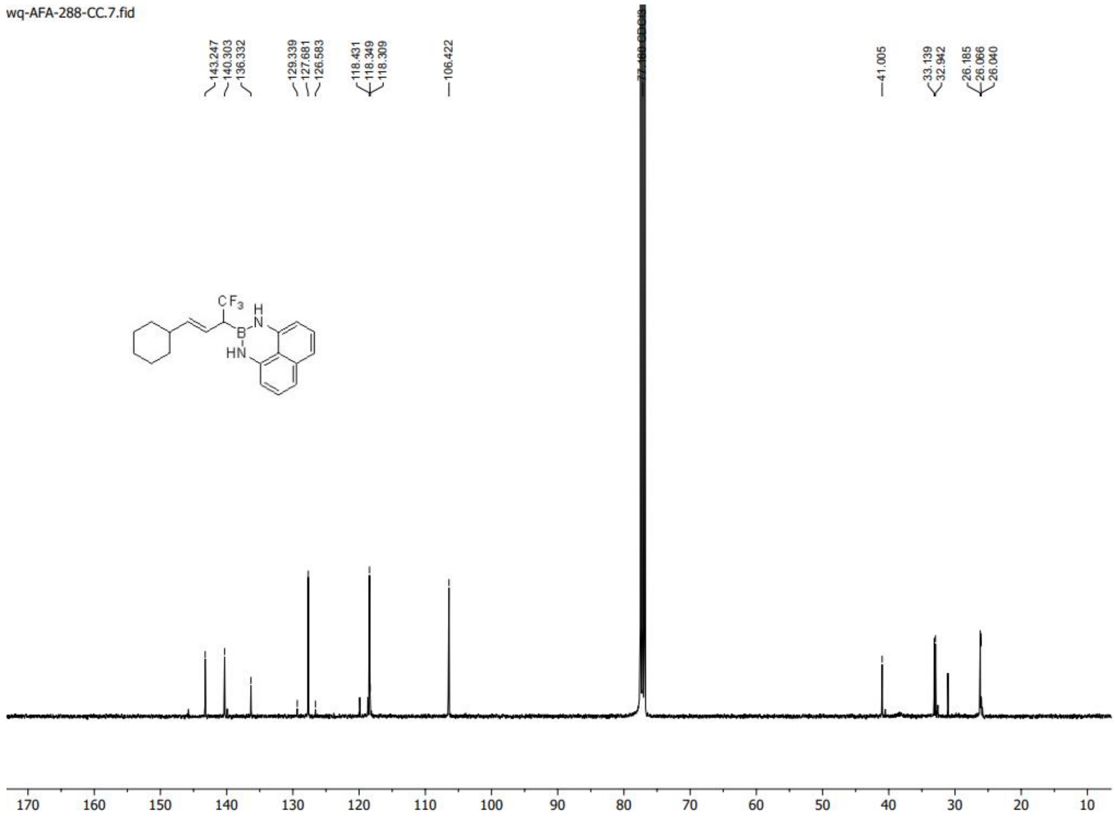

(*S*,*E*)-2-(4-Cyclohexyl-1,1,1-trifluorobut-3-en-2-yl)-2,3-dihydro-1*H*-naphtho[1,8-*de*][1,3,2]diazaborinine (^{19}F NMR)

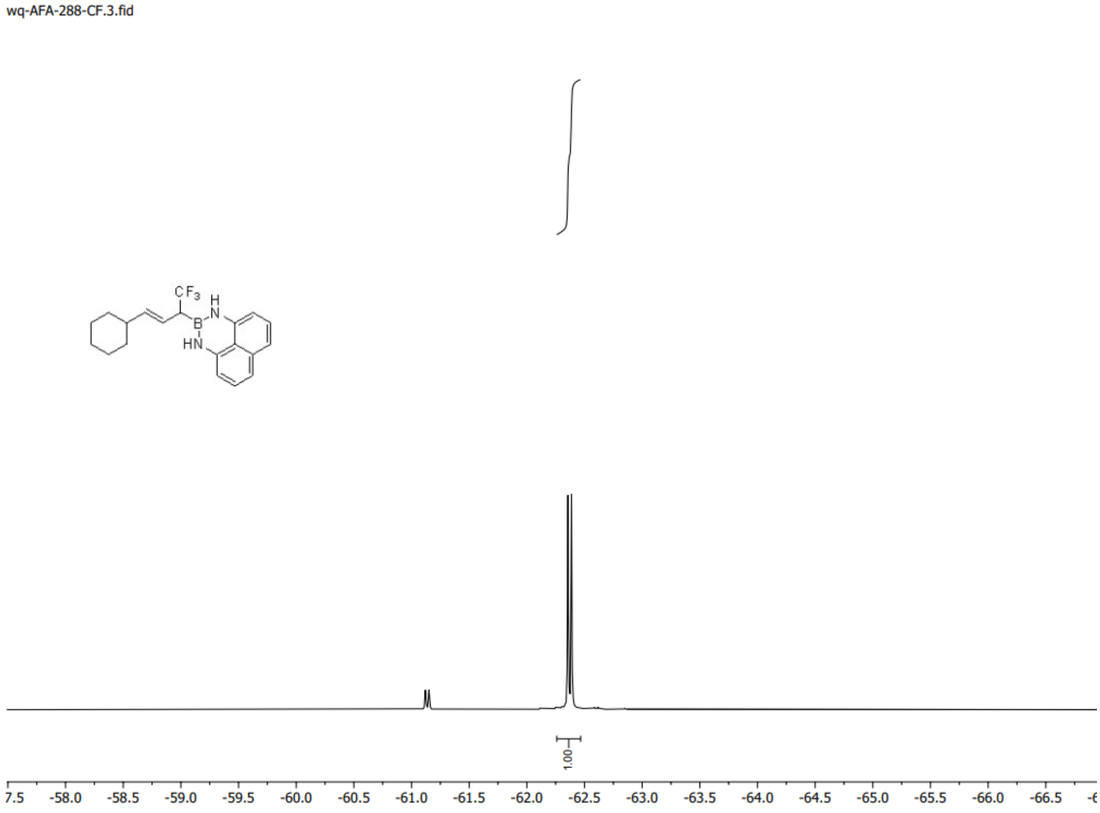

(*S*,*E*)-2-(4-Cyclohexyl-1,1,1-trifluorobut-3-en-2-yl)-2,3-dihydro-1*H*-naphtho[1,8-*de*][1,3,2]diazaborinine (^{11}B NMR)

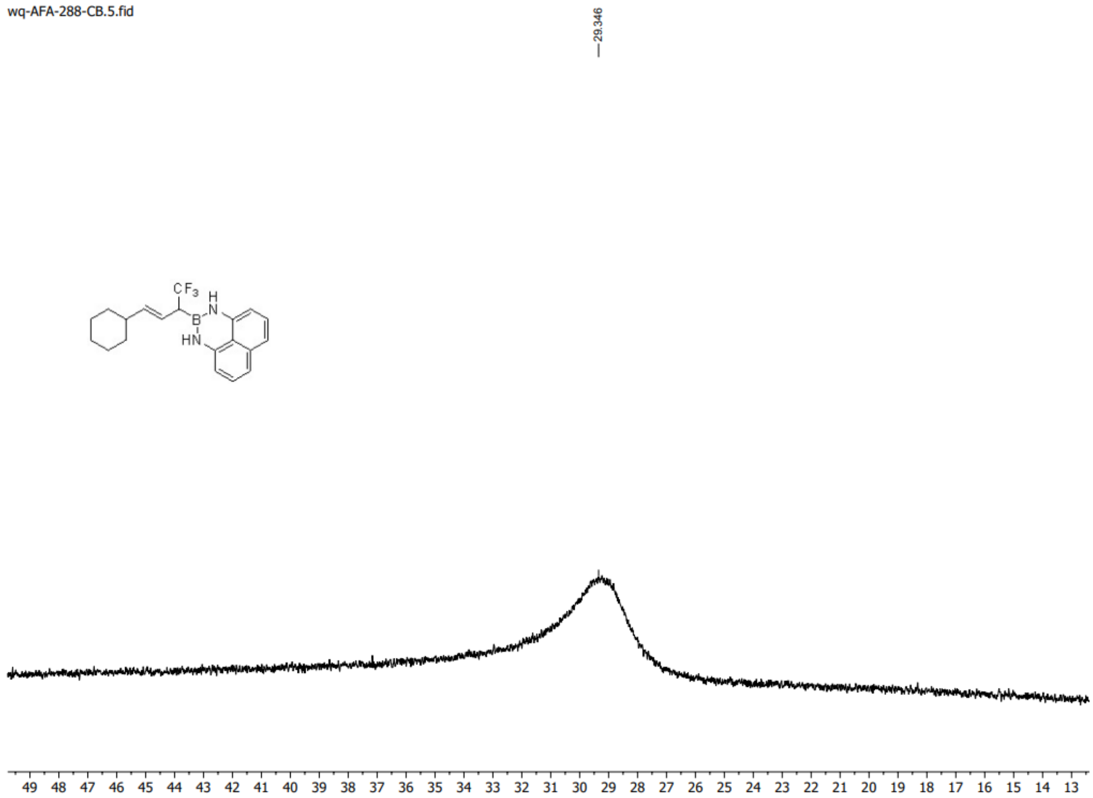

(*S*,*E*)-(4-Cyclohexyl-1,1,1-trifluorobut-3-en-2-yl)boronic acid (**1f**) (^{1}H NMR)

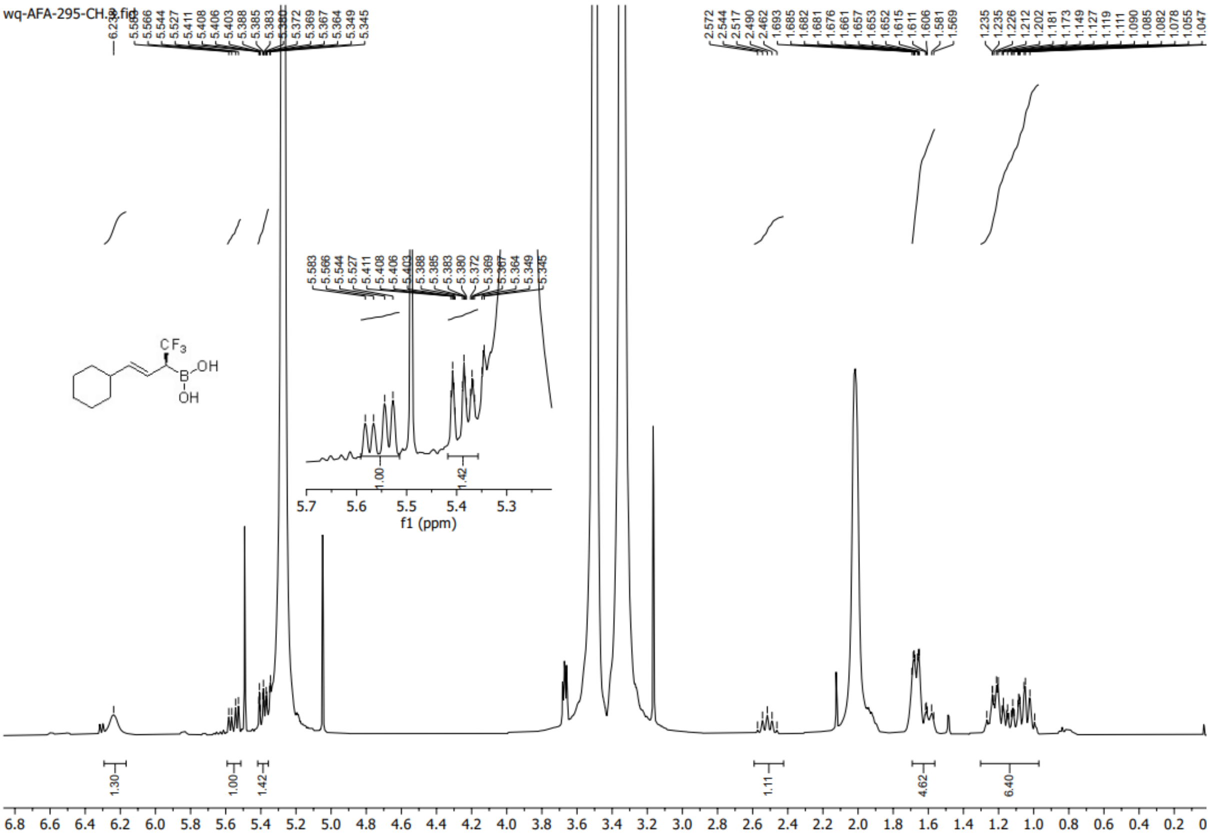

(*S*,*E*)-(4-Cyclohexyl-1,1,1-trifluorobut-3-en-2-yl)boronic acid (**1f**) (^{19}F NMR)

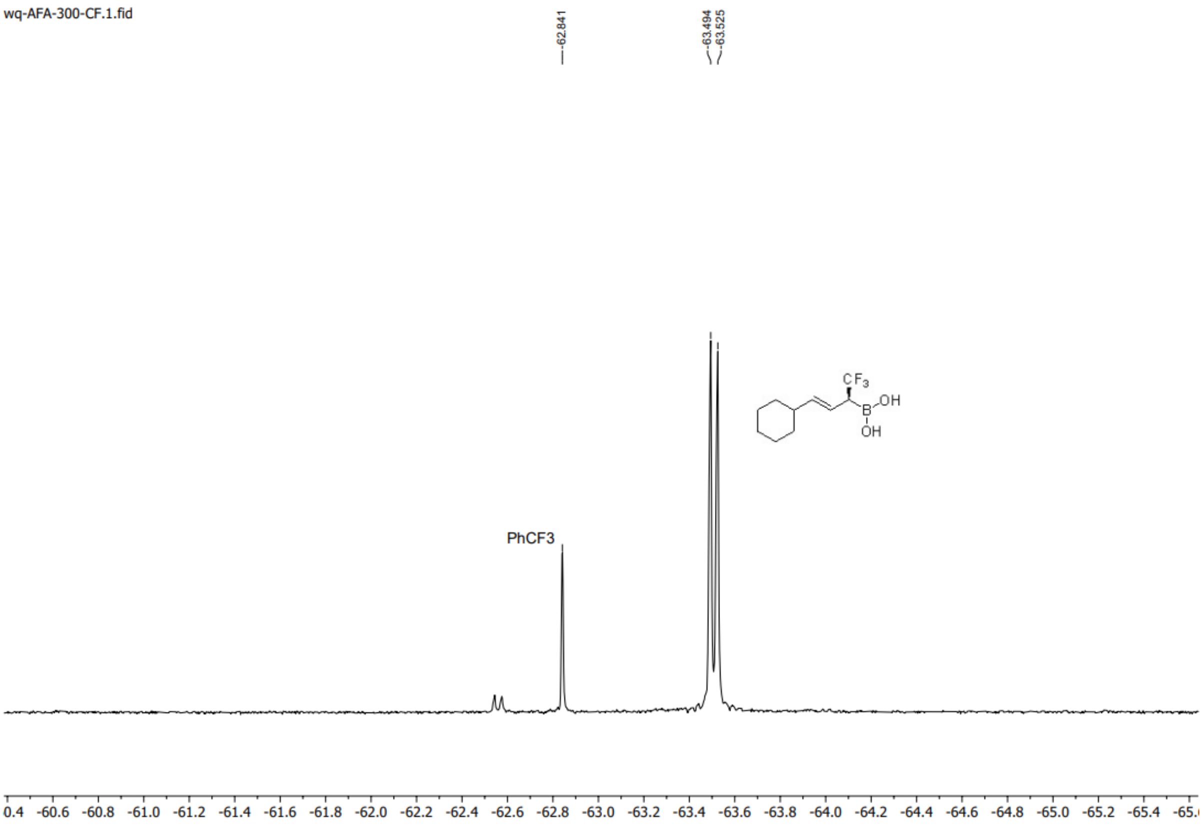

Diethyl (*E*)-styrylboronate (^{1}H NMR)

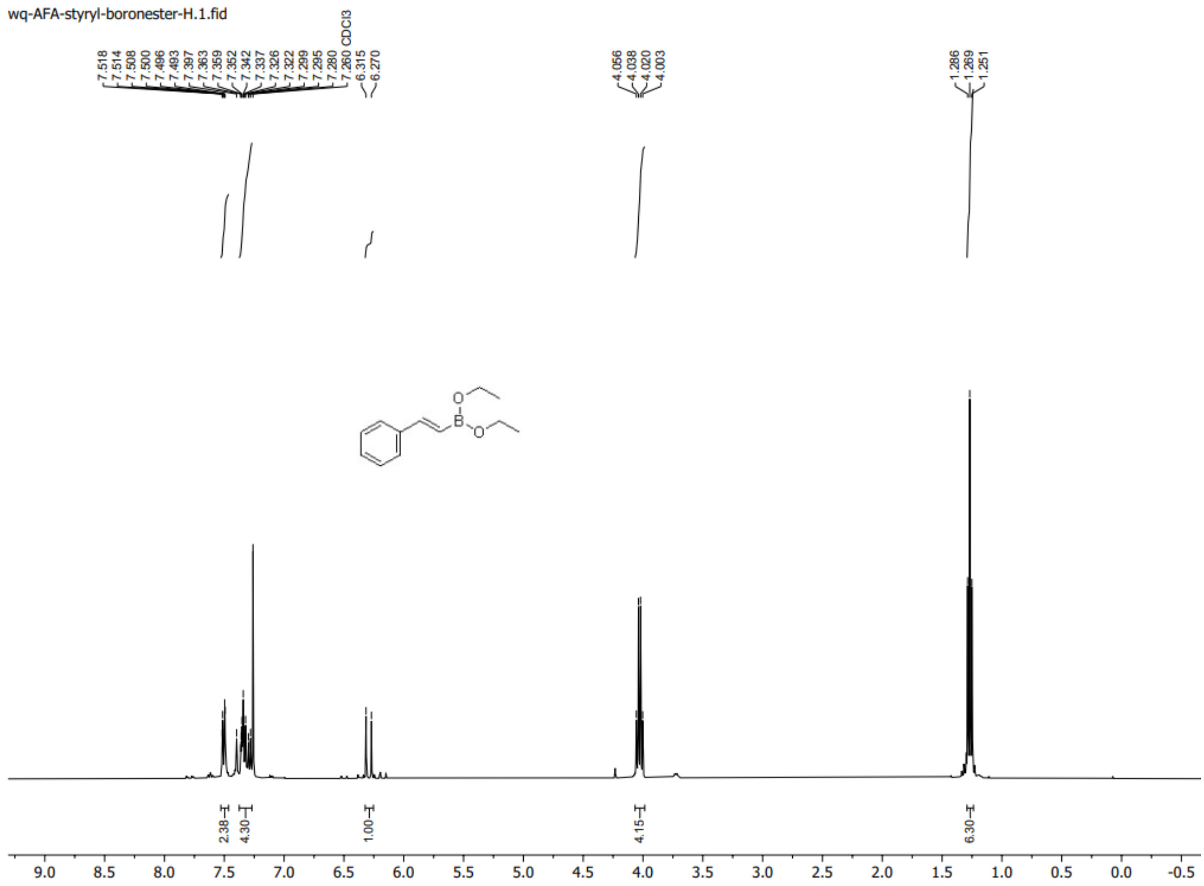

(*S*,*E*)-2-(1,1,1-Trifluoro-4-phenylbut-3-en-2-yl)-2,3-dihydro-1*H*-naphtho[1,8-*de*][1,3,2]diazaborinine (^{1}H NMR)

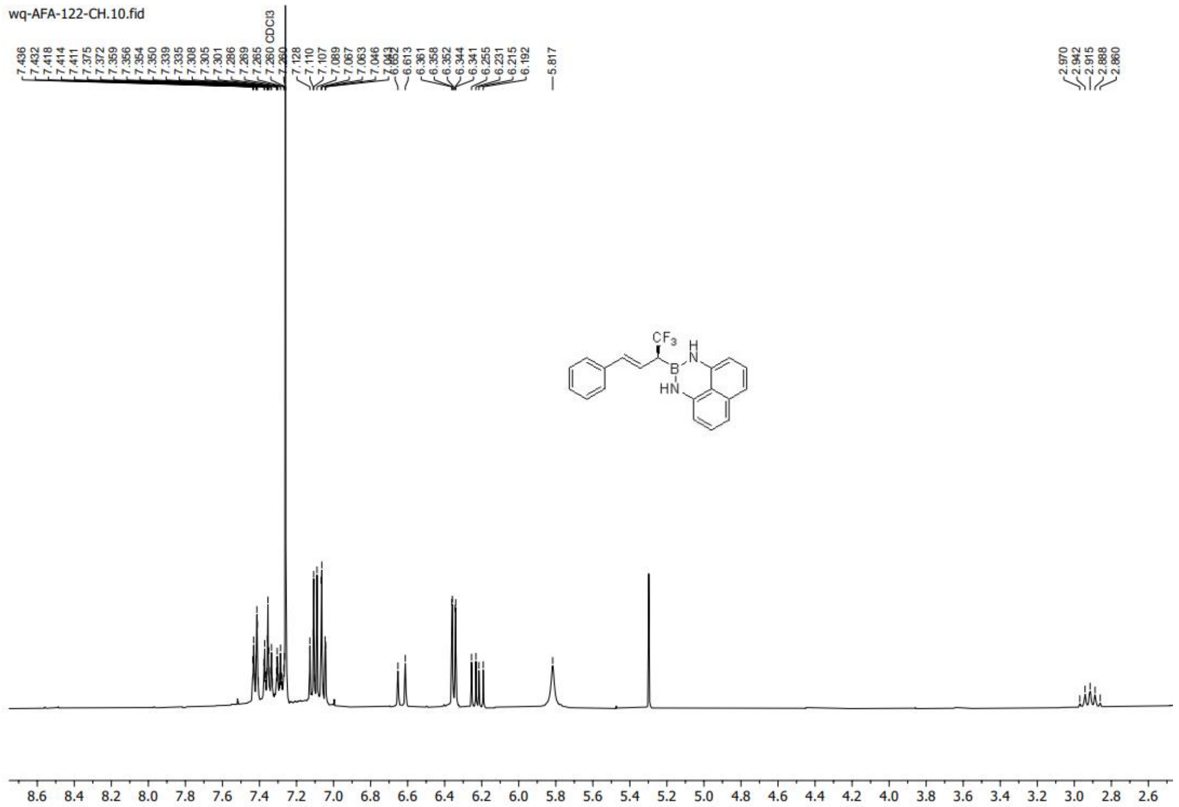

(*S*,*E*)-(1,1,1-Trifluoro-4-phenylbut-3-en-2-yl)boronic acid (**1g**) (^{1}H NMR)

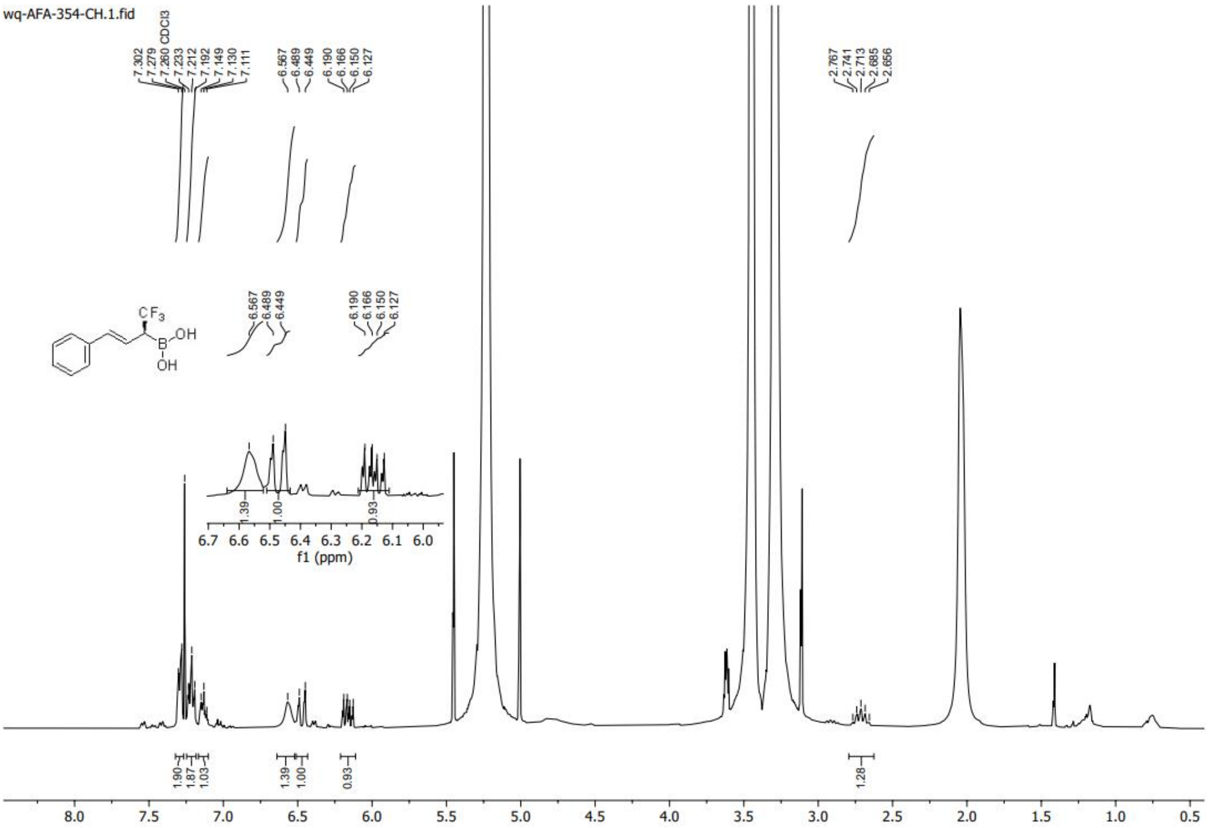

(S,E)-(1,1,1-Trifluoro-4-phenylbut-3-en-2-yl)boronic acid (**1g**) (^{19}F NMR)

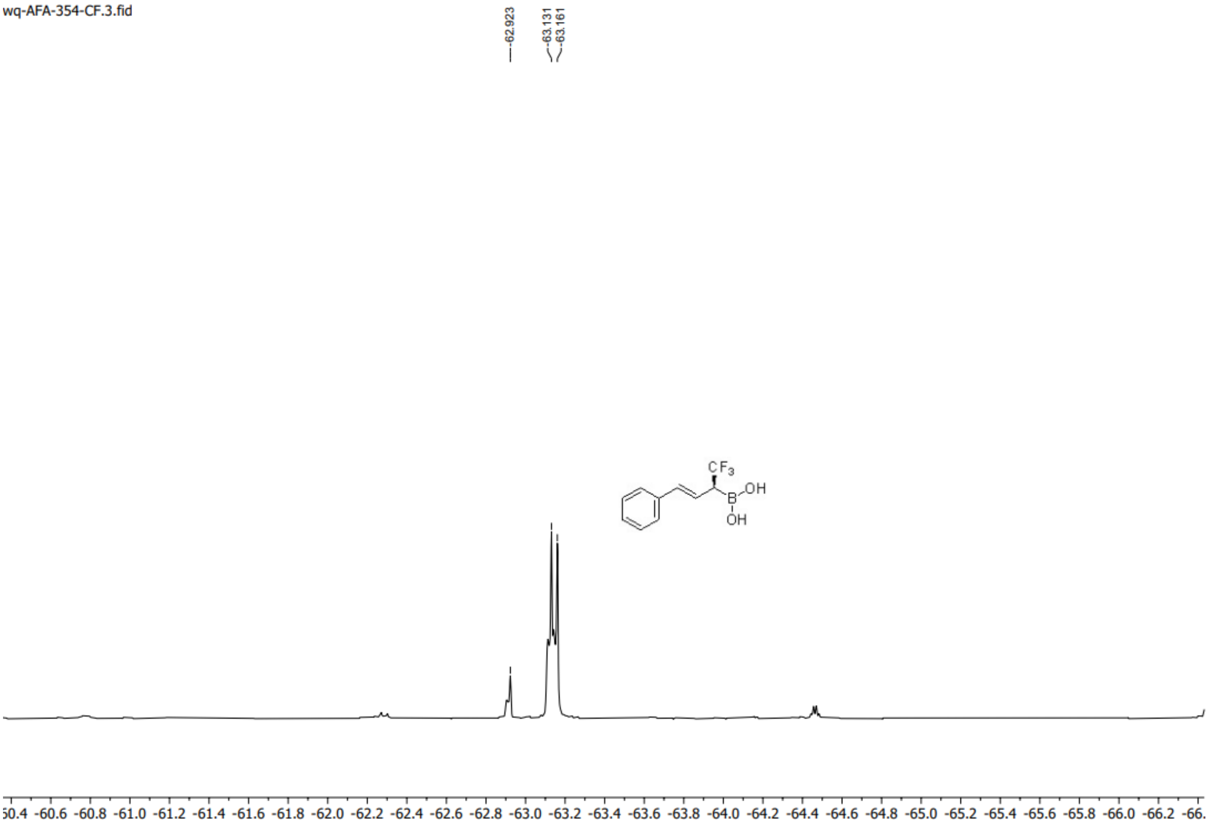

Diethyl (E)-(3,3-dimethylbut-1-en-1-yl)boronate (^{1}H NMR)

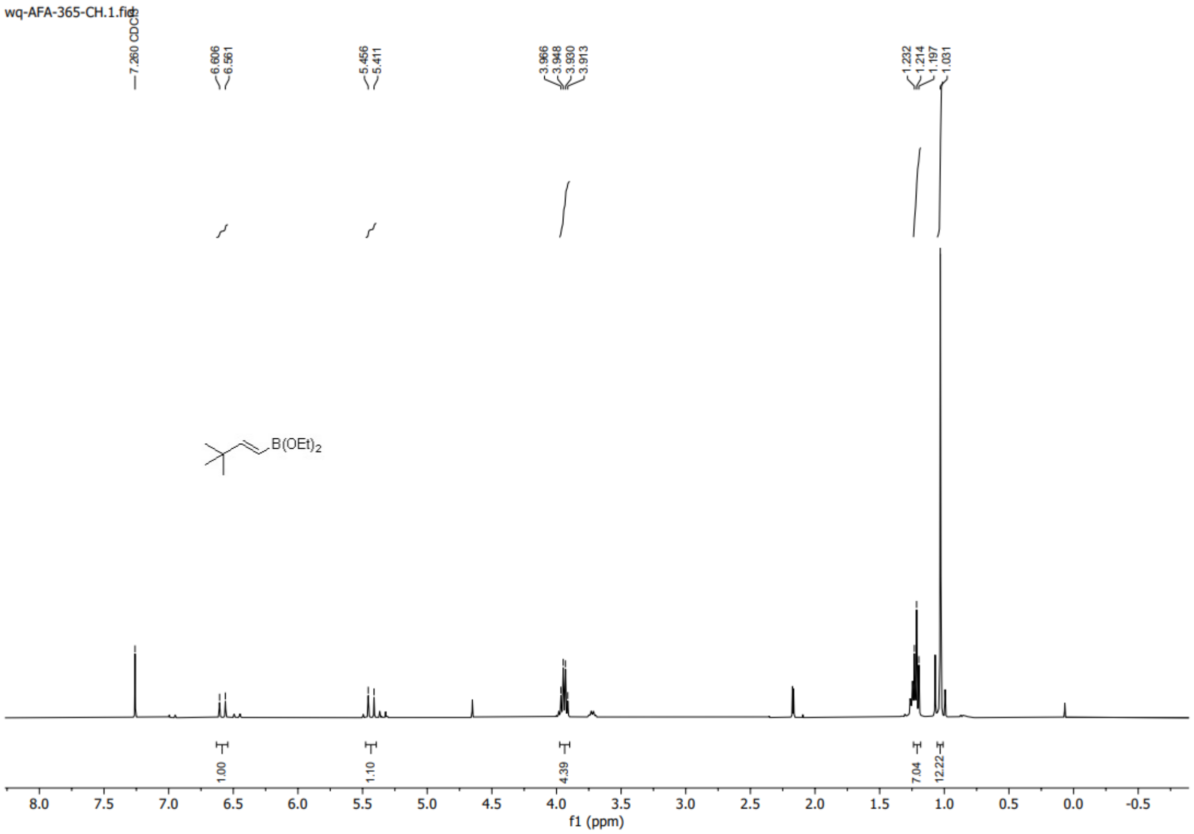

(*S*,*E*)-2-(1,1,1-trifluoro-5,5-dimethylhex-3-en-2-yl)-2,3-dihydro-1*H*-naphtho[1,8-de][1,3,2]diazaborinine (^{1}H NMR)

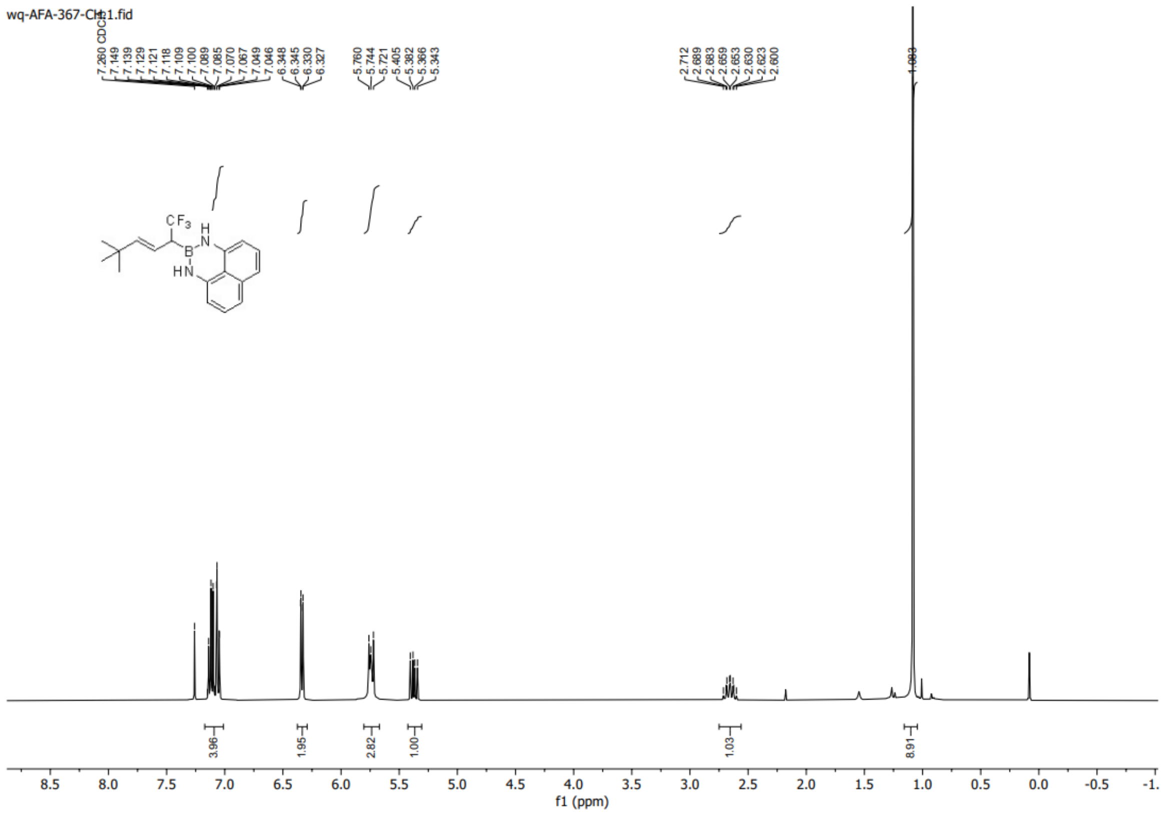

(*S*,*E*)-2-(1,1,1-trifluoro-5,5-dimethylhex-3-en-2-yl)-2,3-dihydro-1*H*-naphtho[1,8-de][1,3,2]diazaborinine (^{13}C NMR)

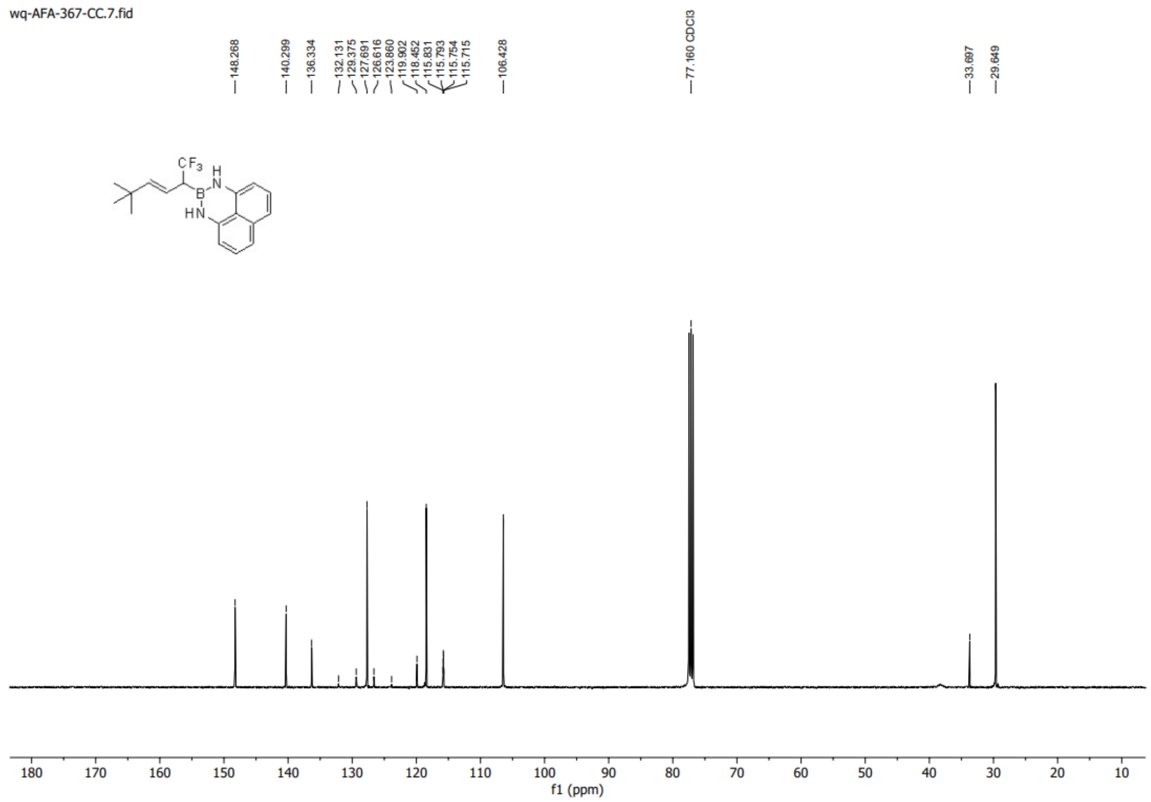

(*S*,*E*)-2-(1,1,1-trifluoro-5,5-dimethylhex-3-en-2-yl)-2,3-dihydro-1*H*-naphtho[1,8-de][1,3,2]diazaborinine (^{19}F NMR)

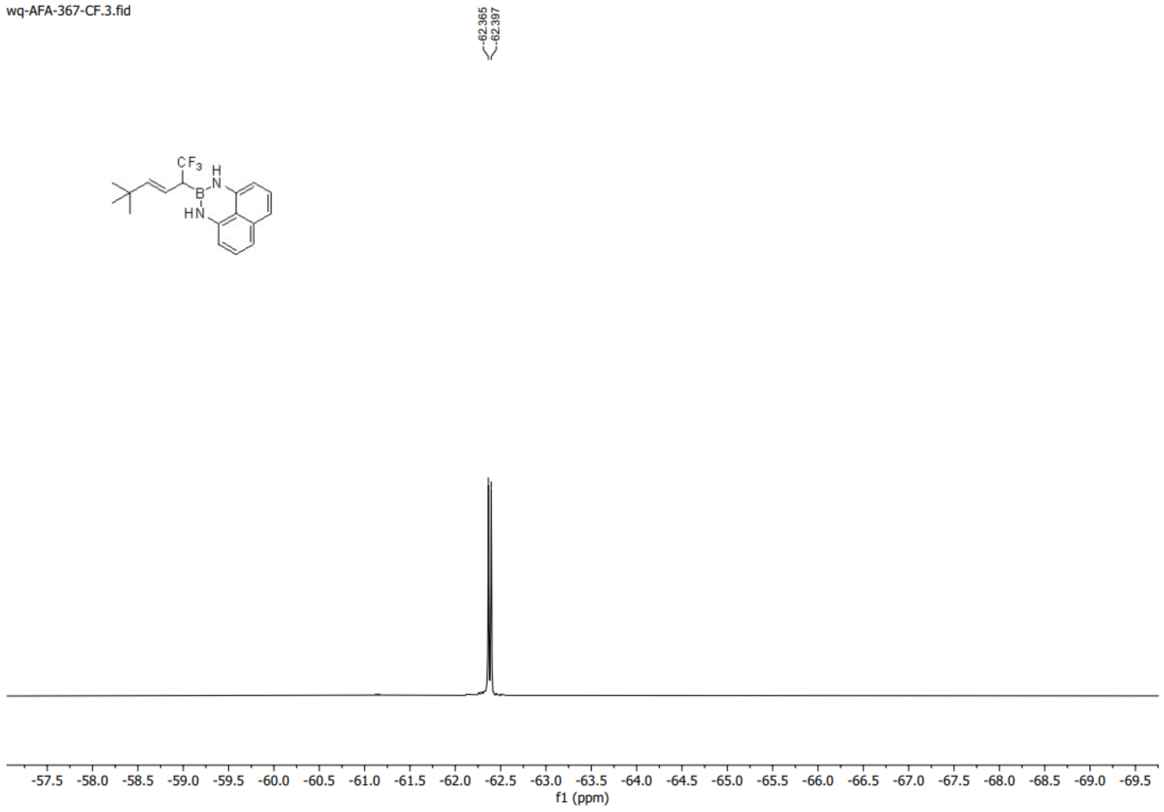

(*S*,*E*)-2-(1,1,1-trifluoro-5,5-dimethylhex-3-en-2-yl)-2,3-dihydro-1*H*-naphtho[1,8-de][1,3,2]diazaborinine (^{11}B NMR)

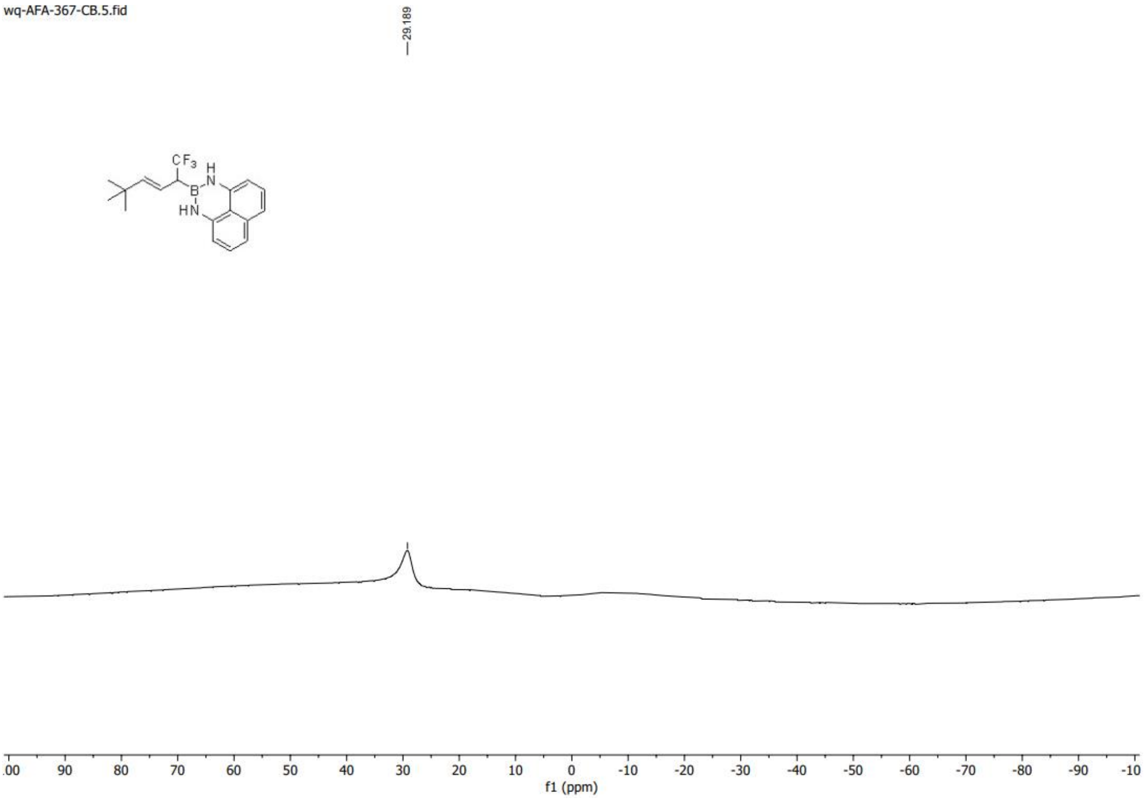

(*S*,*E*)-(1,1,1-trifluoro-5,5-dimethylhex-3-en-2-yl)boronic acid (**1h**) (^{1}H NMR)

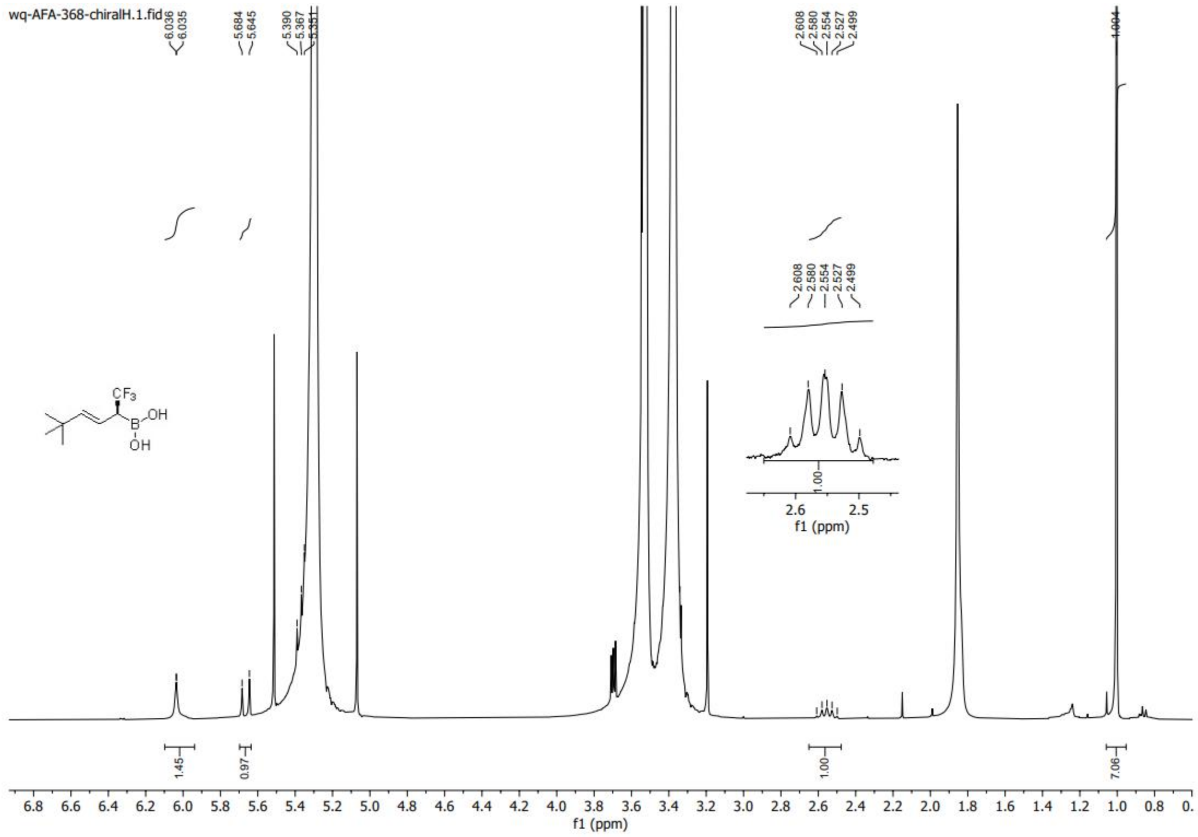

(*S*,*E*)-(1,1,1-trifluoro-5,5-dimethylhex-3-en-2-yl)boronic acid (**1h**) (^{19}F NMR)

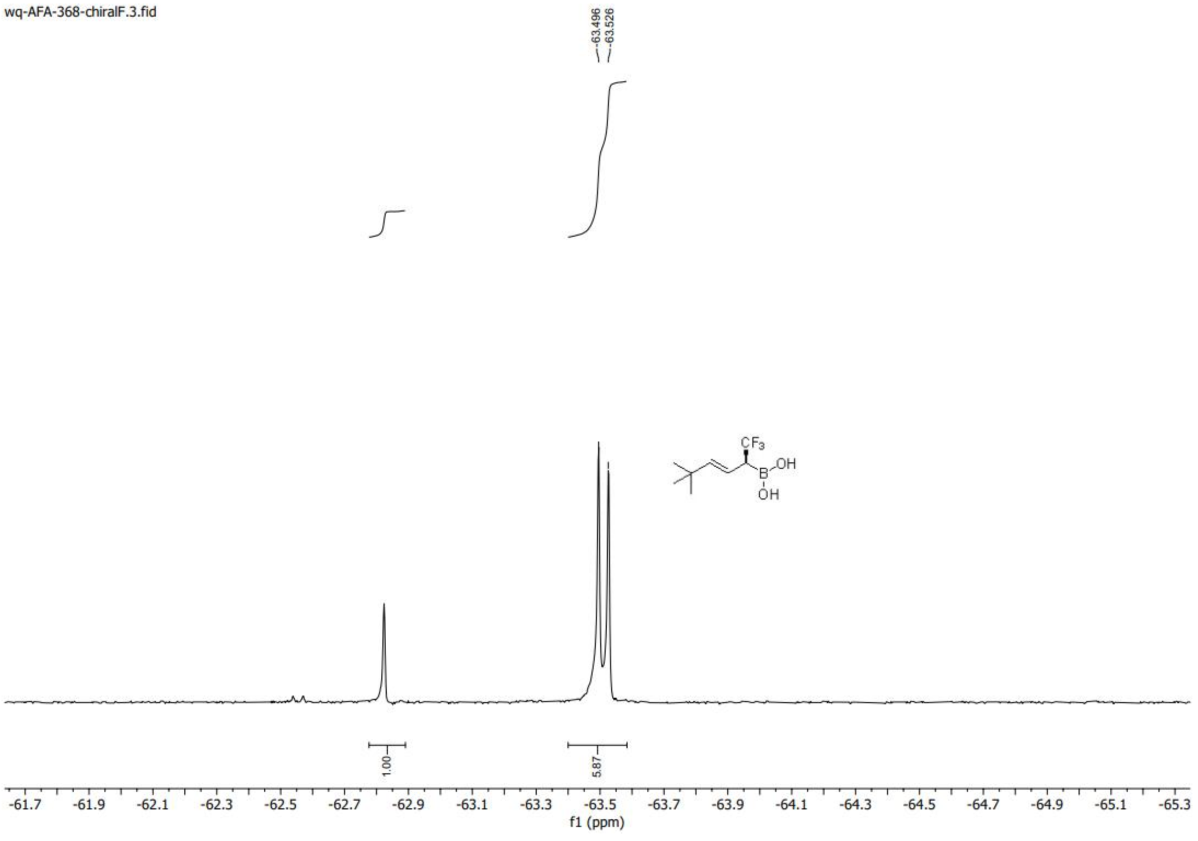

(*R*,*E*)-(1,1,1-Trifluorodec-2-en-4-yl)(trifluoromethyl)sulfane (**3a**) (^{1}H NMR)

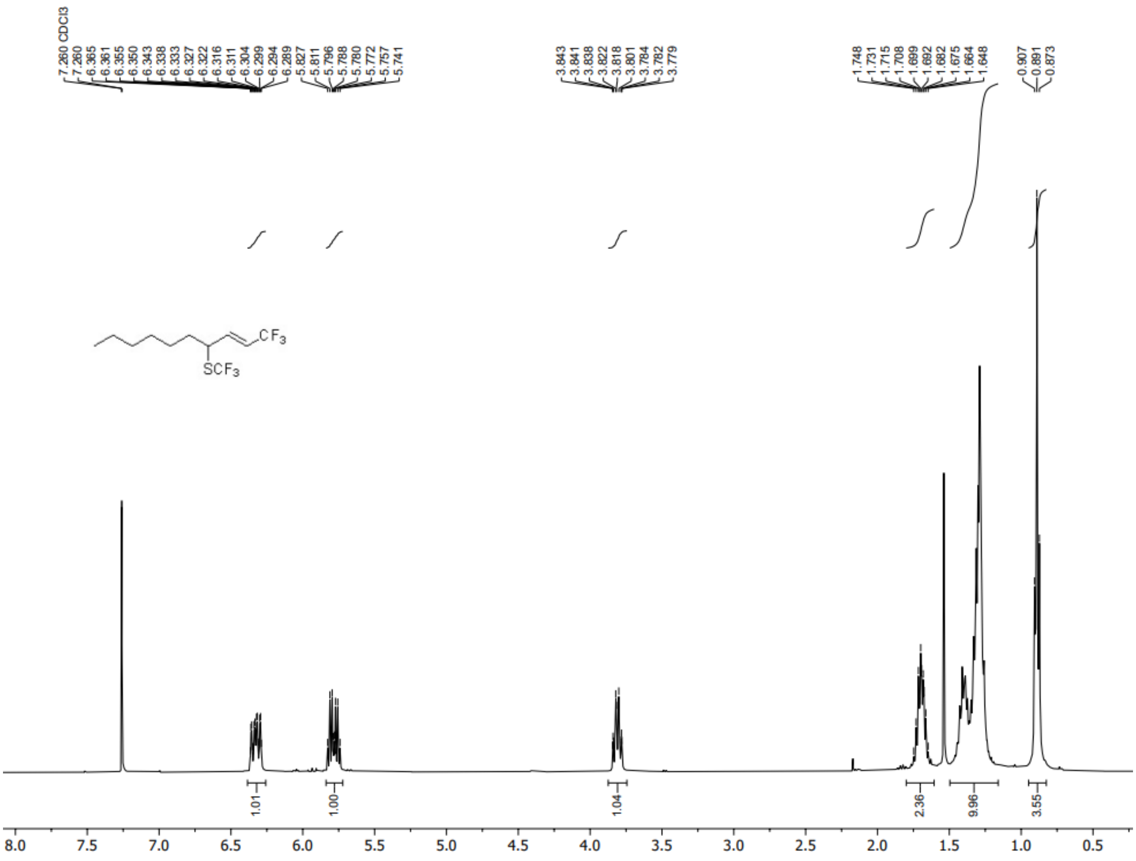

(*R*,*E*)-(1,1,1-Trifluorodec-2-en-4-yl)(trifluoromethyl)sulfane (**3a**) (^{13}C NMR)

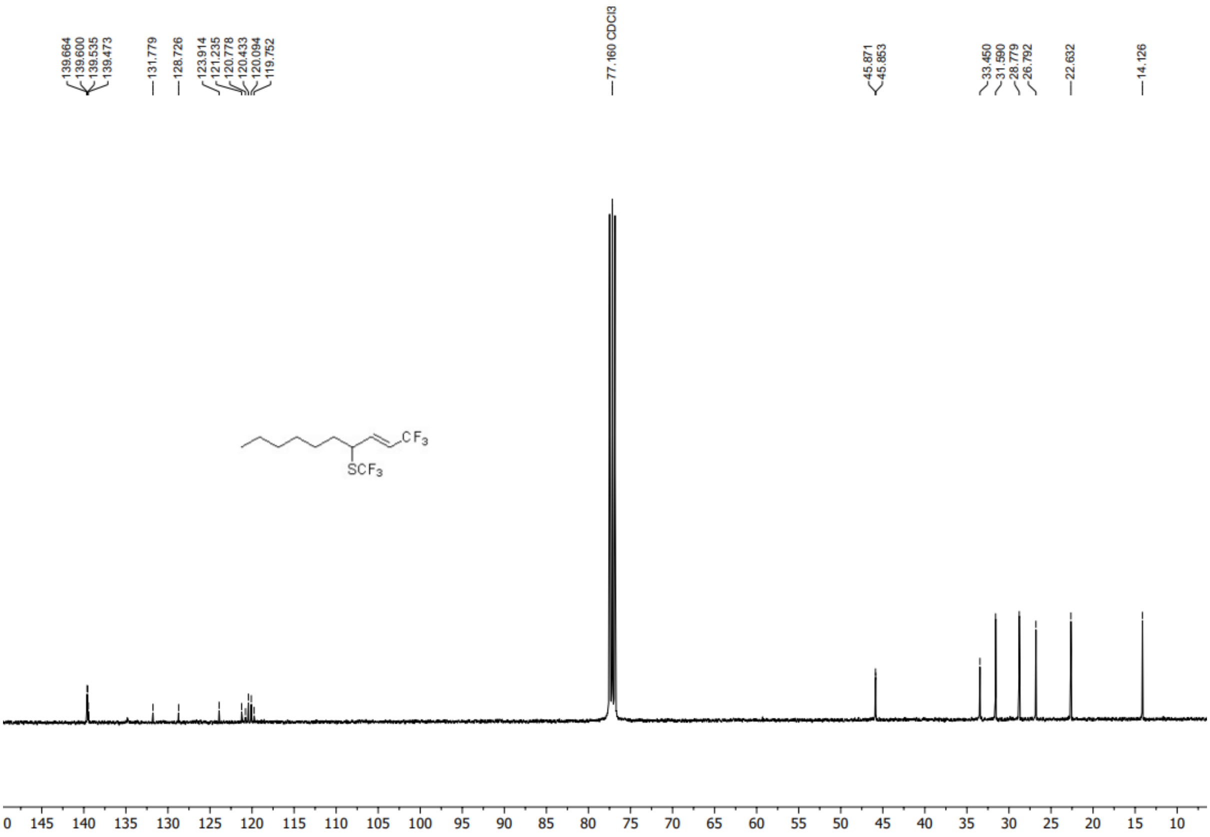

(*R*,*E*)-(1,1,1-Trifluorodec-2-en-4-yl)(trifluoromethyl)sulfane (**3a**) (^{19}F NMR)

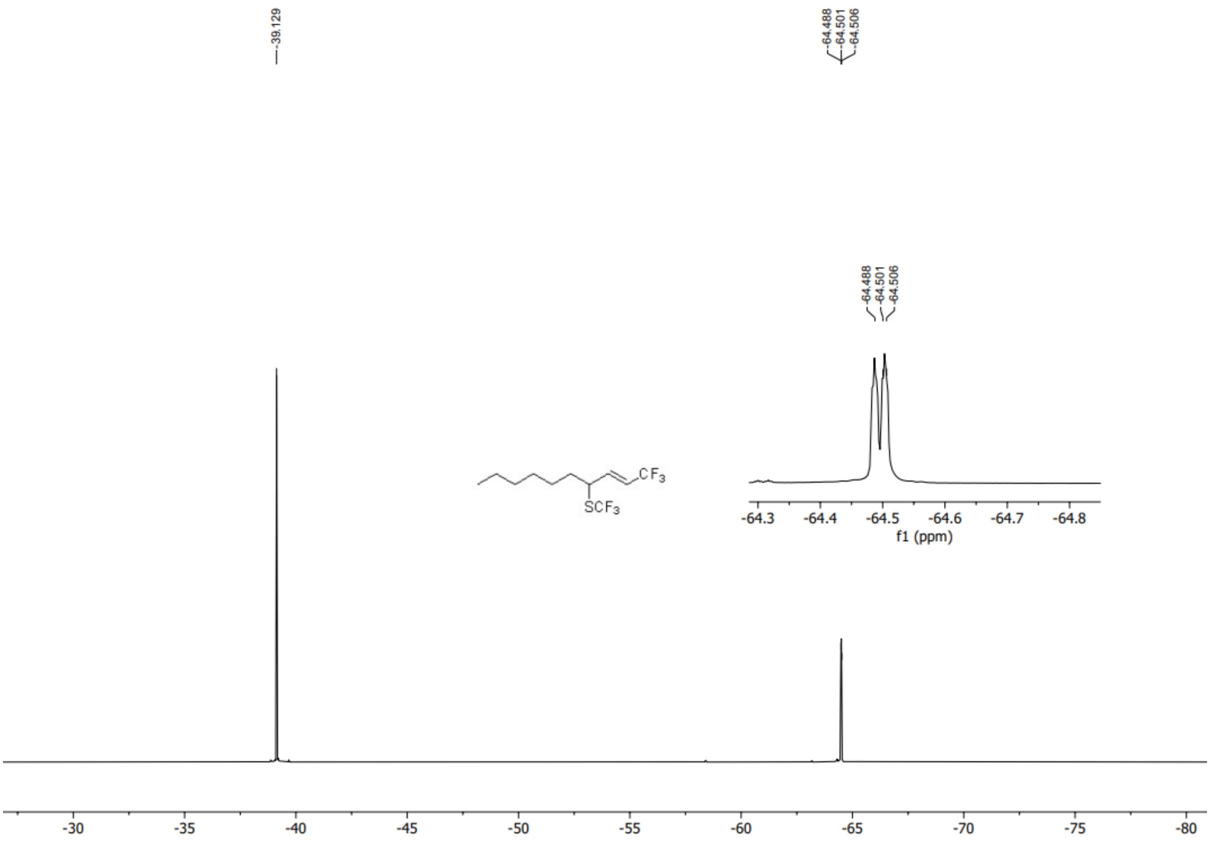

(*R*,*E*)-(5,5,5-Trifluoro-1-phenylpent-3-en-2-yl)(trifluoromethyl)sulfane (**3b**) (^{1}H NMR)

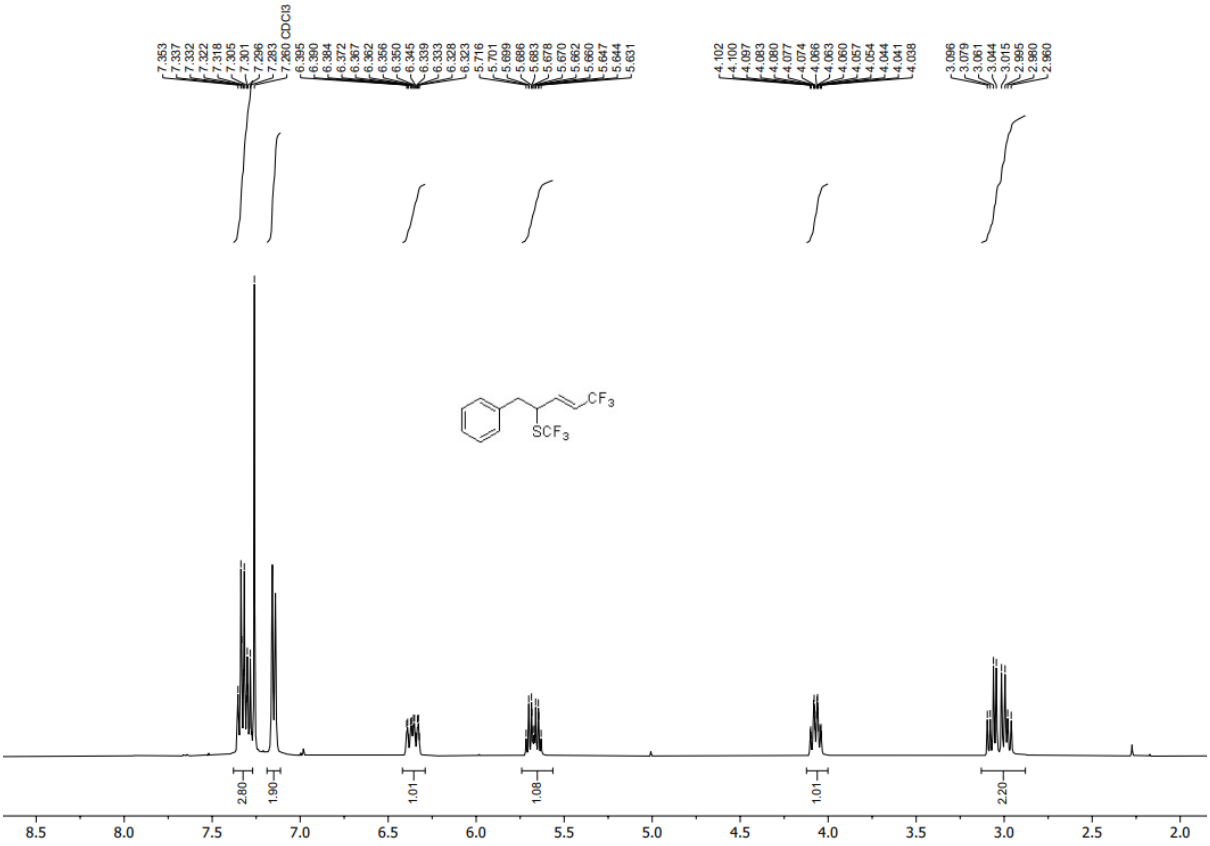

(*R*,*E*)-(5,5,5-Trifluoro-1-phenylpent-3-en-2-yl)(trifluoromethyl)sulfane (**3b**) (^{13}C NMR)

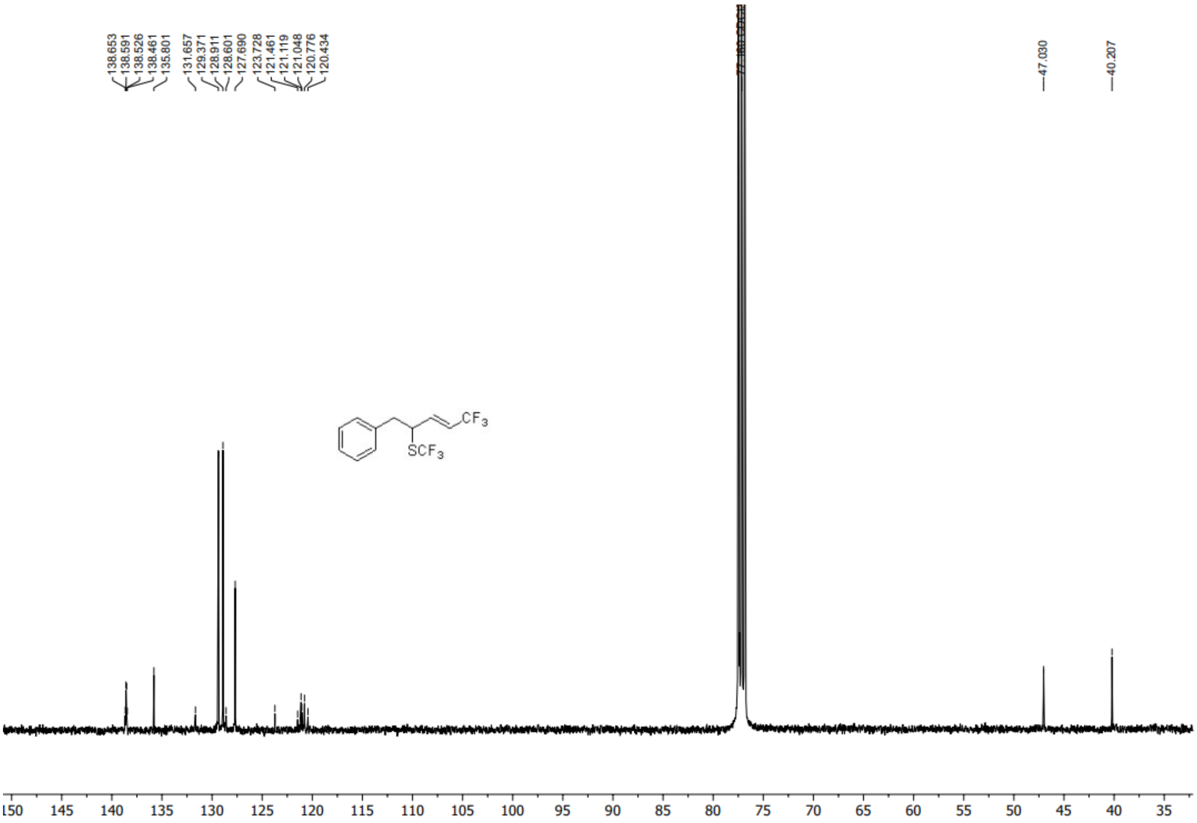

(*R*,*E*)-(5,5,5-Trifluoro-1-phenylpent-3-en-2-yl)(trifluoromethyl)sulfane (**3b**) (^{19F} NMR)

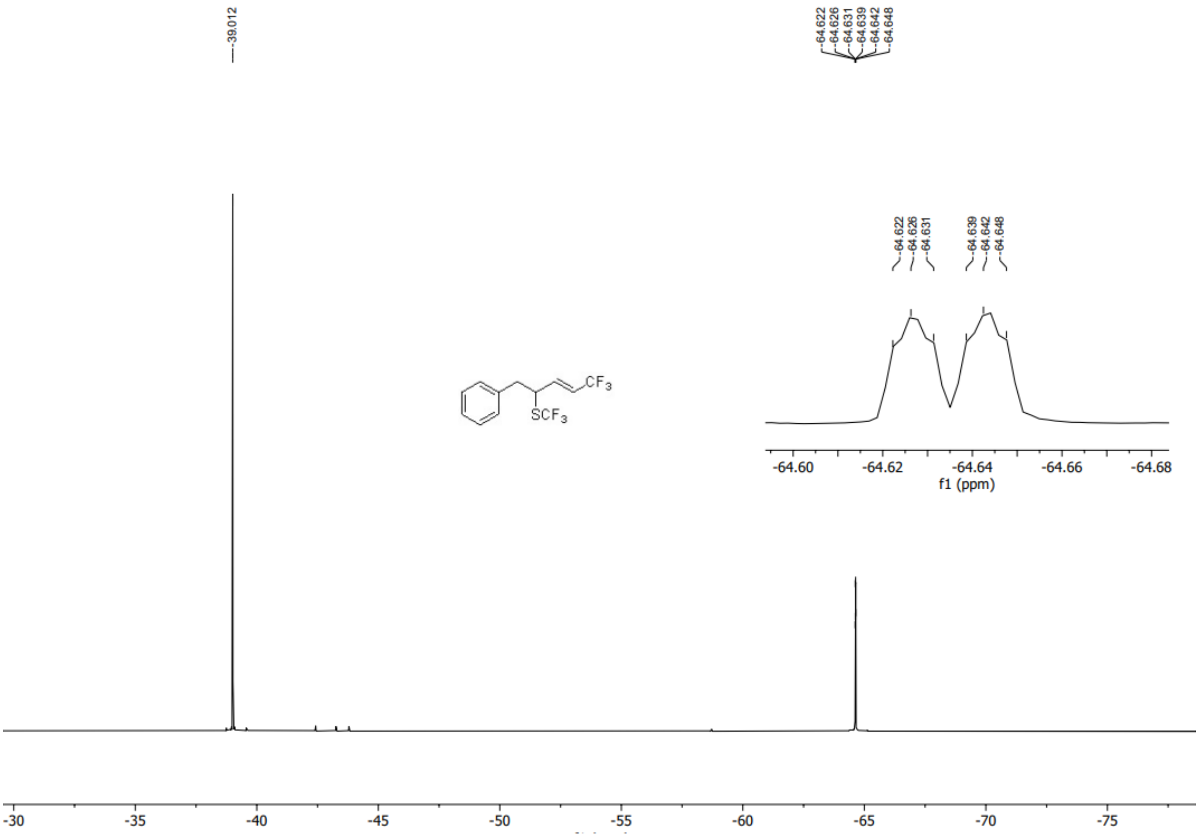

(*R*,*E*)-(8-Chloro-1,1,1-trifluorooct-2-en-4-yl)(trifluoromethyl)sulfane (**3c**) (^{1}H NMR)

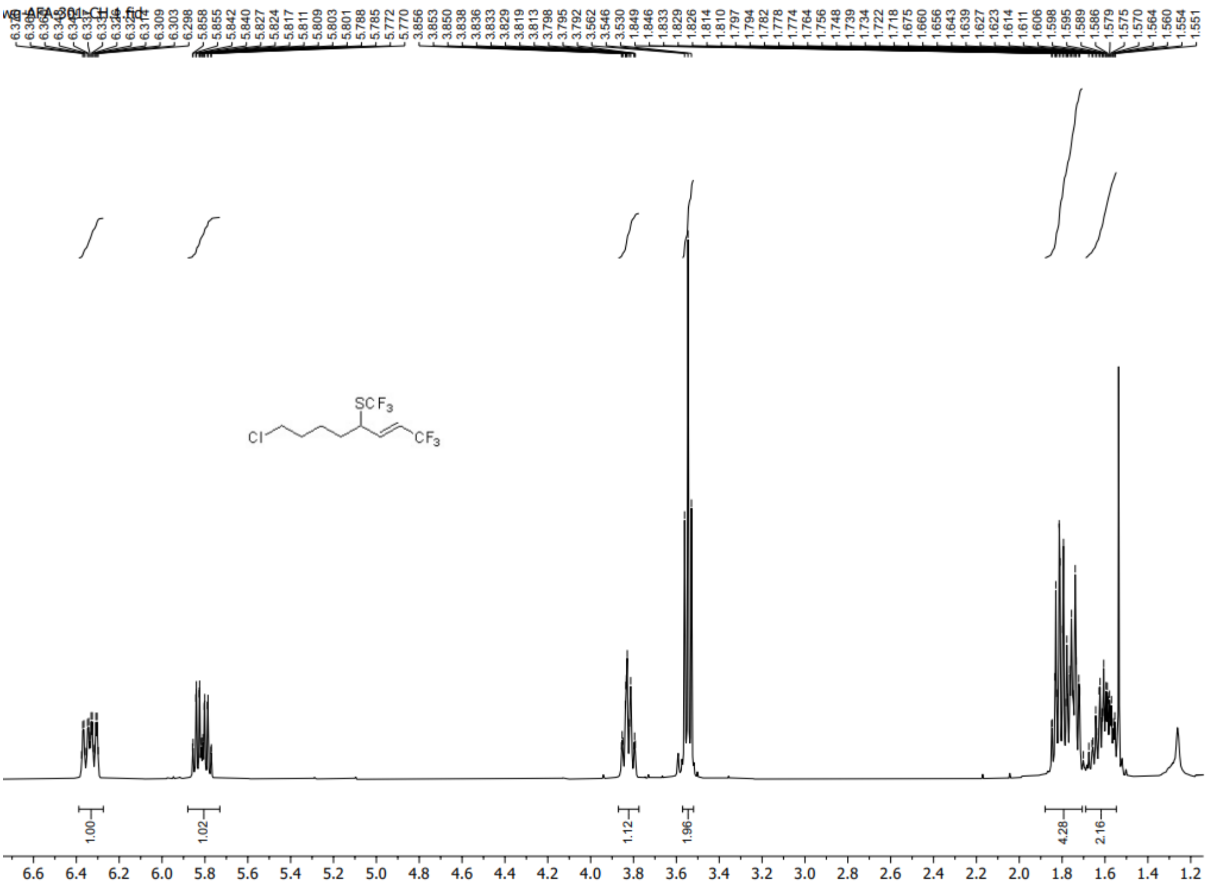

(*R*,*E*)-(8-Chloro-1,1,1-trifluorooct-2-en-4-yl)(trifluoromethyl)sulfane (**3c**) (^{13}C NMR)

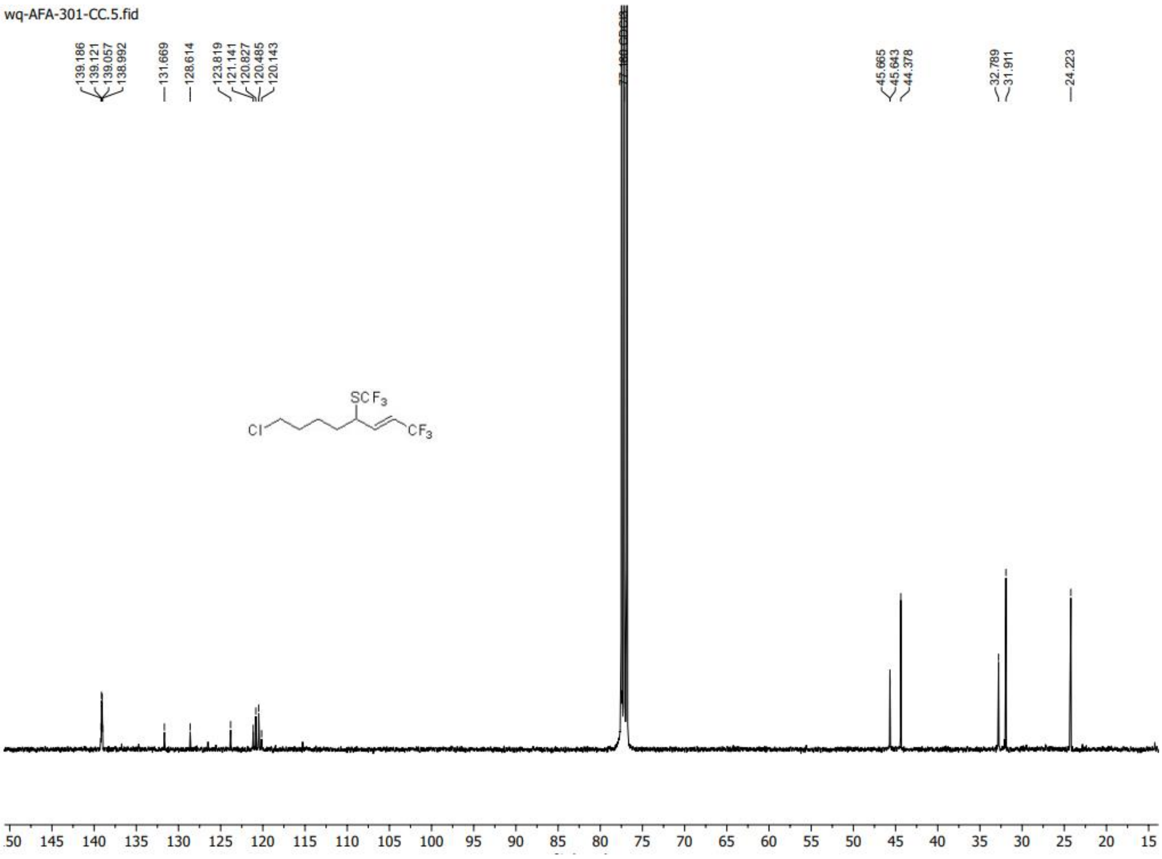

(*R*,*E*)-(8-Chloro-1,1,1-trifluorooct-2-en-4-yl)(trifluoromethyl)sulfane (**3c**) (^{19}F NMR)

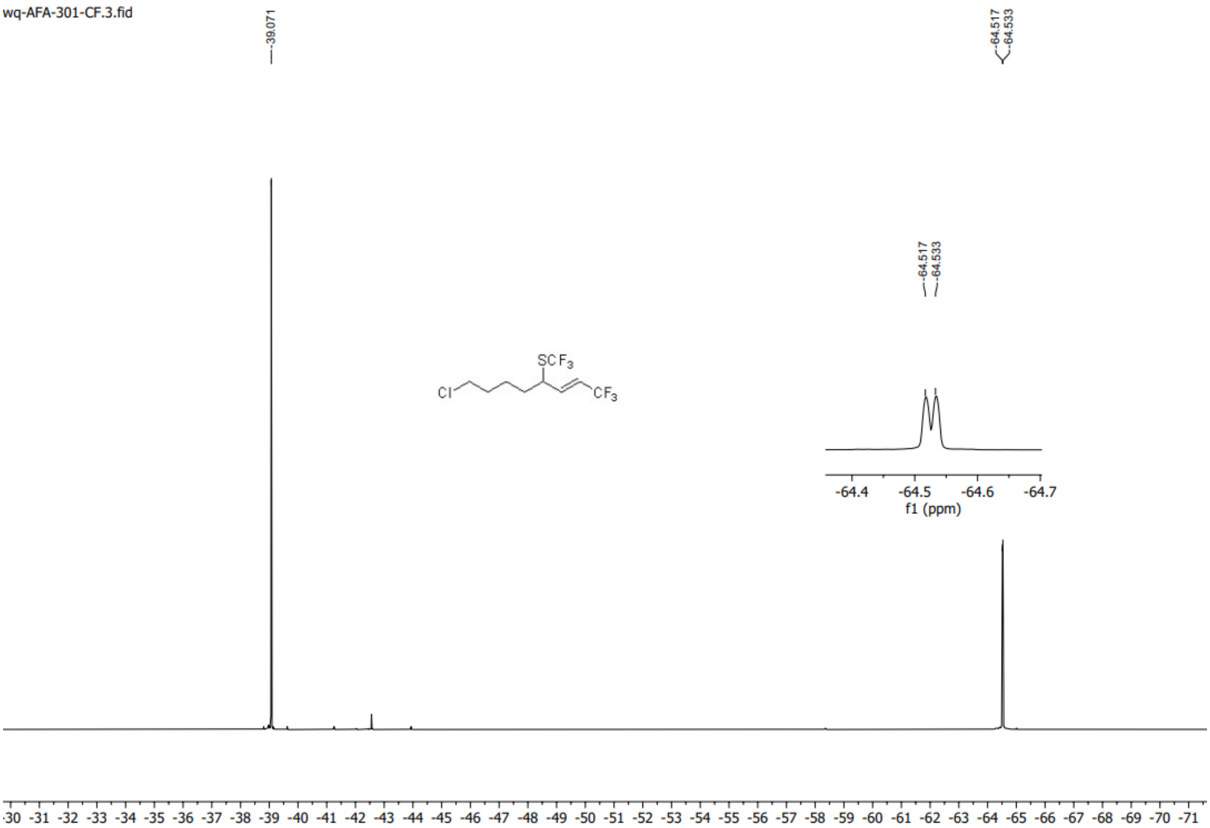

(*R*,*E*)-6,6,6-Trifluoro-3-((trifluoromethyl)thio)hex-4-en-1-yl 4-methylbenzenesulfonate (**3d**)

(^{1}H NMR)

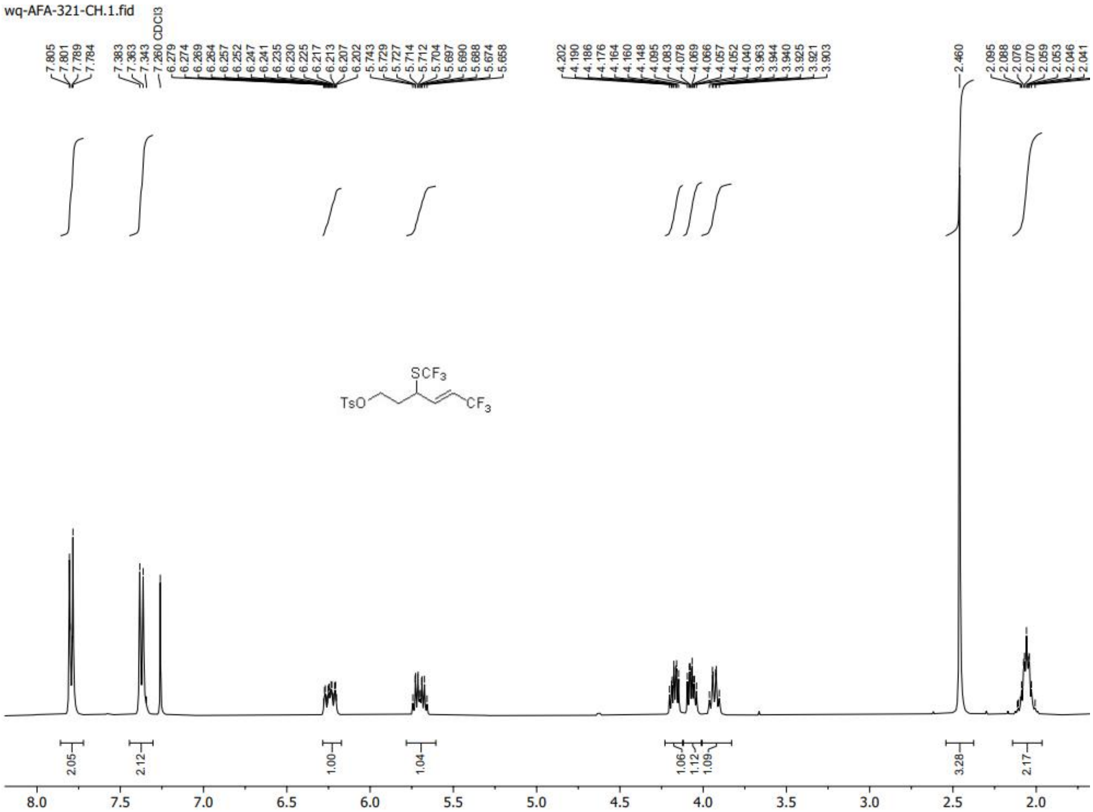

(*R*,*E*)-6,6,6-Trifluoro-3-((trifluoromethyl)thio)hex-4-en-1-yl 4-methylbenzenesulfonate (**3d**)
(^{13}C NMR)

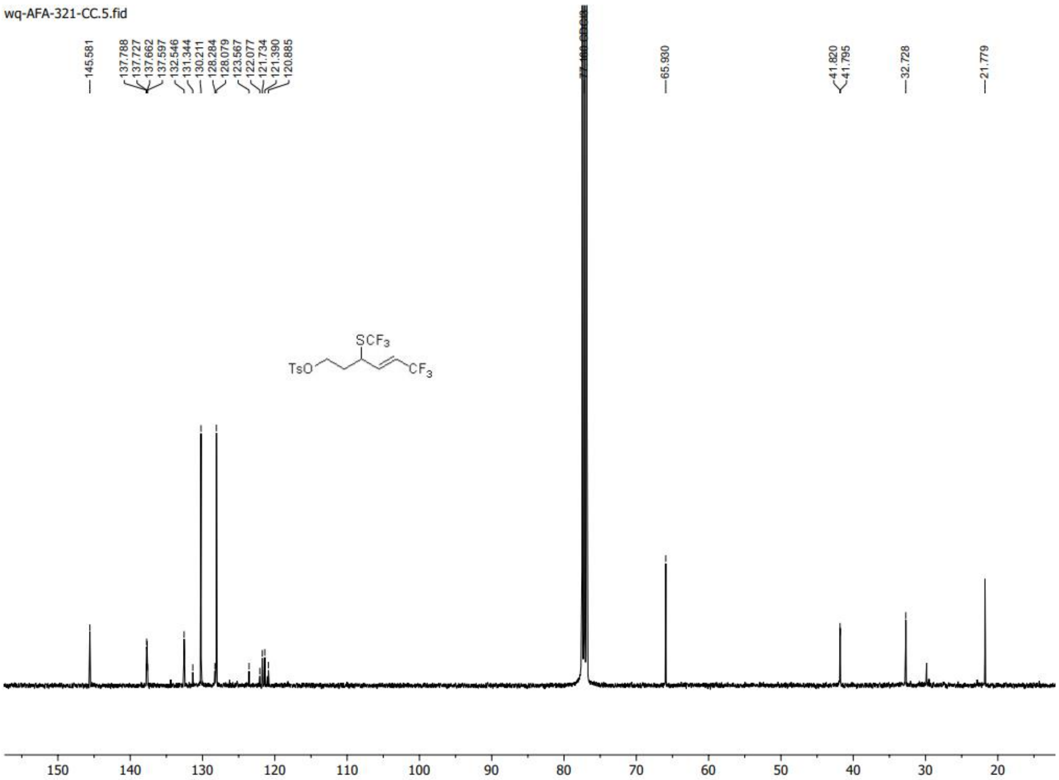

(*R*,*E*)-6,6,6-Trifluoro-3-((trifluoromethyl)thio)hex-4-en-1-yl 4-methylbenzenesulfonate (**3d**)
(^{19}F NMR)

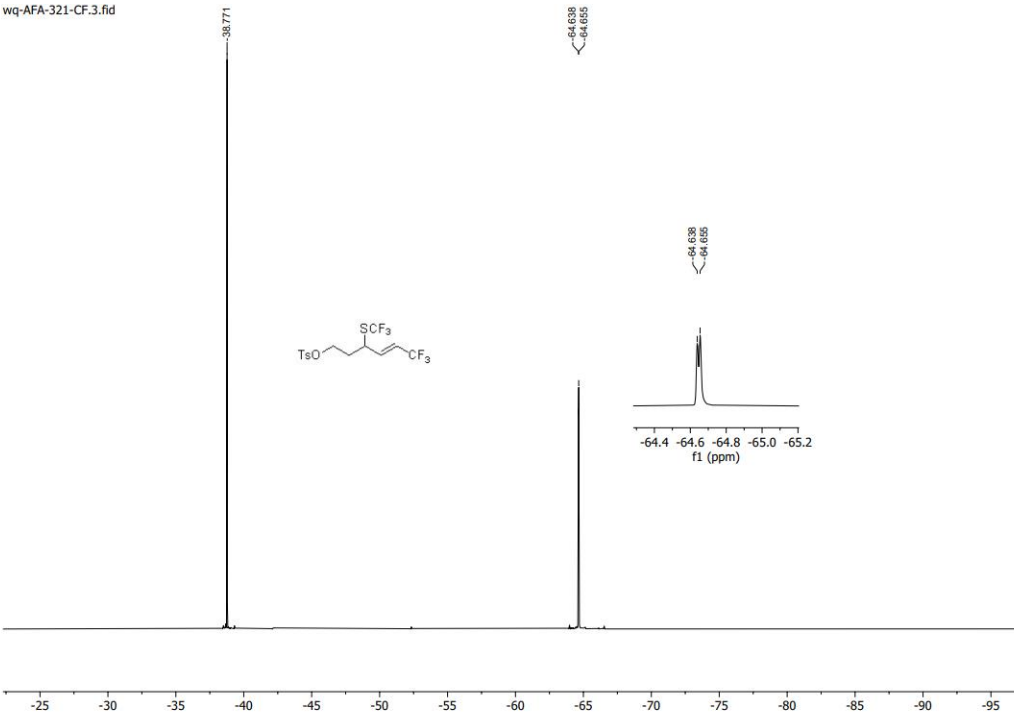

(*S*,*E*)-2-(5,5,5-Trifluoro-2-((trifluoromethyl)thio)pent-3-en-1-yl)isoindoline-1,3-dione (**3e**)

(^{1}H NMR)

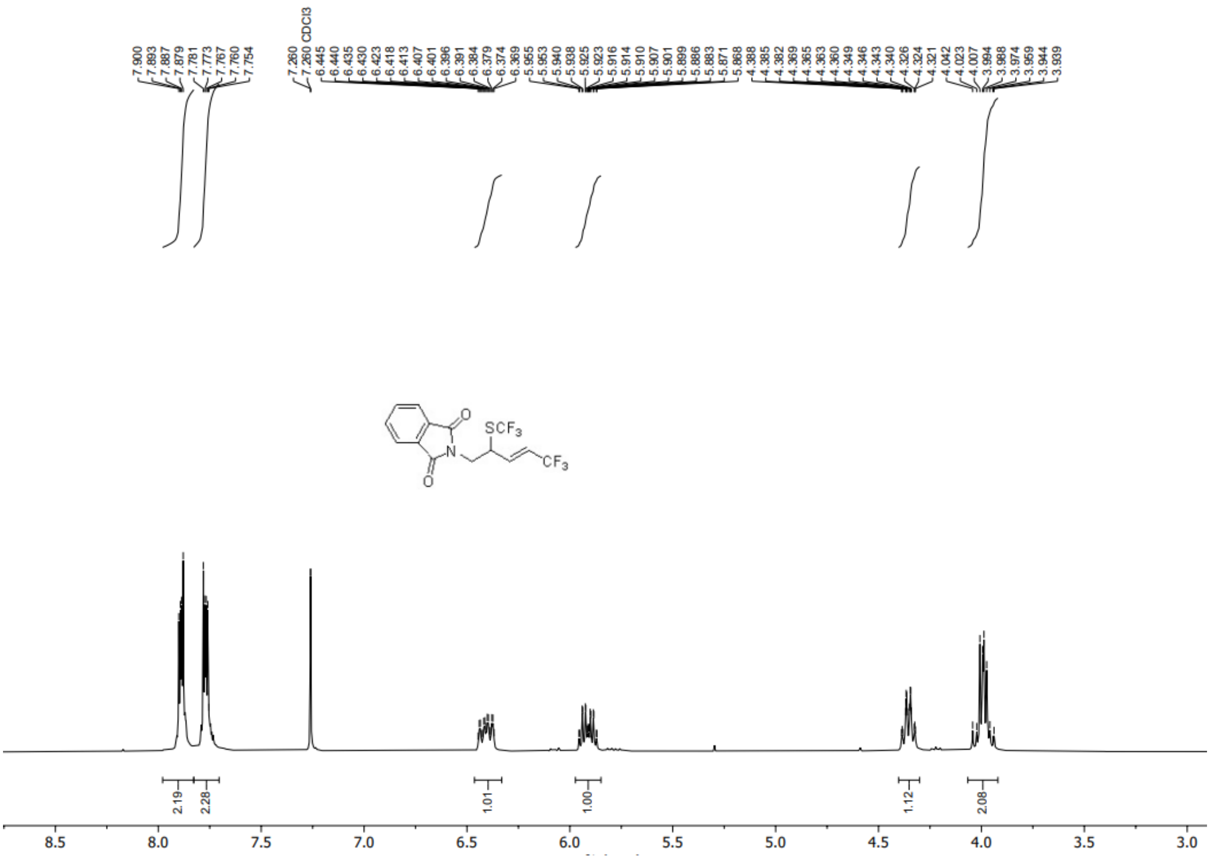

(*S*,*E*)-2-(5,5,5-Trifluoro-2-((trifluoromethyl)thio)pent-3-en-1-yl)isoindoline-1,3-dione (**3e**)

(^{13}C NMR)

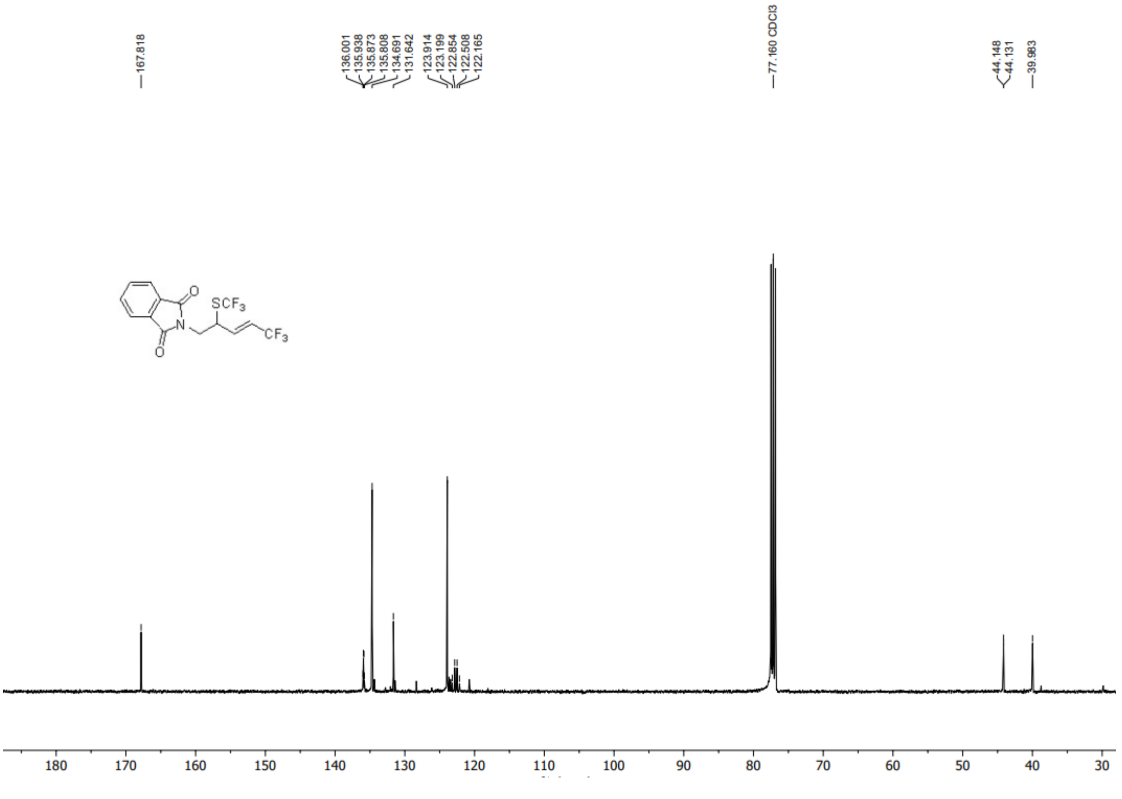

(*S*,*E*)-2-(5,5,5-Trifluoro-2-((trifluoromethyl)thio)pent-3-en-1-yl)isoindoline-1,3-dione (**3e**) (^{19}F NMR)

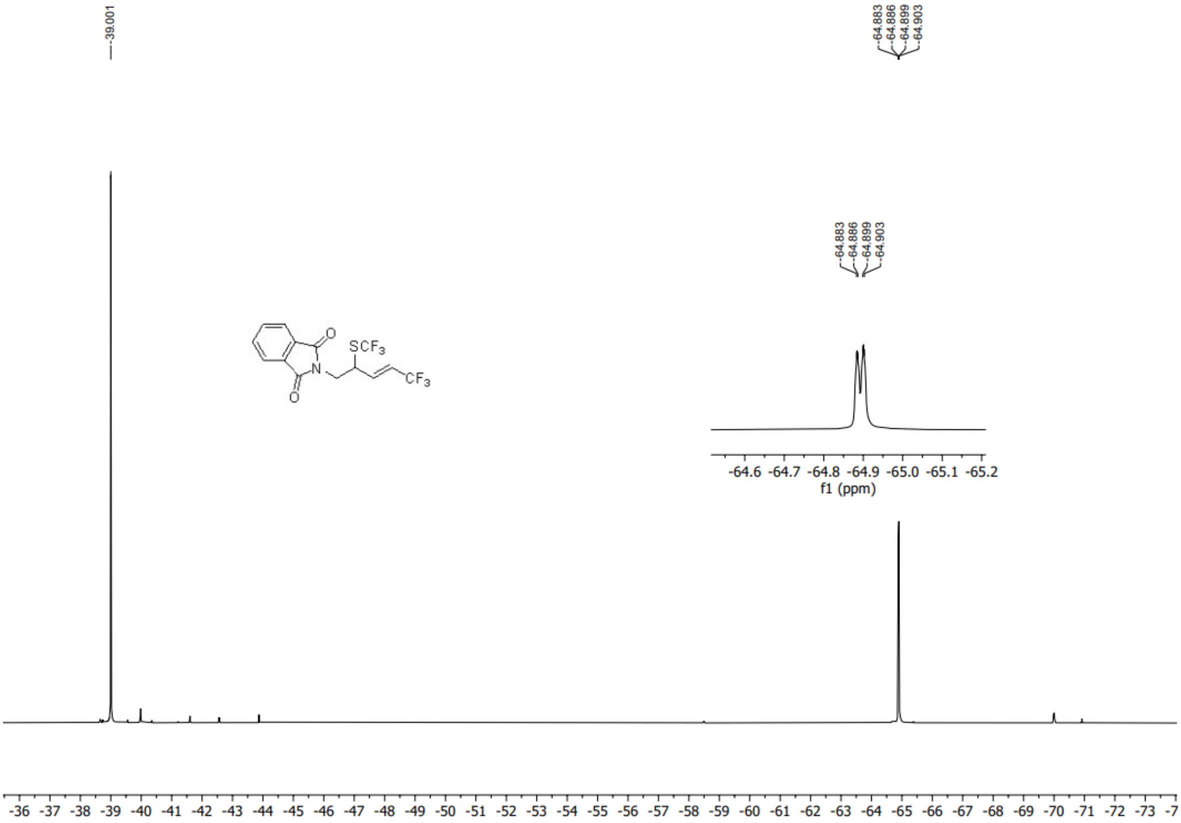

(*R*,*E*)-(1-Cyclohexyl-4,4,4-trifluorobut-2-en-1-yl)(trifluoromethyl)sulfane (**3f**) (^{1}H NMR)

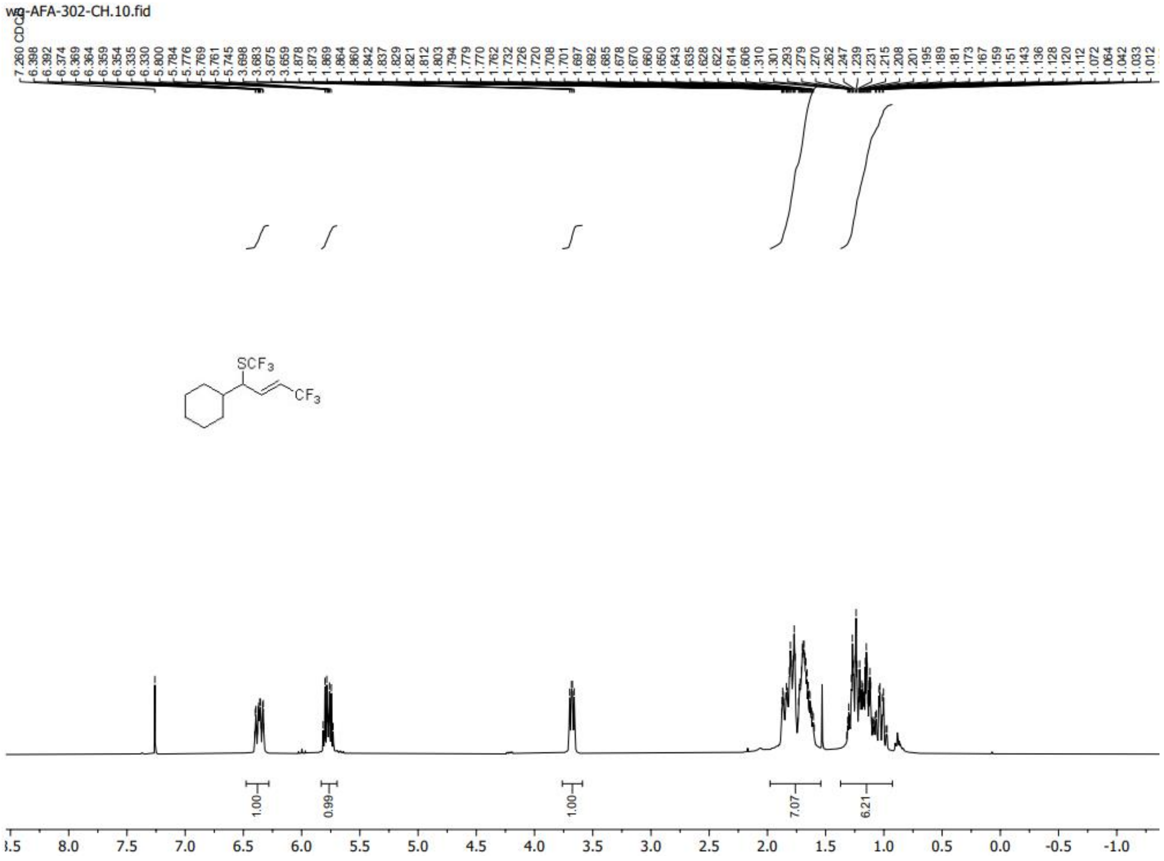

(*R*,*E*)-(1-Cyclohexyl-4,4,4-trifluorobut-2-en-1-yl)(trifluoromethyl)sulfane (**3f**) (^{13}C NMR)

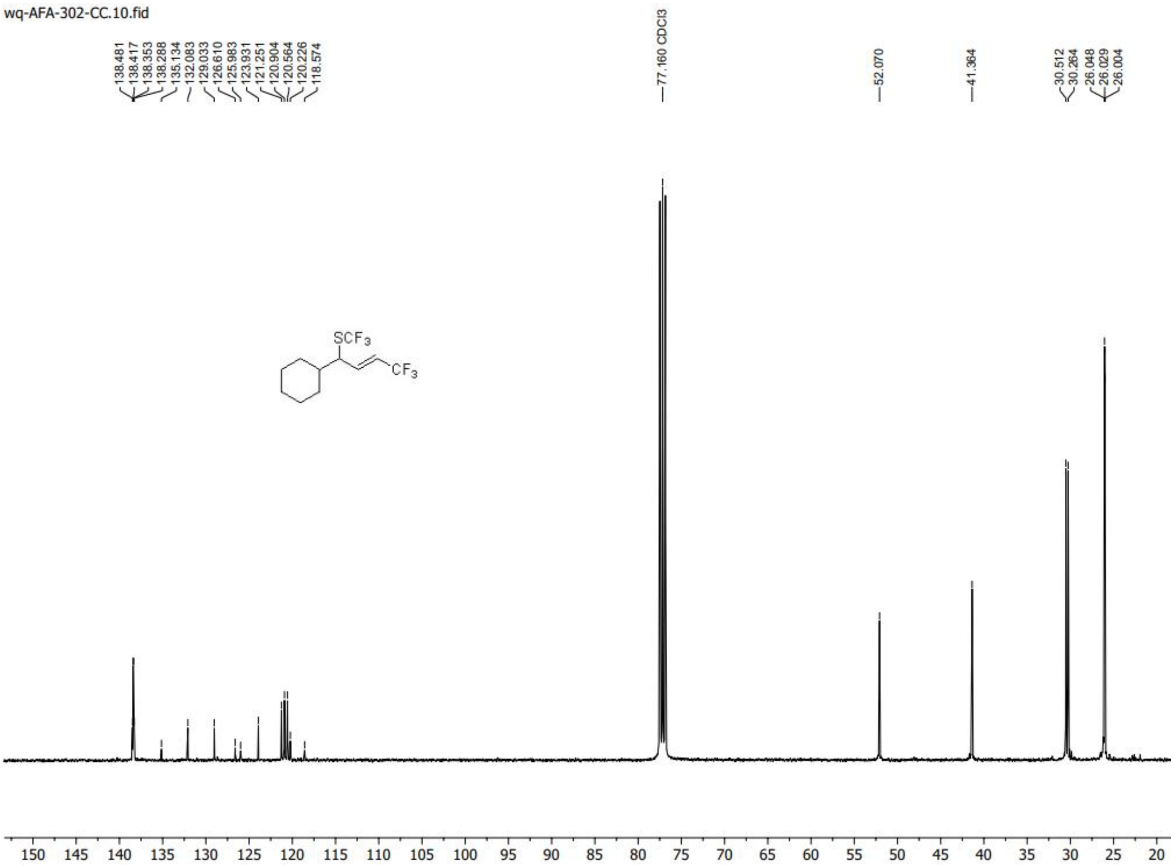

(*R*,*E*)-(1-Cyclohexyl-4,4,4-trifluorobut-2-en-1-yl)(trifluoromethyl)sulfane (**3f**) (^{19}F NMR)

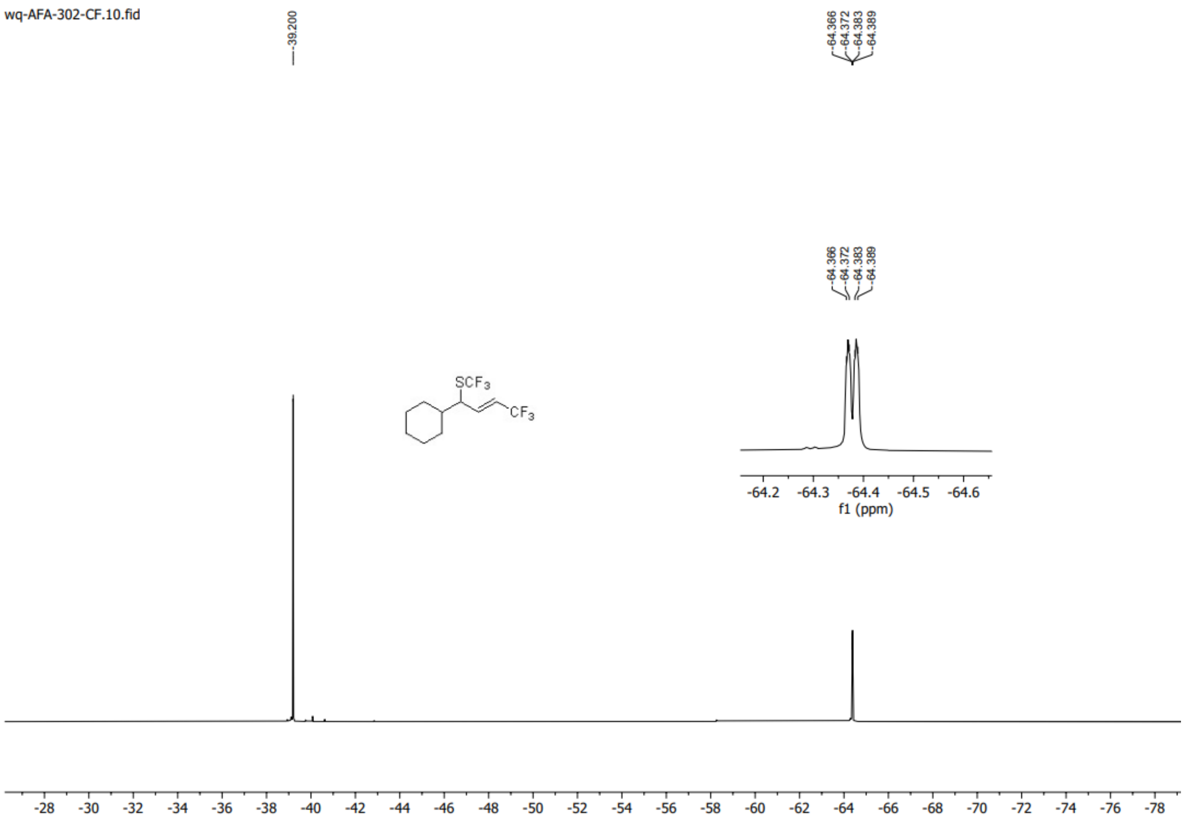

(*S*,*E*)-(4,4,4-Trifluoro-1-phenylbut-2-en-1-yl)(trifluoromethyl)sulfane (**3g**) (^{1}H NMR)

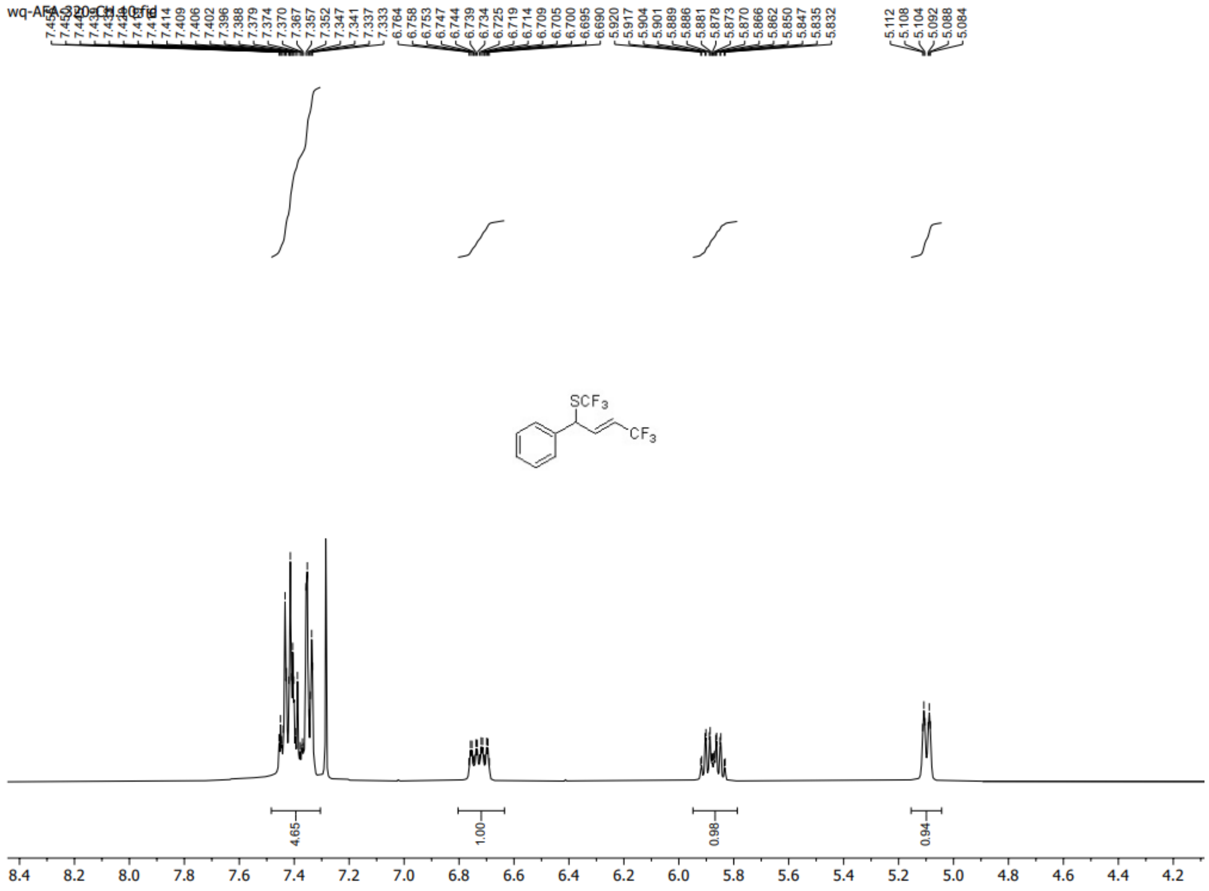

(*S*,*E*)-(4,4,4-Trifluoro-1-phenylbut-2-en-1-yl)(trifluoromethyl)sulfane (**3g**) (^{13}C NMR)

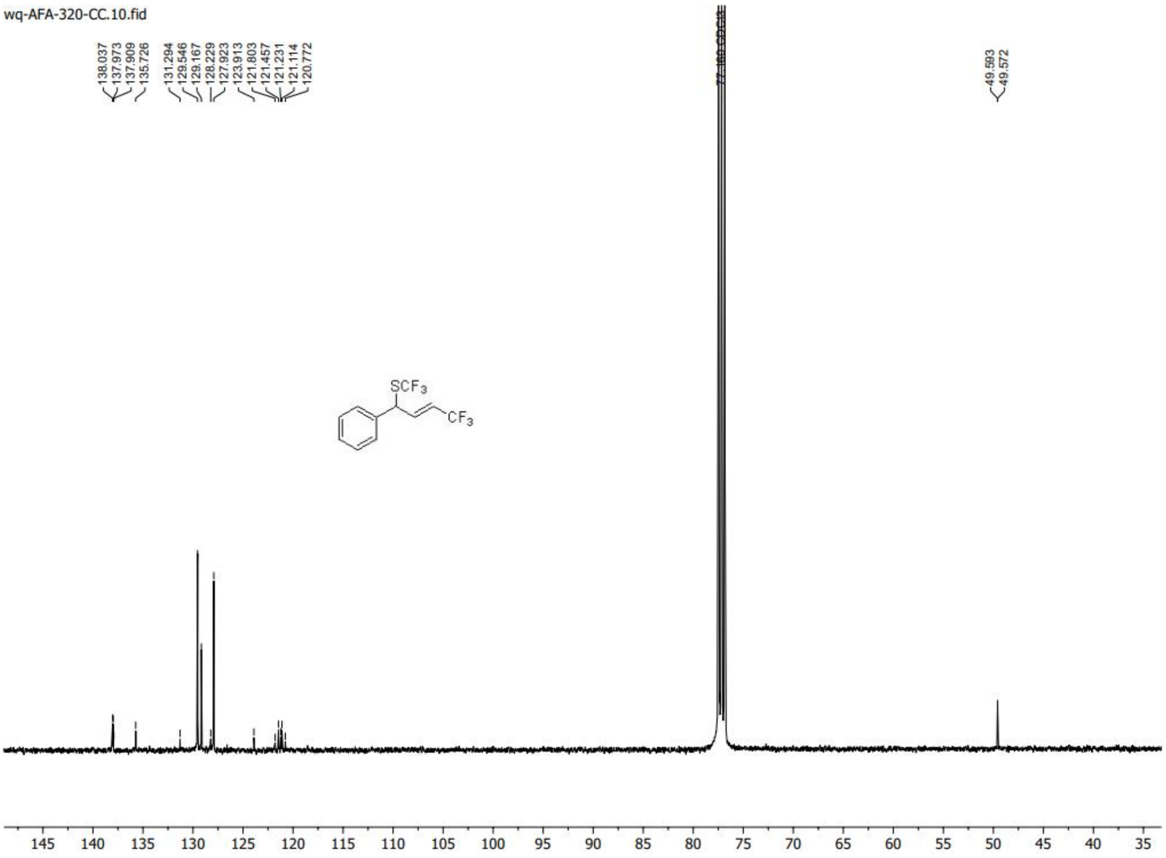

(*S*,*E*)-(4,4,4-Trifluoro-1-phenylbut-2-en-1-yl)(trifluoromethyl)sulfane (**3g**) (^{19}F NMR)

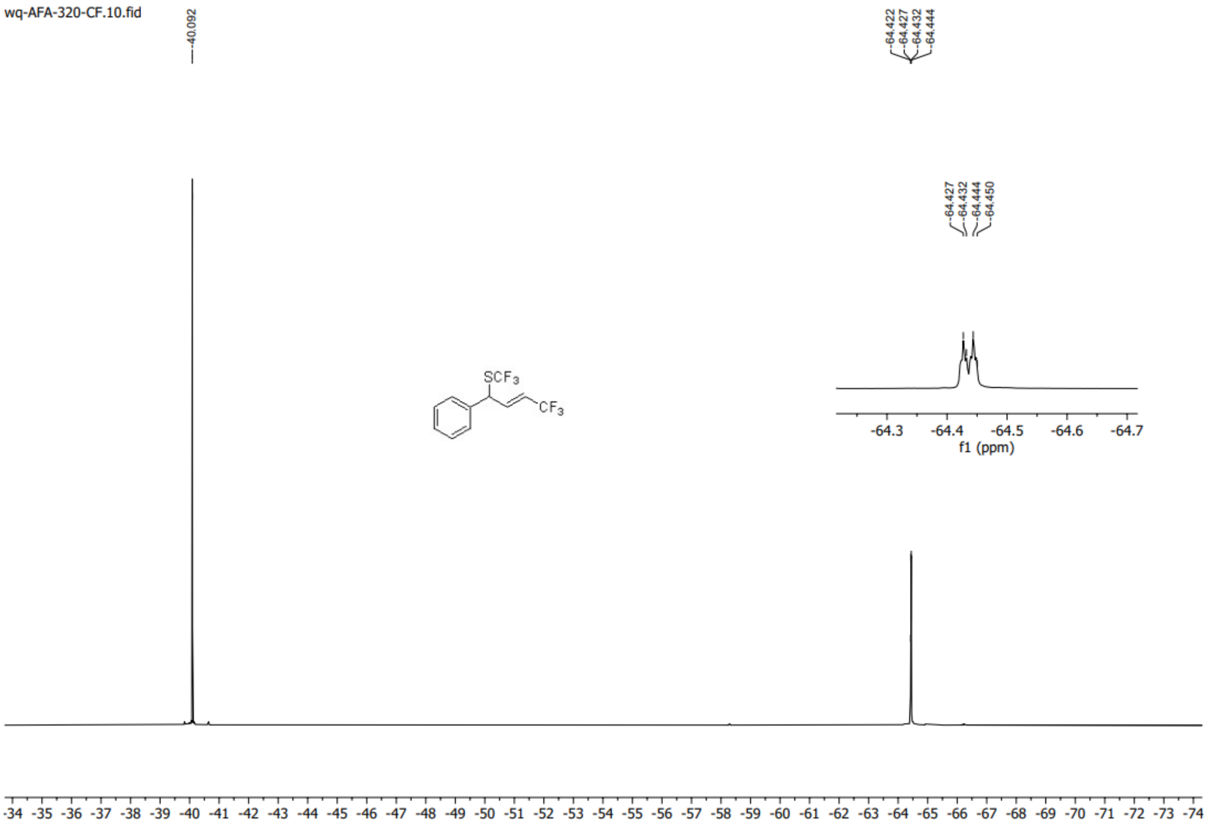

(*S*,*E*)-(6,6,6-trifluoro-2,2-dimethylhex-4-en-3-yl)(trifluoromethyl)sulfane (**3h**) (^{1}H NMR)

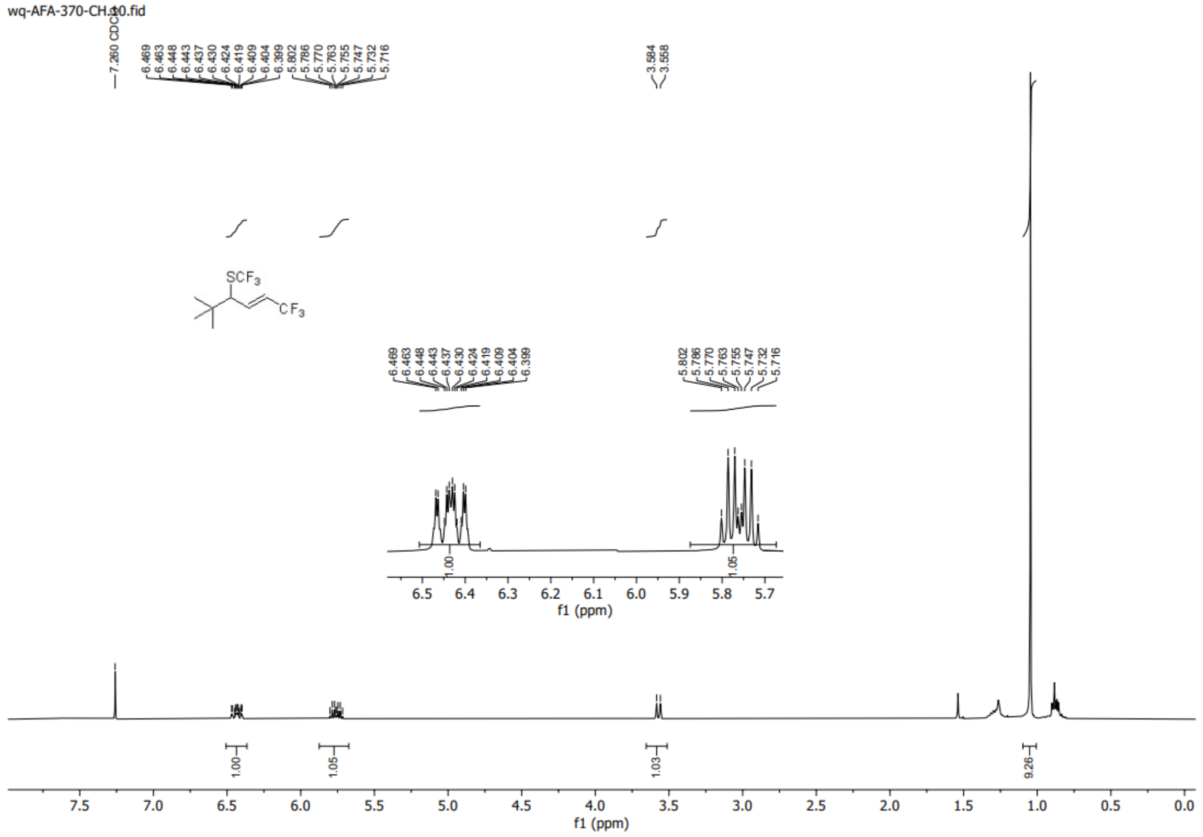

(*S*,*E*)-(6,6,6-trifluoro-2,2-dimethylhex-4-en-3-yl)(trifluoromethyl)sulfane (**3h**) (^{13}C NMR)

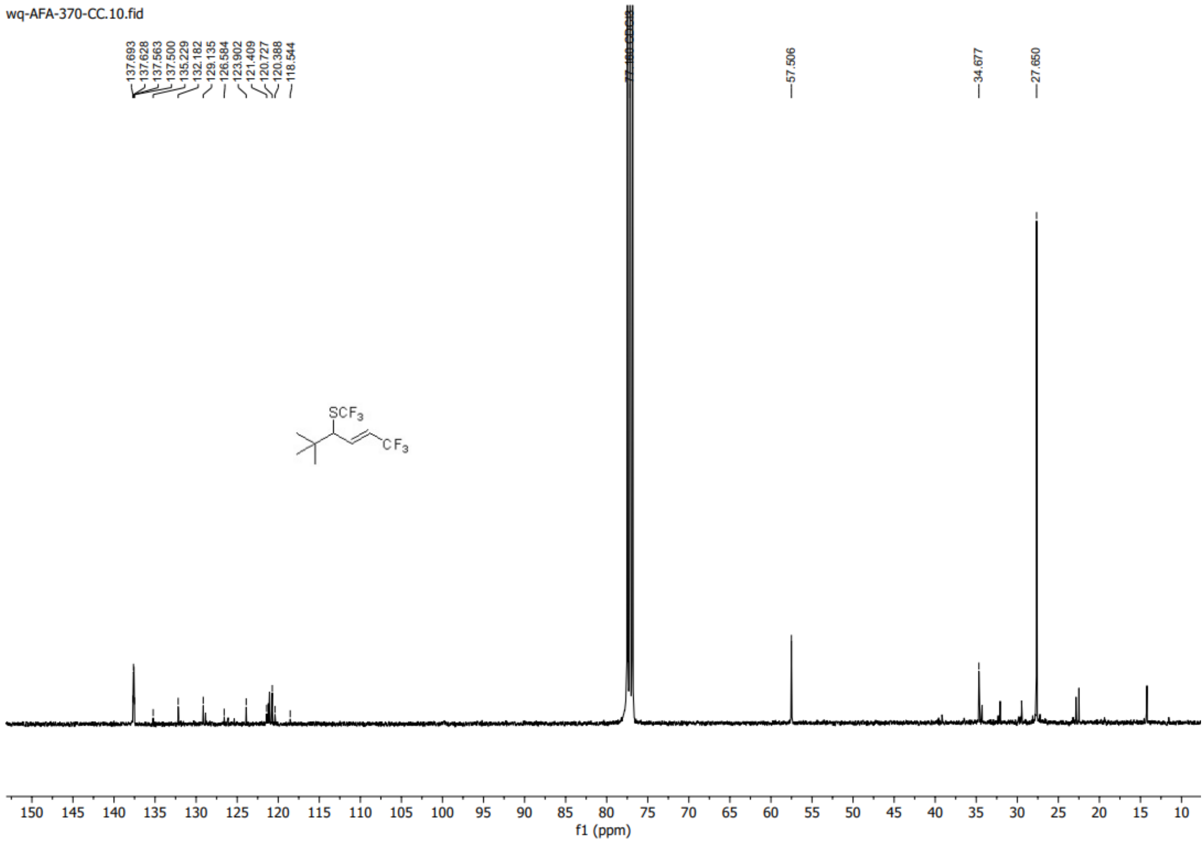

(*S*,*E*)-(6,6,6-trifluoro-2,2-dimethylhex-4-en-3-yl)(trifluoromethyl)sulfane (**3h**) (^{19}F NMR)

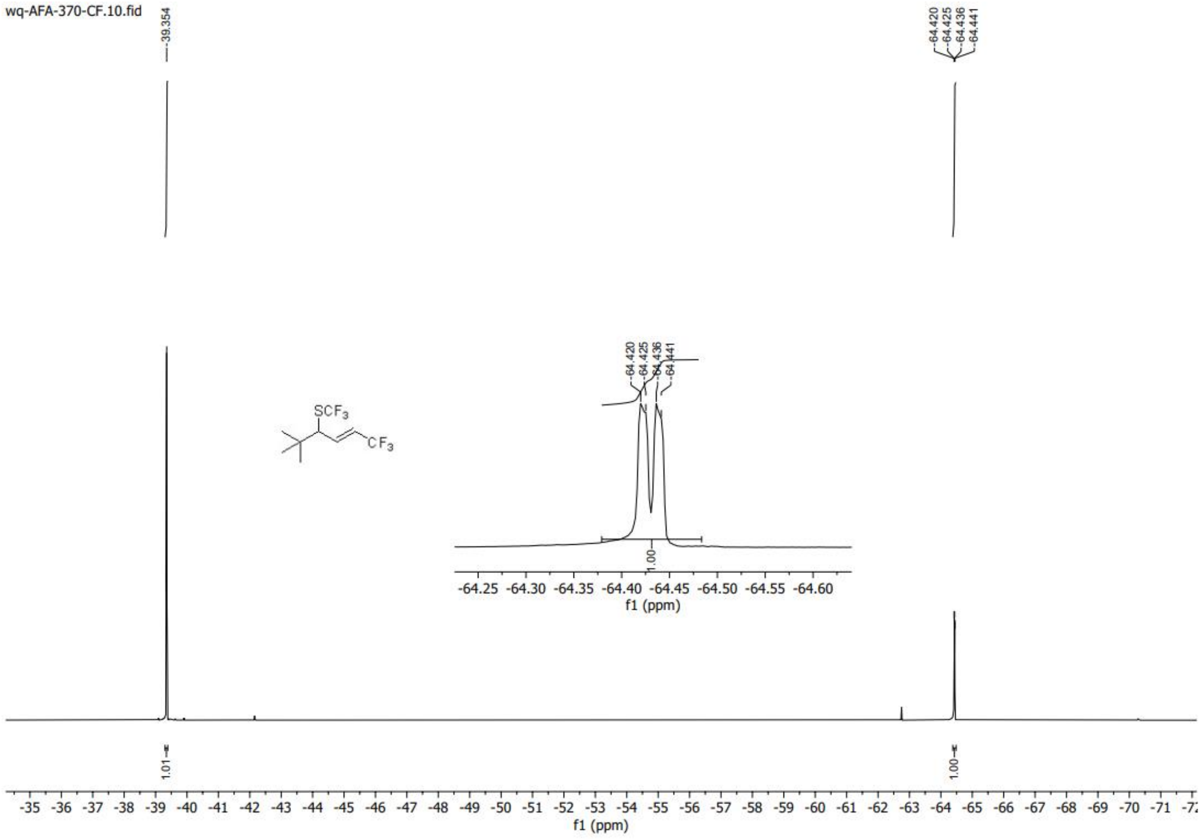

Ethyl (*R*,*E*)-2,2-difluoro-2-((1,1,1-trifluorodec-2-en-4-yl)thio)acetate (**4a**) (^{1}H NMR)

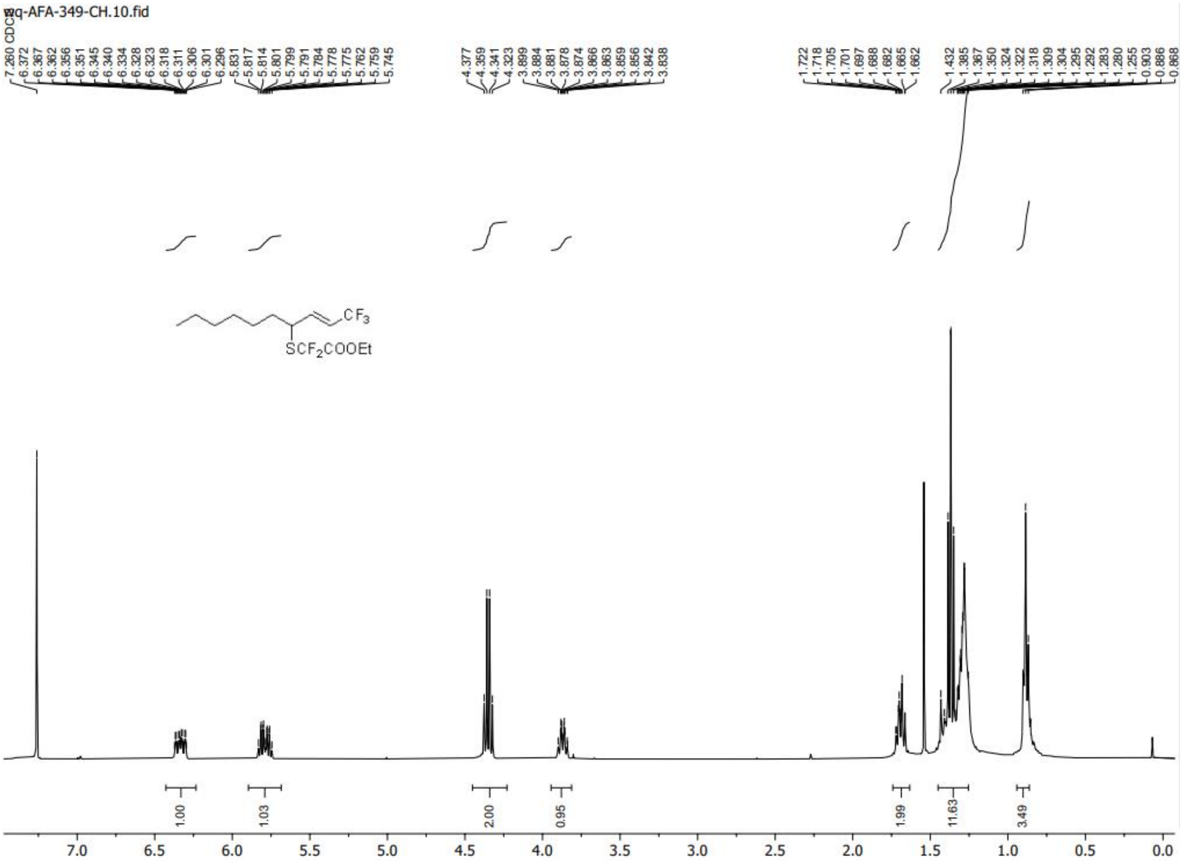

Ethyl (*R*,*E*)-2,2-difluoro-2-((1,1,1-trifluorodec-2-en-4-yl)thio)acetate (**4a**) (^{13}C NMR)

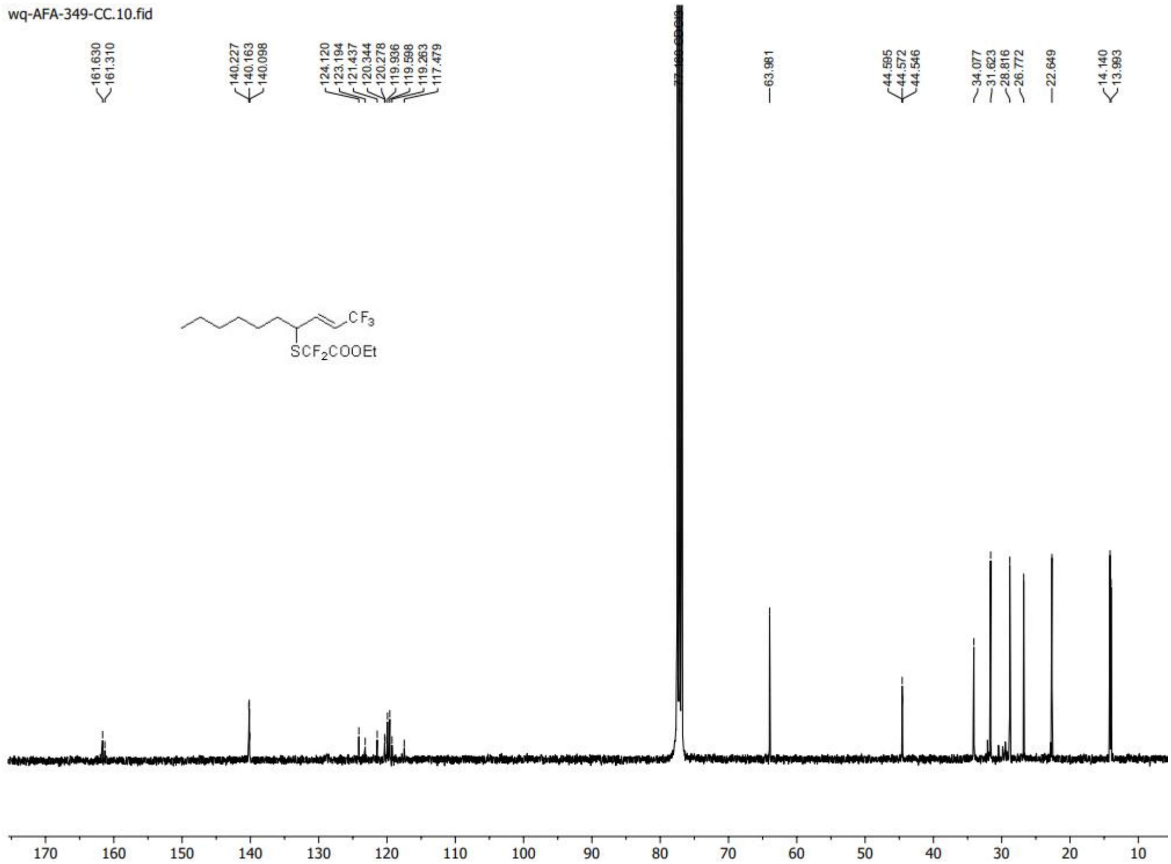

Ethyl (*R*,*E*)-2,2-difluoro-2-((1,1,1-trifluorodec-2-en-4-yl)thio)acetate (**4a**) (^{19}F NMR)

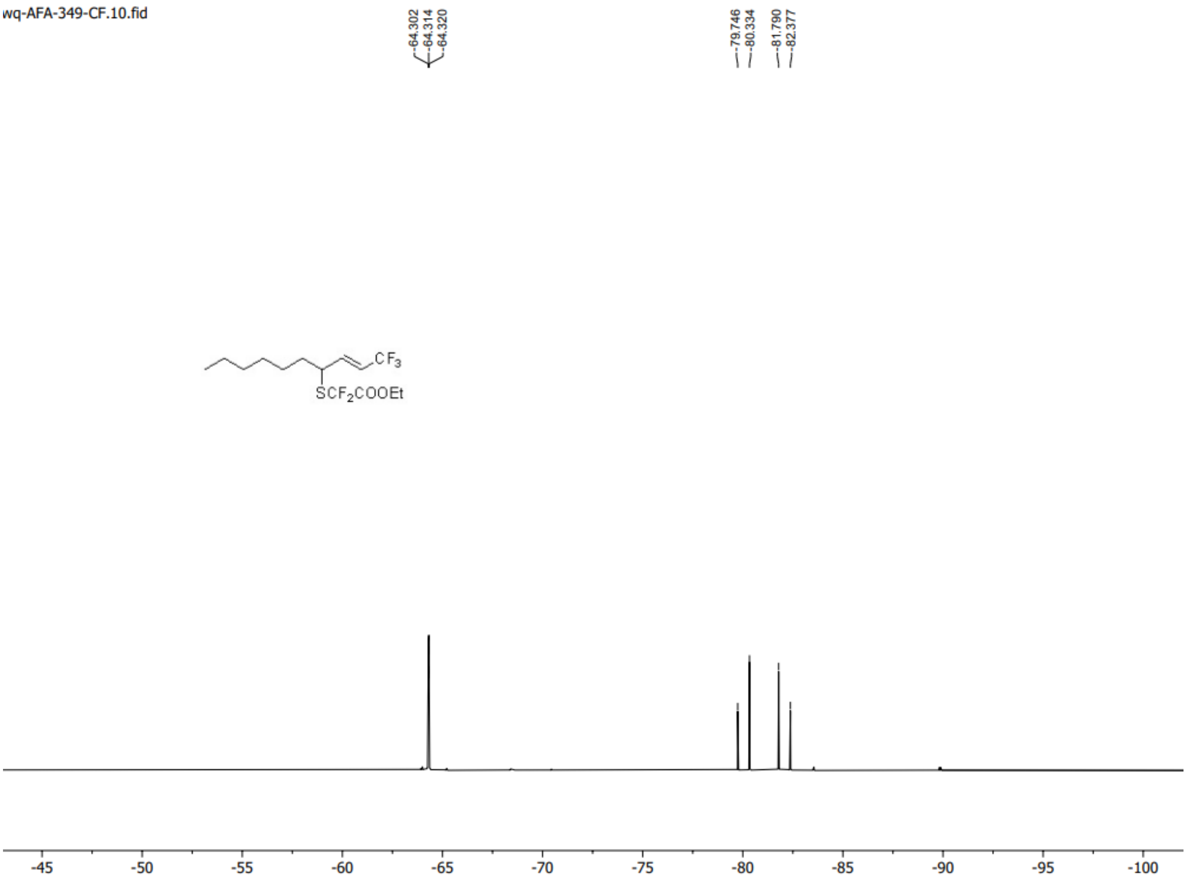

Ethyl (*R*,*E*)-2,2-difluoro-2-((5,5,5-trifluoro-1-phenylpent-3-en-2-yl)thio)acetate (**4b**) (^{1}H NMR)

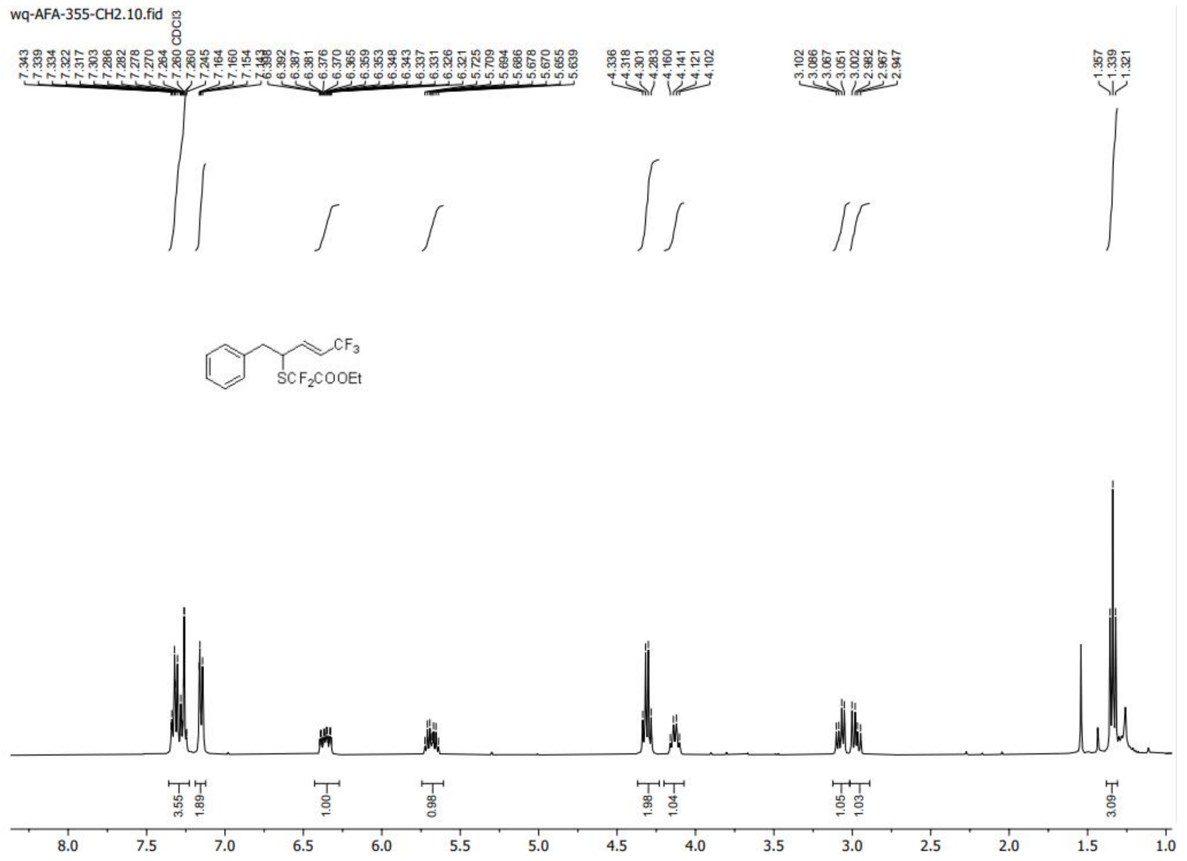

Ethyl (*R*,*E*)-2,2-difluoro-2-((5,5,5-trifluoro-1-phenylpent-3-en-2-yl)thio)acetate (**4b**) (^{13}C NMR)

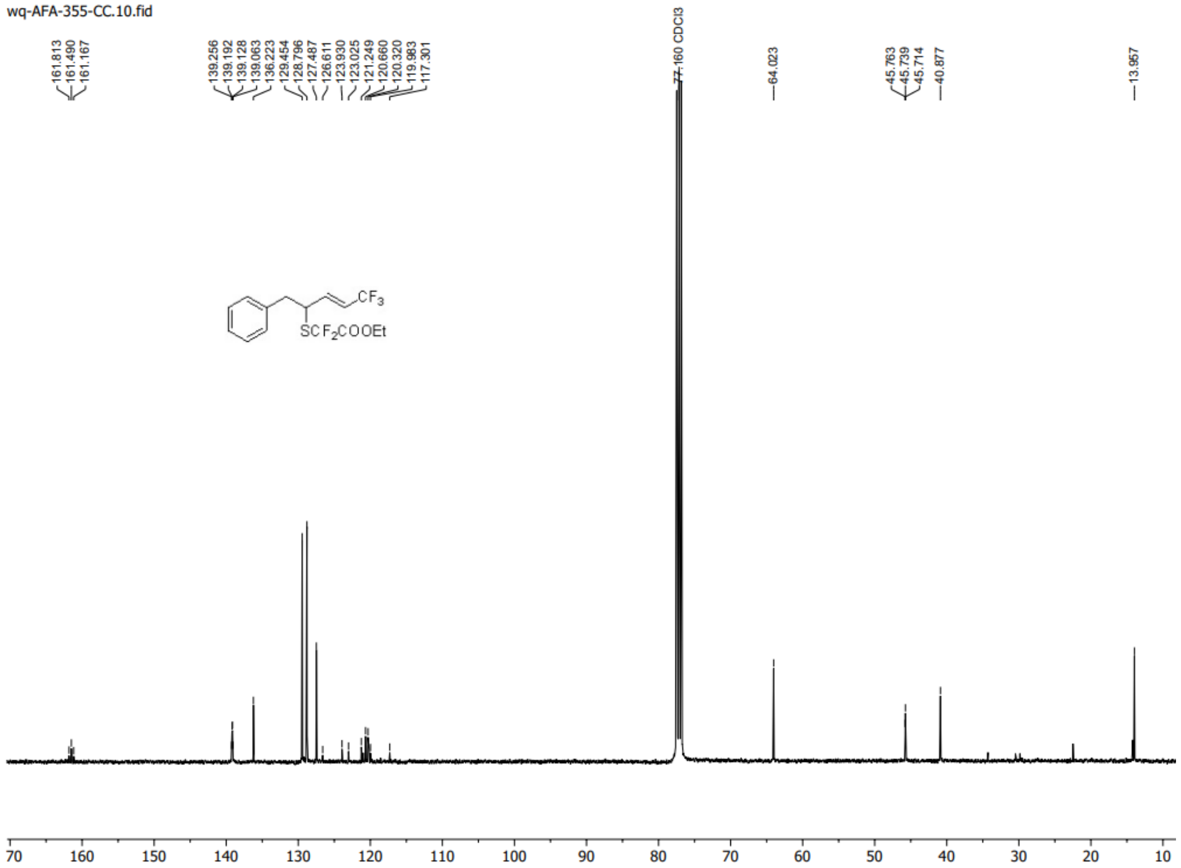

Ethyl (*R*,*E*)-2,2-difluoro-2-((5,5,5-trifluoro-1-phenylpent-3-en-2-yl)thio)acetate (**4b**) (^{19}F NMR)

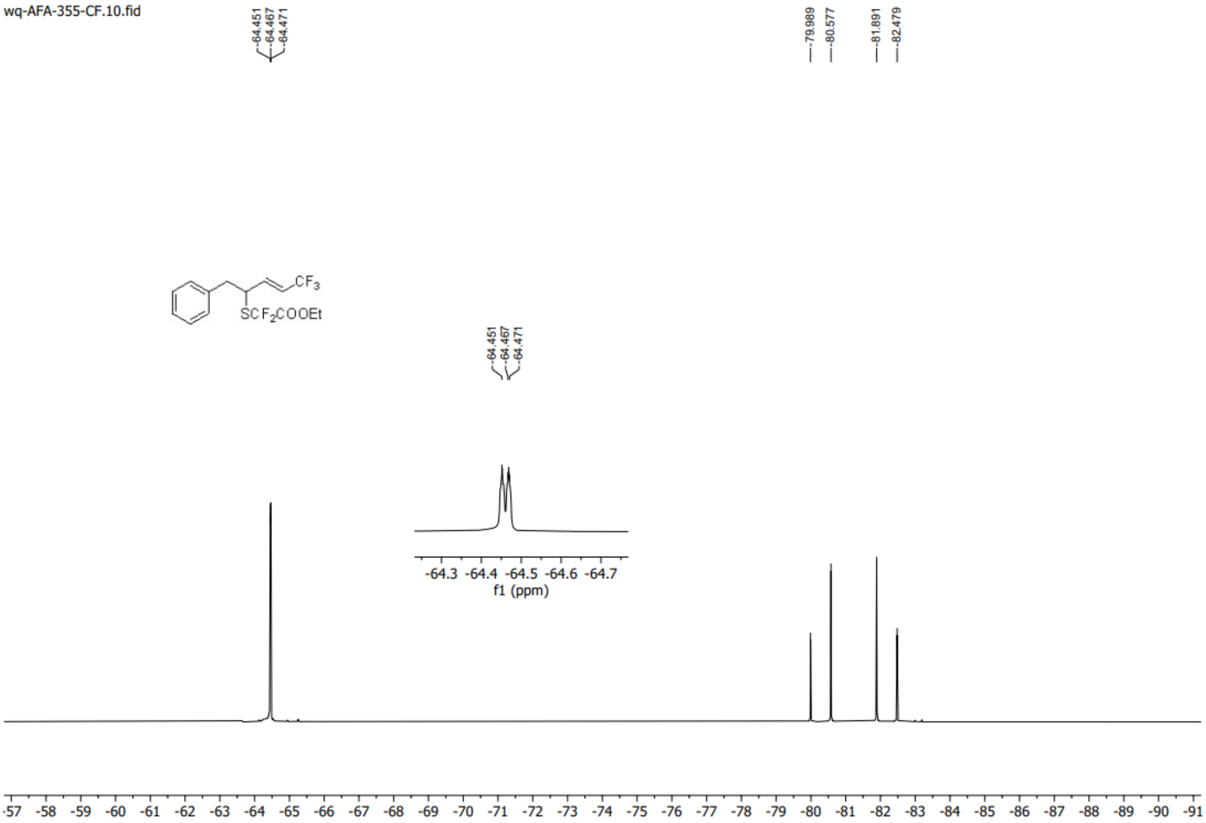

Ethyl (*R*,*E*)-2-((1-cyclohexyl-4,4,4-trifluorobut-2-en-1-yl)thio)-2,2-difluoroacetate (**4c**) (^{1}H NMR)

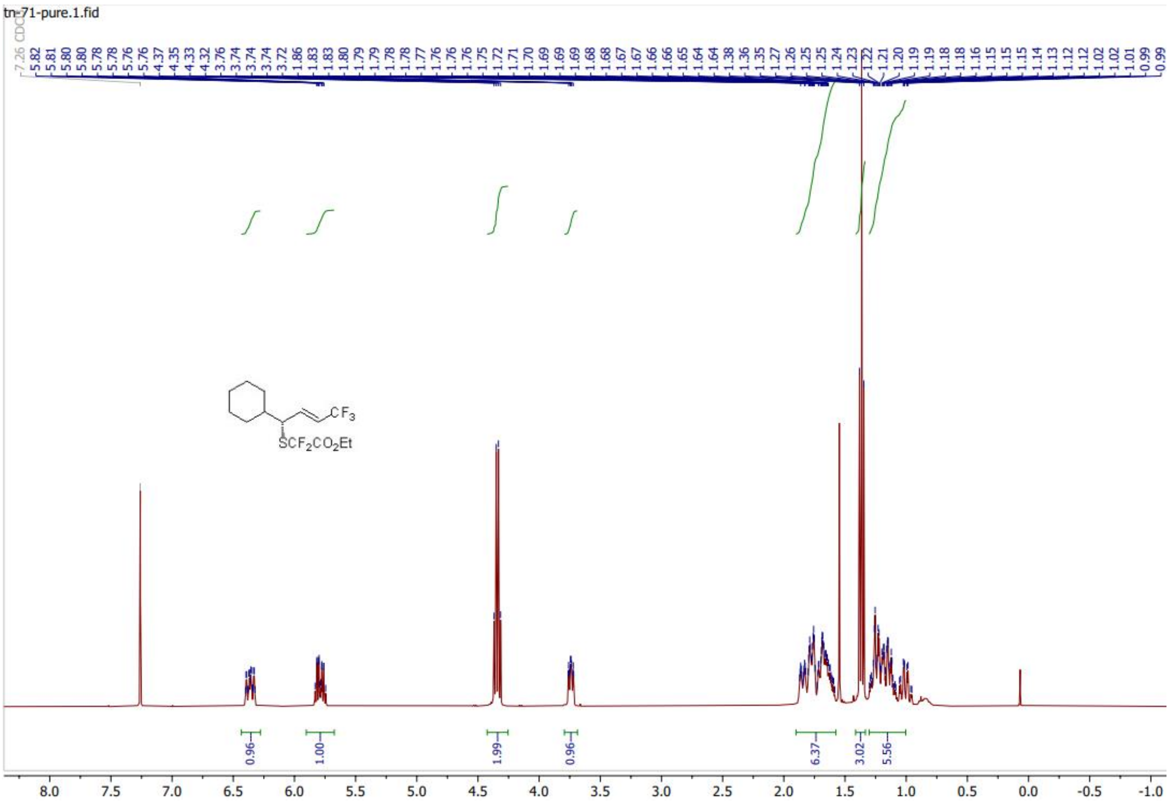

Ethyl (*R*,*E*)-2-((1-cyclohexyl-4,4,4-trifluorobut-2-en-1-yl)thio)-2,2-difluoroacetate (**4c**) (^{13}C NMR)

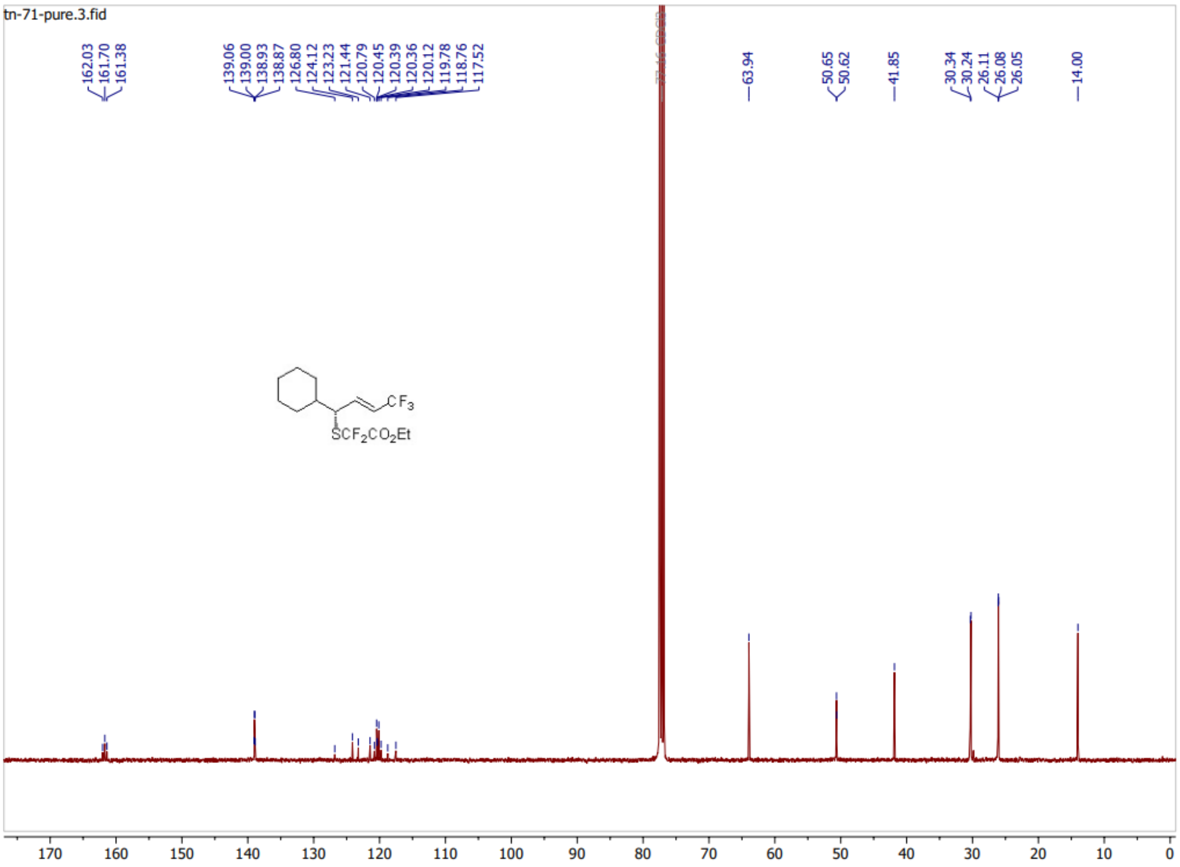

Ethyl (*R*,*E*)-2-((1-cyclohexyl-4,4,4-trifluorobut-2-en-1-yl)thio)-2,2-difluoroacetate (**4c**) (^{19}F NMR)

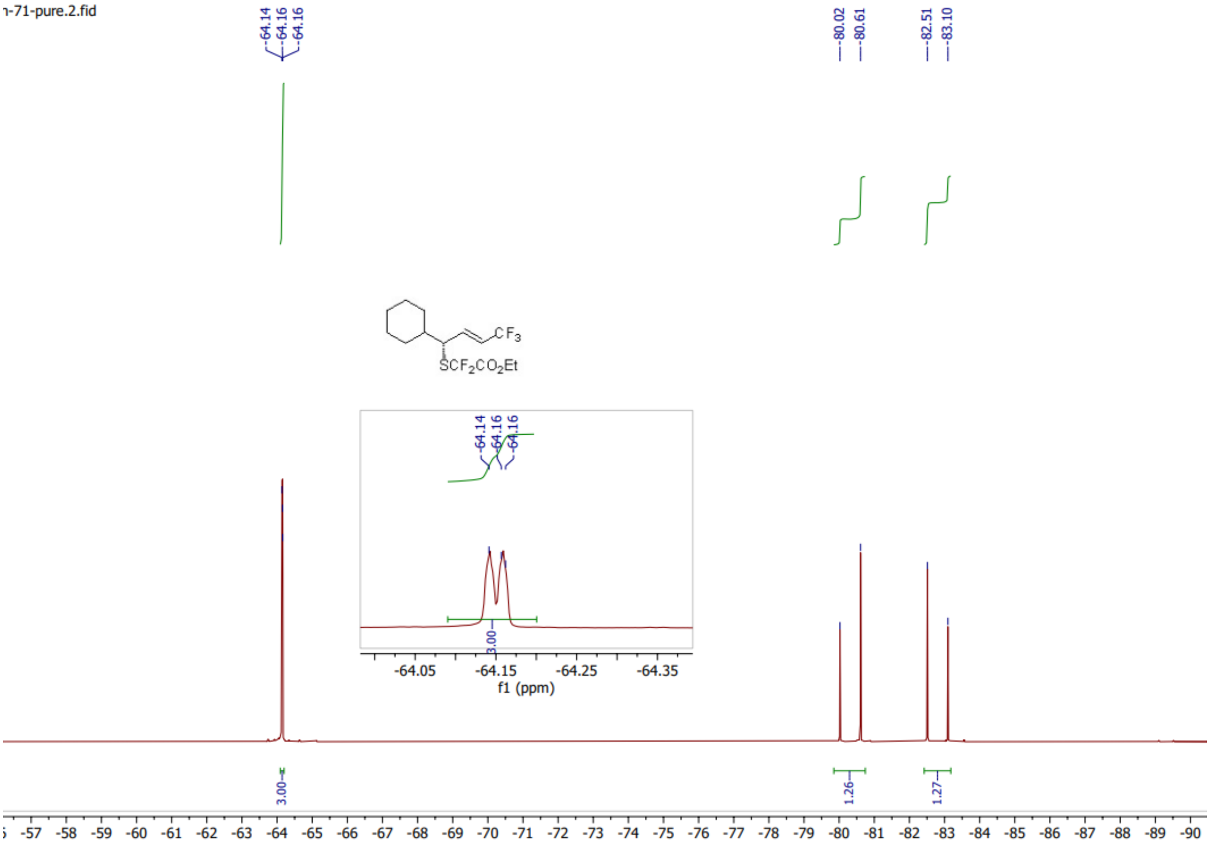

Ethyl (*S*,*E*)-2,2-difluoro-2-((4,4,4-trifluoro-1-phenylbut-2-en-1-yl)thio)acetate (**4d**) (^{1}H NMR)

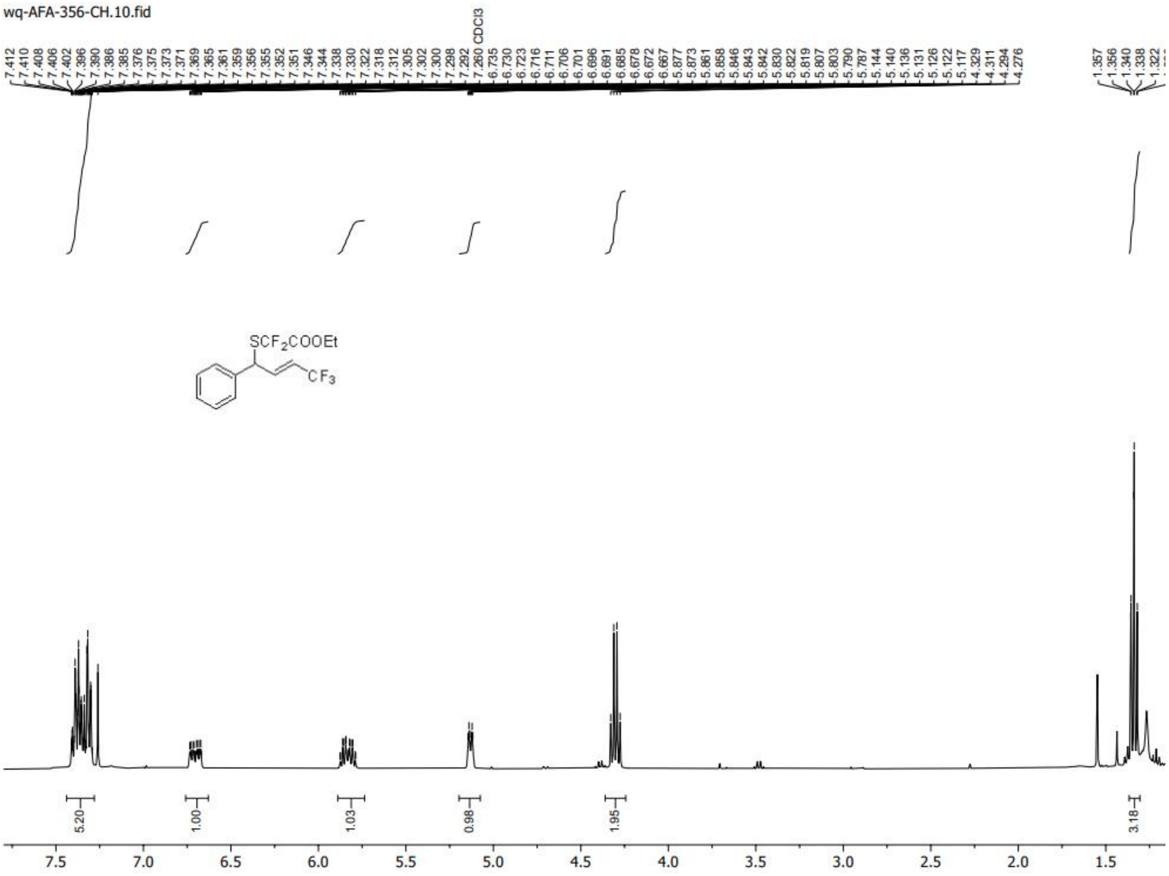

Ethyl (*S*,*E*)-2,2-difluoro-2-((4,4,4-trifluoro-1-phenylbut-2-en-1-yl)thio)acetate (**4d**) (^{13}C NMR)

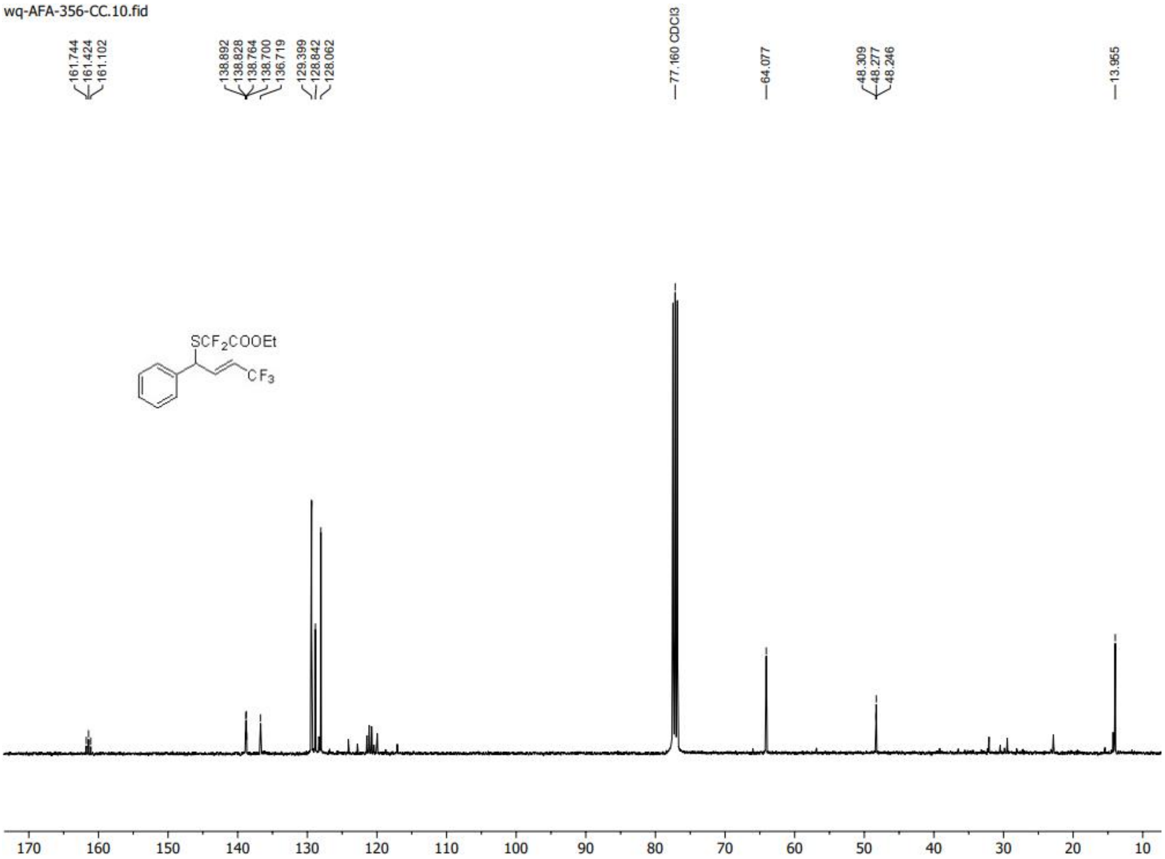

Ethyl (*S*,*E*)-2,2-difluoro-2-((4,4,4-trifluoro-1-phenylbut-2-en-1-yl)thio)acetate (**4d**) (^{19}F NMR)

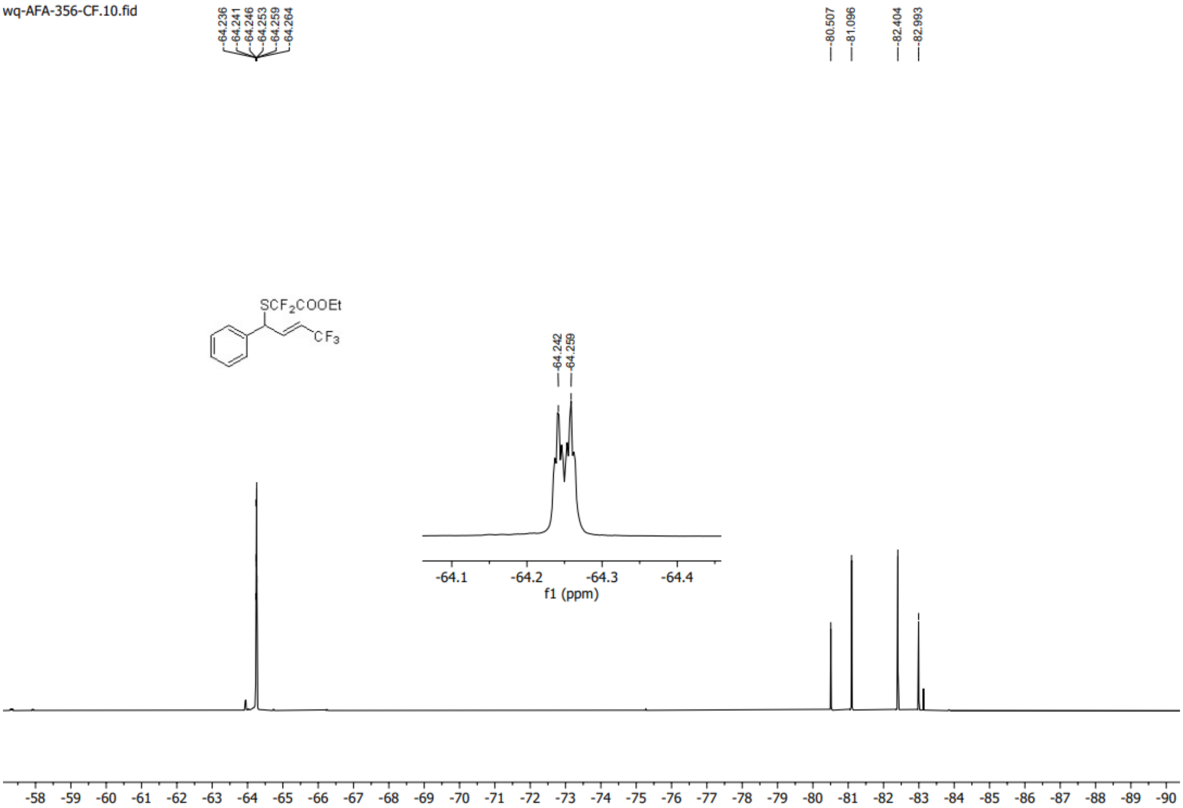

(*R*,*E*)-(Difluoro(phenylsulfonyl)methyl)(1,1,1-trifluorodec-2-en-4-yl)sulfane (**4e**) (^{1}H NMR)

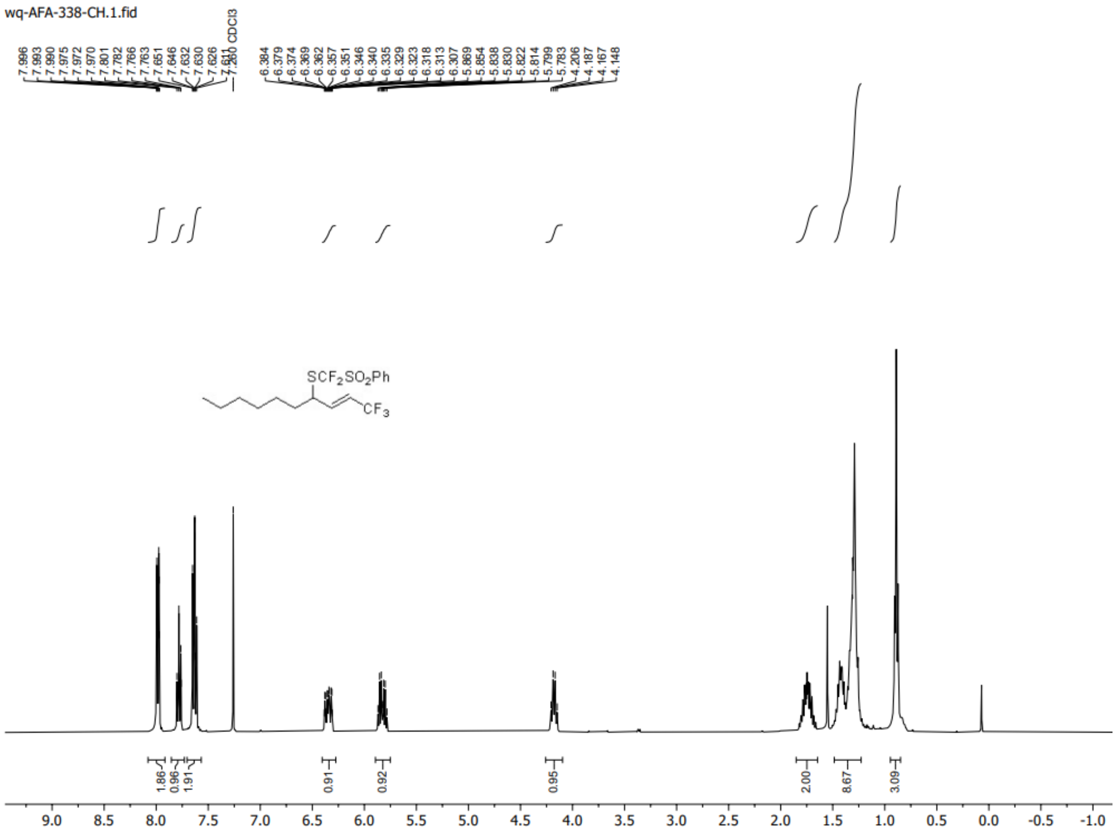

(*R*,*E*)-(Difluoro(phenylsulfonyl)methyl)(1,1,1-trifluorodec-2-en-4-yl)sulfane (**4e**) (^{13}C NMR)

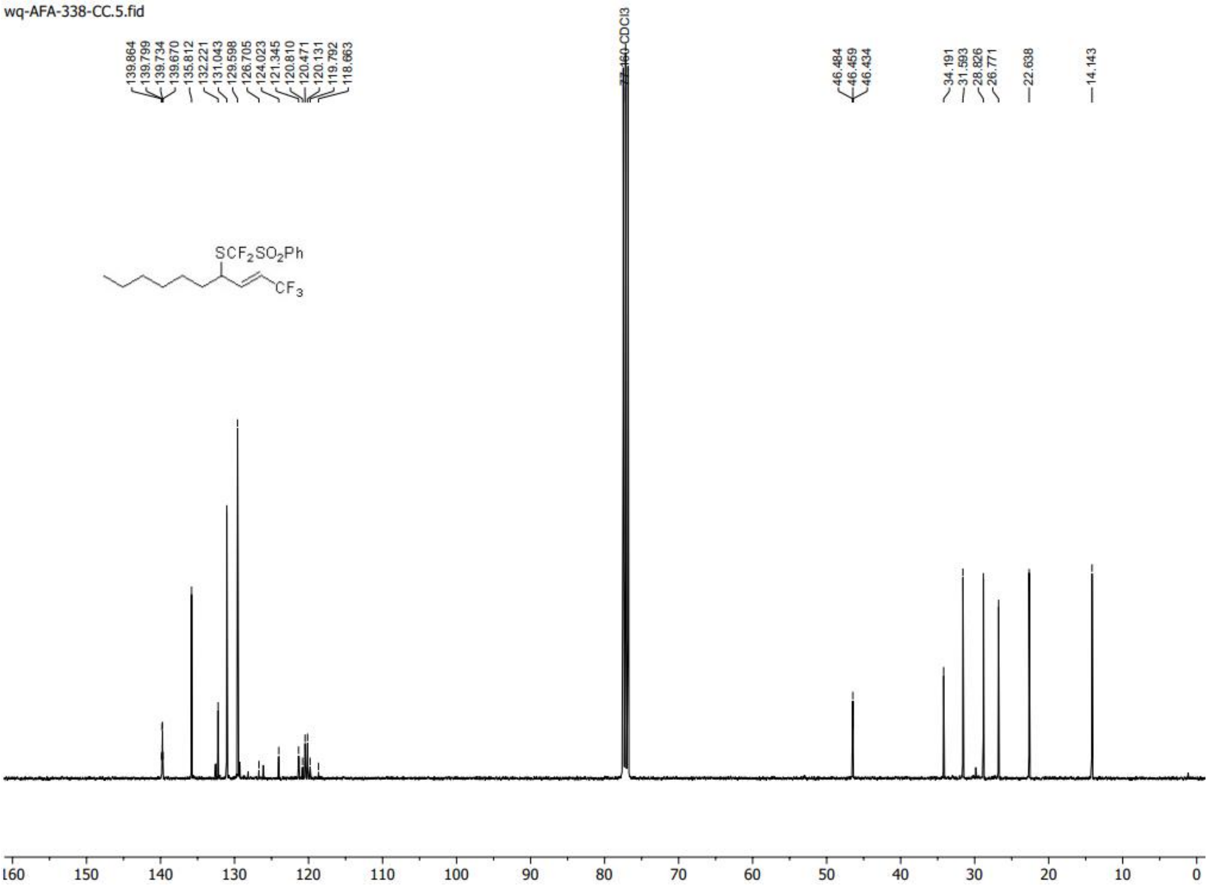

(*R*,*E*)-(Difluoro(phenylsulfonyl)methyl)(1,1,1-trifluorodec-2-en-4-yl)sulfane (**4e**) (^{19}F NMR)

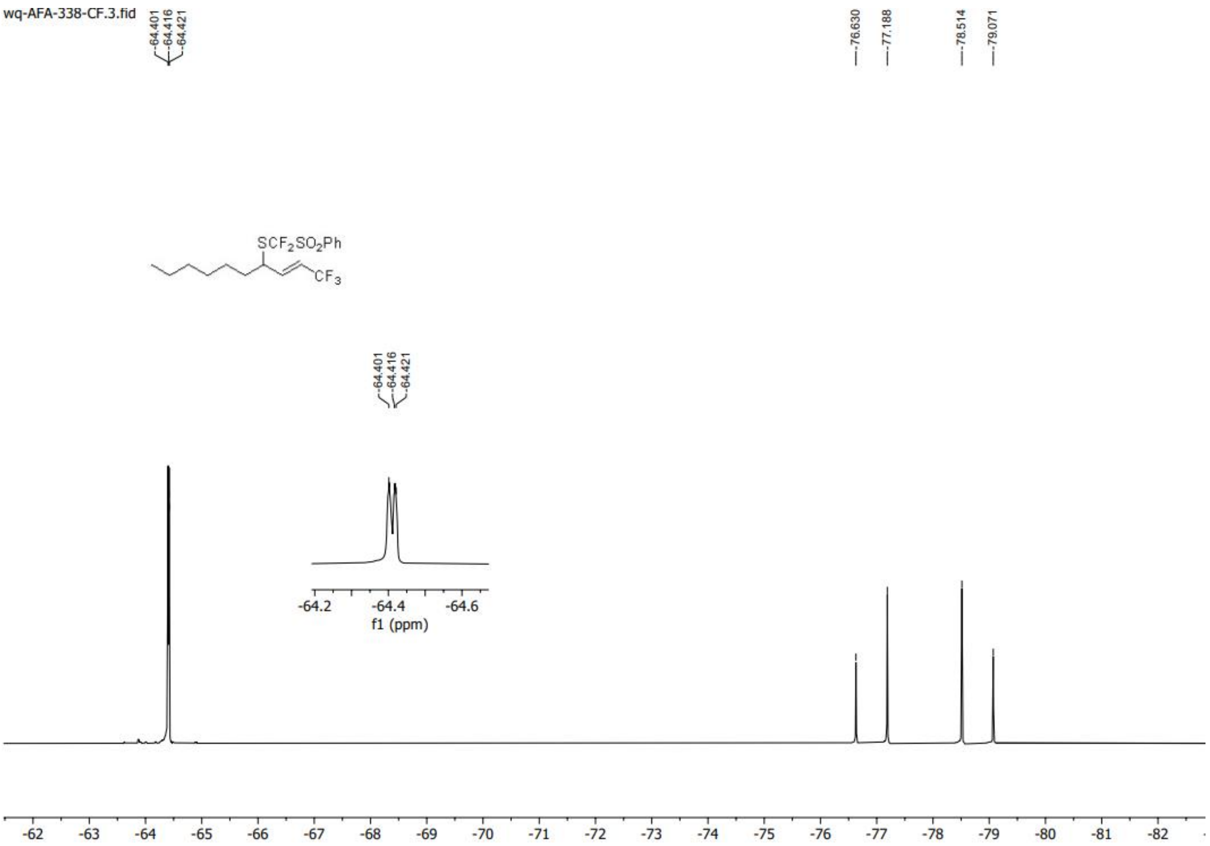

(*R*,*E*)-1,1,1-Trifluoro-4-thiocyanatodec-2-ene (**5a**) (^{1}H NMR)

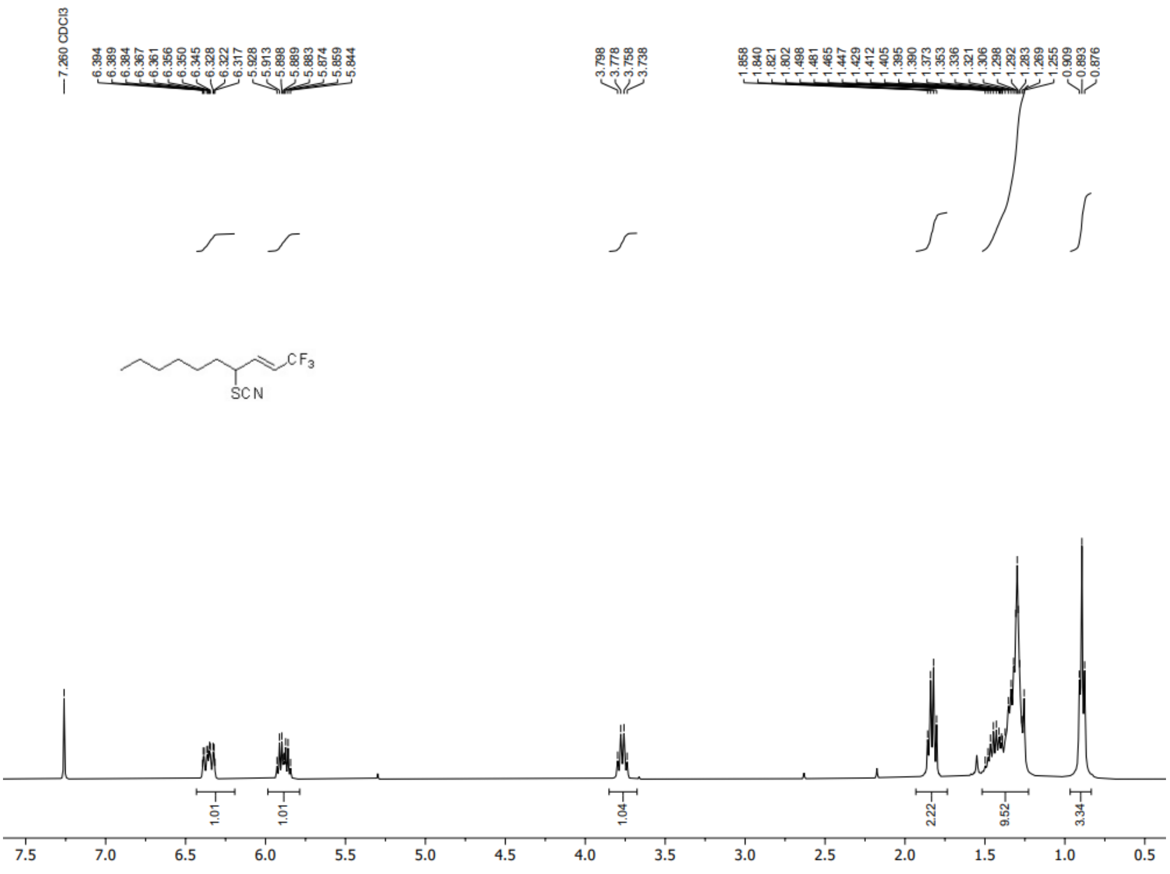

(*R*,*E*)-1,1,1-Trifluoro-4-thiocyanatodec-2-ene (**5a**) (^{13}C NMR)

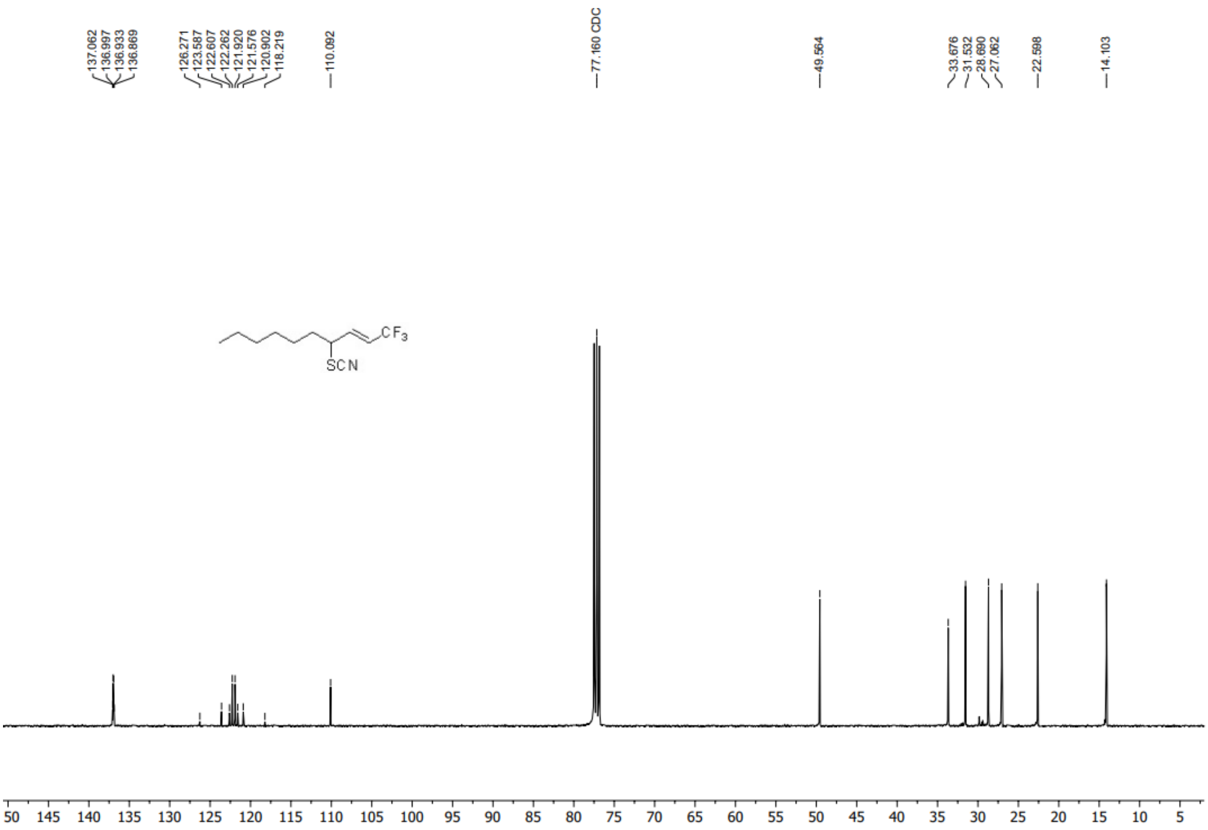

(*R*,*E*)-1,1,1-Trifluoro-4-thiocyanatodec-2-ene (**5a**) (^{19}F NMR)

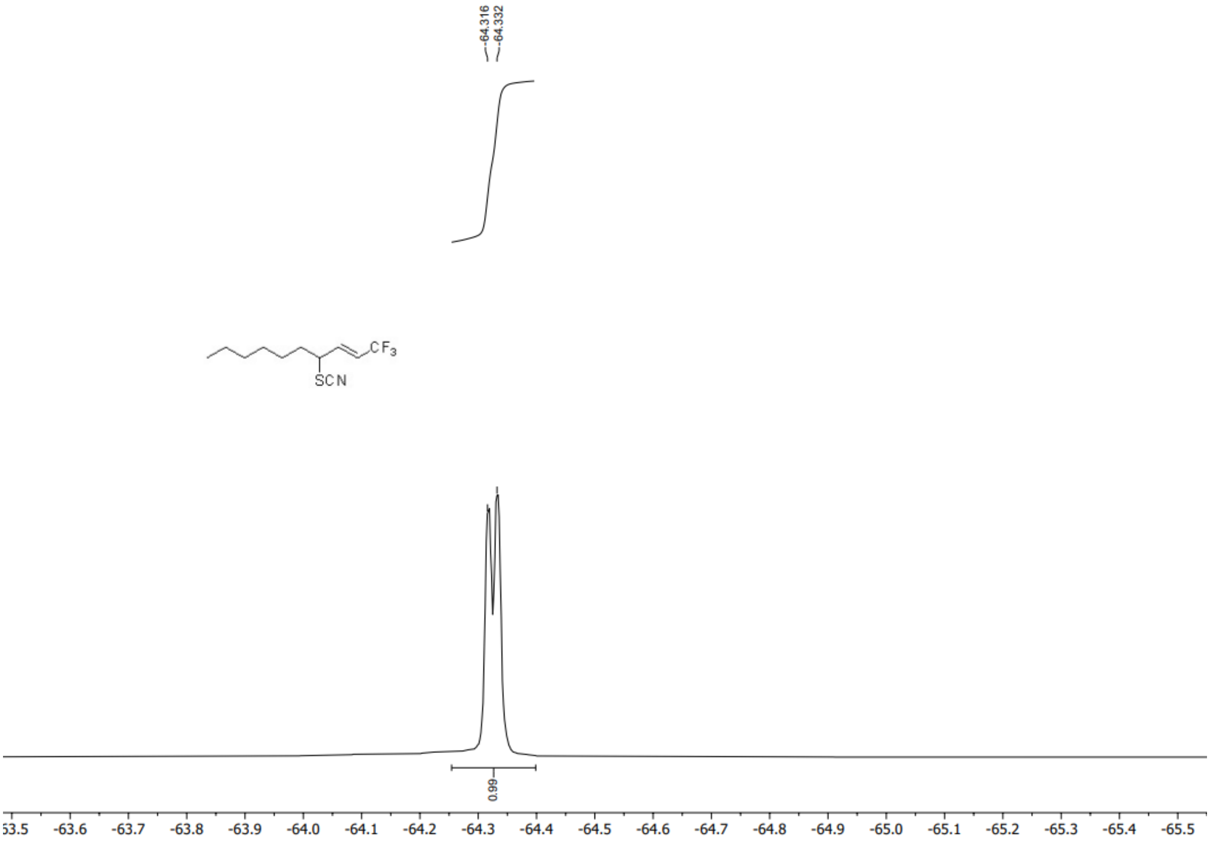

(*S*,*E*)-2-(5,5,5-Trifluoro-2-thiocyanatopent-3-en-1-yl)isoindoline-1,3-dione (**5b**) (^{1}H NMR)

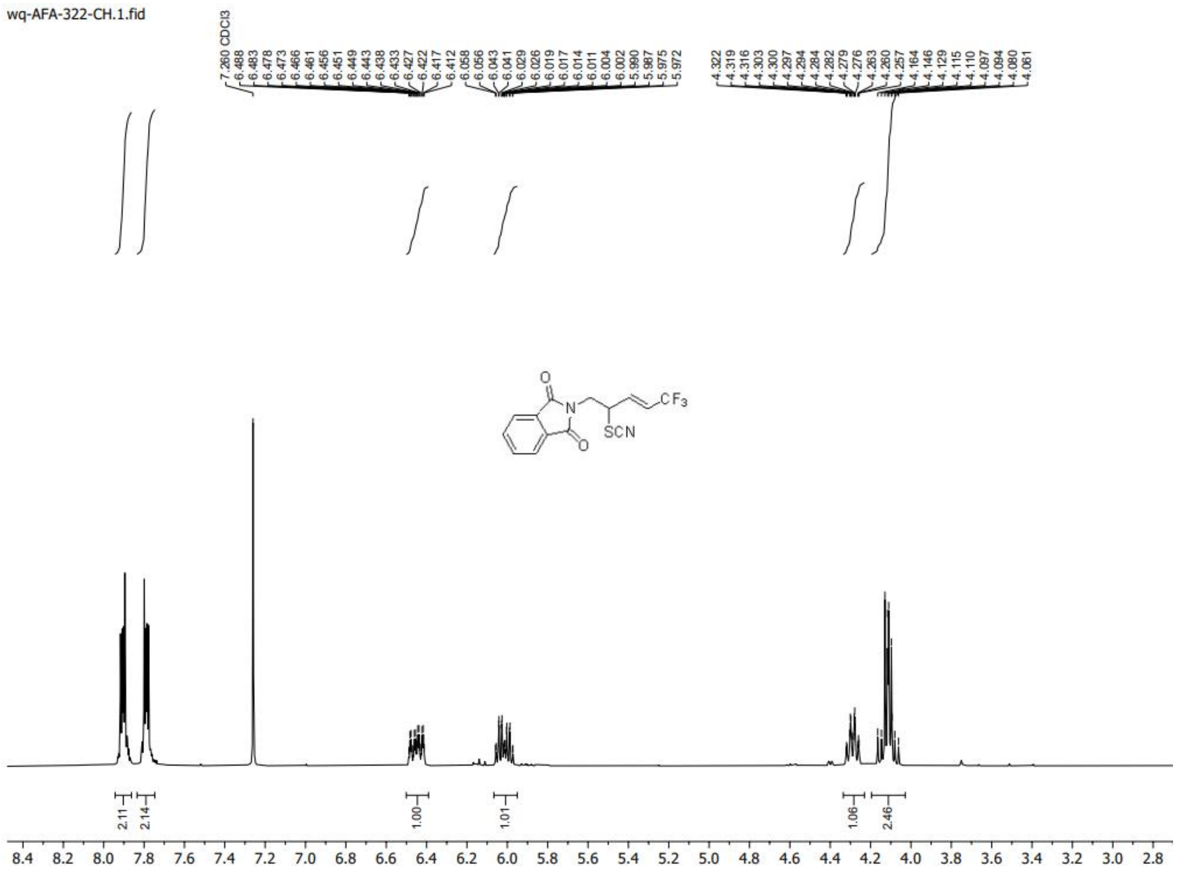

(*S*,*E*)-2-(5,5,5-Trifluoro-2-thiocyanatopent-3-en-1-yl)isoindoline-1,3-dione (**5b**) (^{13}C NMR)

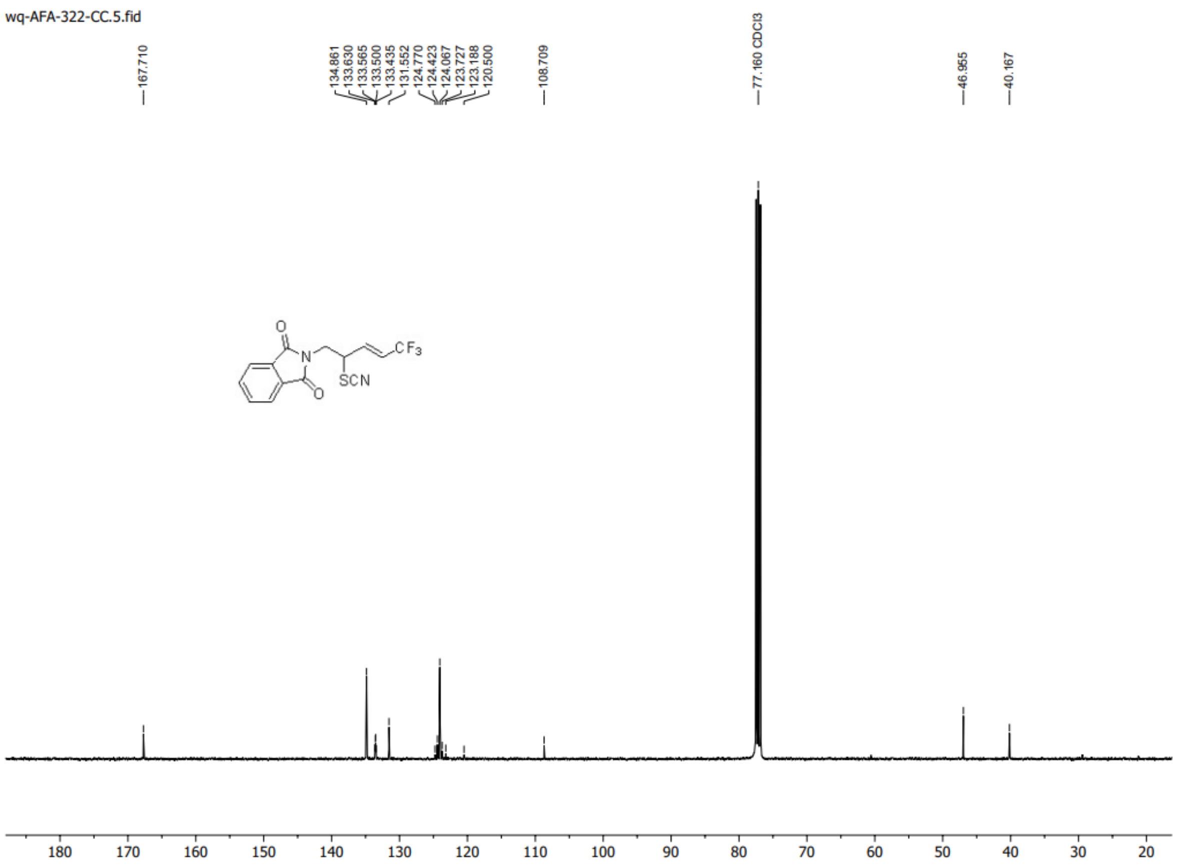

(*S*,*E*)-2-(5,5,5-Trifluoro-2-thiocyanatopent-3-en-1-yl)isoindoline-1,3-dione (**5b**) (^{19}F NMR)

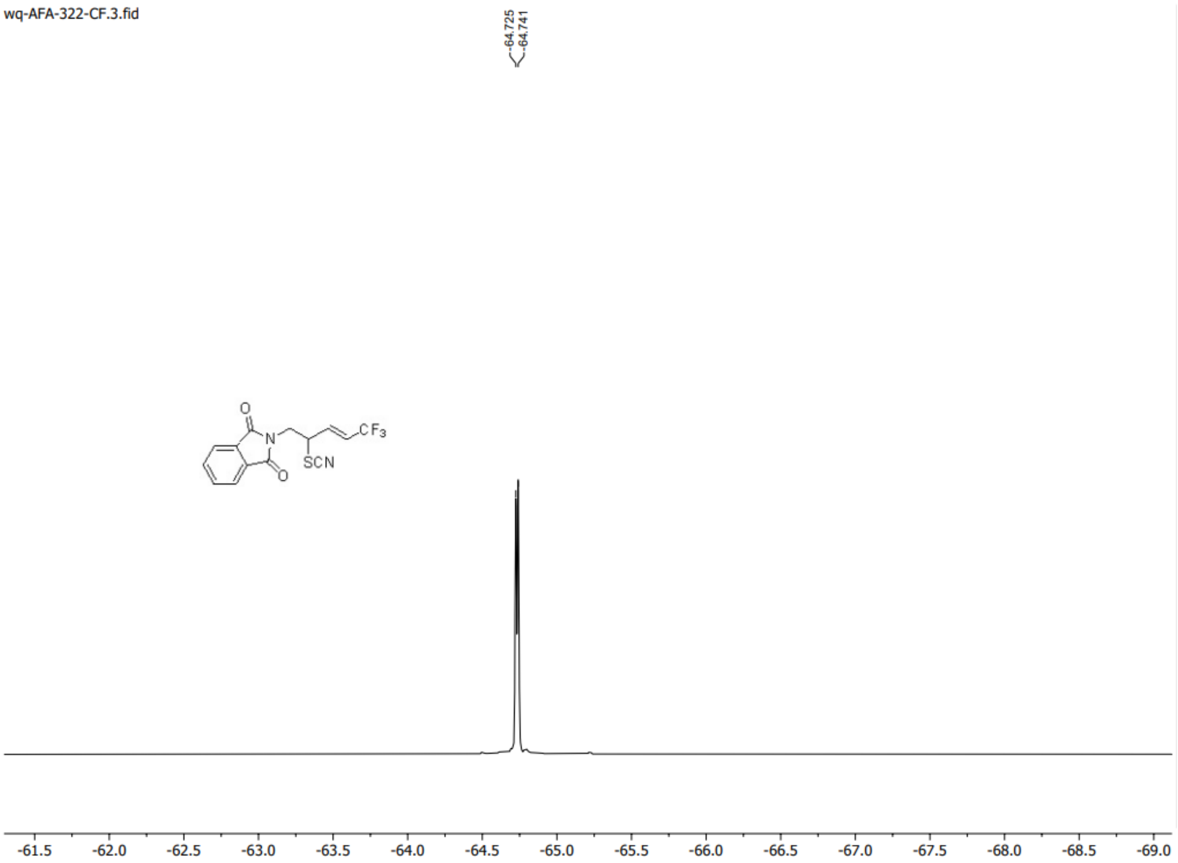

(*R*,*E*)-(4,4,4-Trifluoro-1-thiocyanatobut-2-en-1-yl)cyclohexane (**5c**) (^{1}H NMR)

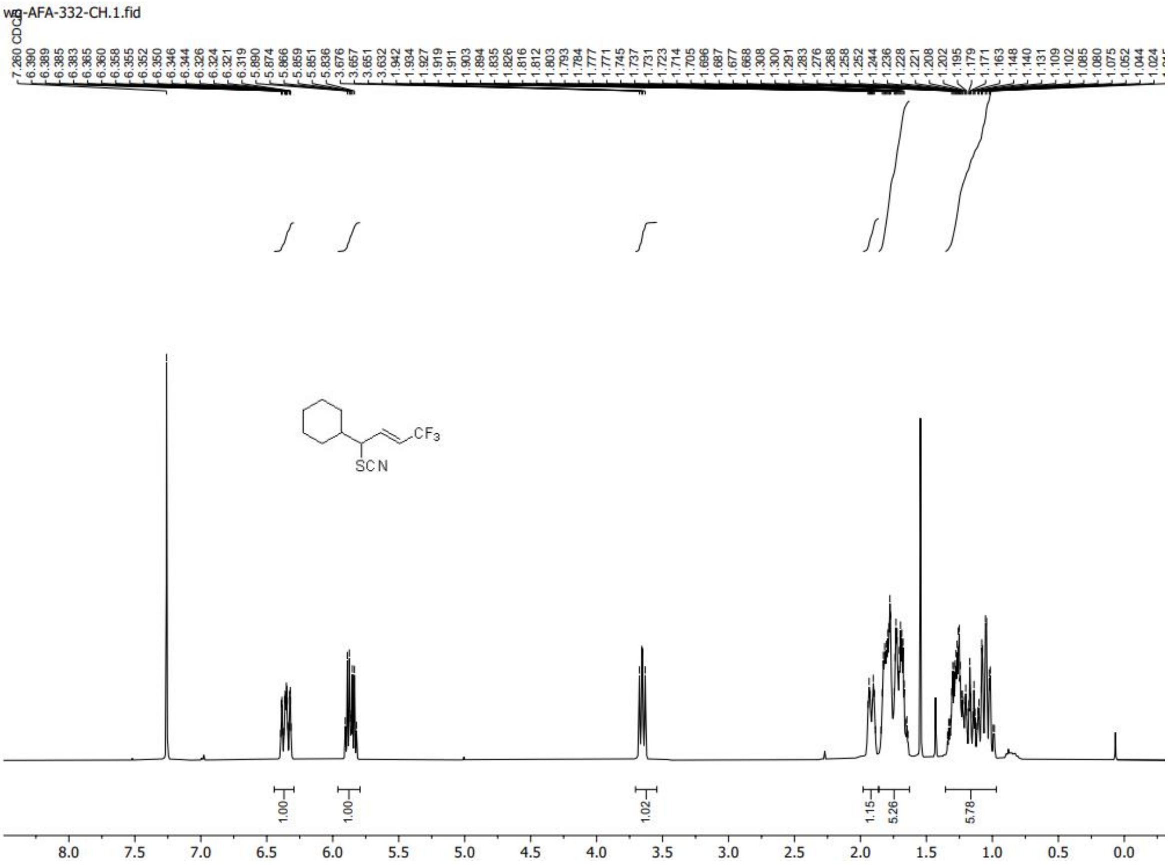

(*R*,*E*)-(4,4,4-Trifluoro-1-thiocyanatobut-2-en-1-yl)cyclohexane (**5c**) (^{13}C NMR)

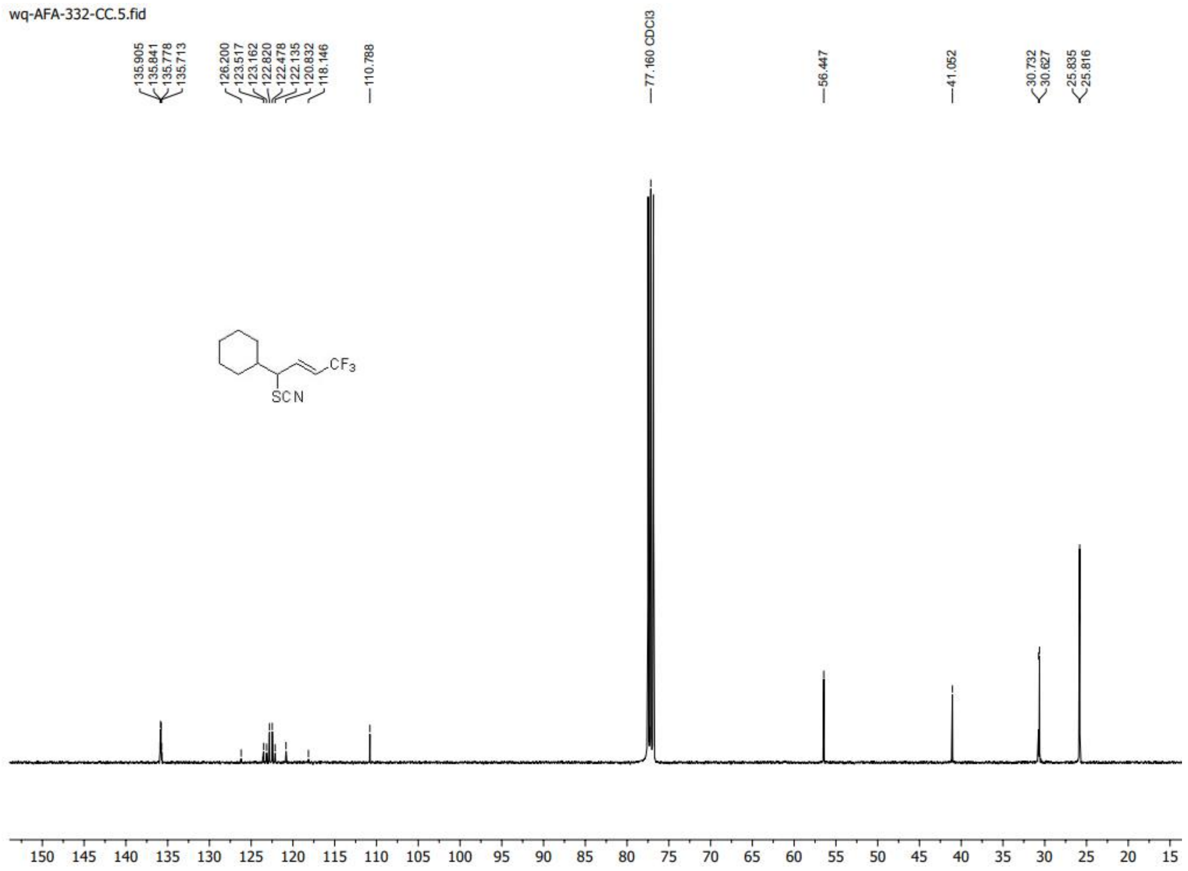

(*R*,*E*)-(4,4,4-Trifluoro-1-thiocyanatobut-2-en-1-yl)cyclohexane (**5c**) (^{19}F NMR)

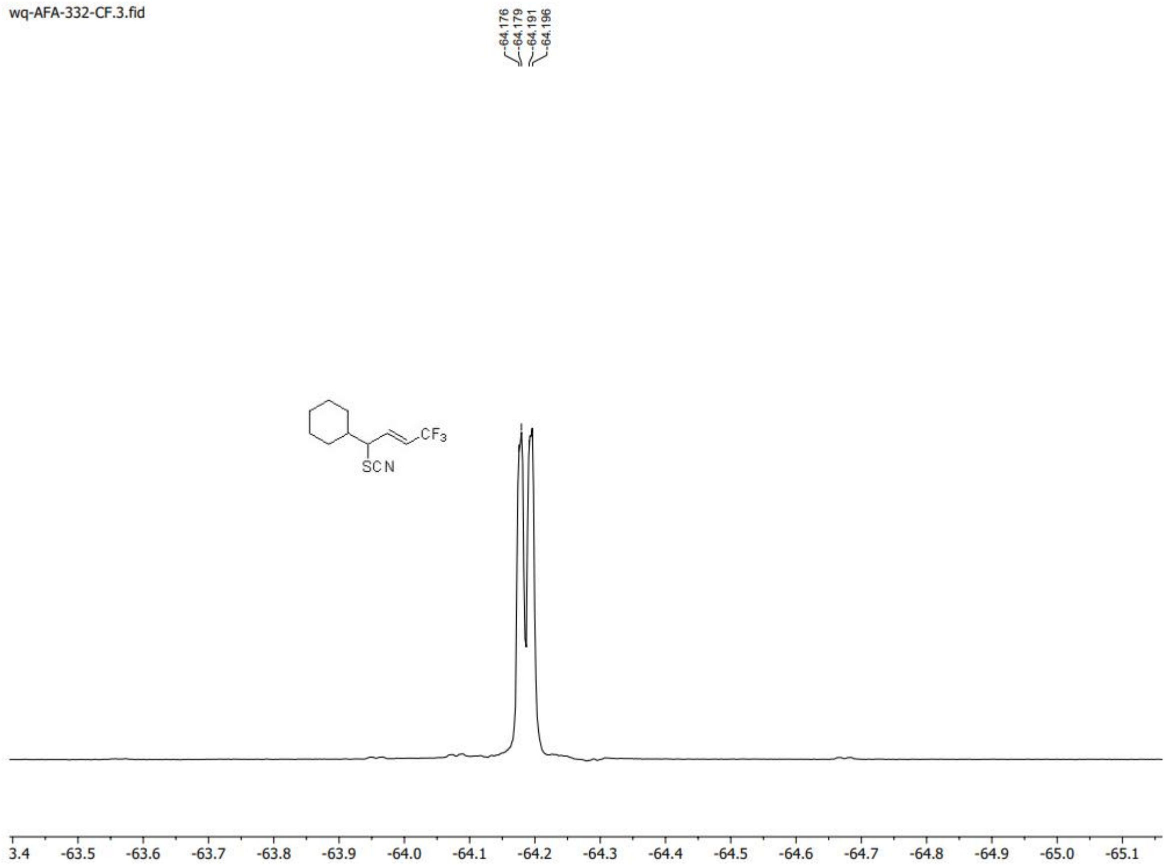

(*R*,*E*)-(4-Methoxyphenyl)(1,1,1-trifluorodec-2-en-4-yl)sulfane (**6a**) (^{1}H NMR)

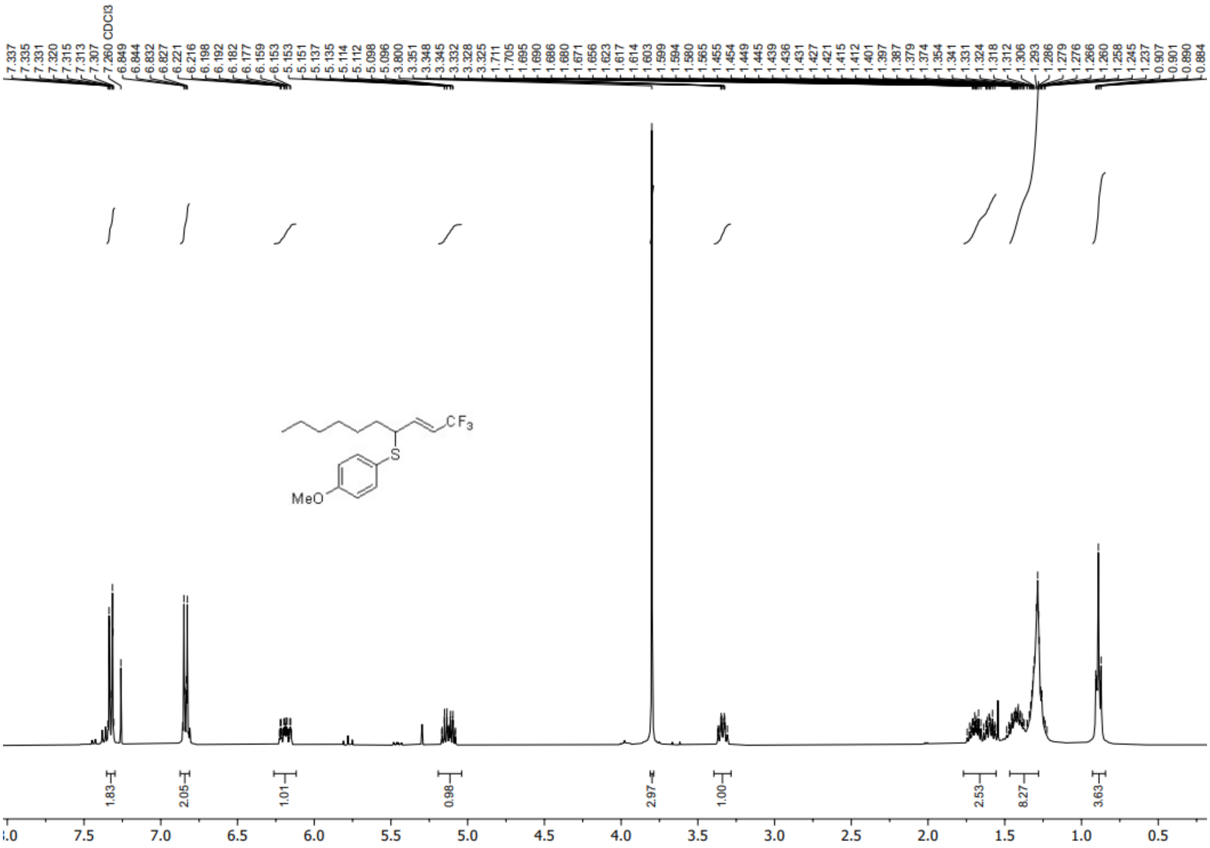

(*R*,*E*)-(4-Methoxyphenyl)(1,1,1-trifluorodec-2-en-4-yl)sulfane (**6a**) (^{13}C NMR)

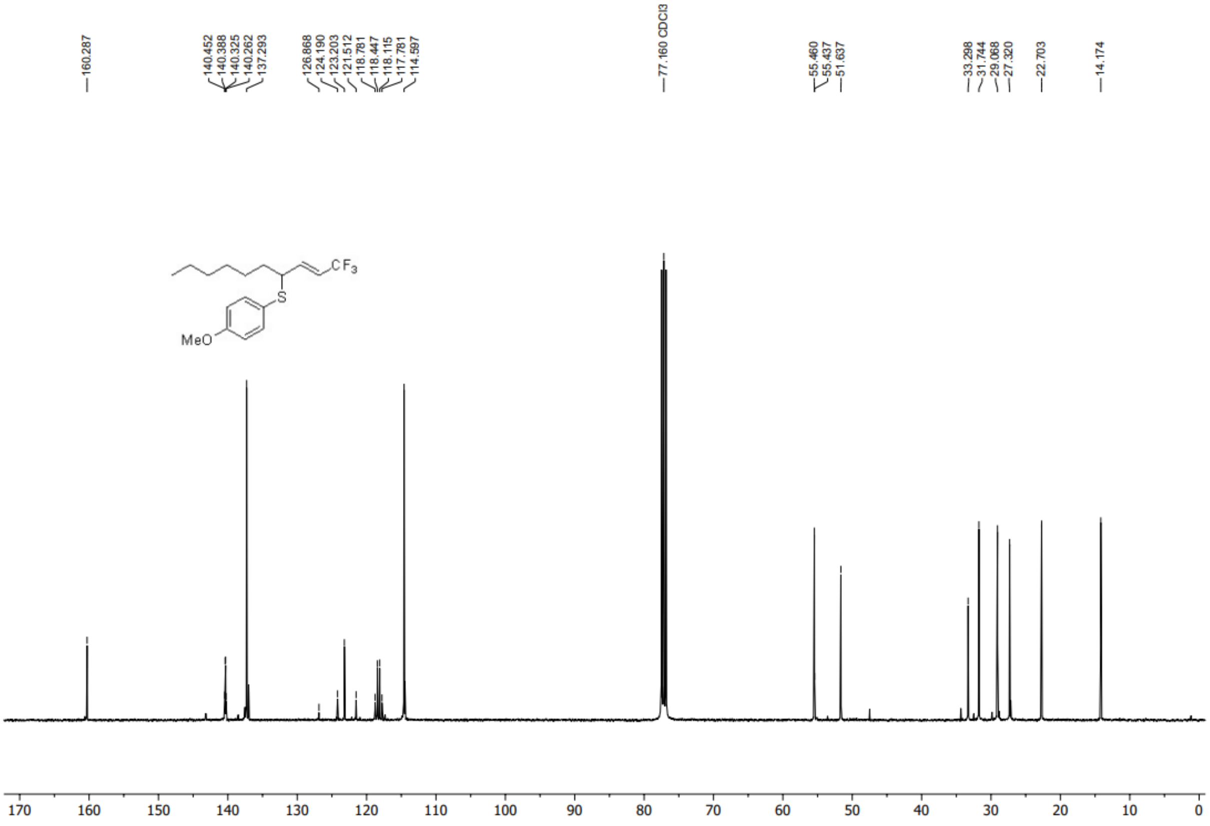

(*R*,*E*)-(4-Methoxyphenyl)(1,1,1-trifluorodec-2-en-4-yl)sulfane (**6a**) (^{19}F NMR)

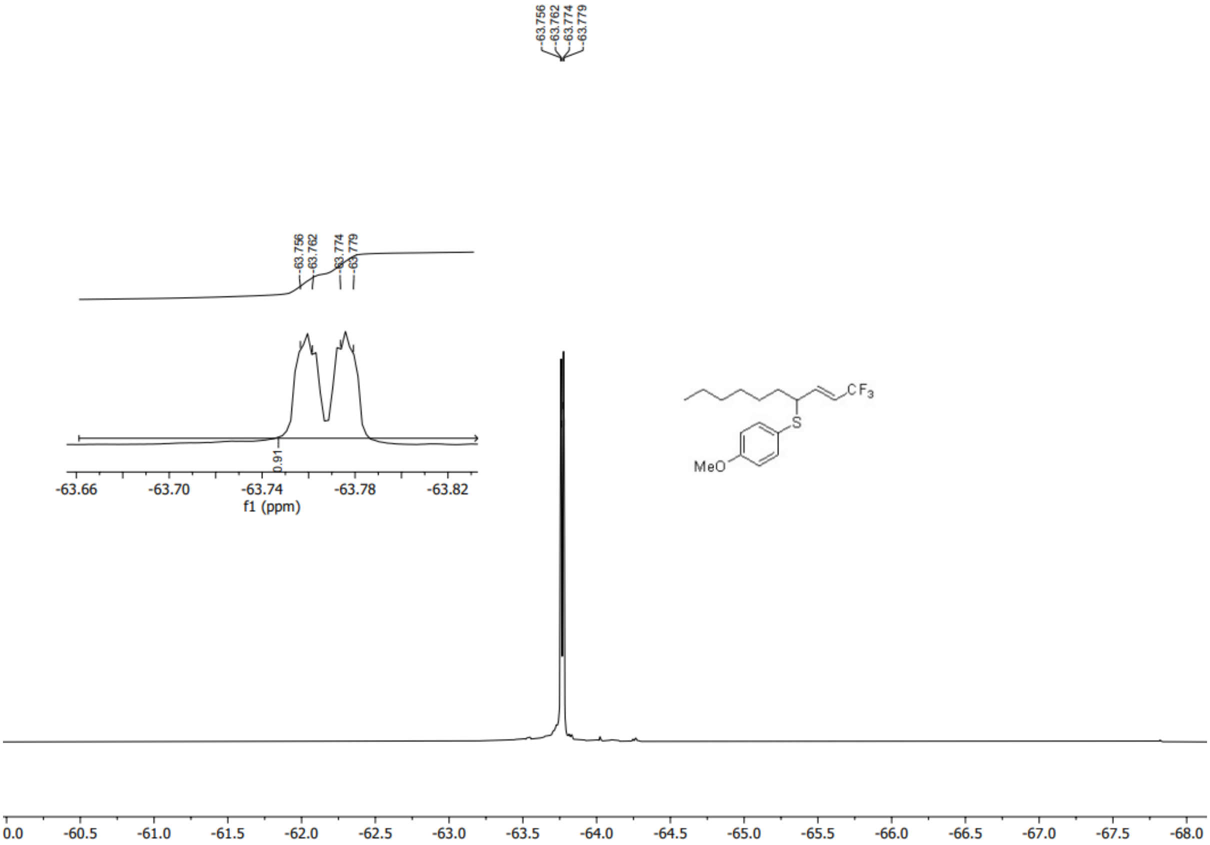

(*R*,*E*)-(4-Methoxyphenyl)(5,5,5-trifluoro-1-phenylpent-3-en-2-yl)sulfane (**6b**) (^{1}H NMR)

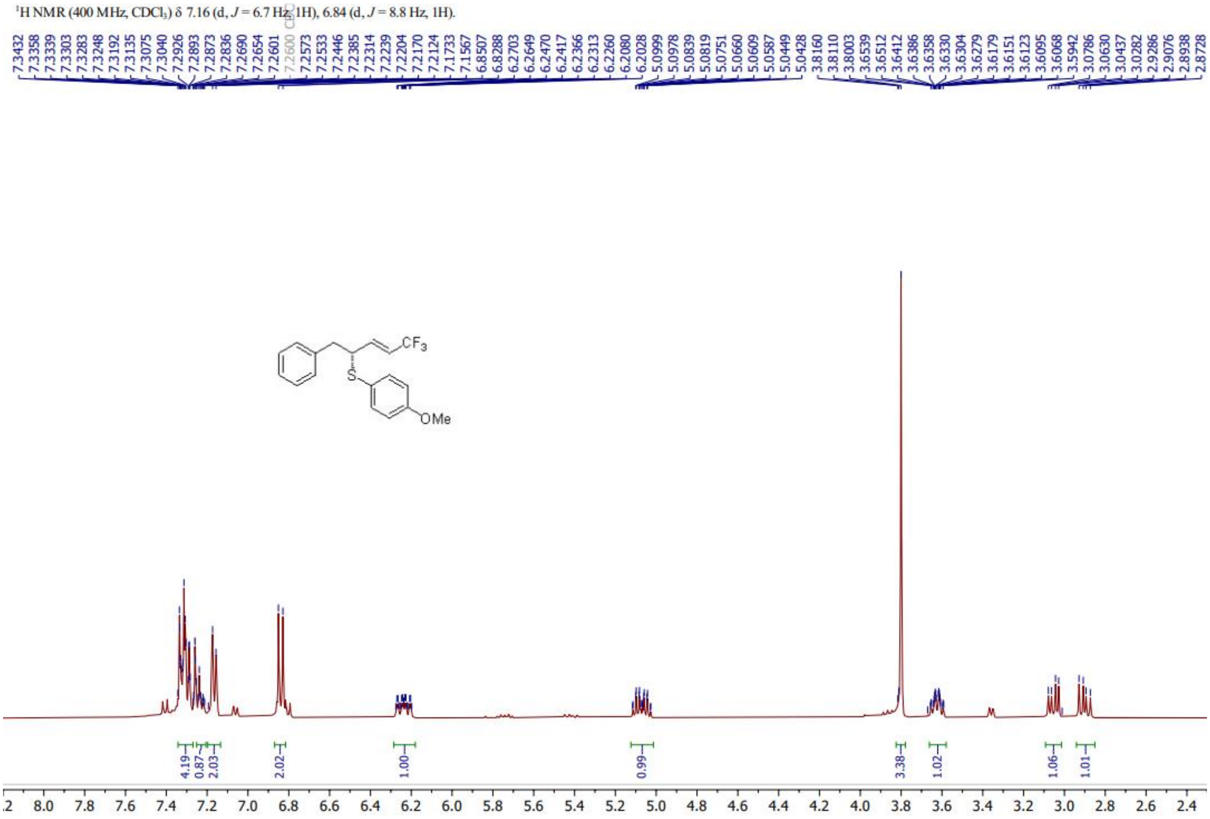

(*R*,*E*)-(4-Methoxyphenyl)(5,5,5-trifluoro-1-phenylpent-3-en-2-yl)sulfane (**6b**) (^{13}C NMR)

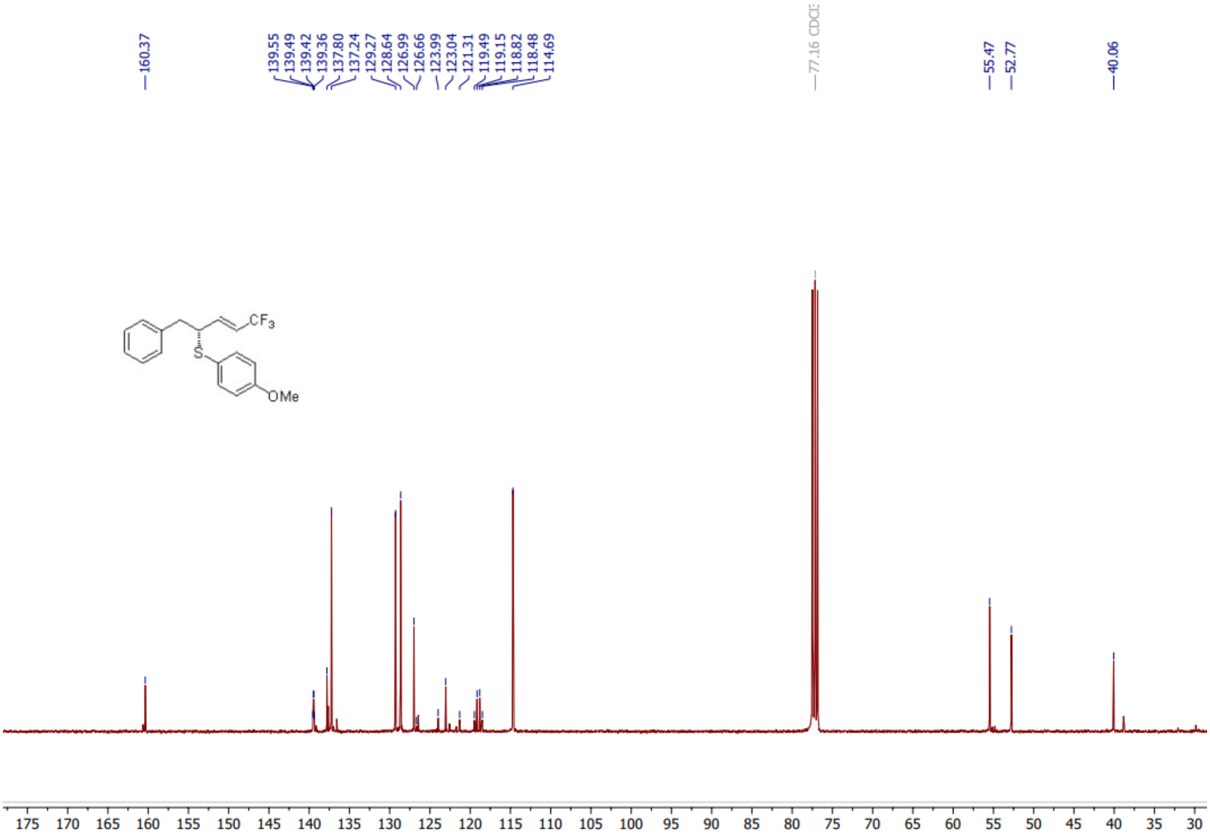

(*R*,*E*)-(4-Methoxyphenyl)(5,5,5-trifluoro-1-phenylpent-3-en-2-yl)sulfane (**6b**) (^{19}F NMR)

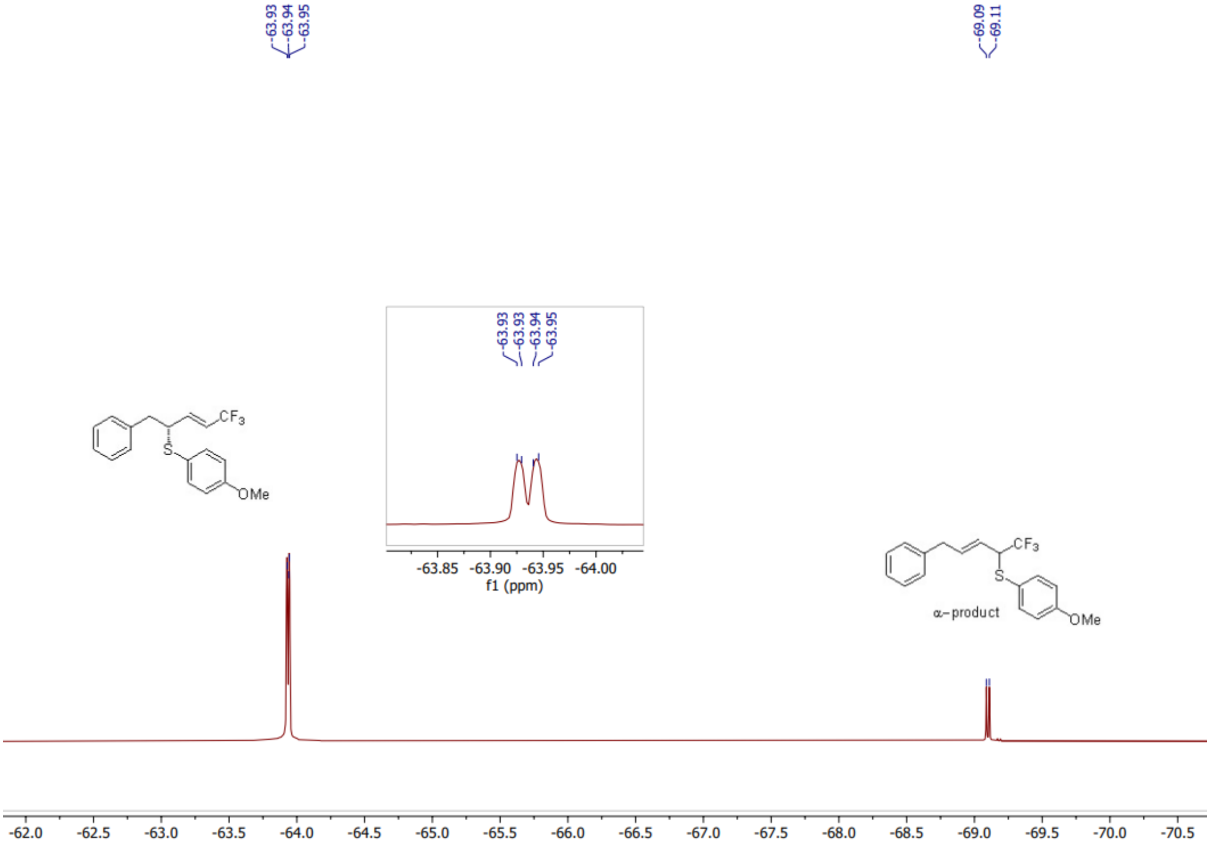

(*R*,*E*)-(8-Chloro-1,1,1-trifluorooct-2-en-4-yl)(4-methoxyphenyl)sulfane (**6c**) (^{1}H NMR)

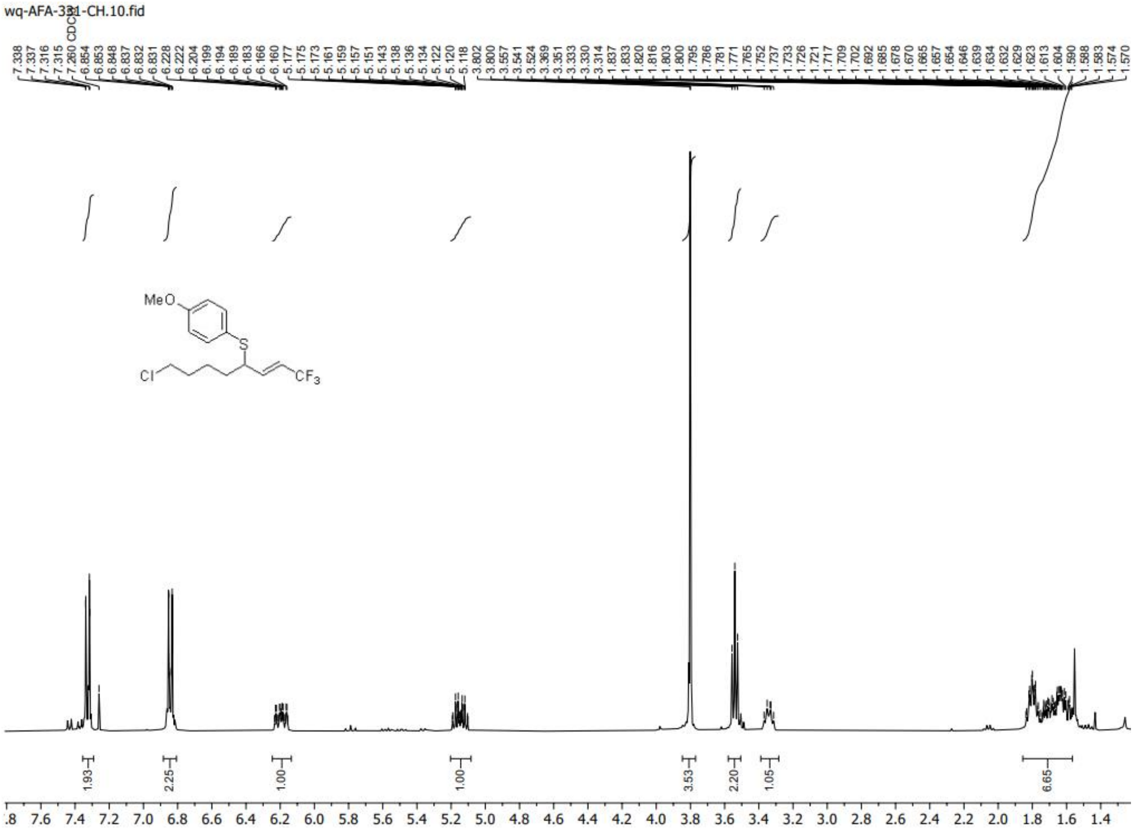

(*R*,*E*)-(8-Chloro-1,1,1-trifluorooct-2-en-4-yl)(4-methoxyphenyl)sulfane (**6c**) (^{13}C NMR)

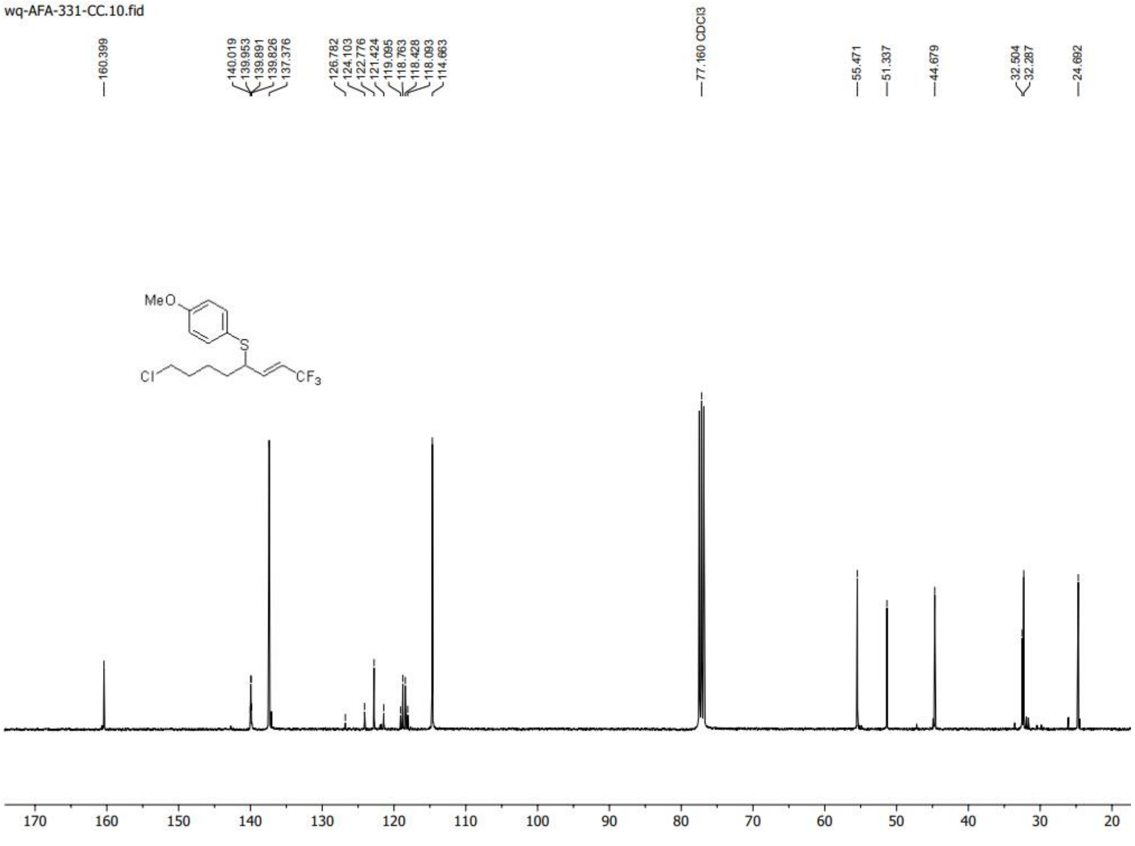

(*R*,*E*)-(8-Chloro-1,1,1-trifluorooct-2-en-4-yl)(4-methoxyphenyl)sulfane (**6c**) (^{19}F NMR)

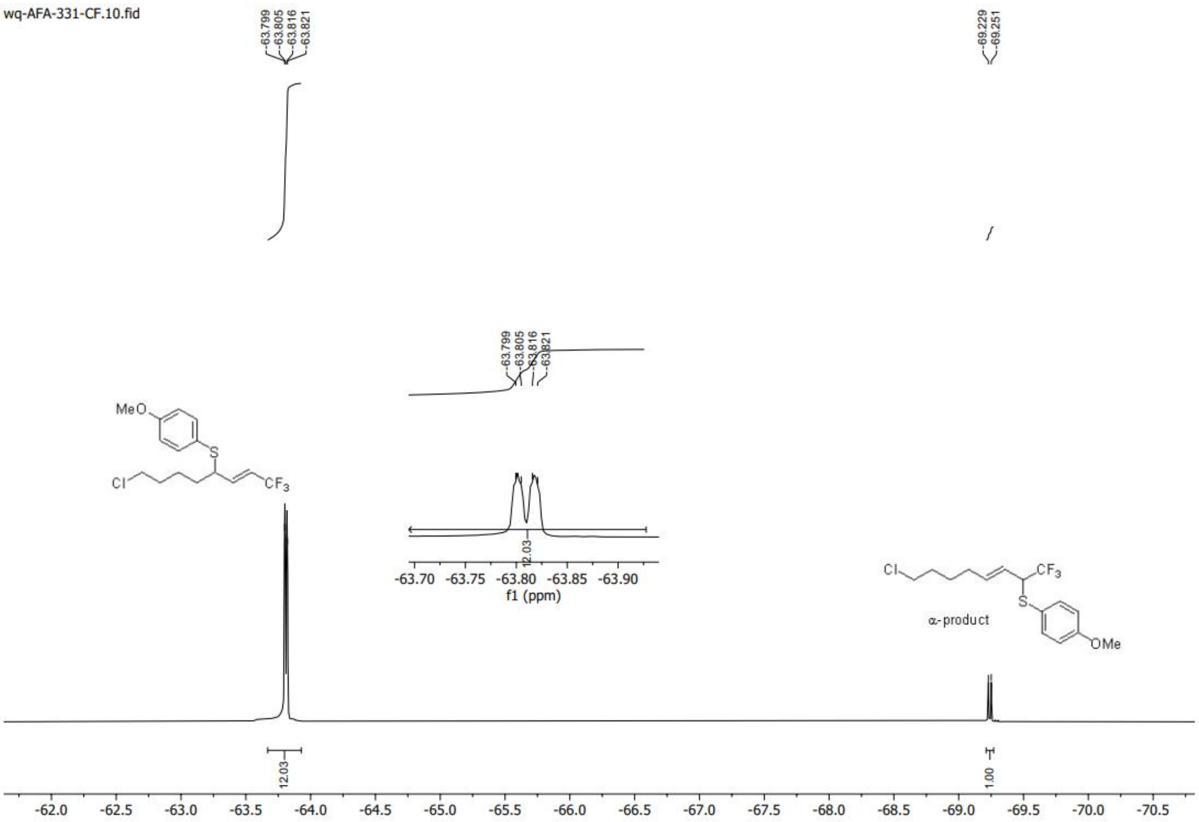

(*E*)-2-(5,5,5-Trifluoro-2-(phenylthio)pent-3-en-1-yl)isoindoline-1,3-dione (**6d**) (^{1}H NMR)

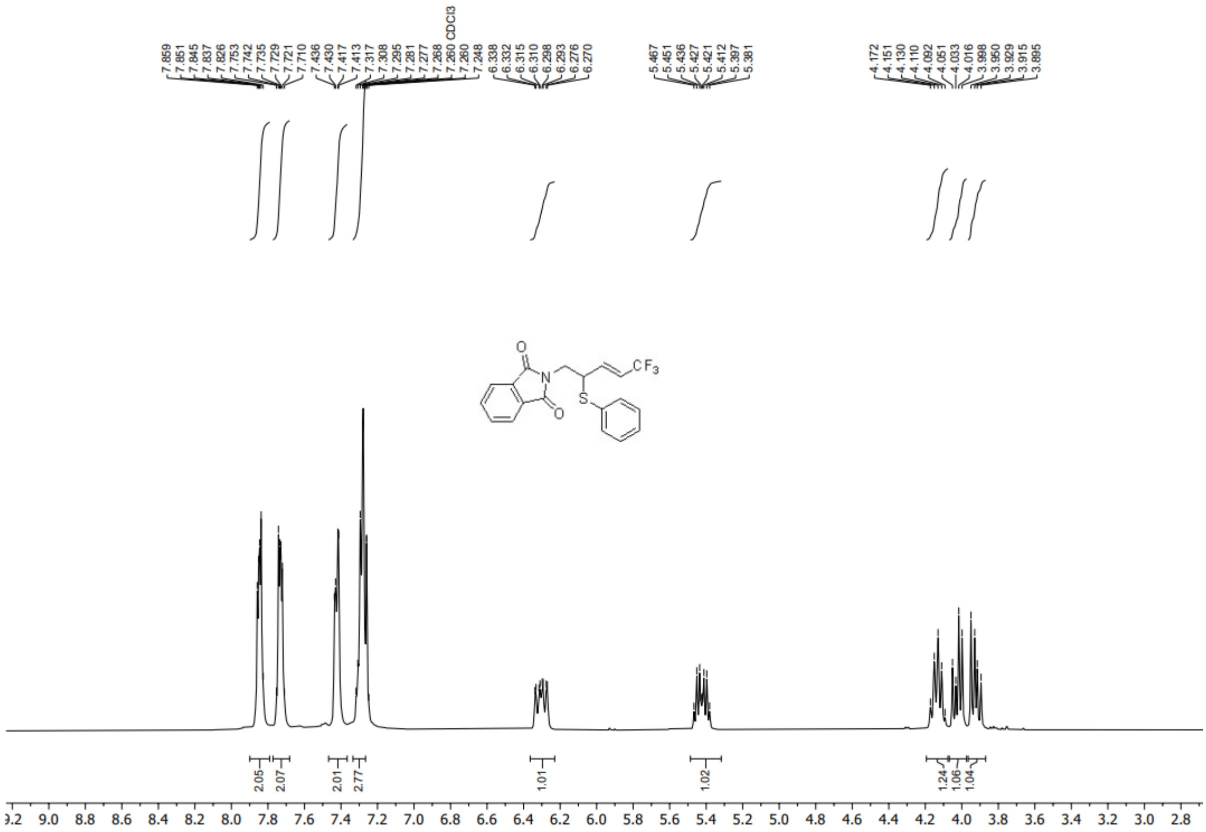

(*E*)-2-(5,5,5-Trifluoro-2-(phenylthio)pent-3-en-1-yl)isoindoline-1,3-dione (**6d**) (^{13}C NMR)

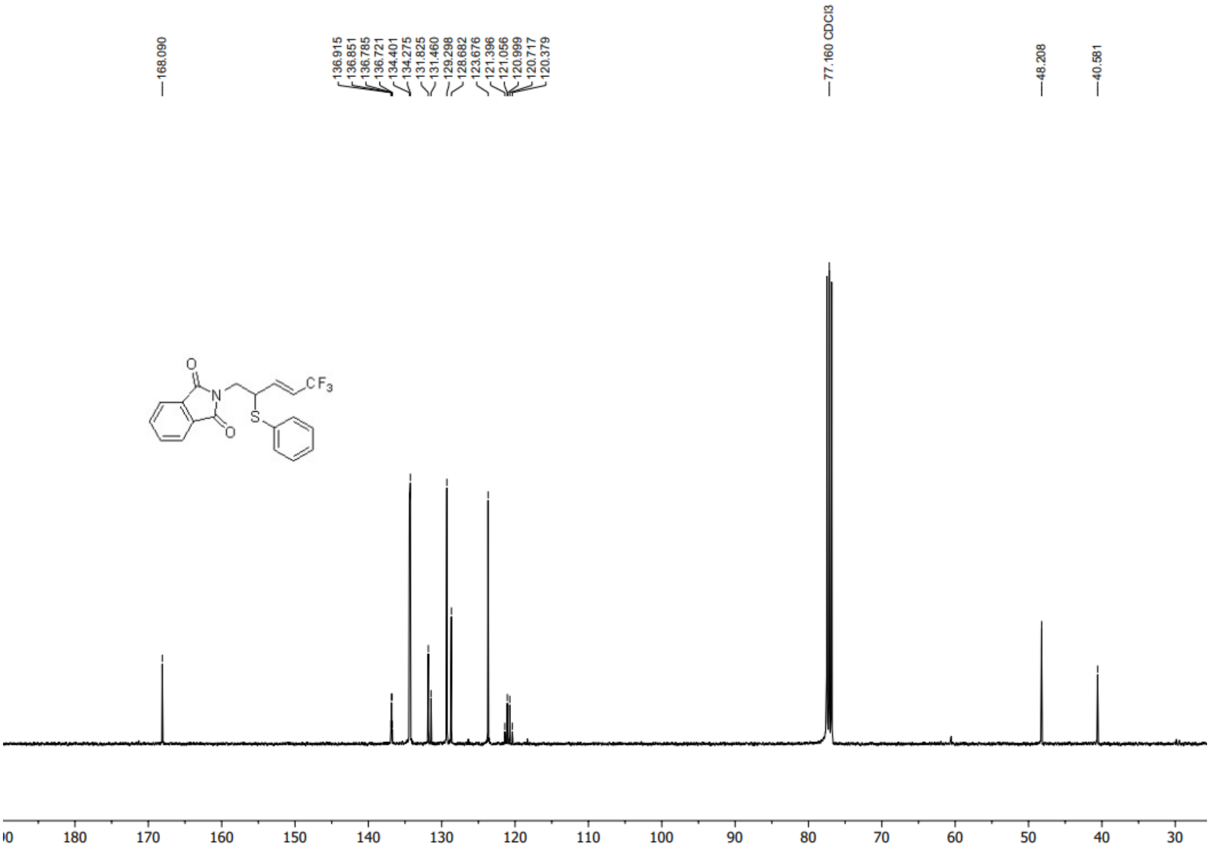

(*E*)-2-(5,5,5-Trifluoro-2-(phenylthio)pent-3-en-1-yl)isoindoline-1,3-dione (**6d**) (^{19}F NMR)

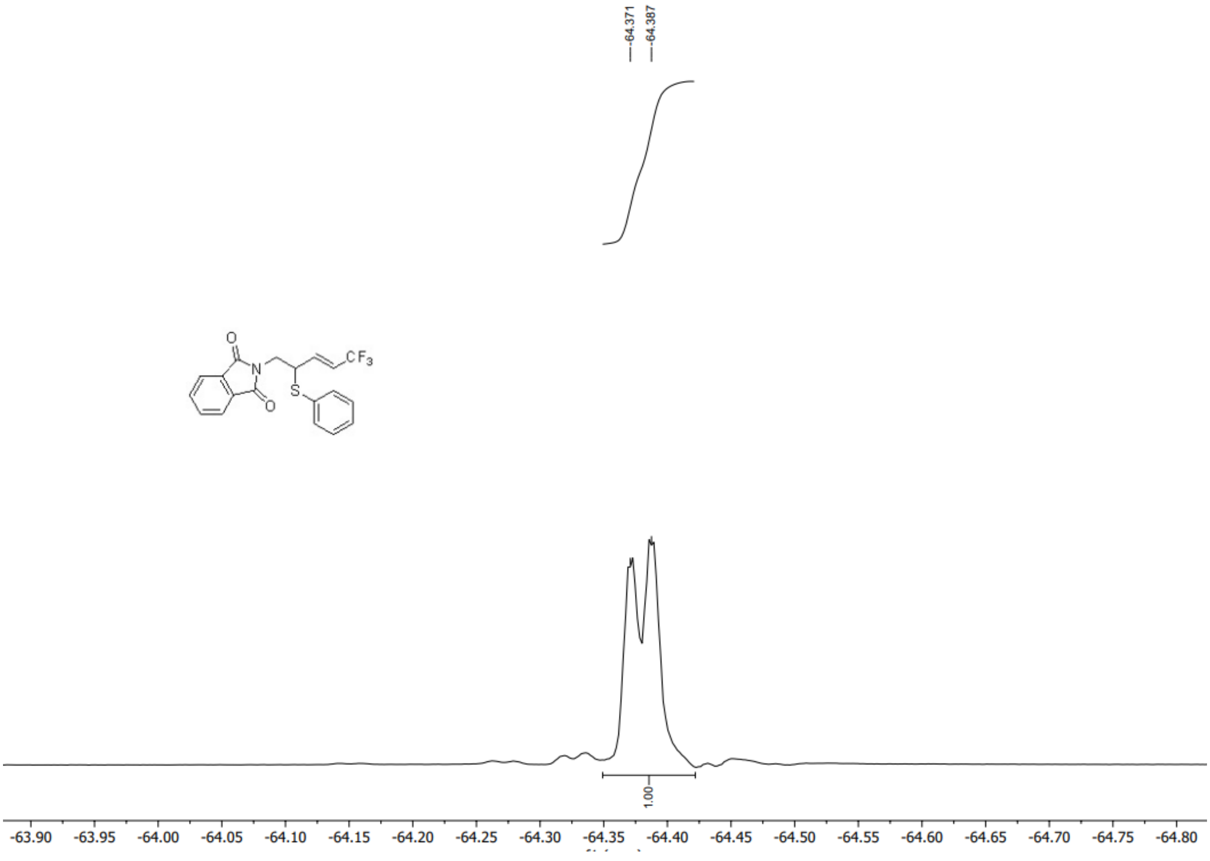

(*R*,*E*)-4-Bromo-1,1,1-trifluorodec-2-ene (**7a**) (^{1}H NMR)

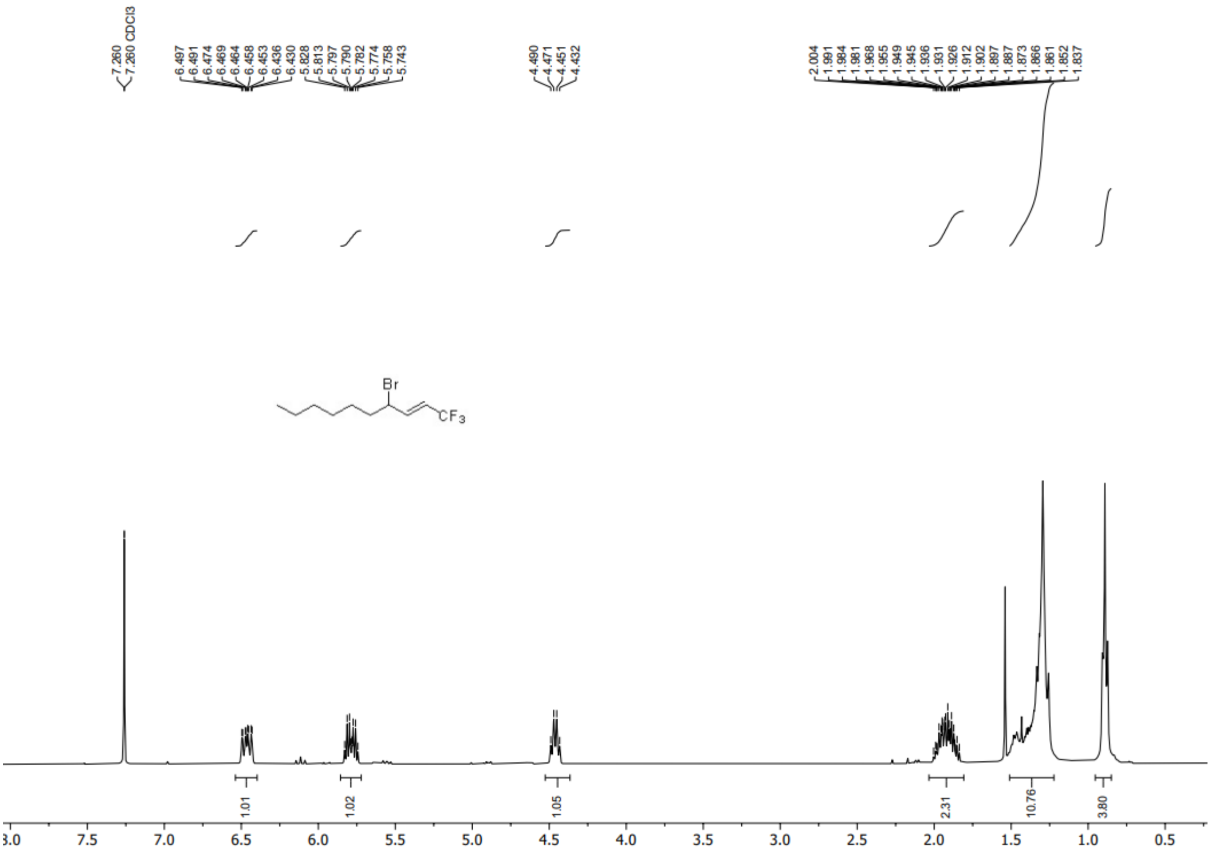

(*R*,*E*)-4-Bromo-1,1,1-trifluorodec-2-ene (**7a**) (^{13}C NMR)

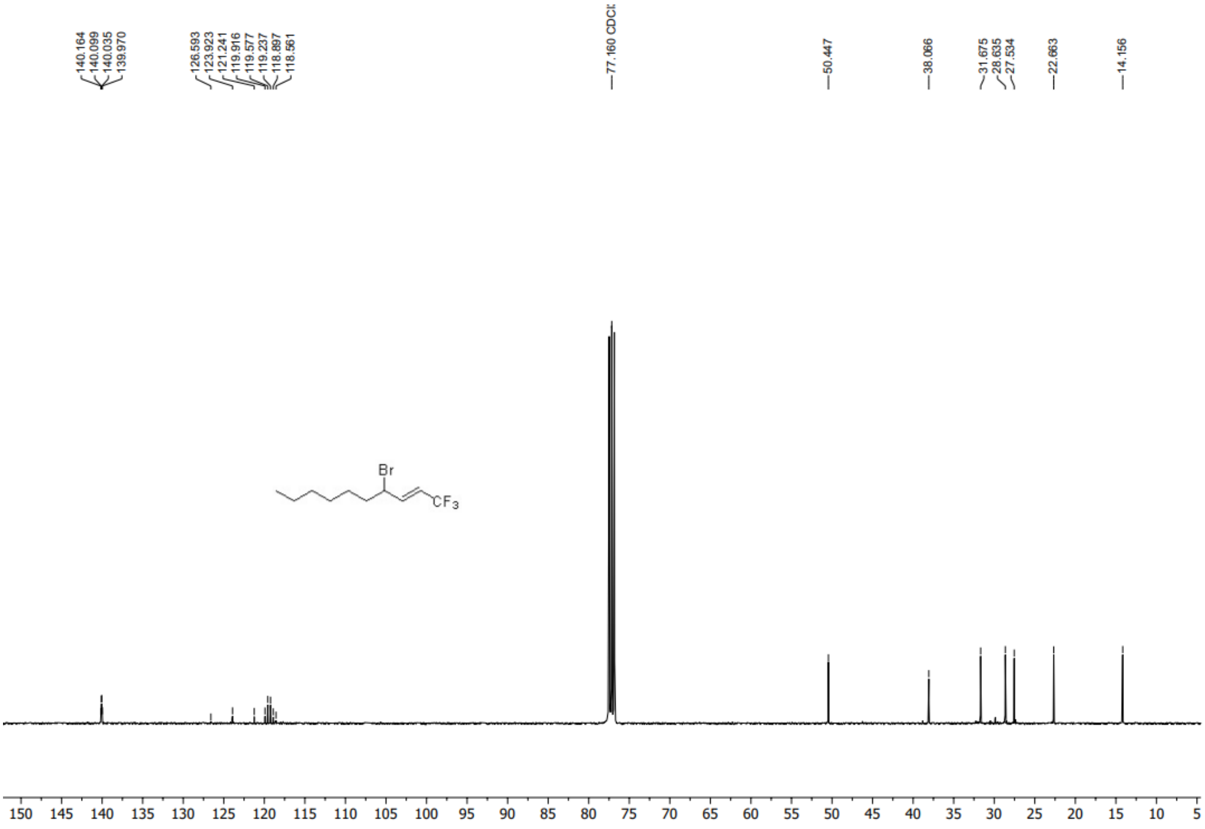

(*R*,*E*)-4-Bromo-1,1,1-trifluorodec-2-ene (**7a**) (^{19}F NMR)

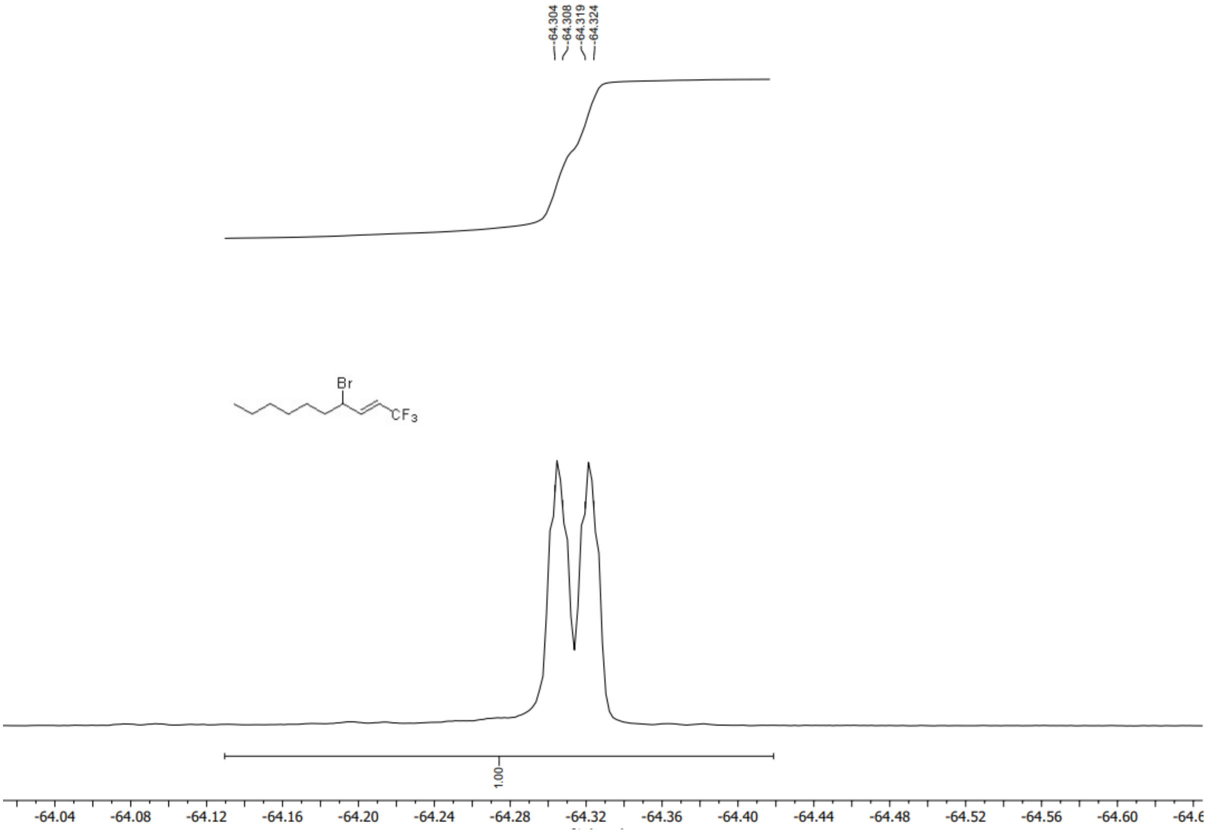

(*S*,*E*)-2-(2-Bromo-5,5,5-trifluoropent-3-en-1-yl)isoindoline-1,3-dione (**7b**) (^{1}H NMR)

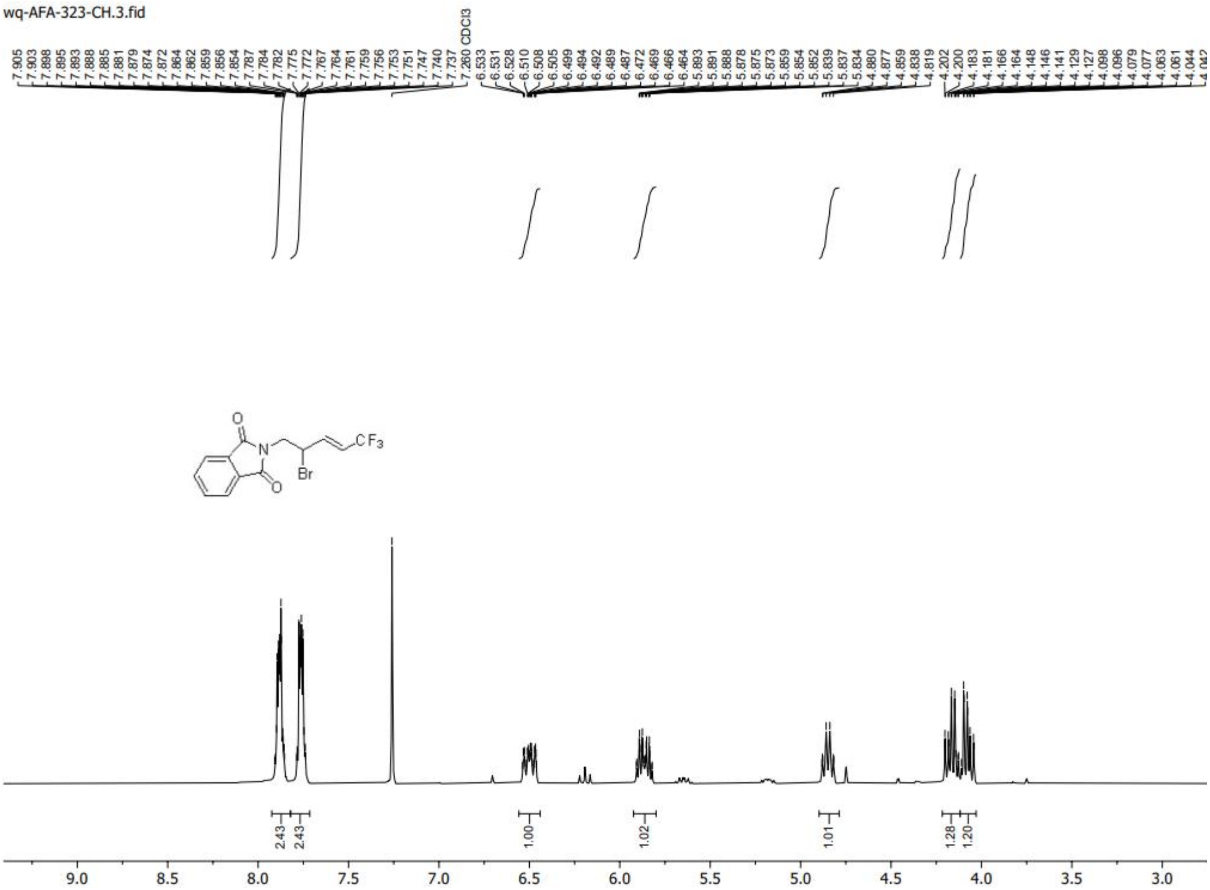

(*S*,*E*)-2-(2-Bromo-5,5,5-trifluoropent-3-en-1-yl)isoindoline-1,3-dione (**7b**) (^{13}C NMR)

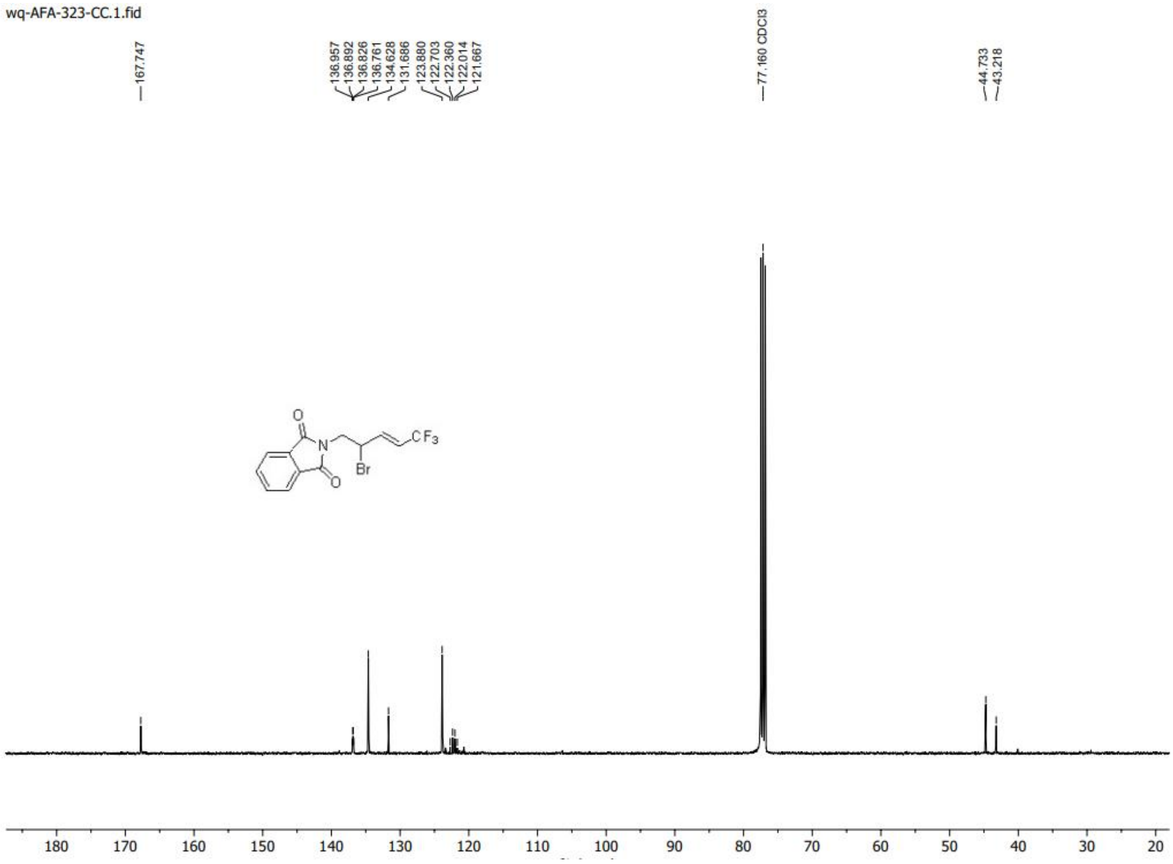

(*S*,*E*)-2-(2-Bromo-5,5,5-trifluoropent-3-en-1-yl)isoindoline-1,3-dione (**7b**) (^{19}F NMR)

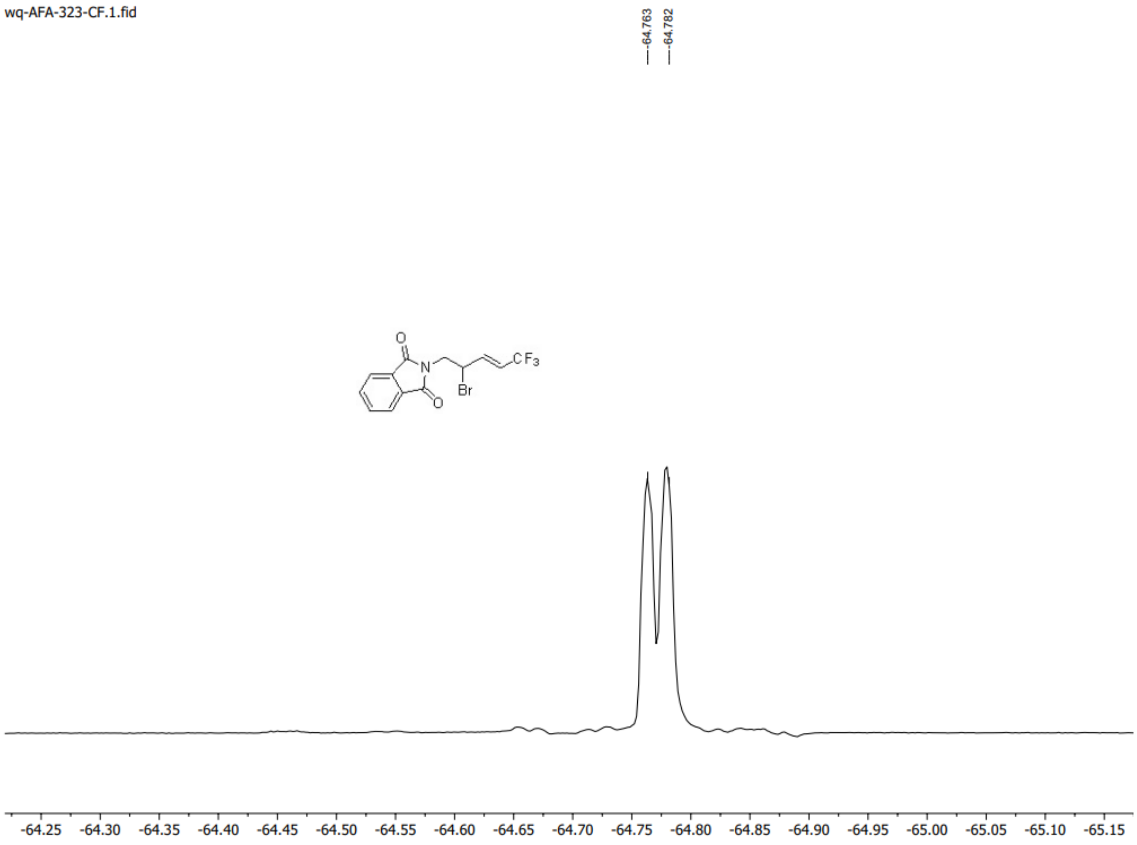

(*S*,*E*)-2-(2-Chloro-5,5,5-trifluoropent-3-en-1-yl)isoindoline-1,3-dione (**7c**) (^{1}H NMR)

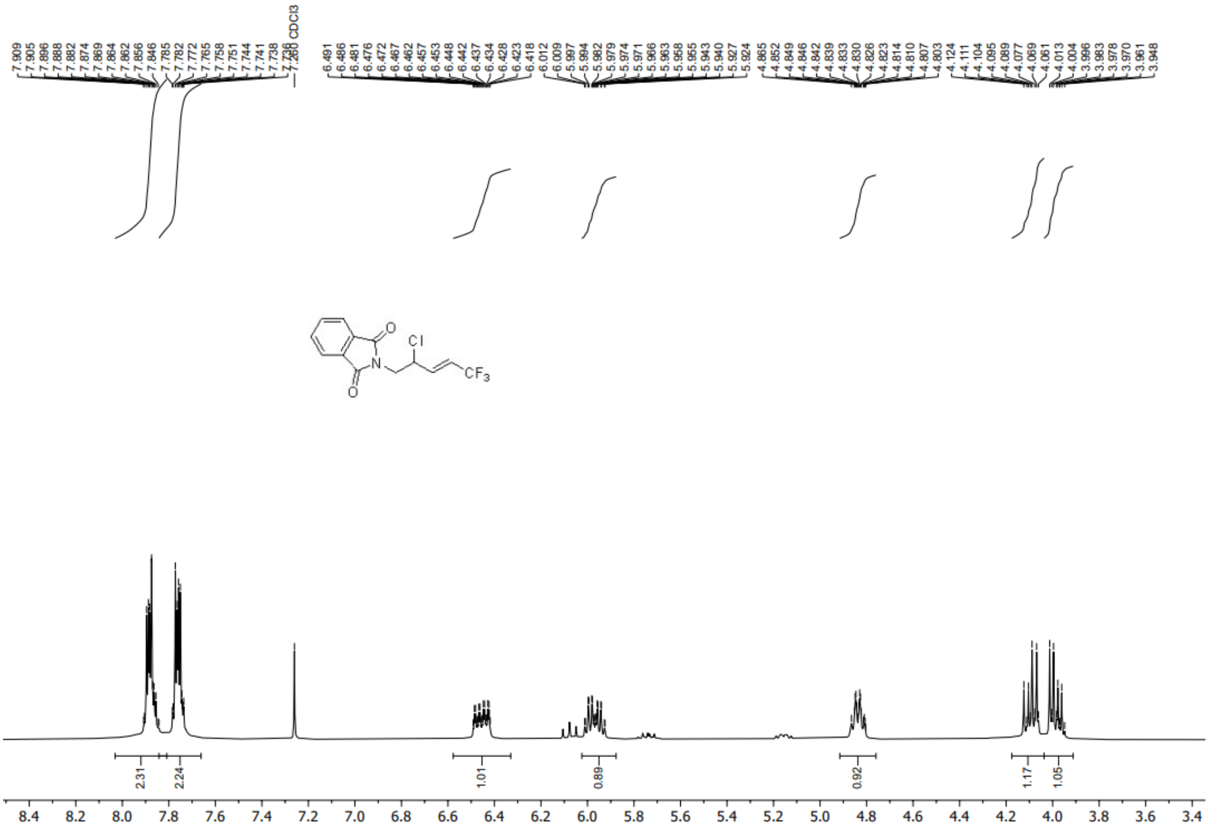

(*S*,*E*)-2-(2-Chloro-5,5,5-trifluoropent-3-en-1-yl)isoindoline-1,3-dione (**7c**) (^{13}C NMR)

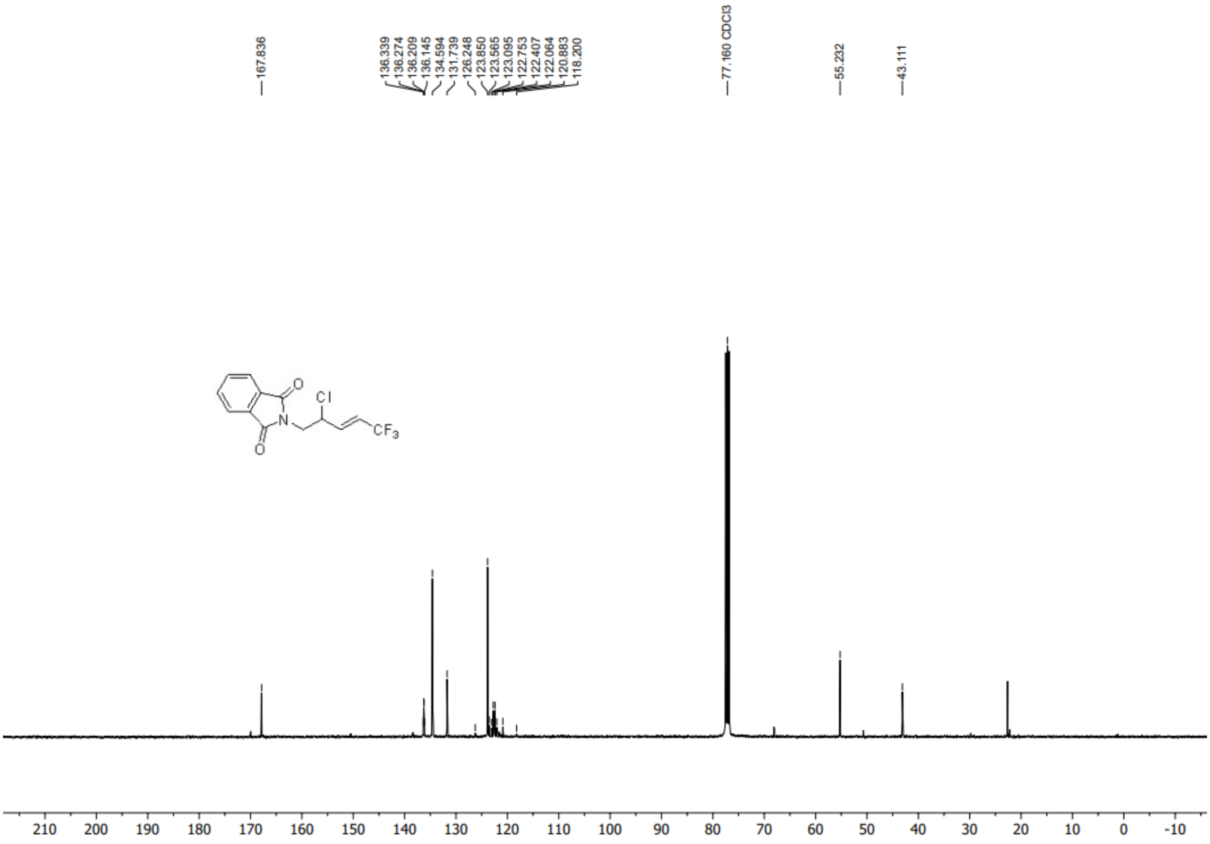

(*S*,*E*)-2-(2-Chloro-5,5,5-trifluoropent-3-en-1-yl)isoindoline-1,3-dione (**7c**) (^{19}F NMR)

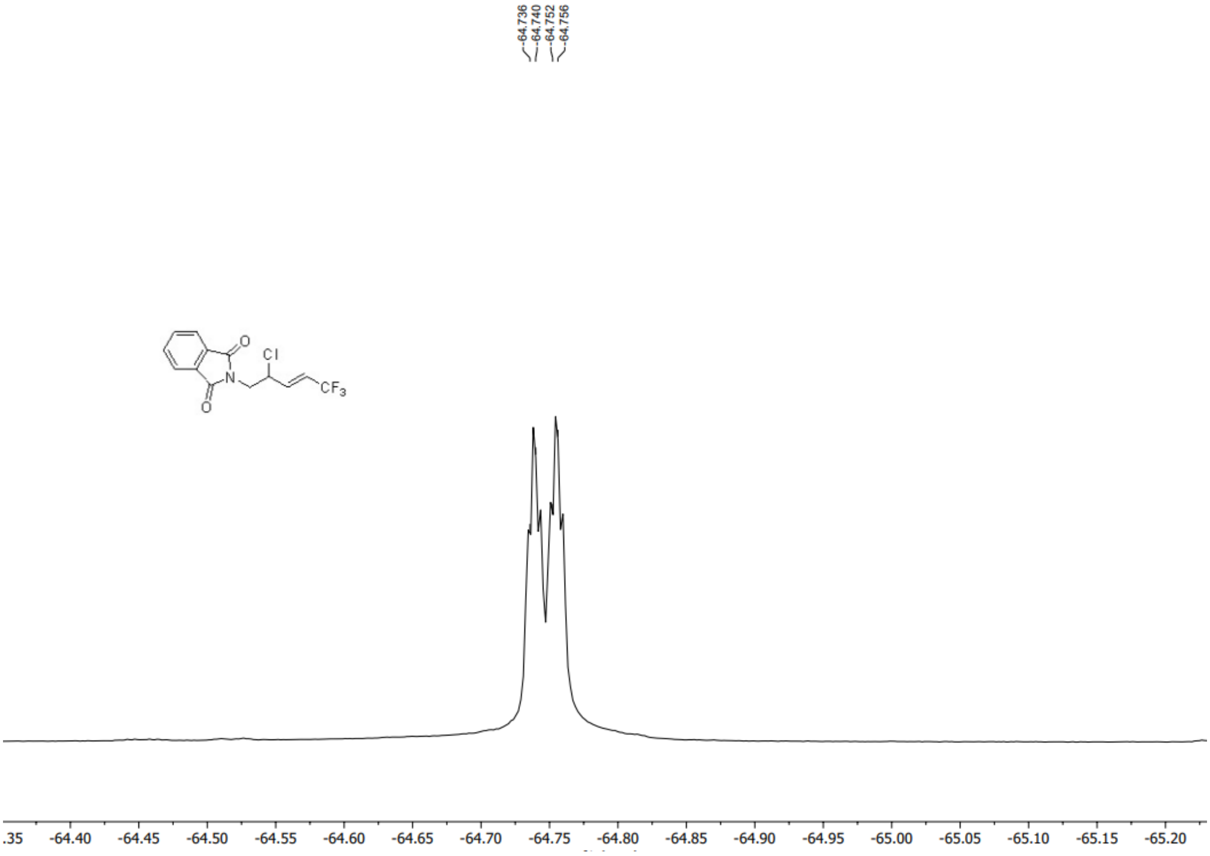

Reference

[1] P.-P. Zhang, M. Li, X.-S. Xue, C.-F. Xu, Q.-C. Zhao, Y.-F. Liu, H.-Y. Wang, Y.-L. Guo, L. Lu, Q.-L. Shen, *J. Org. Chem.* **2016**, *81*, 7486.

[2] E. Ismalaj, Q. Glenadel, T. Billard, *Eur. J. Org. Chem.* **2017**, 1911.

[3] E. Ismalaj, D. L. Bars, T. Billard, *Angew. Chem. Int. Ed.* **2016**, *55*, 4790.

[4] D. Wu, J.-S. Qiu, P. G. Karmaker, H.-Q. Yin, F.-X. Chen, *J. Org. Chem.* **2018**, *83*, 1576.

[5] F. Zhu, E. Miller, S.-Q. Zhang, D. Yi, S. O'Neill, X. Hong, M. A. Walczak, *J. Am. Chem. Soc.* **2018**, *140*, 18140.

[6] D. J. Winternheimer, C. A. Merlic, *Org. Lett.* **2010**, *12*, 2508.

[7] G. Pisella, A. Gagnebin, J. Waser, *Org. Lett.* **2020**, *22*, 3884.

[8] S. J. T. Jonker, R. Jayarajan, T. Kireilis, M. Deliaval, L. Eriksson, K. J. Szabó, *J. Am. Chem. Soc.* **2020**, *142*, 21254.

[9] O. A. Argintaru, D. Ryu, I. Aron, G. A. Molander, *Angew. Chem. Int. Ed.* **2013**, *52*, 13656.
